# Rhodium-catalyzed *ortho*-heteroarylation of phenols: directing group-enabled switching of the electronic bias for heteroaromatic coupling partner†
†Electronic supplementary information (ESI) available: Detailed information on experimental procedures, characterization data and crystallographic, and X-ray crystal structures (CIF) of CCDC 1837385 (**3n**). For ESI and crystallographic data in CIF or other electronic format see DOI: 10.1039/c8sc02529k


**DOI:** 10.1039/c8sc02529k

**Published:** 2018-07-18

**Authors:** Yimin Wu, Wei Li, Linfeng Jiang, Luoqiang Zhang, Jingbo Lan, Jingsong You

**Affiliations:** a Key Laboratory of Green Chemistry and Technology of Ministry of Education , College of Chemistry , Sichuan University , 29 Wangjiang Road , Chengdu 610064 , PR China . Email: jsyou@scu.edu.cn

## Abstract

The directed oxidative C–H/C–H cross-coupling reactions between a functionalized arene and a heteroarene typically exhibit an electronic bias for the heteroaromatic coupling partner. Disclosed herein is a conception of directing group enabled-switching of the electronic bias for coupling partner from the electron-deficient to electron-rich heteroarene, demonstrating that the modification of the directing group may match the latent reactivity of heteroarene substrates caused by the distinctly different electronic nature. In this work, we develop a Rh(iii)-catalyzed *ortho*-heteroarylation of phenols with greatly important electron-rich heteroarenes such as benzothiophene, benzofuran, thiophene, furan and pyrrole *via* two-fold C–H activation, which presents broad substrate scopes of both phenols and electron-rich heteroarenes and shows the advantage of tolerance of reactive functional groups, especially halogen. This work also provides a new strategy for the construction of π-conjugated furan-fused heteroacenes prevalent in materials science in dramatically simplified procedures, which makes the protocol highly applicable.

## Abstract 2

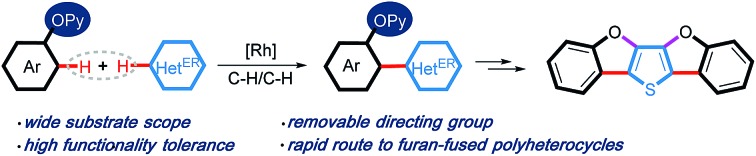
A highly efficient *ortho*-heteroarylation of phenols with diverse electron-rich heteroarenes has been developed to forge heteroaryl-2-hydroxyphenyl structural motifs. The removal of the directing group and subsequent intramolecular cyclization make this protocol applicable in the rapid construction of π-conjugated heteroacenes.

## Introduction

**Scheme 1:**
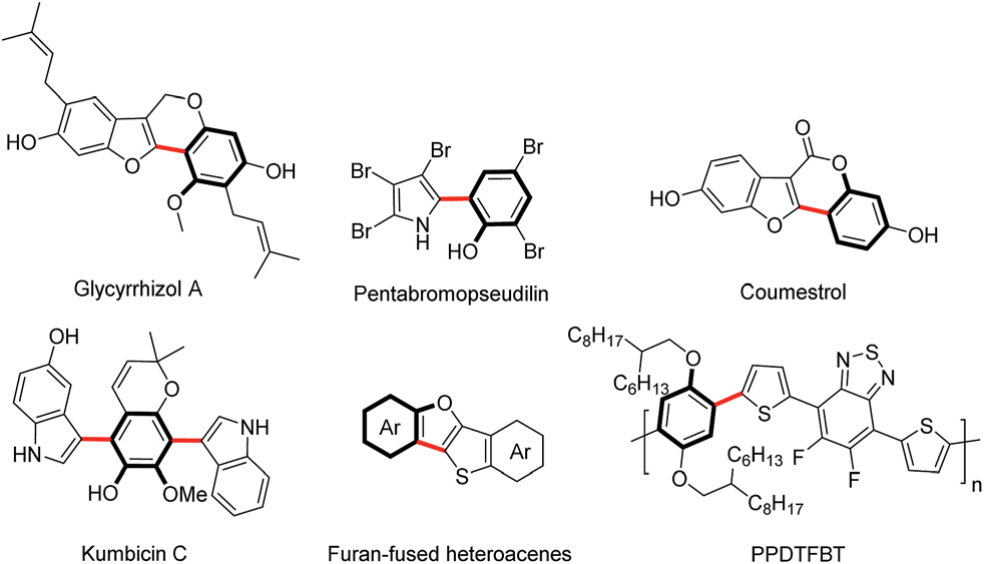
Selected examples of natural products, pharmaceuticals and organic functional material molecules.

Aryl-heteroaryl scaffolds containing 2-hydroxyphenyl units, especially for electron-rich heteroaryl-2-hydroxyphenyl, are prevalent in the field of natural products, pharmaceuticals and materials science (Scheme 1).^1^,^2^ Over the past decades, significant efforts have been devoted to develop various reliable and efficient methods for the synthesis of such structural linkages, including conventional transition metal-catalyzed C–X/C–M cross-coupling reactions^3^ and recently developed *ortho*-C–H (hetero)arylations of phenols with (hetero)arylating reagents such as organic halides and pseudohalides through the chelation assistance.^4^ From the viewpoint of step and atom economy, transition metal-catalyzed oxidative C–H/C–H cross-coupling would undoubtedly be one of the most straightforward routes to assemble these aryl-heteroaryl skeletons,^5^ which obviates wasteful prefunctionalization of coupling reactants and the use of organometallic reagents.

Although the past few years have witnessed tremendous advancements in the realm of C–H bond activation, there remain persistent obstacles associated with the latent reactivity of substrates and the regioselectivity issue. Recently, the directing group strategy has proved to be a very useful solution to such challenges through a cyclometalation.^6^ Through judicious choice of directing group, the modification of the coordinating capability as well as the ring size of forming metallacycle species enables to dramatically influence the reactivity of substrates caused by the distinctly different electronic nature and further match a variety of substrates, thus efficiently extending the compatibility of protocols for coupling reactants.

**Scheme 2:**
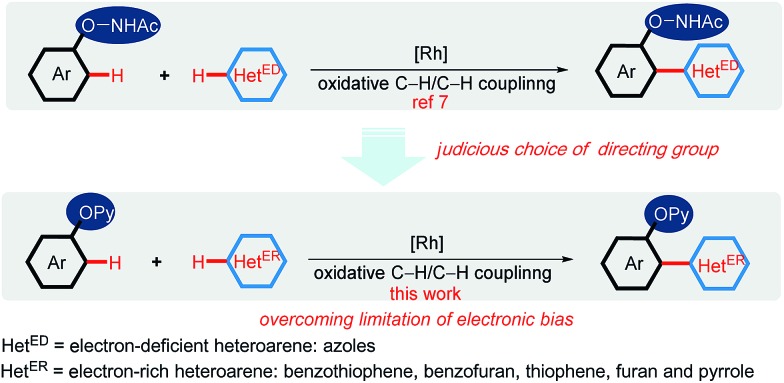
Directing group-enabled switching of the electronic bias for coupling partner from the electron-deficient to electron-rich heteroarenes.

Recent research has shown that the directed oxidative C–H/C–H cross-coupling reactions between a functionalized arene and a heteroarene typically exhibit an electronic bias for the heteroaromatic coupling partner. Using the oxyacetamide (O–NHAc) as the directing group, You's and Zhao's groups disclosed the Rh(iii)-catalyzed mono/bis-*ortho*-heteroarylation of phenols with electron-deficient heteroarenes such as azoles through internal and external oxidative C–H activation strategies, respectively (Scheme 2).^7^ However, transition metal-catalyzed oxidative C–H/C–H cross-coupling reactions between phenols and greatly important electron-rich heteroarenes, such as thiophene, furan and pyrrole, remain unsolved so far. Thus, it is required to develop an innovative system to overcome such a limitation caused by the distinctly different electronic nature of substrates. Herein, we wish to present a convenient strategy to achieve the switching of the electronic bias for coupling partner from the electron-deficient to electron-rich heteroarenes by judicious choice of directing group.

## Results and discussion

**Scheme 3:**
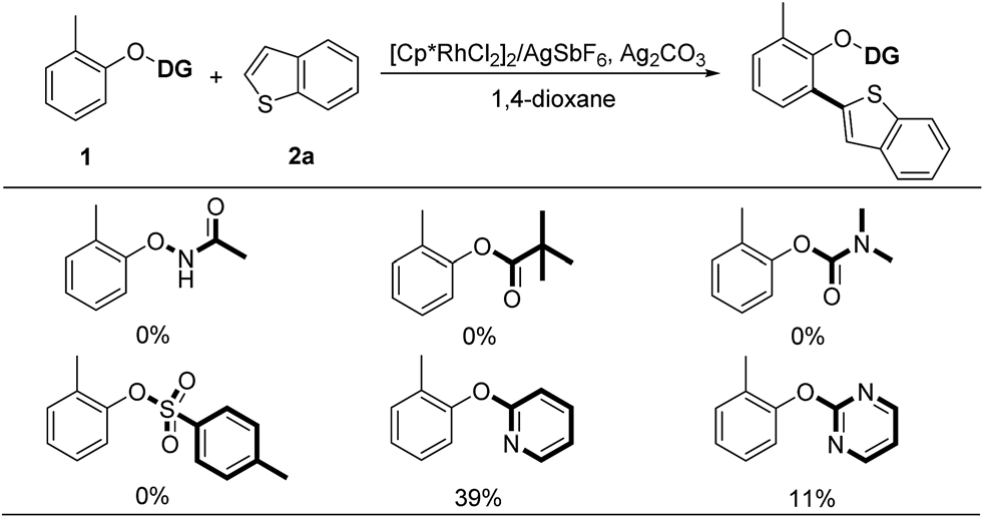
Judicious choice of directing group. Reaction conditions: **1** (0.20 mmol, 1.0 equiv.), **2a** (3.0 equiv.), [Cp*RhCl_2_]_2_ (5.0 mol%), AgSbF_6_ (20 mol%), Ag_2_CO_3_ (3.0 equiv.) and 1,4-dioxane (0.5 mL) at 150 °C under an N_2_ atmosphere for 24 h.

Considering the wide application of thiophene-based scaffolds,1e,^2^ we initially selected the cross-coupling between phenol derivatives (**1**) and electron-rich benzothiophene (**2a**) as a model reaction.^8^ At the outset of our investigation, we tested the feasibility of oxidative C–H/C–H cross-coupling between phenol and benzothiophene under the privileged [Cp*RhCl_2_]_2_/AgSbF_6_ catalytic system, using the commonly used *O*-carbamate, acyloxy, oxyacetamide and tosyl as the directing groups (Scheme 3). However, these directing groups were incapable of promoting the oxidative coupling reactions. Among various directing groups in C–H bond activation, the 2-pyridyl has exhibited a powerful potential because of its impressive coordination ability to transition metal center.^9^ Furthermore, the 2-pyridinyl group is readily introduced to phenols and the resulting 2-pyridyloxyl moiety is easily cleavable to produce the free phenol.4c,4d,^9^ Thus, we turned to investigate the 2-pyridyl as the directing group. It was delighted to observe that the desired *ortho*-heteroarylated product could be obtained in 39% yield (Scheme 3).

Subsequently, we tried to screen several parameters as shown in Tables S1–S4.† Among the oxidants investigated, we found that copper salts were superior to other inorganic salts (Table S1†). Among the solvents used, 1,4-dioxane was the best choice and a concentrated reaction system was more efficient (Table S2†). Further optimization of additives showed that the combination of acid with base could improve the yield of **3a** (Table S4†). Finally, the reaction delivered **3a** in 82% yield when [Cp*RhCl_2_]_2_ (5 mol%) was used in combination with AgSbF_6_ (20 mol%), PivOH (1.0 equiv.), CsOPiv (30 mol%) and Cu(OAc)_2_ (3.0 equiv.) in 1,4-dioxane at 150 °C for 24 h (Table S4,† entry 6).

**Scheme 4:**
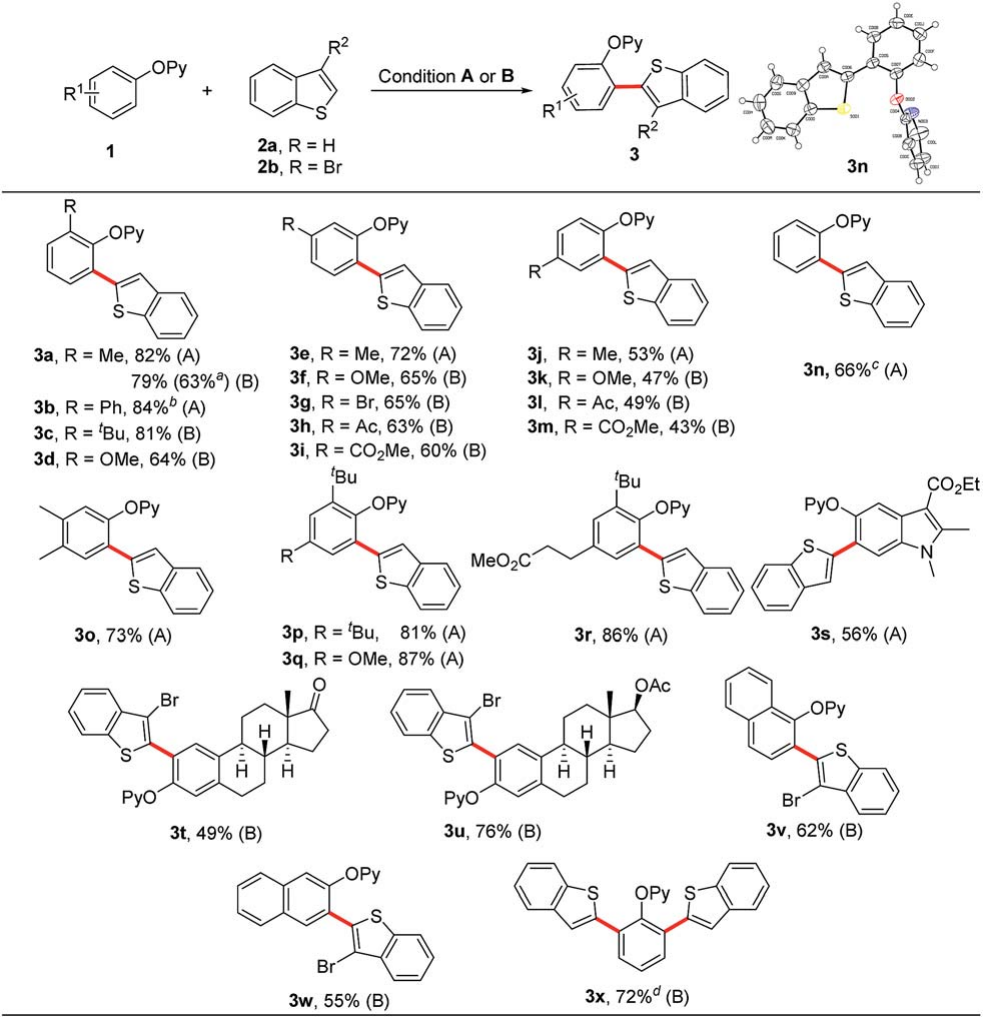
Scope of phenol derivatives. Reaction conditions (A) **1** (0.20 mmol), **2a** (3.0 equiv.), [Cp*RhCl_2_]_2_ (5 mol%), AgSbF_6_ (20 mol%), Cu(OAc)_2_ (3.0 equiv.), PivOH (1.0 equiv.), CsOPiv (30 mol%) and 1,4-dioxane (0.5 mL) at 150 °C under an N_2_ atmosphere for 24 h. Reaction conditions (B) **1** (0.20 mmol), **2a** or **2b** (2.0 equiv.), [Cp*RhCl_2_]_2_ (5 mol%), AgSbF_6_ (20 mol%), Ag_2_O (2.0 equiv.), Zn(OTf)_2_ (30 mol%) and 1,4-dioxane (0.5 mL) at 100 °C under an N_2_ atmosphere for 24 h. ^*a*^4.0 mmol scale reaction. ^*b*^AgSbF_6_ (40 mol%). ^*c*^Extra addition of Ag_2_CO_3_ (20 mol%). ^*d*^**2a** (3.0 equiv.) and Ag_2_O (3.0 equiv.).

With the optimal system in hand, we assessed the scope of this coupling reaction. As shown in Scheme 4, a variety of phenol substrates reacted efficiently with benzothiophene to give the corresponding *ortho*-heteroarylated products in moderate to high yields. For the *meta*-substituted and 3,4-disubstituted phenols, the heteroarylation occurred at the less hindered site (Scheme 4, **3e–3i** and **3o**). It is emphasized that the phenols bearing a more bulky group at the *ortho* position such as *tert*-butyl could also deliver the coupled products in good yields (Scheme 4, **3c** and **3p–3r**). In the presence of extra Ag_2_CO_3_ (20 mol%), the *ortho*-unsubstituted 2-phenoxypyridine could selectively undergo the *ortho*-heteroarylation to afford the monosubstituted product **3n** in 66% yield without observation of the diarylated product **3x** (Scheme 4, **3n**). The symmetrical bis-heteroarylated product could also be obtained in one step in 72% yield using Ag_2_O as the oxidant and Zn(OTf)_2_ as the additive at 100 °C (Scheme 4, **3x**). It is worthy of note that the reaction of **1a** with **2a** could be carried out on a 4.0 mmol scale with an acceptable yield of 63% (Scheme 4, **3a**), thus demonstrating the applicability of this method for mass production. Gratefully, phenol-containing natural products and pharmaceuticals such as estrone, estradiol and mecarbinate and naphthalenol derivatives smoothly underwent the *ortho*-heteroarylation, providing the corresponding cross-coupled products in satisfactory yields (Scheme 4, **3s–3w**).

**Scheme 5:**
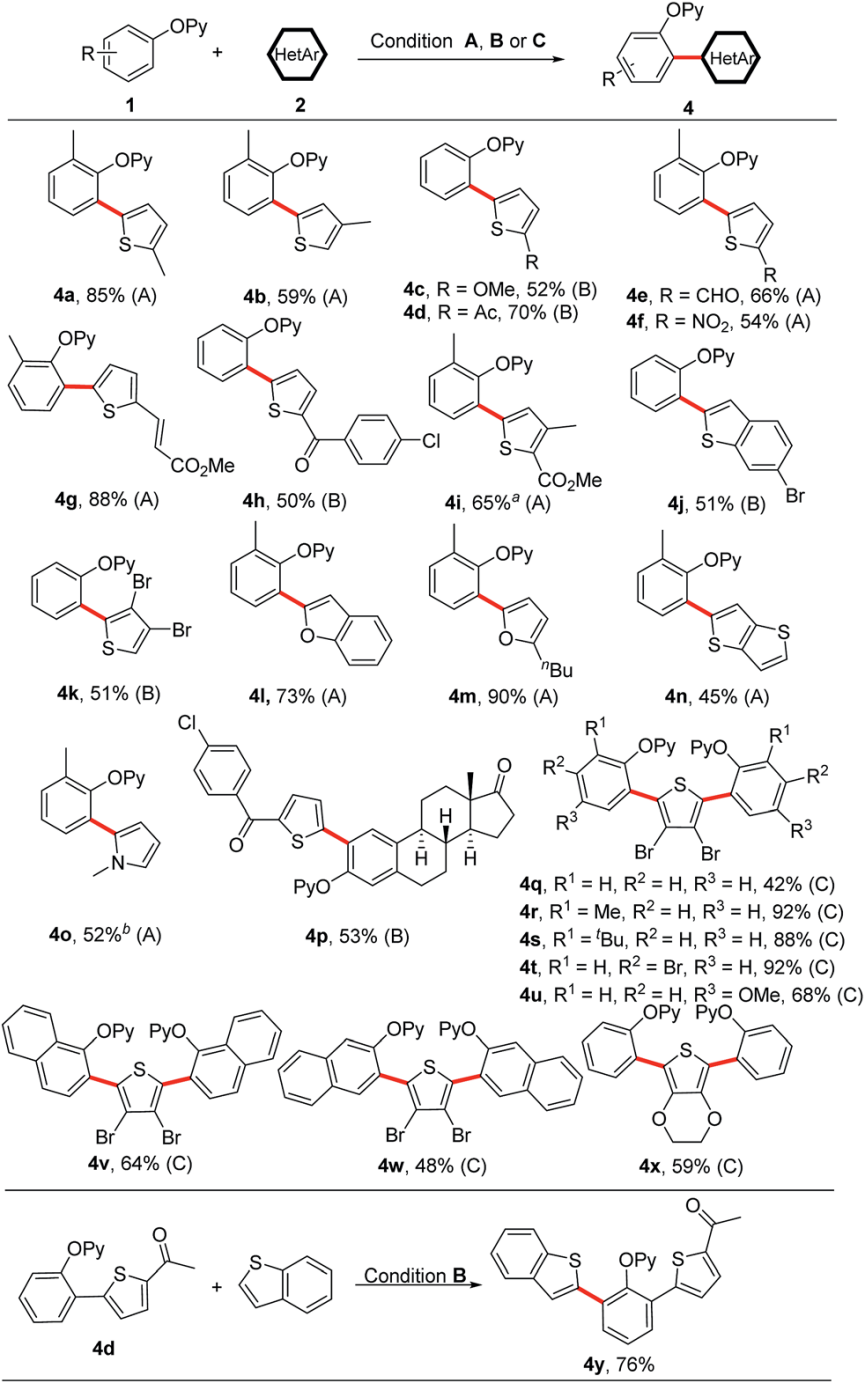
Scope of heteroarenes. Reaction conditions (A) **1** (0.20 mmol), **2** (3.0 equiv.), [Cp*RhCl_2_]_2_ (5 mol%), AgSbF_6_ (20 mol%), Cu(OAc)_2_ (3.0 equiv.), PivOH (1.0 equiv.), CsOPiv (30 mol%) and 1,4-dioxane (0.5 mL) at 150 °C under an N_2_ atmosphere for 24 h. Reaction condition (B) **1** (0.20 mmol), **2** (2.0 equiv.), [Cp*RhCl_2_]_2_ (5 mol%), AgSbF_6_ (20 mol%), Ag_2_O (2.0 equiv.), Zn(OTf)_2_ (30 mol%) and 1,4-dioxane (0.5 mL) at 100 °C under an N_2_ atmosphere for 24 h. Reaction condition (C) **1** (0.60 mmol), **2** (0.20 mmol), [Cp*RhCl_2_]_2_ (10 mol%), AgSbF_6_ (40 mol%), Ag_2_O (4.0 equiv.), Zn(OTf)_2_ (60 mol%) and 1,4-dioxane (0.5 mL) at 100 °C under an N_2_ atmosphere for 48 h. ^*a*^Without PivOH and CsOPiv. ^*b*^48 h.

Next, we turned our attention to heteroarene substrates (Scheme 5). To our delight, a broad range of electron-rich heteroarenes including benzothiophene, benzofuran, thiophene, furan and pyrrole engaged in this reaction in moderate to good yields (Scheme 5, **4a–4o**). Thiophenes bearing both the electron-donating and electron-withdrawing groups could afford the coupled products (Scheme 5, **4a–4i**). 3-Methylthiophene participated in the coupling reaction exclusively at the less sterically hindered 5-position (Scheme 5, **4b**). The synthetic utility of this protocol is underscored by its tolerance towards a wide range of reactive functionalities such as chloro, bromo, formyl, nitro, acetyl, ester, alkenyl and methoxy groups, which easily undergo further transformations. Even 3,4-dibromothiophene could also furnish the coupled product **4k** in 51% yield. Especially, starting from 3,4-dibromothiophene, an array of symmetrical 2,5-diaryl thiophenes could be successfully obtained in moderate to excellent yields in one-pot synthesis (Scheme 5, **4q–4x**). Thieno[3,2-*b*]thiophene and *N*-methyl pyrrole were also suitable substrates, delivering the coupled products in synthetically useful yields (Scheme 5, **4n** and **4o**). In addition, it is grateful to obtain the coupled product **4p**, which supports the robustness of our protocol in the functionalization of steroid derivatives. Treatment of **4d** with benzothiophene further yielded the unsymmetrical diheteroaryl-substituted product **4y**. Notably, while a broad range of electron-rich heteroarenes smoothly underwent the coupling reaction, the electron-deficient heteroarenes did not provide any desired products, which makes the reaction complementary to the previously reported protocols by You's and Zhao's groups.

**Scheme 6:**
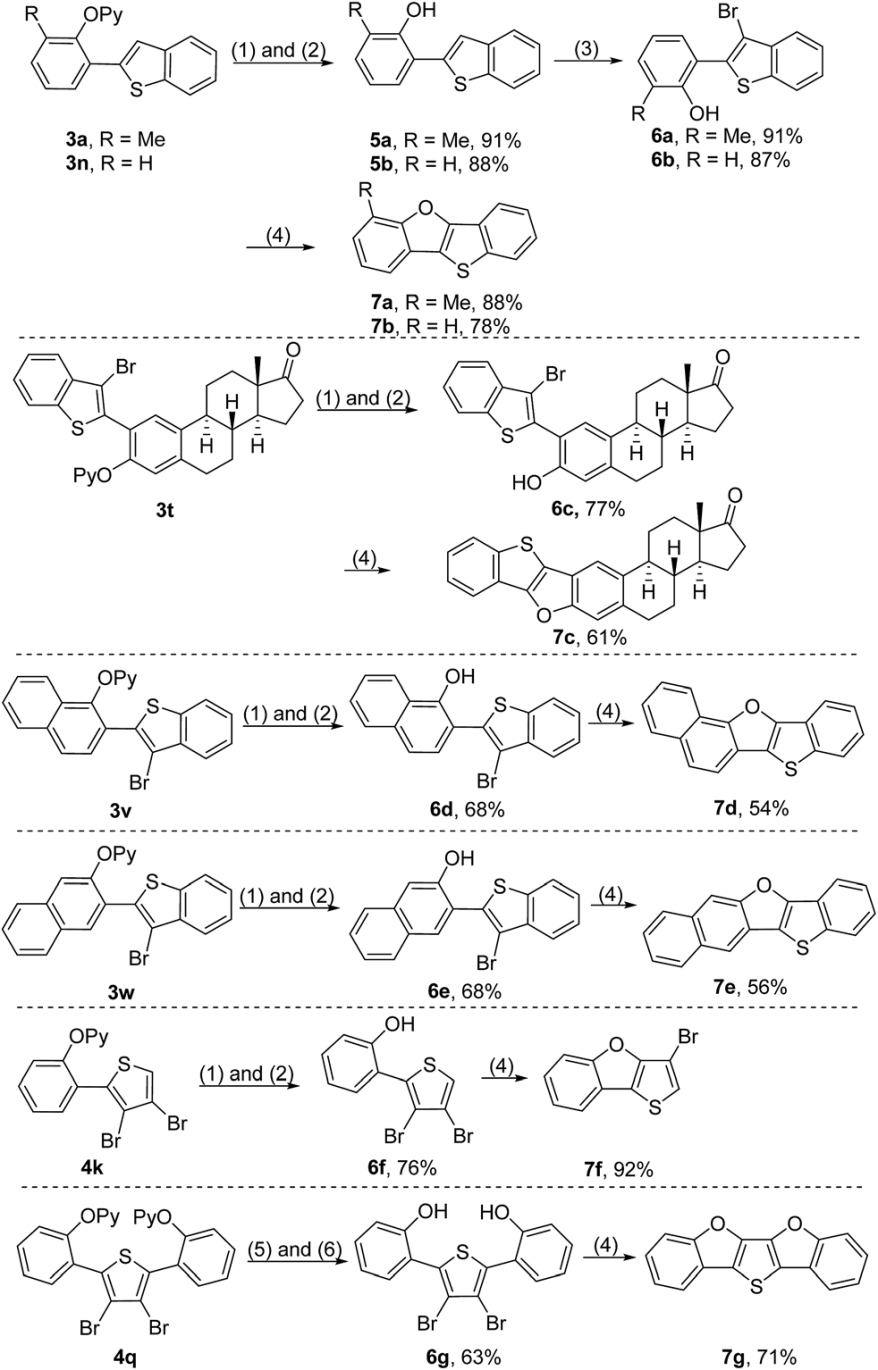
Construction of furan-fused heteroacenes. Reaction conditions: (1) MeOTf and toluene at 100 °C for 2 h. (2) Na/MeOH under reflux for 30 min. (3) NBS at r.t. for 18 h. (4) CuO, K_2_CO_3_ and pyridine under reflux. (5) MeOTf and toluene at 100 °C for 18 h. (6) Na/MeOH under reflux for 6 h.

**Scheme 7:**
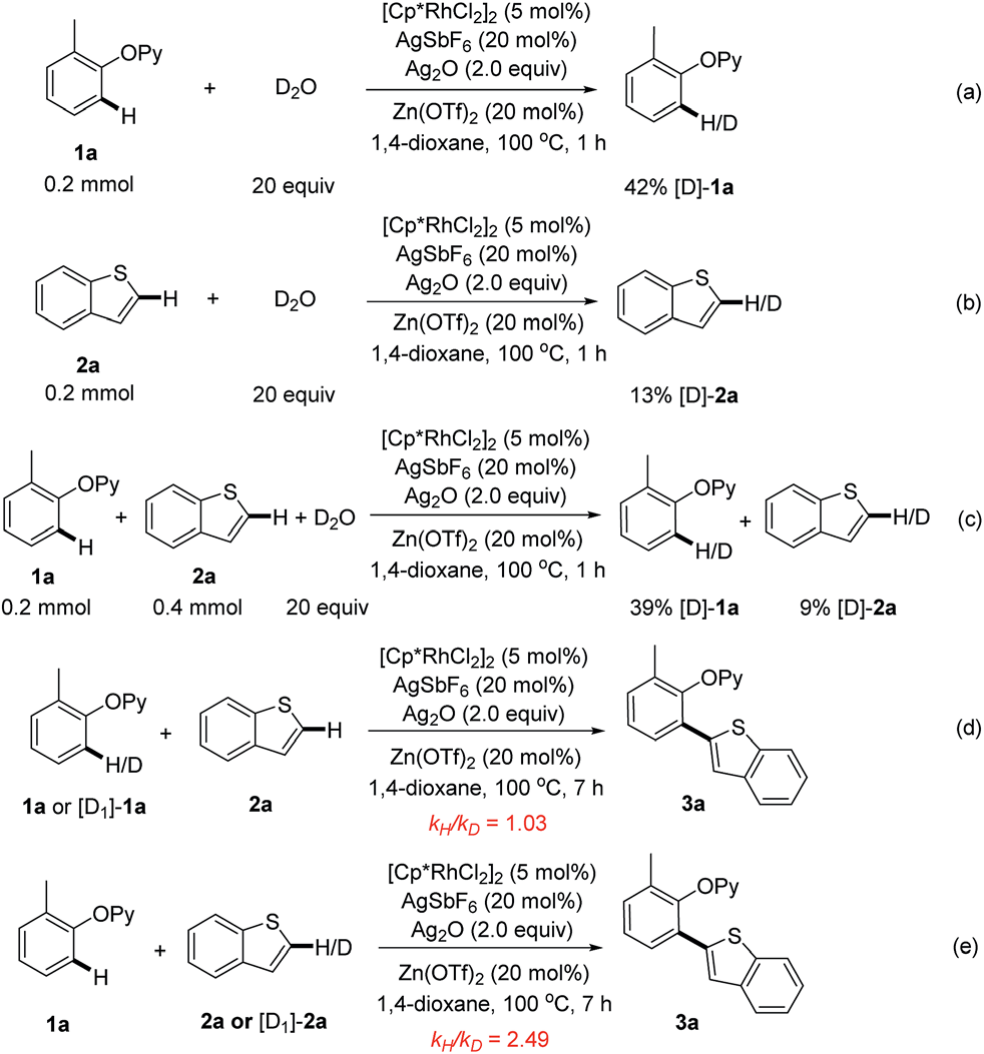
Deuterium-labeling and kinetic isotope effect experiments.

In order to further illustrate the synthetic utility of this protocol, a series of transformations of the resultant *ortho*-heteroarylated phenol derivatives were explored. The 2-pyridyl group of the coupled products could be removed by sequential treatment with methyl trifluoromethanesulfonate (MeOTf) in dry toluene and a refluxing Na/MeOH solution, producing 2-heteroarylated phenols (Scheme 6).4c,4d,^9^ Considering that extended π-conjugated furan-fused heteroacenes are promising functional organic materials for organic field-effect transistors (OFETs), organic light-emitting diodes (OLEDs) and liquid crystals,^2^ we attempted to convert *ortho*-heteroarylated phenols to furan-fused heteroarenes. Starting from **3a** and **3n**, benzo[4,5]thieno[3,2-*b*]benzofurans were rapidly constructed by sequential removal of the directing group, bromination and intramolecular cyclization in satisfactory total yields (Scheme 6, **7a** and **7b**). Because the oxidative coupling reaction developed herein enables tolerance of the reactive bromide groups, the direct use of bromo-substituted thiophenes as a starting material could significantly streamline synthetic routes (Scheme 6, **7c–7g**). For example, starting from diarylated 3,4-dibromothiophene **4q**, a new extended π-conjugated molecule, thieno[3,2-*b*:4,5-*b*′]bis[1]benzofuran (**7g**), could be constructed rapidly, highlighting the charm of this protocol in the construction of furan-fused heteroacenes.

Finally, for insight into this rhodium-catalyzed reaction, the H/D exchange control experiments were conducted. When 2-(*o*-tolyloxy)pyridine (**1a**) was treated with D_2_O for 1 h under the optimized catalytic conditions, 42% of **1a** was deuterated (Scheme 7a), while a 13% H/D scrambling was observed with benzothiophene (**2a**) (Scheme 7b). The exposure of **1a** and **2a** to D_2_O could produce similar ratios of deuterated products (Scheme 7c). These observations implied that the C–H activation processes of both **1a** and **2a** could be reversible. Next, the kinetic isotope effect (KIE) experiments were performed. A KIE value of 1.03 for **1a** was obtained while 2.49 was observed for **2a** (Scheme 7d and 7e), indicating that the C2–H cleavage of benzothiophene might be involved in the rate-limiting step. Based on the above results and the previously reported literature,^10^ we speculated that a tentative mechanism could consist of (1) the coordination of the 2-phenoxypyridine to [Cp*Rh(iii)] and subsequent *ortho*-C–H activation of arene, (2) the reaction of the resulting rhodacycle intermediate with a heteroarene to form the key aryl-Rh(iii)-heteroaryl, (3) the reductive elimination to deliver the *ortho*-heteroarylated product, and (4) the re-oxidation of Rh(i) to close the catalytic cycle (Scheme S2†).

## Conclusions

In summary, we have developed a rhodium-catalyzed *ortho*-heteroarylation of phenols with greatly important electron-rich heteroarenes such as benzothiophene, benzofuran, thiophene, furan and pyrrole *via* two-fold C–H activation. This protocol features the readily installed and removable directing group, broad substrate scope and excellent functional group tolerance. From oxyacetamide to 2-pyridyloxyl as the directing group,^7^ the scope of coupling substrates switches from electron-deficient to electron-rich heteroarenes, which would bring inspiration for the solution of the electronic bias of heteroaromatic coupling partner in the directed oxidative C–H/C–H cross-coupling reactions between two (hetero)arenes. Additionally, the coupled products can further be transformed to furan-fused heteroacenes in good yields, which makes the method highly applicable.

## Conflicts of interest

There are no conflicts to declare.

## Supplementary Material

Supplementary information

Crystal structure data
