# Supplementary material for: Rhodium-catalyzed ortho-heteroarylation of phenols: directing group-enabled switching of the electronic bias for heteroaromatic coupling partner
Source: Chem Sci. 2018 Jul 18;9(33):6878–82. doi: 10.1039/c8sc02529k (PMC6114993; doi:10.1039/c8sc02529k)
Supplement: Supplementary file 1 [file SC-009-C8SC02529K-s001.pdf]

Electronic Supplementary Material (ESI) for Chemical Science.
This journal is © The Royal Society of Chemistry 2018

Supporting Information

Rhodium-catalyzed *ortho*-heteroarylation of phenols: directing group-enabled switching of the electronic bias for heteroaromatic coupling partner

Yimin Wu,[a] Wei Li,[a] Linfeng Jiang,[a] Luoqiang Zhang,[a] Jingbo Lan[a] and Jingsong You*[a]

[a]Key Laboratory of Green Chemistry and Technology of Ministry of Education, College of Chemistry, Sichuan University, 29 Wangjiang Road, Chengdu 610064, China

*E-mail: jsyou@scu.edu.cn

Table of contents

I. General remarks…………………………………………………………………S3

II. General procedure for the synthesis of 2-phenoxypyridine derivatives….….S3

III. Screening of directing groups……………..…….……………….…...………...S4

IV. Optimization of the oxidative *ortho*-heteroarylation of phenol derivatives.......S4

V. General procedure for the oxidative *ortho*-heteroarylation of phenol derivatives…..………………………………………………………….……….…...S6

VI. Gram-scale synthesis of **3a**……………………….……………………….…...…S7

VII. Procedure for removal of the directing group……………………….……......…....S8

VIII. Construction of furan-fused heteroacenes…………………………….…..S12

IX. Mechanistic study……………...……………………….………………….....…S17

X. Single-crystal X-ray structure of **3n**………..……….……….....…..…...S21

XI. Experimental data for the described substances……………..….……………...S22

XII. References………………………...S44

XIII. Copies of ^{1}H and ^{13}C NMR spectra…………………….................................S46

I. General remarks

NMR spectra were recorded on a Agilent 400-MR DD2 or a Bruker AMX-400MHz spectrometer. The ^{1}H NMR (400 MHz) chemical shifts were measured relative to $CDCl_3$ or DMSO-d_6 as the internal reference (DMSO-d_6: δ = 2.50 ppm; $CDCl_3$: δ = 7.26 ppm). The ^{13}C NMR (100 MHz) chemical shifts were given using $CDCl_3$ or DMSO-d_6 as the internal standard (DMSO-d_6: δ = 39.52 ppm; $CDCl_3$: δ = 77.16 ppm). High-resolution mass spectra (HRMS) were obtained with a Shimadzu LCMS-IT-TOF (ESI) or a Waters-Q-TOF-Premier (ESI). X-Ray single-crystal diffraction data were collected on an Oxford Xcalibur E single crystal diffractometer. Melting points were determined with XRC-1 and are uncorrected.

All reagents were obtained from commercial suppliers and used without further purification unless otherwise stated. $RhCl_3{\cdot}3H_2O$ was purchased from Shaanxi Kaida Chemical Engineering (China) CO., Ltd. $AgSbF_6$ and $Cu(OAc)_2$ were purchased from Alfa Aesar. $[Cp^*RhCl_2]_2$[1] and 2-phenoxypyridine derivatives[2] were prepared according to the literature procedures. All solvents were dried with an innovative technology solvent purification system (model no.: PS-MD-5). All reactions were carried out under a nitrogen atmosphere.

II. General procedure for the synthesis of 2-phenoxypyridine derivatives[2]

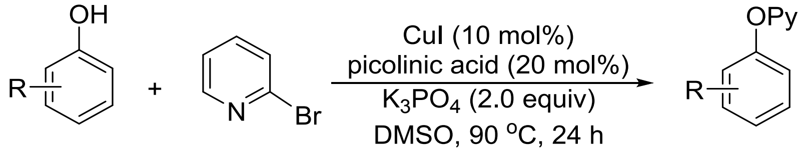

A 100 mL oven-dried round bottomed flask was charged with a magnetic stiring bar, CuI (190 mg, 1.0 mmol, 10 mol%), picolinic acid (246 mg, 2.0 mmol, 20 mol%), phenol (11 mmol), and K_3PO_4 (4.24 g, 20 mmol). The tube was then evacuated and back-filled with N_2. The procedure of evacuation/backfill was sequentially repeated two additional times. It was then added with 2-bromopyridine (10 mmol) and dimethylsulfoxide (25 mL) by syringe under an N_2 atmosphere. The tube was placed in a pre-heated oil bath at 90 ºC and the reaction mixture was stirred for 24 h. The reaction mixture was cooled to room temperature and quenched with water (20 mL). CH_2Cl_2 (30 mL) was added and the mixture was stirred. The organic layer was separated and the aqueous layer was extracted twice more with dichloromethane (10 mL). Combined organic layer was washed with water and dried over Na_2SO_4. After removal of the solvent under reduced pressure, the residue was purified via silica gel

column using petromleum ester and ethyl acetate.

III. Screening of directing groups

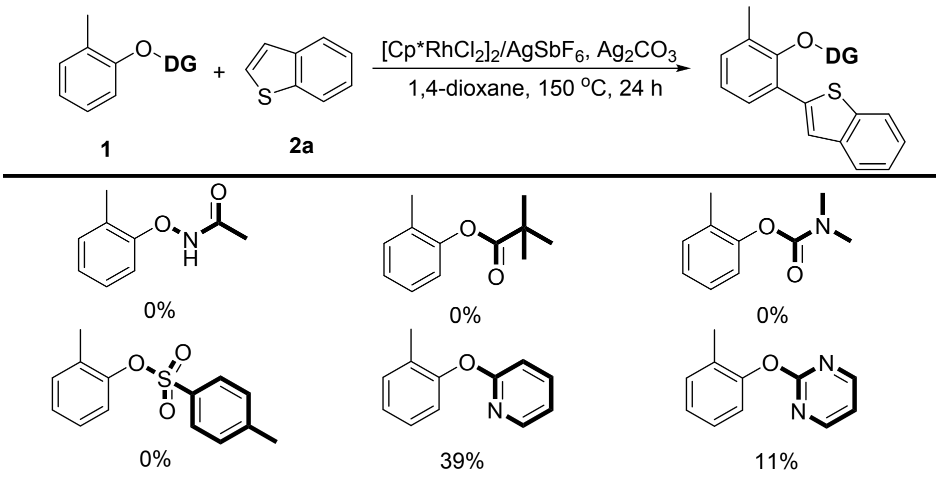

Scheme S1. Screening of directing groups

A flame-dried Schlenk test tube with a magnetic stirring bar was charged with phenol derivative (0.20 mmol), **2a** (3.0 equiv), $[Cp^*RhCl_2]_2$ (5.0 mol%), $AgSbF_6$ (20 mol%), Ag_2CO_3 (3.0 equiv), and 1,4-dioxane (0.5 mL). The reaction mixture was allowed to stir for 5 min at room temperature under an N_2 atmosphere, and then heated at 150 °C in a pre-heated oil bath for 24 h. The reaction mixture was then cooled to room temperature, diluted with 10 mL of CH_2Cl_2, filtered through a celite pad, and washed with 25-35 mL of CH_2Cl_2. The combined organic extracts were concentrated under reduced pressure and the resulting residue was purified by column chromatography on silica gel to provide the desired product.

IV. Optimization of the oxidative *ortho*-heteroarylation of phenol derivatives

A flame-dried Schlenk test tube with a magnetic stirring bar was charged with 2-(*o*-tolyloxy)pyridine (**1a**, 0.20 mmol), benzothiophene (**2a**, 3.0 equiv), the catalyst (5.0-10 mol%), $AgSbF_6$ (20 mol%), oxidant (3.0 equiv), acid (1.0 equiv), base (30 mol%), and solvent. The reaction mixture was allowed to stir for 5 min at room temperature under an N_2 atmosphere, and then heated at 150 °C in a pre-heated oil bath for 24 h. The reaction mixture was then cooled to room temperature, diluted with 10 mL of CH_2Cl_2, filtered through a celite pad, and washed with 25-35 mL of CH_2Cl_2. The combined organic extracts were concentrated under reduced pressure and the resulting residue was purified by column chromatography on silica gel (petroleum ether/acetone/Et_3N = 120/5/1, v/v/v) to provide the desired product **3a**.

(1) *Table S1.* Screening of oxidant[a]

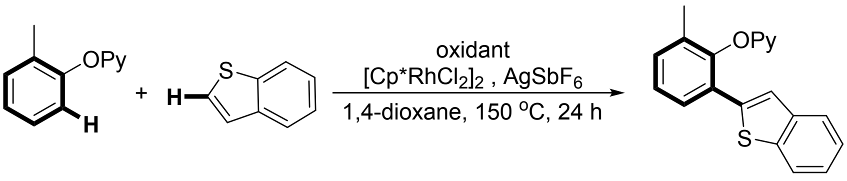

1a **2a** **3a**

Entry	Oxidant	Yield (%)[b]
1	Ag_2CO_3	39
2	AgOAc	16
3	$Cu(OAc)_2$	67
4	$Cu(OAc)_2.H_2O$	29
5	$Cu(OTf)_2$	trace
6	$CuCl_2$	n.d
7	CuI	n.d
8	BQ	trace
9	$PhI(OAc)_2$	trace
10	$K_2S_2O_8$	trace
11	$NaClO_3$	trace
12	O_2	n.d

[a]Reaction conditions: 2-(*o*-tolyloxy) pyridine **1a** (37.0 mg, 0.2 mmol), benzo[*b*]thiophene **2a** (80.4 mg, 0.6 mmol), $[Cp*RhCl_2]_2$ (5.0 mol%), $AgSbF_6$ (20 mol%), oxidant (3.0 equiv), and 1,4-dioxane (0.5 mL) at 150 °C for 24 h under an N_2 atmosphere. [b]Yields of isolated products. n.d = no detection.

Table S2. **Screening of solvent**[a]

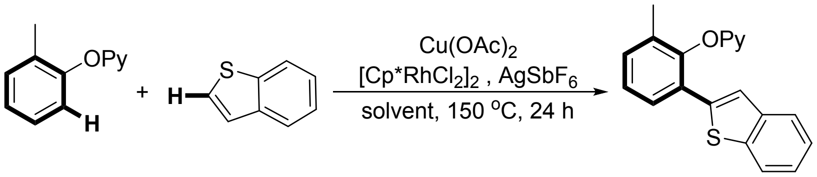

1a **2a** **3a**

Entry	Solvent	Yield (%)[b]
1	1,4-dioxane	68
2[c]	1,4-dioxane	49
3	THF	59
4	MeOH	n.d
5	DMF	21
6	DCE	22
7	toluene	51
8	*o*-xylene	30

[a]Reaction conditions: 2-(*o*-tolyloxy) pyridine **1a** (37.0 mg, 0.2 mmol), benzo[*b*]thiophene **2a**

(80.4 mg, 0.6 mmol), [Cp*$RhCl_2$]$_2$ (5.0 mol%), $AgSbF_6$ (20 mol%), $Cu(OAc)_2$ (3.0 equiv), and solvent (0.5 mL) at 150 °C for 24 h under an N_2 atmosphere. [b]Yields of isolated products. [c]1,4-Dioxane (1.0 mL). THF = tetrahydrogen furan. DMF = dimethyl formamide. DCE = 1,2-dichloroethane. n.d = no detection.

***Table S3.* Screening of rhodium catalyst**[a]

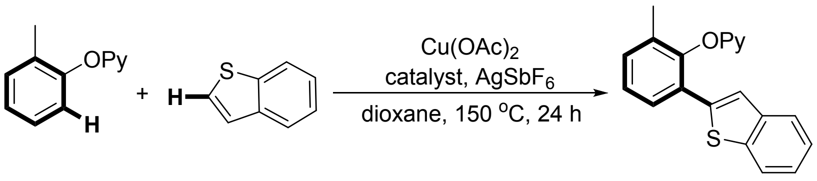

Entry	Catalyst (mol%)	Yield (%)[b]
1	-	n.d
2	[Cp*Rh(MeCN)$_3$][SbF_6]$_2$ (10)	59[c]
3	Rh(PPh_3)$_3$Cl (10)	42
4	[Rh(cod)Cl]$_2$ (5)	37
5	[Cp*$RhCl_2$]$_2$ (5)	68

[a]Reaction conditions: 2-(*o*-tolyloxy) pyridine **1a** (37.0 mg, 0.2 mmol), benzo[*b*]thiophene **2a** (80.4 mg, 0.6 mmol), catalyst (5.0-10 mol%), $AgSbF_6$ (20 mol%), $Cu(OAc)_2$ (3.0 equiv), and 1,4-dioxane (0.5 mL) at 150 °C for 24 h under an N_2 atmosphere. [b]Yields of isolated products. [c]Without $AgSbF_6$. n.d = no detection.

***Table S4.* Screening of additive**[a]

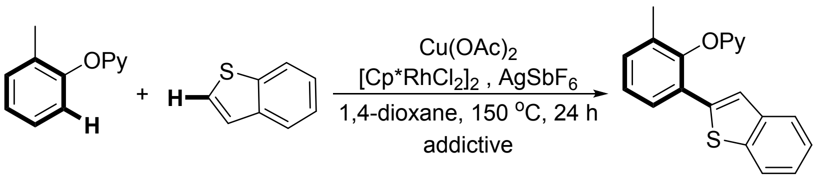

Entry	Additive	Yield (%)[b]
1	PivOH	78
2	AcOH	66
3	PivOH and CsOPiv	65
4	PivOH and KOAc	71
5	PivOH and AgOPiv	72
**6**	**PivOH and CsOPiv**	**82**

[a]Reaction conditions: 2-(*o*-tolyloxy) pyridine **1a** (37.0 mg, 0.2 mmol), benzo[*b*]thiophene **2a** (80.4 mg, 0.6 mmol), [Cp*$RhCl_2$]$_2$ (5.0 mol%), $AgSbF_6$ (20 mol%), $Cu(OAc)_2$ (3.0 equiv), acid (1.0 equiv) , base (30 mol%), and 1,4-dioxane (0.5 mL) at 150 °C for 24 h under an N_2 atmosphere. [b]Yields of isolated products.

V. General procedure for the oxidative *ortho*-heteroarylation of phenol derivatives

Condition A: A flame-dried Schlenk test tube with a magnetic stirring bar was charged with phenol derivative (0.20 mmol), heteroarene (3.0 equiv), $[Cp^*RhCl_2]_2$ (5.0 mol%), $AgSbF_6$ (20 mol%), $Cu(OAc)_2$ (3.0 equiv), PivOH (1.0 equiv) , CsOPiv (30 mol%), and 1,4-dioxane (0.5 mL). The reaction mixture was allowed to stir for 5 min at room temperature under an N_2 atmosphere, and then heated at 150 °C in a pre-heated oil bath for 24 h. The reaction mixture was then cooled to room temperature, diluted with 10 mL of CH_2Cl_2, filtered through a celite pad, and washed with 25-35 mL of CH_2Cl_2. The combined organic extracts were concentrated under reduced pressure and the resulting residue was purified by column chromatography on silica gel to provide the desired product.

Condition B: A flame-dried Schlenk test tube with a magnetic stirring bar was charged with phenol derivative (0.20 mmol), heteroarene (2.0 equiv), $[Cp^*RhCl_2]_2$ (5.0 mol%), $AgSbF_6$ (20 mol%), Ag_2O (2.0 equiv), $Zn(OTf)_2$ (30 mol%), and 1,4-dioxane (0.5 mL). The reaction mixture was allowed to stir for 5 min at room temperature under an N_2 atmosphere, and then heated at 100 °C in a pre-heated oil bath for 24 h. The reaction mixture was then cooled to room temperature, diluted with 10 mL of CH_2Cl_2, filtered through a celite pad, and washed with 25-35 mL of CH_2Cl_2. The combined organic extracts were concentrated under reduced pressure and the resulting residue was purified by column chromatography on silica gel to provide the desired product.

Condition C: A flame-dried Schlenk test tube with a magnetic stirring bar was charged with phenol derivative (0.60 mmol), heteroarene (0.20 mmol), $[Cp^*RhCl_2]_2$ (10.0 mol%), $AgSbF_6$ (40 mol%), Ag_2O (4.0 equiv), $Zn(OTf)_2$ (60 mol%), and 1,4-dioxane (0.5 mL). The reaction mixture was allowed to stir for 5 min at room temperature under an N_2 atmosphere, and then heated at 100 °C in a pre-heated oil bath for 48h. The reaction mixture was then cooled to room temperature, diluted with 10 mL of CH_2Cl_2, filtered through a celite pad, and washed with 25-35 mL of CH_2Cl_2. The combined organic extracts were concentrated under reduced pressure and the resulting residue was purified by column chromatography on silica gel to provide the desired product.

VI. Gram-scale synthesis of 3a

A flame-dried Schlenk test tube with a magnetic stirring bar was charged with **1a** (4.0 mmol), **2a** (2.0 equiv), $[Cp^*RhCl_2]_2$ (5.0 mol%), $AgSbF_6$ (20 mol%), Ag_2O (2.0

equiv), $Zn(OTf)_2$ (30 mol%), and 1,4-dioxane (4.0 mL). The reaction mixture was allowed to stir for 5 min at room temperature under an N_2 atmosphere, and then heated at 100 °C in a pre-heated oil bath for 48 h. The reaction mixture was then cooled to room temperature, diluted with 20 mL of CH_2Cl_2, filtered through a celite pad, and washed with 40-50 mL of CH_2Cl_2. The combined organic extracts were concentrated under reduced pressure and the resulting residue was purified by column chromatography on silica gel using petroleum ether/acetone/Et_3N (120/5/1, v/v/v) as the eluent to yield **3a** as a white solid (798 mg, 63% yield).

VII. Procedure for removal of the directing group[3]

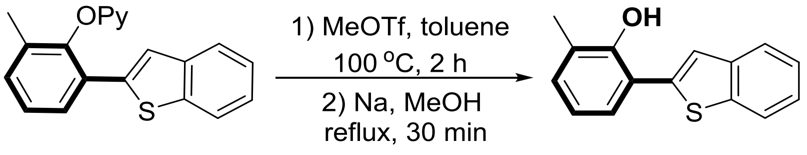

3a **5a**, 91%

To a solution of **3a** (63.4 mg, 0.2 mmol) in dry toluene (2 mL) was added MeOTf (67 μL, 0.6 mmol) under an N_2 atmosphere. The reaction mixture was stirred at 100 °C for 2 h. After cooling to room temperature, the solvent was evaporated under vacuum. Without further purification, the crude pyridinium was subsequently added to a mixture of Na (115 mg, 5.0 mmol) and dry methanol (4.0 mL) under N_2. The reaction mixture was heated at 80 °C for 30 min. Then it was allowed to cool and H_2O (6 mL) was added, and then 2M HCl aqueous solution was added to acidify the reaction system. The resulting mixture was extracted with EtOAc (3 × 10 mL). The combined organic layers were dried over anhydrous Na_2SO_4. After filtration, the solvent was evaporated under reduced pressure and the residue was purified by column chromatography on silica gel using petroleum ether/acetone (24/1, v/v) as the eluent to yield **5a** (43.7 mg) in 91% yield as a white solid. M.p.: 64-66 °C. ^1H NMR (400 MHz, $CDCl_3$): δ = 2.34 (s, 3H), 5.71 (s, 1H), 6.92 (t, J = 7.2 Hz, 1H), 7.18 (d, J = 6.8 Hz, 1H), 7.33 (d, J = 8.0 Hz, 1H), 7.37-7.41 (m, 2H), 7.50 (s, 1H), 7.82 (d, J = 7.2 Hz, 1H), 7.88 (d, J = 7.2 Hz, 1H) ppm. ^{13}C NMR (100 MHz, $CDCl_3$): δ = 16.4, 120.4, 120.5, 122.3, 122.8, 123.8, 124.7, 124.8, 125.1, 128.2, 131.5, 139.7, 140.2, 140.4, 151.2 ppm. HRMS (ESI$^-$): calcd for $C_{15}H_{11}OS$ $[M-H]^-$ 239.0536, found 239.0527.

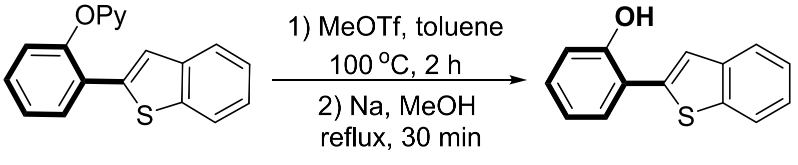

3n **5b**, 88%

To a solution of **3n** (60.6 mg, 0.2 mmol) in dry toluene (2 mL) was added MeOTf (67 μL, 0.6 mmol) under an N_2 atmosphere. The reaction mixture was stirred at 100 °C for 2 h. After cooling to room temperature, the solvent was evaporated under vacuum. Without further purification, the crude pyridinium was subsequently added to a mixture of Na (115 mg, 5.0 mmol) and dry methanol (4.0 mL) under N_2. The reaction mixture was heated at 80 °C for 30 min. Then it was allowed to cool and H_2O (6 mL) was added, and then 2 M HCl aqueous solution was added to acidify the reaction system. The resulting mixture was extracted with EtOAc (3 × 10 mL). The combined organic layers were dried over anhydrous Na_2SO_4. After filtration, the solvent was evaporated under reduced pressure and the residue was purified by column chromatography on silica gel using petroleum ether/acetone (24/1, v/v) as the eluent to yield **5b** (39.8 mg) in 88% yield as a white solid. M.p.: 85-86 °C. ^{1}H NMR (400 MHz, DMSO-d_6): δ = 6.91 (t, J = 7.6 Hz, 1H), 7.02 (d, J = 8.0 Hz, 1H), 7.21 (t, J = 7.6 Hz, 1H), 7.29-7.37 (m, 2H), 7.69 (d, J = 7.6 Hz, 1H), 7.84 (d, J = 7.6 Hz, 1H), 7.94 (d, J = 10.4 Hz, 2H), 10.39 (s, 1H) ppm. ^{13}C NMR (100 MHz, DMSO-d_6): δ = 116.5, 119.7, 120.3, 121.6, 121.9, 123.4, 124.1, 124.4, 128.6, 129.4, 138.8, 139.9, 140.2, 154.3 ppm. HRMS (ESI$^-$): calcd for $C_{14}H_9OS$ [M-H]$^-$ 225.0380, found 225.0376.

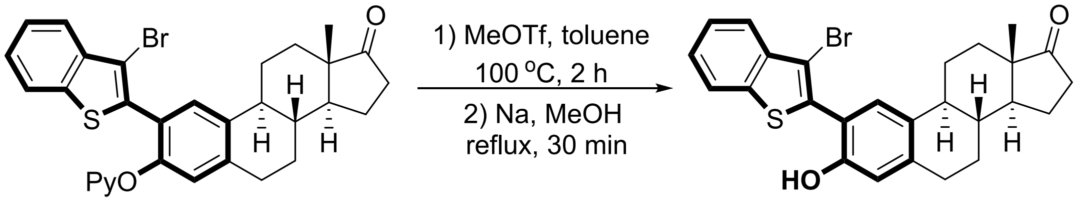

3t **6c**, 77%

To a solution of **3t** (111.6 mg, 0.2 mmol) in dry toluene (2 mL) was added MeOTf (67 μL, 0.6 mmol) under an N_2 atmosphere. The reaction mixture was stirred at 100 °C for 2 h. After cooling to room temperature, the solvent was evaporated under vacuum. Without further purification, the crude pyridinium was subsequently added to a mixture of Na (115 mg, 5.0 mmol) and dry methanol (4.0 mL) under N_2. The reaction mixture was heated at 80 °C for 30 min. Then it was allowed to cool and H_2O (6 mL) was added, and then 2M HCl aqueous solution was added to acidify the reaction system. The resulting mixture was extracted with EtOAc (3 × 10 mL). The combined organic layers were dried over anhydrous Na_2SO_4. After filtration, the solvent was evaporated under reduced pressure and the residue was purified by column chromatography on silica gel using petroleum ether/acetone (9/1, v/v) as the eluent to

yield **6c** (74.1 mg) in 77% yield as a white solid. M.p.: 165-166 °C. ^{1}H NMR (400 MHz, $CDCl_3$): δ = 0.92 (s, 3H), 1.42-1.69 (m, 6H), 1.94 (d, J = 11.6 Hz, 1H), 2.03-2.19 (m, 3H), 2.28-2.39 (m, 2H), 2.47-2.54 (m, 1H), 2.93-2.96 (m, 2H), 5.40 (s, 1H), 6.80 (s, 1H), 7.29 (s, 1H), 7.43 (t, J = 8.0 Hz, 1H), 7.50 (t, J = 7.2 Hz, 1H), 7.84 (d, J = 7.6 Hz, 1H), 7.86 (d, J = 8.0 Hz, 1H) ppm. ^{13}C NMR (100 MHz, $CDCl_3$): δ = 14.0, 21.7, 26.0, 26.5, 29.6, 31.6, 36.0, 38.3, 43.9, 48.1, 50.5, 108.2, 116.3, 116.6, 122.4, 123.7, 125.5, 125.9, 128.7, 132.4, 134.3, 138.5, 138.7, 140.3, 151.3, 221.4 ppm. HRMS (ESI$^-$): calcd for $C_{26}H_{24}BrO_2S$ [M-H]$^-$ 479.0686, 481.0665, found 479.0677, 481.0668.

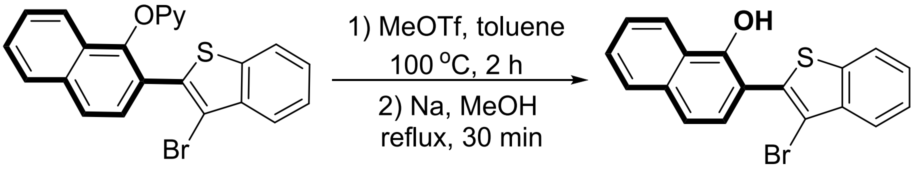

3v **6d**, 68%

To a solution of **3v** (86.4 mg, 0.2 mmol) in dry toluene (2 mL) was added MeOTf (67 μL, 0.6 mmol) under an N_2 atmosphere. The reaction mixture was stirred at 100 °C for 2 h. After cooling to room temperature, the solvent was evaporated under vacuum. Without further purification, the crude pyridinium was subsequently added to a mixture of Na (115 mg, 5.0 mmol) and dry methanol (4.0 mL) under N_2. The reaction mixture was heated at 80 °C for 30 min. Then it was allowed to cool and H_2O (6 mL) was added, and then 2M HCl aqueous solution was added to acidify the reaction system. The resulting mixture was extracted with EtOAc (3 × 10 mL). The combined organic layers were dried over anhydrous Na_2SO_4. After filtration, the solvent was evaporated under reduced pressure and the residue was purified by column chromatography on silica gel using petroleum ether/acetone (24/1, v/v) as the eluent to yield **6d** (48.3 mg) in 68% yield as a white solid. M.p.: 87-88 °C. ^{1}H NMR (400 MHz, DMSO-d_6): δ = 7.43 (d, J = 8.4 Hz, 1H), 7.48-7.52 (m, 2H), 7.54-7.61 (m, 3H), 7.83 (d, J = 7.6 Hz, 1H), 7.91 (d, J = 7.2 Hz, 1H), 8.07 (d, J = 8.0 Hz, 1H), 8.33 (d, J = 7.6 Hz, 1H), 9.87 (s, 1H) ppm. ^{13}C NMR (100 MHz, DMSO-d_6): δ = 107.8, 113.4, 119.2, 122.7, 122.82, 122.83, 125.2, 125.4, 125.6, 127.2, 127.4, 128.5, 134.7, 136.2, 137.9, 138.3, 151.0 ppm. HRMS (ESI$^-$): calcd for $C_{18}H_{10}BrOS$ [M-H]$^-$ 352.9641, 354.9621, found 352.9631, 354.9608.

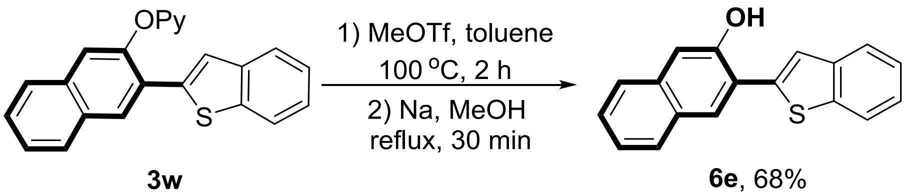

To a solution of **3w** (86.4 mg, 0.2 mmol) in dry toluene (2 mL) was added MeOTf (67 μL, 0.6 mmol) under an N_2 atmosphere. The reaction mixture was stirred at 100 °C for 2 h. After cooling to room temperature, the solvent was evaporated under vacuum. Without further purification, the crude pyridinium was subsequently added to a mixture of Na (115 mg, 5.0 mmol) and dry methanol (4.0 mL) under N_2. The reaction mixture was heated at 80 °C for 30 min. Then it was allowed to cool and H_2O (6 mL) was added, and then 2M HCl aqueous solution was added to acidify the reaction system. The resulting mixture was extracted with EtOAc (3 × 10 mL). The combined organic layers were dried over anhydrous Na_2SO_4. After filtration, the solvent was evaporated under reduced pressure and the residue was purified by column chromatography on silica gel using petroleum ether/acetone (9/1, v/v) as the eluent to yield **6e** (48.3 mg) in 68% yield as a white solid. M.p.: 49-50 °C. ^{1}H NMR (400 MHz, DMSO-d_6): δ = 7.31-7.35 (m, 2H), 7.45-7.52 (m, 2H), 7.54-7.58 (m, 1H), 7.76 (d, J = 8.4 Hz, 1H), 7.82 (d, J = 7.2 Hz, 1H), 7.86 (d, J = 8.0 Hz, 1H), 7.99 (s, 1H), 8.06 (d, J = 7.6 Hz, 1H), 10.34 (s, 1H) ppm. ^{13}C NMR (100 MHz, DMSO-d_6): δ = 107.2, 109.5, 122.2, 122.8, 122.9, 123.5, 125.5, 125.75, 125.81, 127.2, 127.3, 128.0, 131.9, 134.9, 135.7, 137.6, 138.0, 153.1 ppm. HRMS (ESI$^-$): calcd for $C_{18}H_{10}BrOS$ [M-H]$^-$ 352.9641, 354.9621, found 352.9637, 354.9612.

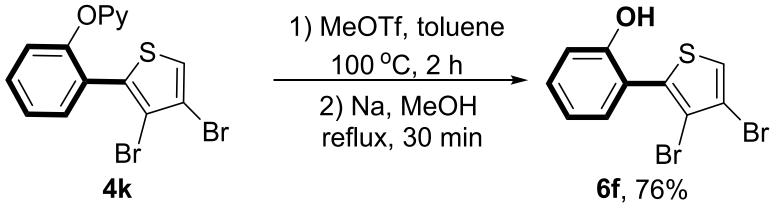

To a solution of **4k** (82 mg, 0.2 mmol) in dry toluene (2 mL) was added MeOTf (67 μL, 0.6 mmol) under an N_2 atmosphere. The reaction mixture was stirred at 100 °C for 2 h. After cooling to room temperature, the solvent was evaporated under vacuum. Without further purification, the crude pyridinium was subsequently added to a mixture of Na (115 mg, 5.0 mmol) and dry methanol (4.0 mL) under N_2. The reaction mixture was heated at 80 °C for 30 min. Then it was allowed to cool and H_2O (6 mL) was added, and then 2M HCl aqueous solution was added to acidify the reaction system. The resulting mixture was extracted with EtOAc (3 × 10 mL). The combined

organic layers were dried over anhydrous Na_2SO_4. After filtration, the solvent was evaporated under reduced pressure and the residue was purified by column chromatography on silica gel using petroleum ether/acetone (24/1, v/v) as the eluent to yield **6f** (50.6 mg) in 76% yield as yellow oil. ^{1}H NMR (400 MHz, DMSO-d_6): δ = 6.88 (t, J = 8.4 Hz, 1H), 6.95 (d, J = 8.0 Hz, 1H), 7.27 (t, J = 8.0 Hz, 2H), 7.95 (s, 1H), 9.96 (s, 1H) ppm. ^{13}C NMR (100 MHz, DMSO-d_6): δ = 112.4, 112.6, 116.0, 119.0, 119.1, 124.3, 130.7, 131.6, 136.6, 155.1 ppm. HRMS (ESI$^-$): calcd for $C_{10}H_5Br_2OS$ [M-H]$^-$ 330.8433, 332.8413, 334.8392, found 330.8430, 332.8407, 334.8386.

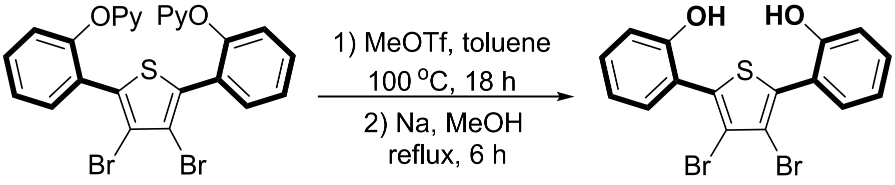

4q **6g**, 63%

To a solution of **4q** (115.8 mg, 0.2 mmol) in dry toluene (4 mL) was added MeOTf (201 μL, 1.8 mmol) under an N_2 atmosphere. The reaction mixture was stirred at 100 °C for 18 h. After cooling to room temperature, the solvent was evaporated under vacuum. Without further purification, the crude pyridinium was subsequently added to Na (345 mg, 15.0 mmol) and dry methanol (10.0 mL) under N_2. The reaction mixture was heated at 80 °C for 6 h. Then it was allowed to cool and H_2O (15 mL) was added, and then 2M HCl aqueous solution was added to acidify the reaction system. The resulting mixture was extracted with EtOAc (3 x 20 mL). The combined organic layers were dried over anhydrous Na_2SO_4. After filtration, the solvent was evaporated under reduced pressure and the residue was purified by column chromatography on silica gel using petroleum ether/ acetone (4/1, v/v) as the eluent to yield **6g** (53.6 mg) in 63% yield as a white solid. M.p.: 183-185 °C. ^{1}H NMR (400 MHz, DMSO-d_6): δ = 6.90 (t, J = 7.2 Hz, 2H), 6.97 (d, J = 8.0 Hz, 2H), 7.26-7.32 (m, 4H), 10.02 (s, 2H) ppm. ^{13}C NMR (100 MHz, DMSO-d_6): δ = 112.5, 116.0, 118.97, 119.03, 130.6, 131.6, 131.2, 155.2 ppm. HRMS (ESI$^-$): calcd for $C_{16}H_9Br_2O_2S$ [M-H]$^-$ 422.8695, 424.8675, 426.8655, found 422.8687, 424.8662, 426.8651.

VIII. Construction of benzofuran-fused heteroarenes[4]

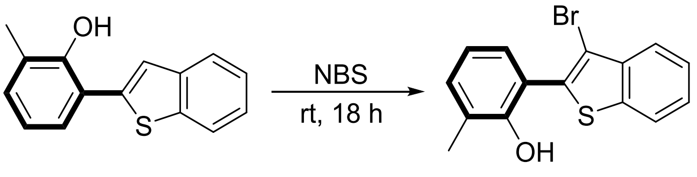

5a **6a**, 91%

N-Bromosuccinimide (39.2 mg, 0.22 mmol) was added portion-wise to a solution of **5a** (48.0 mg, 0.20 mmol) in dichloromethane (2 mL) at 0 ºC and the mixture was allowed to stir at room temperature for 18 h. After removal of the solvent under reduced pressure, the residue was purified via silica gel column chromatography (petroleum ether/ethyl acetate = 40/1, v/v) afforded the desired product as a white solid (57.9 mg, 91% yield). M.p.: 71-73 °C. ^{1}H NMR (400 MHz, DMSO-d_6): δ = 2.25 (s, 3H), 6.87 (t, J = 7.6 Hz, 1H), 7.16 (d, J = 7.6 Hz, 1H), 7.22 (d, J = 7.2 Hz, 1H), 7.48 (t, J = 7.2 Hz, 1H), 7.56 (t, J = 7.6 Hz, 1H), 7.79 (d, J = 8.0 Hz, 1H), 8.04 (d, J = 8.0 Hz, 1H), 8.83 (s, 1H) ppm. ^{13}C NMR (100 MHz, DMSO-d_6): δ = 16.8, 107.1, 119.3, 119.7, 122.7, 122.8, 125.4, 125.6, 125.7, 129.4, 132.0, 136.2, 137.8, 138.0, 153.1 ppm. HRMS (ESI$^-$): calcd for $C_{15}H_{10}BrOS$ [M-H]$^-$ 316.9641, 318.9621, found 316.9644, 318.9620.

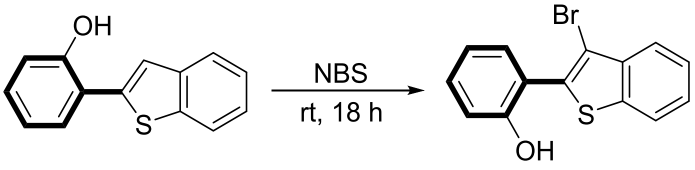

5b **6b**, 87%

N-Bromosuccinimide (35.6 mg, 0.20 mmol) was added portion-wise to a solution of **5b** (45.2 mg, 0.20 mmol) in dichloromethane (2 mL) at 0 ºC and the mixture was allowed to stir at room temperature for 18 h. After removal of the solvent under reduced pressure, the residue was purified via silica gel column chromatography (petroleum ether/ethyl acetate = 40/1, v/v) afforded the desired product as yellow oil (53.0 mg, 87% yield). ^{1}H NMR (400 MHz, DMSO-d_6): δ = 6.93 (t, J = 7.6 Hz, 1H), 7.01 (d, J = 7.6 Hz, 1H), 7.32 (t, J = 7.6 Hz, 1H), 7.36 (d, J = 7.6 Hz, 1H), 7.47 (t, J = 7.2 Hz, 1H), 7.53 (t, J = 7.2 Hz, 1H), 7.79 (d, J = 7.6 Hz, 1H), 8.02 (d, J = 8.0 Hz, 1H), 9.98 (s, 1H) ppm. ^{13}C NMR (100 MHz, DMSO-d_6): δ = 106.5, 116.1, 118.97, 118.99, 122.6, 122.7, 125.4, 125.5, 130.7, 131.8, 136.0, 137.6, 137.8, 155.3 ppm. HRMS (ESI$^-$): calcd for $C_{14}H_8BrOS$ [M-H]$^-$ 302.9485, 304.9464, found 302.9485, 304.9466.

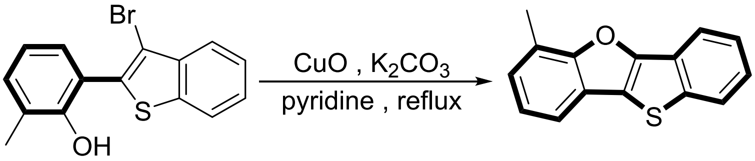

6a **7a**, 88%

A flame-dried Schlenk test tube with a magnetic stirring bar was charged with **6a**

(63.6 mg, 0.2 mmol), K_2CO_3 (138 mg, 1.0 mmol), and CuO (49.4 mg, 0.6 mmol) under an N_2 atmosphere. The tube was then evacuated and back-filled with N_2. The procedure of evacuation/backfill was sequentially repeated two additional times. Under a counter flow of argon, it was then added with pyridine (3 mL) by syringe. The resulting mixture was stirred for 3 min at room temperature, and then refluxed for 4 h. when the resulting solution was cooled to room temperature, the solvent was removed under reduced pressure. Purification via silica gel column chromatography (petroleum ether/dichloromethane = 4/1, v/v) afforded the desired product as a white solid (42.1 mg, 88% yield). M.p.: 112-114 °C. ^{1}H NMR (400 MHz, $CDCl_3$): δ = 2.64 (s, 3H), 7.17 (d, J = 7.2 Hz, 1H), 7.23 (t, J = 7.6 Hz, 1H), 7.37 (t, J = 7.6 Hz, 1H), 7.46 (t, J = 7.6 Hz, 1H), 7.54 (d, J = 7.6 Hz, 1H), 7.86 (d, J = 8.4 Hz, 1H), 8.02 (d, J = 8.0 Hz, 1H) ppm. ^{13}C NMR (100 MHz, $CDCl_3$): δ = 15.4, 117.2, 119.0, 119.8, 123.0, 123.4, 123.7, 124.5, 124.9, 125.0, 125.4, 126.2, 142.1, 152.9, 157.9 ppm. HRMS (ESI$^+$): calcd for $C_{15}H_{11}OS$ $[M+H]^+$ 239.0525, found 239.0519.

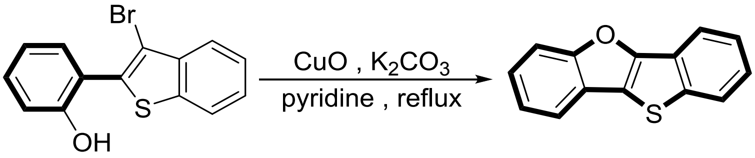

6b **7b**, 78%

A flame-dried Schlenk test tube with a magnetic stirring bar was charged with **6b** (63.6 mg, 0.2 mmol), K_2CO_3 (138 mg, 1.0 mmol), and CuO (49.4 mg, 0.6 mmol) under an N_2 atmosphere. The tube was then evacuated and back-filled with N_2. The procedure of evacuation/backfill was sequentially repeated two additional times. Under a counter flow of argon, it was then added with pyridine (3 mL) by syringe. The resulting mixture was stirred for 3 min at room temperature, and then refluxed for 4 h. when the resulting solution was cooled to room temperature, the solvent was removed under reduced pressure. Purification via silica gel column chromatography (petroleum ether/dichloromethane = 4/1, v/v) afforded the desired product as a white solid (35.0 mg, 78% yield). M.p.: 114-115 °C. ^{1}H NMR (400 MHz, DMSO-d_6): δ = 7.40 (t, J = 7.2 Hz, 1H), 7.44-7.49 (m, 2H), 7.55 (t, J = 7.2 Hz, 1H), 7.81 (d, J = 8.0 Hz, 1H), 7.95 (d, J = 6.8 Hz, 1H), 8.05 (d, J = 8.0 Hz, 1H), 8.13 (d, J = 8.4 Hz, 1H) ppm. ^{13}C NMR (100 MHz, DMSO-d_6): δ = 112.7, 118.2, 119.4, 120.1, 123.3, 123.8, 124.2, 124.9, 125.41, 125.44, 125.5, 141.5, 152.2, 158.2 ppm. HRMS (ESI$^+$): calcd for $C_{14}H_8NaOS$ $[M+Na]^+$ 247.0188, found 247.0192.

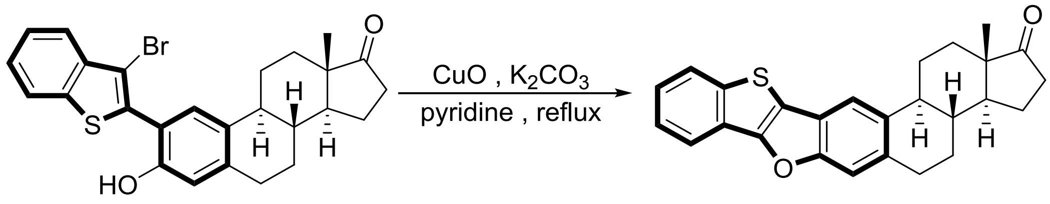

A flame-dried Schlenk test tube with a magnetic stirring bar was charged with **6c** (96.2 mg, 0.2 mmol), K_2CO_3 (138 mg, 1.0 mmol), and CuO (49.4 mg, 0.6 mmol) under an N_2 atmosphere. The tube was then evacuated and back-filled with N_2. The procedure of evacuation/backfill was sequentially repeated two additional times. Under a counter flow of argon, it was then added with pyridine (3 mL) by syringe. The resulting mixture was stirred for 3 min at room temperature, and then refluxed for 4 h. when the resulting solution was cooled to room temperature, the solvent was removed under reduced pressure. Purification via silica gel column chromatography (petroleum ether/acetone = 8/1, v/v) afforded the desired product as a white solid (48.8 mg, 61% yield). M.p.: 206-207 °C. ^{1}H NMR (400 MHz, $CDCl_3$): δ = 0.94 (s, 3H), 1.48-1.75 (m, 6H), 2.01-2.21 (m, 4H), 2.39-2.44 (m, 1H), 2.50-2.58 (m, 2H), 3.07-3.10 (m, 2H), 7.35-7.39 (m, 2H), 7.46 (t, J = 7.6 Hz, 1H), 7.64 (s, 1H), 7.87 (d, J = 7.6 Hz, 1H), 7.97 (d, J = 8.0 Hz, 1H) ppm. ^{13}C NMR (100 MHz, $CDCl_3$): δ = 14.0, 21.8, 26.4, 26.7, 30.2, 31.7, 36.0, 38.3, 44.5, 48.1, 50.7, 112.2, 116.0, 118.8, 119.6, 122.1, 124.5, 124.7, 125.0, 125.4, 134.5, 135.7, 141.9, 152.8, 157.6, 221.1 ppm. HRMS (ESI$^+$): calcd for $C_{26}H_{24}NaO_2S$ $[M+Na]^+$ 423.1389, found 423.1387.

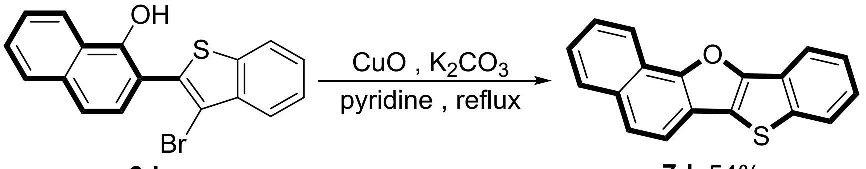

A flame-dried Schlenk test tube with a magnetic stirring bar was charged with **6d** (71 mg, 0.2 mmol), K_2CO_3 (138 mg, 1.0 mmol), and CuO (49.4 mg, 0.6 mmol) under an N_2 atmosphere. The tube was then evacuated and back-filled with N_2. The procedure of evacuation/backfill was sequentially repeated two additional times. Under a counter flow of argon, it was then added with pyridine (3 mL) by syringe. The resulting mixture was stirred for 3 min at room temperature, and then refluxed for 4 h. when the resulting solution was cooled to room temperature, the solvent was removed under reduced pressure. Purification via silica gel column chromatography (petroleum ether/dichloromethane = 4/1, v/v) afforded the desired product as a white solid (29.6 mg, 54% yield). M.p.: 237-238 °C. ^{1}H NMR (400 MHz, $CDCl_3$): δ = 7.43 (t, J = 7.6

Hz, 1H), 7.47-7.53 (m, 3H), 7.92 (d, J = 8.4 Hz, 1H), 7.99 (t, J = 9.2 Hz, 2H), 8.03 (s, 1H), 8.05 (d, J = 7.6 Hz, 1H), 8.17 (s, 1H) ppm. ^{13}C NMR (100 MHz, $CDCl_3$): δ = 108.4, 117.6, 118.1, 120.2, 124.6, 124.8, 125.20, 125.21, 125.47, 125.54, 128.1, 128.2, 130.6, 131.5, 142.7, 155.1, 158.0 ppm. HRMS (ESI$^+$): calcd for $C_{18}H_{10}NaOS$ $[M+Na]^+$ 297.0345, found 297.0343.

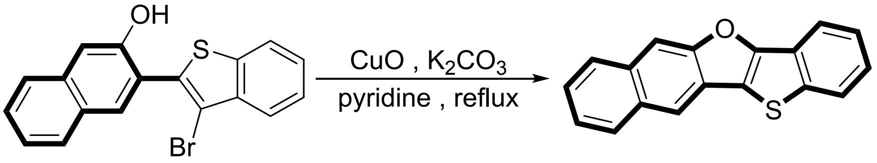

6e **7e**, 56%

A flame-dried Schlenk test tube with a magnetic stirring bar was charged with **6e** (71 mg, 0.2 mmol), K_2CO_3 (138 mg, 1.0 mmol), and CuO (49.4 mg, 0.6 mmol) under an N_2 atmosphere. The tube was then evacuated and back-filled with N_2. The procedure of evacuation/backfill was sequentially repeated two additional times. Under a counter flow of argon, it was then added with pyridine (3 mL) by syringe. The resulting mixture was stirred for 3 min at room temperature, and then refluxed for 4 h. when the resulting solution was cooled to room temperature, the solvent was removed under reduced pressure. Purification via silica gel column chromatography (petroleum ether/dichloromethane = 4/1, v/v) afforded the desired product as a white solid (30.7 mg, 56% yield). M.p.: 190-192 °C. ^{1}H NMR (400 MHz, $CDCl_3$): δ = 7.40 (t, J = 7.6 Hz, 1H), 7.50-7.56 (m, 2H), 7.66 (t, J = 7.6 Hz, 1H), 7.78 (dd, J = 11.6 Hz, 8.8Hz, 2H), 7.91 (d, J = 8.0 Hz, 1H), 7.98 (d, J = 8.0 Hz, 1H), 8.11 (d, J = 8.0 Hz, 1H), 8.47 (d, J = 8.4 Hz, 1H) ppm. ^{13}C NMR (100 MHz, $CDCl_3$): δ = 118.1, 119.6, 119.7, 120.0, 120.3, 122.2, 124.1, 124.5, 124.7, 125.1, 125.4, 125.6, 126.8, 128.6, 131.7, 141.8, 152.6, 154.2 ppm. HRMS (ESI$^+$): calcd for $C_{18}H_{11}OS$ $[M+H]^+$ 275.0525, found 275.0525.

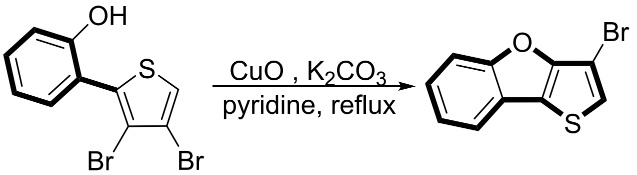

6f **7f**, 92%

A flame-dried Schlenk test tube with a magnetic stirring bar was charged with **6f** (66.8 mg, 0.2 mmol), K_2CO_3 (138 mg, 1.0 mmol), and CuO (49.4 mg, 0.6 mmol) under an N_2 atmosphere. The tube was then evacuated and back-filled with N_2. The procedure of evacuation/backfill was sequentially repeated additional two times. Under a counter flow of argon, it was then added with pyridine (3 mL) by syringe.

The resulting mixture was stirred for 3 min at room temperature, and then refluxed for 4 h. When the resulting solution was cooled to room temperature, the solvent was removed under reduced pressure. Purification via silica gel column chromatography (petroleum ether/dichloromethane = 4/1, v/v) afforded the desired product as yellow oil (46.8 mg, 92% yield). ^{1}H NMR (400 MHz, DMSO-d_6): δ = 7.38 (t, J = 7.2 Hz, 1H), 7.44 (t, J = 6.8 Hz, 1H), 7.78 (d, J = 8.0 Hz, 1H), 7.92 (s, 1H), 7.94 (d, J = 7.2 Hz, 1H) ppm. ^{13}C NMR (100 MHz, DMSO-d_6): δ = 93.6, 112.8, 119.0, 120.1, 123.4, 123.9, 125.6, 126.6, 155.3, 158.3 ppm. HRMS (ESI$^+$): calcd for $C_{10}H_6BrOS$ $[M+H]^+$ 252.9317, 254.9297, found 252.9311, 254.9295.

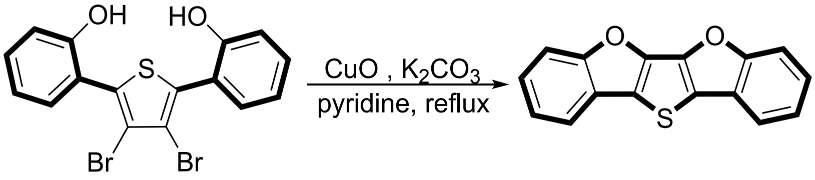

6g **7g**, 71%

A flame-dried Schlenk test tube with a magnetic stirring bar was charged with **6g** (85.2 mg, 0.2 mmol), K_2CO_3 (276 mg, 2.0 mmol), and CuO (98.8 mg, 1.2 mmol) under an N_2 atmosphere. The tube was then evacuated and back-filled with N_2. The procedure of evacuation/backfill was sequentially repeated additional two times. Under a counter flow of argon, it was then added with pyridine (5 mL) by syringe. The resulting mixture was stirred for 3 min at room temperature, and then refluxed for 12 h. When the resulting solution was cooled to room temperature, the solvent was removed under reduced pressure. Purification via silica gel column chromatography (petroleum ether/dichloromethane = 4/1, v/v) afforded the desired product as a white solid (37.8 mg, 71% yield). M.p.: 193-195 °C. ^{1}H NMR (400 MHz, DMSO-d_6): δ = 7.41-7.48 (m, 4H), 7.82 (d, J = 7.6 Hz, 2H), 8.01 (d, J = 7.2 Hz, 2H) ppm. ^{13}C NMR (100 MHz, DMSO-d_6): δ = 112.9, 119.7, 122.6, 123.9, 124.2, 125.6, 141.2, 158.1 ppm. HRMS (ESI$^+$): calcd for $C_{16}H_8NaO_2S$ $[M+Na]^+$ 287.0137, found 287.0138.

IX. Mechanistic study

1. H/D exchange experiments

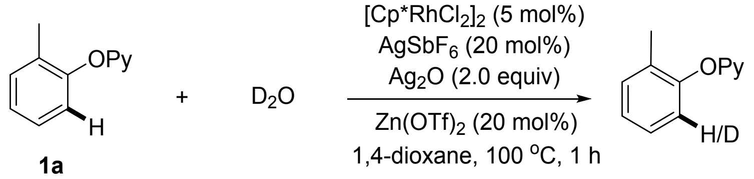

Eq. S1

1a 0.2 mmol — 20 equiv — 42% [D]-**1a**

A flame-dried Schlenk test tube with a magnetic stirring bar was charged with **1a** (37.0 mg, 0.20 mmol), D_2O (73 μL, 20.0 equiv), $[Cp^*RhCl_2]_2$ (5.0 mol%), $AgSbF_6$ (20 mol%), Ag_2O (2.0 equiv), $Zn(OTf)_2$ (30 mol%), and 1,4-dioxane (0.5 mL). The reaction mixture was allowed to stir for 5 min at room temperature under an N_2 atmosphere, and then heated at 100 °C in a pre-heated oil bath for 1 h. The reaction mixture was then cooled to room temperature, diluted with 10 mL of CH_2Cl_2, filtered through a celite pad, and washed with 25-35 mL of CH_2Cl_2. The combined organic extracts were concentrated under reduced pressure and the resulting residue was purified by column chromatography on silica gel (petroleum ether/acetone/Et_3N = 120/5/1, v/v/v) to provide the desired product. The deuterated ratio was calculated from ^1H NMR analysis. The ^1H NMR analysis showed that 42% hydrogen at the *ortho*-position of the phenyl ring of **1a** was deuterated.

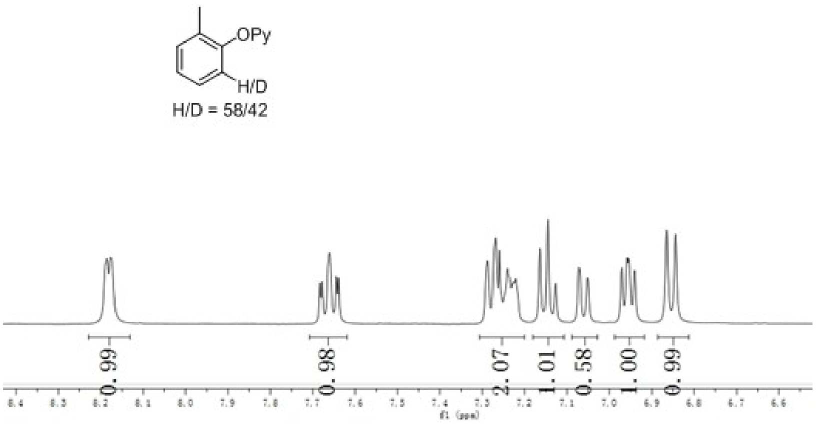

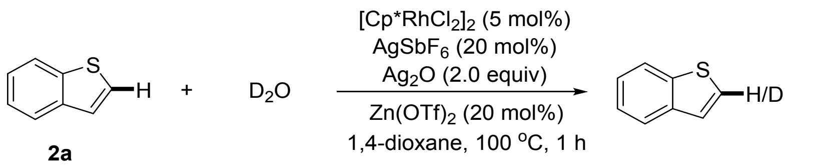

Eq. S2

2a 0.2 mmol | 20 equiv | 13% [D]-**2a**

A flame-dried Schlenk test tube with a magnetic stirring bar was charged with **2a** (26.8 mg, 0.20 mmol), D_2O (73 μL, 20.0 equiv), $[Cp^*RhCl_2]_2$ (5.0 mol%), $AgSbF_6$ (20 mol%), Ag_2O (2.0 equiv), $Zn(OTf)_2$ (30 mol%), and 1,4-dioxane (0.5 mL). The reaction mixture was allowed to stir for 5 min at room temperature under an N_2 atmosphere, and then heated at 100 °C in a pre-heated oil bath for 1 h. The reaction mixture was then cooled to room temperature, diluted with 10 mL of CH_2Cl_2, filtered through a celite pad, and washed with 25-35 mL of CH_2Cl_2. The combined organic extracts were concentrated under reduced pressure and the resulting residue was

purified by column chromatography on silica gel (petroleum ether) to provide the desired product. The deuterated ratio was calculated from ^{1}H NMR analysis. The ^{1}H NMR analysis showed that 13% hydrogen at the 2-position of **2a** was deuterated.

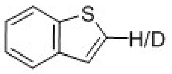

H/D = 87/13

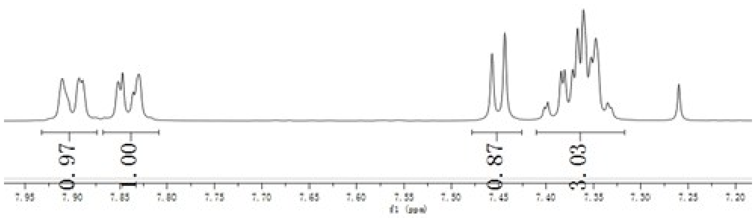

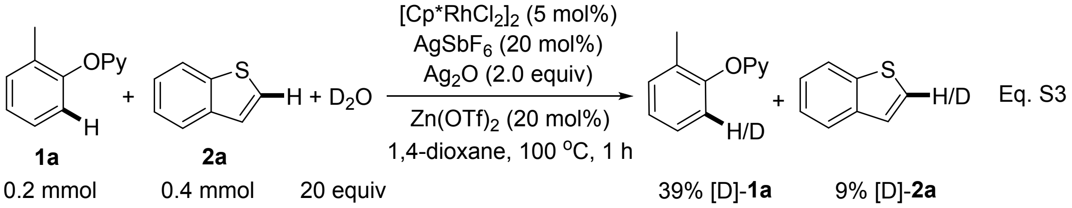

A flame-dried Schlenk test tube with a magnetic stirring bar was charged with **1a** (37.0 mg, 0.20 mmol), **2a** (2.0 equiv), D_2O (73 μL, 20.0 equiv), $[Cp^*RhCl_2]_2$ (5.0 mol%), $AgSbF_6$ (20 mol%), Ag_2O (2.0 equiv), $Zn(OTf)_2$ (30 mol%), and 1,4-dioxane (0.5 mL). The reaction mixture was allowed to stir for 5 min at room temperature under an N_2 atmosphere, and then heated at 100 °C in a pre-heated oil bath for 1 h. The reaction mixture was then cooled to room temperature, diluted with 10 mL of CH_2Cl_2, filtered through a celite pad, and washed with 25-35 mL of CH_2Cl_2. The combined organic extracts were concentrated under reduced pressure and the resulting residue was purified by column chromatography on silica gel (petroleum ether/acetone/Et_3N = 120/5/1, v/v/v) to provide [D]-**1a**, [D]-**2a** and **3a** (7.6 mg, 12% yield). The deuterated ratio was calculated from ^{1}H NMR analysis. The ^{1}H NMR analysis showed that 39% hydrogen at the *ortho*-position of the phenyl ring of **1a** and 9% hydrogen at the 2-position of **2a** were deuterated.

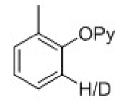

H/D = 61/39

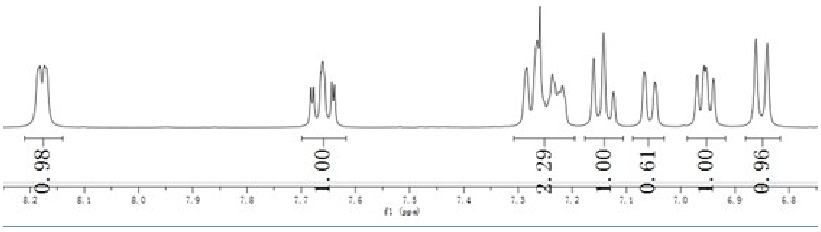

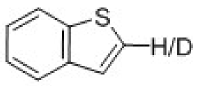

H/D = 91/9

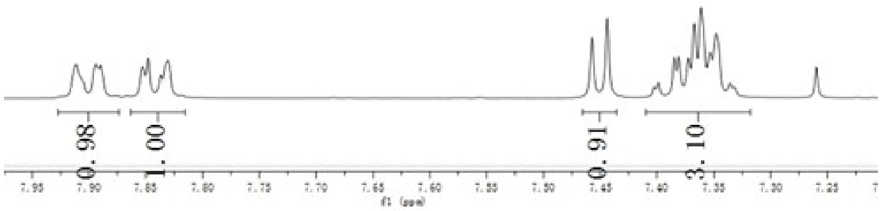

2. Kinetic isotope experiments

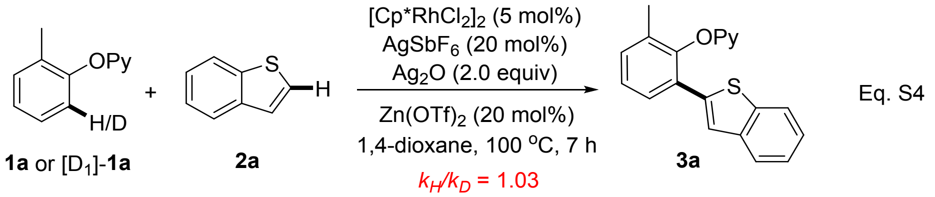

A flame-dried Schlenk test tube with a magnetic stirring bar was charged with **1a** (37.0 mg, 0.20 mmol) or [D_1]-**1a**(37.2 mg, 0.20 mmol), **2a** (2.0 equiv), [Cp*$RhCl_2$]$_2$ (5.0 mol%), $AgSbF_6$ (20 mol%), Ag_2O (2.0 equiv), Zn(OTf)$_2$ (30 mol%), and 1,4-dioxane (0.5 mL). The reaction mixture was allowed to stir for 5 min at room temperature under an N_2 atmosphere, and then heated at 100 °C in a pre-heated oil bath for 7 h. The reaction mixture was then cooled to room temperature, diluted with 10 mL of CH_2Cl_2, filtered through a celite pad, and washed with 25-35 mL of CH_2Cl_2. The combined organic extracts were concentrated under reduced pressure and the resulting residue was purified by column chromatography on silica gel (petroleum ether/acetone/Et_3N = 120/5/1, v/v/v) to provide the desired product.

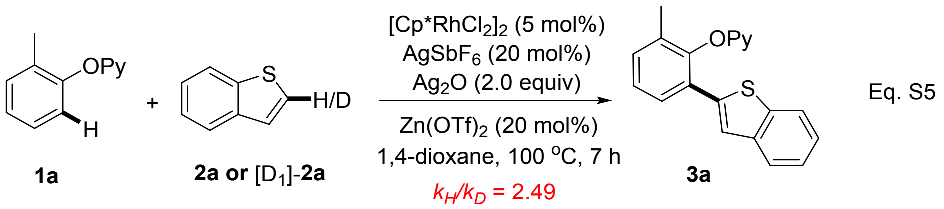

A flame-dried Schlenk test tube with a magnetic stirring bar was charged with **1a** (37.0 mg, 0.20 mmol), **2a** (2.0 equiv) or [D_1]-**2a** (2.0 equiv), $[Cp^*RhCl_2]_2$ (5.0 mol%), $AgSbF_6$ (20 mol%), Ag_2O (2.0 equiv), $Zn(OTf)_2$ (30 mol%), and 1,4-dioxane (0.5 mL). The reaction mixture was allowed to stir for 5 min at room temperature under an N_2 atmosphere, and then heated at 100 °C in a pre-heated oil bath for 7 h. The reaction mixture was then cooled to room temperature, diluted with 10 mL of CH_2Cl_2, filtered through a celite pad, and washed with 25-35 mL of CH_2Cl_2. The combined organic extracts were concentrated under reduced pressure and the resulting residue was purified by column chromatography on silica gel (petroleum ether/acetone/Et_3N = 120/5/1, v/v/v) to provide the desired product.

3. Possible reaction mechanism

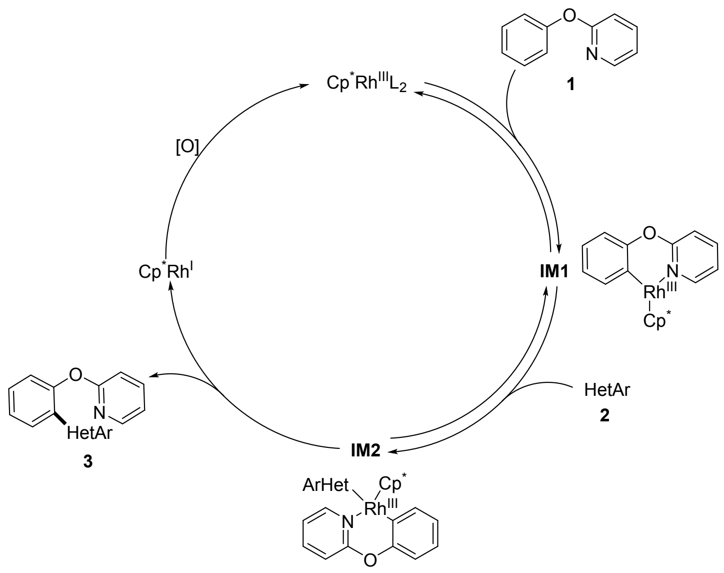

Scheme S2. Plausible mechanistic pathway

X. Single-crystal X-ray structure of 3n

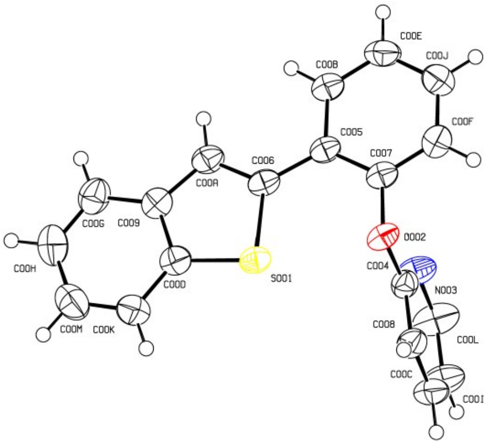

Figure S1. ORTEP diagram of **3n**. Thermal ellipsoids are shown at the 50% probability level. CCDC 1837385 (**3n**) contains the supplementary crystallographic data for this paper. These data can be obtained free of charge from The Cambridge Crystallographic Data Centre via www.ccdc.cam.ac.uk/data request/cif.

XI. Experimental data for the described substances

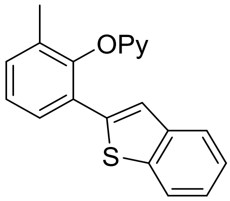

2-(2-(Benzo[*b*]thiophen-2-yl)-6-methylphenoxy)pyridine (3a)

Purification via silica gel column chromatography (petroleum ether/acetone/Et_3N = 120/5/1, v/v/v) afforded the desired product **3a** as a white solid (52.1 mg, 82% yield). M.p.: 137-139 °C. ^{1}H NMR (400 MHz, $CDCl_3$): δ = 2.16 (s, 3H), 6.86-6.89 (m, 2H), 7.22-7.29 (m, 4H), 7.54 (s, 1H), 7.58-7.65 (m, 2H), 7.69 (d, J =7.6 Hz, 1H), 7.74 (d, J = 7.6 Hz, 1H), 8.09 (d, J = 4.4 Hz, 1H) ppm. ^{13}C NMR (100 MHz, $CDCl_3$): δ = 7.1, 110.4, 118.1, 122.0, 122.9, 123.6, 124.2, 126.0, 128.0, 128.4, 131.3, 132.8, 139.5, 139.7, 140.0, 140.3, 148.0, 148.8, 163.0 ppm. HRMS (ESI$^+$): calcd for $C_{20}H_{15}NNaOS$ $[M+Na]^+$ 340.0767, found 340.0775.

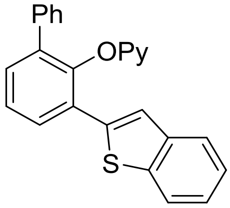

2-((3-(Benzo[*b*]thiophen-2-yl)-[1,1'-biphenyl]-2-yl)methyl)pyridine (3b)

Purification via silica gel column chromatography (petroleum ether/acetone/Et_3N = 120/5/1, v/v/v) afforded the desired product **3b** as a white solid (64.2 mg, 84% yield). M.p.: 104-106 °C. ^1H NMR (400 MHz, $CDCl_3$): δ = 6.63-6.67 (m, 2H), 7.16-7.19 (m, 1H), 7.22-7.29 (m, 4H), 7.34-7.44 (m, 5H), 7.59 (s, 1H), 7.69 (d, J = 7.2 Hz, 1H), 7.74 (d, J = 7.6 Hz, 1H), 7.79 (dd, J = 6.8 Hz, 2.4 Hz, 1H), 7.90 (dd, J = 4.8 Hz, 1.6 Hz, 1H) ppm. ^{13}C NMR (100 MHz, $CDCl_3$): δ = 110.9, 117.8, 122.0, 123.2, 123.7, 124.2, 124.3, 126.2, 127.3, 128.0, 129.1, 129.3, 129.7, 131.3, 137.0, 138.0, 138.9, 139.5, 140.0, 140.4, 147.4, 147.7, 162.9 ppm. HRMS (ESI$^+$): calcd for $C_{25}H_{18}NOS$ $[M+H]^+$ 380.1104, found 380.1108.

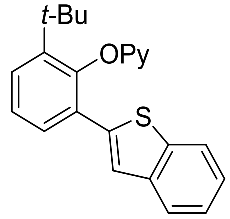

2-(2-(Benzo[*b*]thiophen-2-yl)-6-(*tert*-butyl)phenoxy)pyridine (3c)

Purification via silica gel column chromatography (petroleum ether/acetone/Et_3N = 120/5/1, v/v/v) afforded the desired product **3c** as a white solid (58.2 mg, 81% yield). M.p.: 97-98 °C. ^1H NMR (400 MHz, DMSO-d_6): δ = 1.30 (s, 9H), 6.80 (t, J = 6.0 Hz, 1H), 6.92 (d, J = 8.0 Hz, 1H), 7.22-7.29 (m, 2H), 7.32 (t, J = 7.6 Hz, 1H), 7.50-7.52 (m, 2H), 7.57 (d, J = 7.6 Hz, 1H), 7.61 (t, J = 6.8 Hz, 1H), 7.70 (d, J = 7.2 Hz, 1H), 7.81 (d, J = 8.4 Hz, 1H), 7.87 (d, J = 3.2 Hz, 1H) ppm. ^{13}C NMR (100 MHz, DMSO-d_6): δ = 30.6, 34.8, 110.9, 118.1, 122.0, 123.3, 123.5, 124.3, 125.6, 127.9, 129.1, 129.3, 139.1, 139.35, 139.47, 139.50, 142.9, 146.9, 148.9, 162.7 ppm. HRMS (ESI$^+$): calcd for $C_{23}H_{22}NOS$ $[M+H]^+$ 360.1417, found 360.1417.

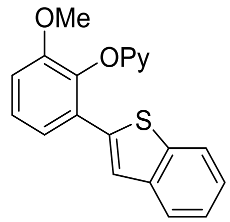

2-(2-(Benzo[*b*]thiophen-2-yl)-6-methoxyphenoxy)pyridine (3d)

Purification via silica gel column chromatography (petroleum ether/acetone/Et_3N = 90/10/1, v/v/v) afforded the desired product **3d** as a white solid (42.6 mg, 64% yield).

M.p.: 154-156 °C. ^{1}H NMR (400 MHz, DMSO-d_6): δ = 3.68 (s, 3H), 7.02 (t, J = 6.0 Hz,1H), 7.12 (d, J = 8.4 Hz, 1H), 7.16 (d, J = 8.0 Hz, 1H), 7.28-7.35 (m, 3H), 7.50 (d, J = 8.0 Hz, 1H), 7.79-7.83 (m, 3H), 7.88 (d, J = 7.6 Hz, 1H), 8.02 (d, J = 4.8 Hz, 1H) ppm. ^{13}C NMR (100 MHz, DMSO-d_6): δ = 56.0, 110.6, 112.7, 118.6, 120.5, 122.0, 122.8, 123.7, 124.6, 124.7, 126.2, 128.1, 138.3, 138.8, 139.2, 139.5, 139.9, 147.1, 152.6, 162.4 ppm. HRMS (ESI$^+$): calcd for $C_{20}H_{16}NO_2S$ $[M+H]^+$ 334.0896, found 334.0893.

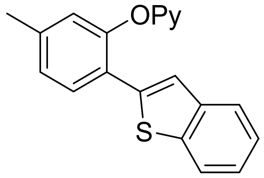

2-(2-(Benzo[*b*]thiophen-2-yl)-5-methylphenoxy)pyridine (3e)

Purification via silica gel column chromatography (petroleum ether/acetone/Et_3N = 120/5/1, v/v/v) afforded the desired product **3e** as a white solid (45.6 mg, 72% yield). M.p.: 136-138 °C. ^{1}H NMR (400 MHz, $CDCl_3$): δ = 2.37 (s, 3H), 6.90-6.93 (m, 2H), 7.00 (s, 1H), 7.09 (d, J = 7.6 Hz, 1H), 7.23-7.29 (m, 2H), 7.58 (d, J = 2.0 Hz, 1H), 7.60-7.64 (m, 1H), 7.69 (d, J = 8.0 Hz, 2H), 7.74 (d, J = 7.6 Hz, 1H), 8.16 (d, J = 4.4 Hz, 1H) ppm. ^{13}C NMR (100 MHz, $CDCl_3$): δ = 21.3, 111.5, 118.5, 122.0, 122.3, 123.5, 123.7, 124.1, 124.2, 124.7, 126.6, 129.9, 139.4, 139.5, 139.8, 140.0, 140.1, 147.9, 150.5, 163.5 ppm. HRMS (ESI$^+$): calcd for $C_{20}H_{15}NNaOS$ $[M+Na]^+$ 340.0767, found 340.0767.

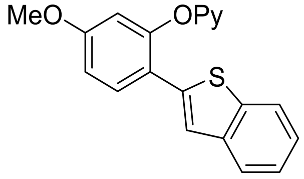

2-(2-(Benzo[*b*]thiophen-2-yl)-5-methoxyphenoxy)pyridine (3f)

Purification via silica gel column chromatography (petroleum ether/acetone/Et_3N = 90/10/1, v/v/v) afforded the desired product **3f** as a white solid (43.3 mg, 65% yield). M.p.: 145-147 °C. ^{1}H NMR (400 MHz, $CDCl_3$): δ = 3.83 (s,3H), 6.75 (d, J = 2.4 Hz, 1H), 6.87 (d, J = 6.4 Hz, 1H), 6.94-6.97 (m, 2H), 7.22-7.30 (m, 2H), 7.53 (s, 1H), 7.64-7.70 (m, 2H), 7.72 (d, J = 8.8 Hz, 1H), 7.75 (d, J = 7.6 Hz, 1H), 8.18 (d, J = 3.6

Hz, 1H) ppm. ^{13}C NMR (100 MHz, $CDCl_3$): δ = 55.7, 108.7, 111.6, 111.8, 118.8, 120.3, 121.7, 122.0, 123.4, 124.0, 124.2, 130.9, 139.4, 139.6, 139.8, 140.2, 148.0, 151.7, 160.5, 163.4 ppm. HRMS (ESI$^+$): calcd for $C_{20}H_{16}NO_2S$ $[M+H]^+$ 334.0896, found 334.0895.

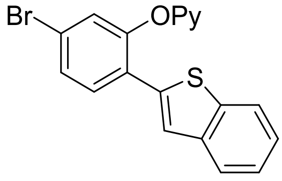

2-(2-(Benzo[*b*]thiophen-2-yl)-5-bromophenoxy)pyridine (3g)

Purification via silica gel column chromatography (petroleum ether/acetone/Et_3N = 120/4/1, v/v/v) afforded the desired product **3g** as a white solid (49.6 mg, 65% yield). M.p.: 162-163 °C. ^{1}H NMR (400 MHz, $CDCl_3$): δ = 6.99 (t, J = 3.6 Hz, 1H), 7.01 (s, 1H), 7.27-7.33 (m, 2H), 7.37 (d, J = 2.0 Hz, 1H), 7.42 (d, J = 8.4 Hz, 1H), 7.62 (s, 1H), 7.67 (d, J = 8.4 Hz, 1H), 7.72 (t, J = 7.2 Hz, 2H), 7.77 (d, J = 6.8 Hz, 1H), 8.17 (d, J = 3.6 Hz, 1H) ppm. ^{13}C NMR (100 MHz, $CDCl_3$): δ = 111.9, 119.2, 122.1, 123.1, 123.8, 124.5, 124.6, 126.4, 126.7, 128.7, 131.1, 138.2, 139.8, 139.9, 140.2, 148.0, 151.2, 162.9 ppm. HRMS (ESI$^+$): calcd for $C_{19}H_{13}BrNOS$ $[M+H]^+$ 381.9896, 383.9875, found 381.9889, 383.9869.

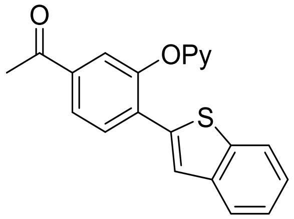

1-(4-(Benzo[*b*]thiophen-2-yl)-3-(pyridin-2-yloxy)phenyl)ethan-1-one (3h)

Purification via silica gel column chromatography (petroleum ether/acetone/Et_3N = 90/10/1, v/v/v) afforded the desired product **3h** as a white solid (43.5 mg, 63% yield). M.p.: 178-180 °C. ^{1}H NMR (400 MHz, DMSO-d_6): δ = 2.60 (s, 3H), 7.13 (t, J = 6.0 Hz,1H), 7.25 (d, J = 8.4 Hz, 1H), 7.33-7.40 (m, 2H), 7.74 (s, 1H), 7.87 (d, J = 8.0 Hz, 1H), 7.90 (d, J = 7.2 Hz, 1H), 7.94 (d, J = 7.6 Hz, 2H), 8.04 (s, 1H), 8.09 (d, J = 3.6 Hz, 1H), 8.12 (d, J = 8.4 Hz, 1H) ppm. ^{13}C NMR (100 MHz, DMSO-d_6): δ = 26.9, 111.9, 119.5, 122.2, 123.2, 124.1, 124.3, 124.8, 125.2, 125.4, 129.5, 131.1, 137.0, 137.3, 139.1, 139.9, 140.5, 147.4, 150.0, 162.5, 196.8 ppm. HRMS (ESI$^+$): calcd for

$C_{21}H_{16}NO_2S$ [M+H]$^+$ 346.0896, found 346.0891.

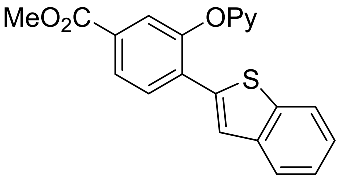

Methyl 4-(benzo[*b*]thiophen-2-yl)-3-(pyridin-2-yloxy)benzoate (3i)

Purification via silica gel column chromatography (petroleum ether/acetone/Et_3N = 90/10/1, v/v/v) afforded the desired product **3i** as a white solid (43.3 mg, 60% yield). M.p.: 165-166 °C. ^1H NMR (400 MHz, DMSO-d_6): δ = 3.86 (s, 3H), 7.15 (dd, J = 7.6 Hz, 4.8 Hz, 1H), 7.26 (d, J = 8.0 Hz, 1H), 7.33-7.40 (m, 2H), 7.70 (d, J = 2.0 Hz, 1H), 7.86-7.88 (m, 1H), 7.90-7.95 (m, 3H), 8.04 (s, 1H), 8.10-8.11 (m, 1H), 8.13 (d, J = 8.0 Hz, 1H) ppm. ^{13}C NMR (100 MHz, DMSO-d_6): δ = 52.5, 112.1, 119.6, 122.2, 124.1, 124.29, 124.34, 124.8, 125.3, 126.0, 129.6, 130.1, 131.2, 136.9, 139.1, 139.9, 140.6, 147.5, 149.9, 162.3, 165.2 ppm. HRMS (ESI$^+$): calcd for $C_{21}H_{16}NO_3S$ [M+H]$^+$ 362.0845, found 362.0840.

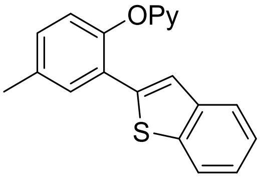

2-(2-(Benzo[*b*]thiophen-2-yl)-4-methylphenoxy)pyridine (3j)

Purification via silica gel column chromatography (petroleum ether/acetone/Et_3N = 120/5/1, v/v/v) afforded the desired product **3j** as a white solid (33.6 mg, 53% yield). M.p.: 99-101 °C. ^1H NMR (400 MHz, $CDCl_3$): δ = 2.43 (s, 3H), 6.92-6.95 (m, 2H), 7.10 (d, J = 8.0 Hz, 1H), 7.20 (dd, J = 8.0 Hz, 2.0 Hz, 1H), 7.24-7.32 (m, 2H), 7.62-7.67 (m, 3H), 7.72 (d, J = 6.8 Hz, 1H), 7.77 (d, J = 7.6 Hz, 1H), 8.16 (dd, J = 4.8 Hz, 1.2 Hz, 1H) ppm. ^{13}C NMR (100 MHz, $CDCl_3$): δ = 21.2, 111.4, 118.5, 122.0, 122.8, 123.3, 123.7, 124.26, 124.27, 127.2, 130.1, 130.6, 135.3, 139.4, 139.5, 140.1, 140.2, 147.9, 148.4, 163.7 ppm. HRMS (ESI$^+$): calcd for $C_{20}H_{15}NNaOS$ [M+Na]$^+$ 340.0767, found 340.0771.

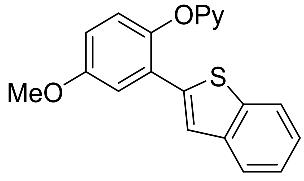

2-(2-(Benzo[*b*]thiophen-2-yl)-4-methoxyphenoxy)pyridine (3k)

Purification via silica gel column chromatography (petroleum ether/acetone/Et_3N = 90/10/1, v/v/v) afforded the desired product **3k** as a white solid (31.3 mg, 47% yield). M.p.: 62-64 °C. ^{1}H NMR (400 MHz, DMSO-d_6): δ = 3.87 (s, 3H), 7.01-7.07 (m, 2H), 7.10 (d, J = 8.4 Hz, 1H), 7.16 (d, J = 9.2 Hz, 1H), 7.29-7.37 (m, 2H), 7.43 (d, J = 2.8 Hz, 1H), 7.80-7.85 (m, 2H), 7.89-7.90 (m, 2H), 8.06 (d, J = 3.6 Hz, 1H) ppm. ^{13}C NMR (100 MHz, DMSO-d_6): δ = 55.7, 111.3, 113.3, 115.5, 118.8, 122.1, 122.8, 123.7, 124.6, 124.7, 125.1, 127.5, 138.2, 139.2, 139.6, 140.1, 143.5, 147.3, 156.5, 163.1 ppm. HRMS (ESI$^+$): calcd for $C_{20}H_{16}NO_2S$ [M+H]$^+$ 334.0896, found 334.0893.

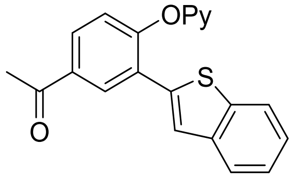

1-(3-(Benzo[*b*]thiophen-2-yl)-4-(pyridin-2-yloxy)phenyl)ethan-1-one (3l)

Purification via silica gel column chromatography (petroleum ether/acetone/Et_3N = 90/10/1, v/v/v) afforded the desired product **3l** as a white solid (43.3 mg, 49% yield). M.p.: 107-109 °C. ^{1}H NMR (400 MHz, DMSO-d_6): δ = 2.69 (s, 3H), 7.16 (t, J = 6.0 Hz, 1H), 7.25 (d, J = 8.4 Hz, 1H), 7.32-7.39 (m, 3H), 7.87 (d, J = 7.2 Hz, 1H), 7.89-7.94 (m, 2H), 7.99-8.01 (m, 2H), 8.12 (d, J = 3.6 Hz, 1H), 8.47 (d, J = 2.4 Hz, 1H) ppm. ^{13}C NMR (100 MHz, DMSO-d_6): δ = 26.9, 112.2, 119.8, 122.2, 123.5, 123.6, 123.9, 124.7, 124.9, 126.7, 129.4, 129.6, 134.0, 137.3, 139.2, 139.6, 140.6, 147.5, 153.9, 162.2, 196.8 ppm. HRMS (ESI$^+$): calcd for $C_{21}H_{16}NO_2S$ [M+H]$^+$ 346.0896, found 346.0892.

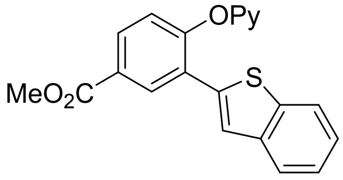

Methyl 3-(benzo[*b*]thiophen-2-yl)-4-(pyridin-2-yloxy)benzoate (3m)

Purification via silica gel column chromatography (petroleum ether/acetone/Et_3N = 90/10/1, v/v/v) afforded the desired product **3m** as a white solid (31.0 mg, 43% yield). M.p.: 99-100 °C. ^{1}H NMR (400 MHz, $CDCl_3$): δ = 3.96 (s, 3H), 7.01-7.06 (m, 2H), 7.25 (d, J = 8.4 Hz, 1H), 7.27-7.35 (m, 2H), 7.70-7.80 (m, 4H), 8.04 (d, J = 6.4 Hz, 1H), 8.18 (d, J = 2.0 Hz, 1H), 8.53 (d, J = 2.4 Hz, 1H) ppm. ^{13}C NMR (100 MHz, $CDCl_3$): δ = 52.4, 112.3, 119.4, 122.1, 122.8, 123.5, 123.9, 124.4, 124.6, 127.1, 127.4, 130.4, 131.8, 138.2, 139.8, 139.9, 140.4, 148.0, 154.5, 162.8, 166.4 ppm. HRMS (ESI$^+$): calcd for $C_{21}H_{16}NO_3S$ [M+H]$^+$ 362.0845, found 362.0843.

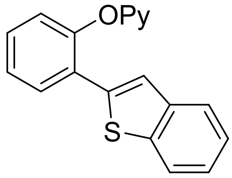

2-(2-(Benzo[*b*]thiophen-2-yl)phenoxy)pyridine (3n)

Purification via silica gel column chromatography (petroleum ether/acetone/Et_3N = 120/5/1, v/v/v) afforded the desired product **3n** as a white solid (40.1 mg, 66% yield). M.p.: 133-135 °C. ^{1}H NMR (400 MHz, $CDCl_3$): δ = 6.96 (d, J = 8.4 Hz, 2H), 7.21 (d, J = 8.0 Hz, 1H), 7.27-7.33 (m, 3H), 7.40 (t, J = 7.6 Hz, 1H), 7.65-7.69 (m, 2H), 7.73 (d, J = 7.6 Hz, 1H), 7.78 (d, J = 7.6 Hz, 1H), 7.83 (d, J = 8.0 Hz, 1H), 8.17 (d, J = 4.4 Hz, 1H) ppm. ^{13}C NMR (100 MHz, $CDCl_3$): δ = 111.6, 118.7, 122.0, 122.9, 123.4, 123.7, 124.3, 124.4, 125.6, 127.6, 129.4, 130.2, 139.2, 139.6, 140.1, 140.2, 148.0, 150.7, 163.5 ppm. HRMS (ESI$^+$): calcd for $C_{19}H_{14}NOS$ [M+H]$^+$ 304.0791, found 304.0795.

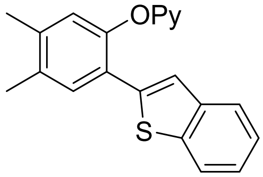

2-(2-(Benzo[*b*]thiophen-2-yl)-4,5-dimethylphenoxy)pyridine (3o)

Purification via silica gel column chromatography (petroleum ether/acetone/Et_3N = 120/5/1, v/v/v) afforded the desired product **3o** as a white solid (48.1 mg, 73% yield). M.p.: 120-122 °C. ^{1}H NMR (400 MHz, $CDCl_3$): δ = 2.28 (s, 3H), 2.32 (s, 3H), 6.90-

6.93 (m, 2H), 6.98 (s, 1H), 7.22-7.31 (m, 2H), 7.57 (s, 2H), 7.61-7.65 (m, 1H), 7.69 (d, *J* = 7.2 Hz, 1H), 7.75 (d, *J* = 8.0 Hz, 1H), 8.15 (dd, *J* = 4.8 Hz, 1.6 Hz, 1H) ppm. ^{13}C NMR (100 MHz, $CDCl_3$): δ = 19.5, 19.9, 111.4, 118.4, 122.0, 122.2, 123.5, 124.1, 124.2, 124.4, 124.8, 131.0, 134.1, 138.5, 139.4, 139.5, 140.0, 140.2, 148.0, 148.4, 163.8 ppm. HRMS (ESI$^+$): calcd for $C_{21}H_{18}NOS$ $[M+H]^+$ 332.1104, found 332.1101.

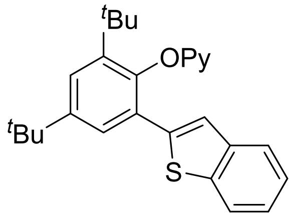

2-(2-(Benzo[*b*]thiophen-2-yl)-4,6-di-*tert*-butylphenoxy)pyridine (3p)

Purification via silica gel column chromatography (petroleum ether/acetone/Et_3N = 120/4/1, v/v/v) afforded the desired product **3p** as a white solid (67.2 mg, 81% yield). M.p.: 164-166 °C. ^{1}H NMR (400 MHz, $CDCl_3$): δ = 1.41 (s, 9H), 1.42 (s, 9H), 6.65 (dd, *J* = 6.0 Hz, 4.8 Hz, 1H), 6.66 (d, *J* = 8.4 Hz, 1H), 7.19-7.27 (m, 2H), 7.36-7.41 (m, 2H), 7.51 (d, *J* = 2.4 Hz, 1H), 7.55 (d, *J* = 2.4 Hz, 1H), 7.63 (d, *J* = 7.2 Hz, 1H), 7.70 (d, *J* = 7.6 Hz, 1H), 7.96 (dd, *J* = 4.2 Hz, 1.6 Hz, 1H) ppm. ^{13}C NMR (100 MHz, $CDCl_3$): δ = 30.9, 31.6, 34.9, 35.5, 110.6, 117.5, 121.9, 123.4, 123.6, 123.8, 124.0, 125.3, 127.0, 128.6, 138.8, 140.0, 141.0, 142.4, 147.2, 147.6, 163.4 ppm. HRMS (ESI$^+$): calcd for $C_{27}H_{29}NNaOS$ $[M+Na]^+$ 438.1862, found 438.1870.

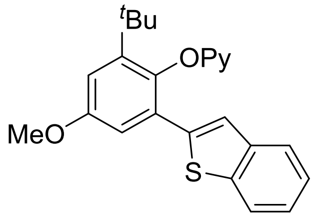

2-(2-(Benzo[*b*]thiophen-2-yl)-6-(*tert*-butyl)-4-methoxyphenoxy)pyridine (3q)

Purification via silica gel column chromatography (petroleum ether/acetone/Et_3N = 150/10/1, v/v/v) afforded the desired product **3q** as a white solid (68.1 mg, 87% yield). M.p.: 151-153 °C. ^{1}H NMR (400 MHz, $CDCl_3$): δ = 1.36 (s, 9H), 3.86 (s, 3H), 6.64 (dd, *J* = 6.8 Hz, 5.2 Hz, 1H), 6.69 (d, *J* = 8.4 Hz, 1H), 7.02 (d, *J* = 2.8 Hz, 1H), 7.06 (d, *J* = 3.2 Hz, 1H), 7.18-7.25 (m, 2H), 7.37-7.41 (m, 2H), 7.62 (d, *J* = 7.2 Hz, 1H), 7.68 (d, *J* = 7.2 Hz, 1H), 7.96 (dd, *J* = 4.8 Hz, 1.2 Hz, 1H) ppm. ^{13}C NMR (100 MHz,

$CDCl_3$): δ = 30.8, 35.5, 55.6, 110.7, 113.2, 114.9, 117.5, 121.9, 123.5, 123.8, 124.0, 124.1, 130.2, 138.8, 139.9, 140.1, 140.4, 143.2, 144.8, 147.6, 156.4, 163.6 ppm. HRMS (ESI$^+$): calcd for $C_{24}H_{23}NNaO_2S$ $[M+Na]^+$ 412.1342, found 412.1352.

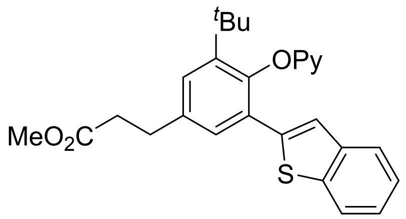

Methyl 3-(3-(benzo[*b*]thiophen-2-yl)-5-(*tert*-butyl)-4-(pyridin-2-yloxy)phenyl)pro-panoate (3r)

Purification via silica gel column chromatography (petroleum ether/acetone/Et_3N = 150/10/1, v/v/v) afforded the desired product **3r** as a white solid (76.8 mg, 86% yield). M.p.: 86-88 °C. ^1H NMR (400 MHz, $CDCl_3$): δ = 1.38 (s, 9H), 2.73 (t, *J* = 8.0 Hz, 2H), 3.03 (t, *J* = 8.0 Hz, 2H), 3.72 (s, 3H), 6.65 (t, *J* = 6.0 Hz, 1H), 6.72 (d, *J* = 7.6 Hz, 1H), 7.18-7.26 (m, 2H), 7.34-7.42 (m, 4H), 7.62 (d, *J* = 7.2 Hz, 1H), 7.69 (d, *J* = 7.6 Hz, 1H), 7.95 (dd, *J* = 4.8 Hz, 1.6 Hz, 1H) ppm. ^{13}C NMR (100 MHz, $CDCl_3$): δ = 30.83, 30.84, 35.3, 35.7, 51.9, 110.8, 117.6, 121.9, 123.4, 123.7, 123.9, 124.0, 128.1, 129.4, 129.6, 137.4, 138.8, 139.9, 140.0, 140.3, 143.4, 147.6, 147.9, 163.3, 173.5 ppm. HRMS (ESI$^+$): calcd for $C_{27}H_{28}NO_3S$ $[M+H]^+$ 446.1784, found 446.1789.

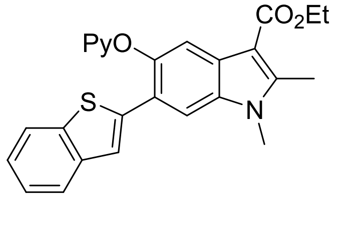

Ethyl 6-(benzo[*b*]thiophen-2-yl)-1,2-dimethyl-5-(pyridin-2-yloxy)-1*H*-indole- -3-carboxylate (3s)

Purification via silica gel column chromatography (petroleum ether/dichloromethane/ethyl acetate/Et_3N = 60/30/10/1, v/v/v/v) afforded the desired product **3s** as a white solid (49.5 mg, 56% yield). M.p.: 245-247 °C. ^1H NMR (400 MHz, $CDCl_3$): δ = 1.36 (t, *J* = 8.0 Hz, 3H), 2.78 (s, 3H), 3.73 (s, 3H), 4.34 (dd, *J* = 14.4 Hz, 7.2 Hz, 2H), 6.87-6.90 (m, 2H), 7.22-7.30 (m, 2H), 7.58 (d, *J* = 7.2 Hz, 1H), 7.62 (d, *J* = 6.8 Hz, 2H), 7.69 (d, *J* = 7.2 Hz, 1H), 7.75 (d, *J* = 8.0 Hz, 1H), 7.91 (s, 1H), 8.14 (d, *J* = 3.6 Hz, 1H) ppm. ^{13}C NMR (100 MHz, $CDCl_3$): δ = 12.2, 14.7, 30.0,

59.6, 104.5, 110.3, 111.0, 115.5, 118.1, 122.0, 122.4, 122.5, 123.5, 124.0, 124.2, 127.5, 134.5, 139.3, 140.0, 140.3, 140.5, 146.3, 147.4, 148.0, 164.4, 165.8 ppm. HRMS (ESI$^+$): calcd for $C_{26}H_{23}N_2O_3S$ $[M+H]^+$ 443.1424, found 443.1429.

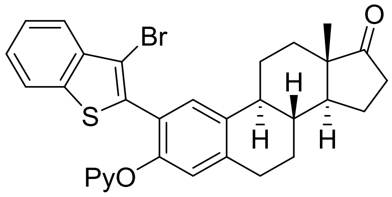

(*8R,9S,13S,14S*)-2-(3-Bromobenzo[*b*]thiophen-2-yl)-13-methyl-3-(pyridin-2-yloxy)-6,7,8,9,11,12,13,14,15,16-decahydro-17*H*-cyclopenta[*a*]phenanthren-17-one (3t)

Purification via silica gel column chromatography (petroleum ether/acetone/Et_3N = 150/10/1, v/v/v) afforded the desired product **3t** as a white solid (54.7 mg, 49% yield). M.p.: 115-116 °C. ^{1}H NMR (400 MHz, $CDCl_3$): δ = 0.94 (s, 3H), 1.64-1.67 (m, 6H), 1.97 (d, J = 12.0 Hz, 1H), 2.05-2.21 (m, 3H), 2.37-2.56 (m, 3H), 2.97-3.00 (m, 2H), 6.81 (d, J = 8.0 Hz, 1H), 6.85 (t, J = 6.0 Hz, 1H), 7.01 (s, 1H), 7.33 (t, J = 6.8 Hz, 1H), 7.40 (t, J = 7.2 Hz, 1H), 7.49-7.54 (m, 2H), 7.70 (d, J = 8.0 Hz, 1H), 7.77 (d, J = 8.0 Hz, 1H), 8.12 (d, J = 3.2 Hz, 1H) ppm. ^{13}C NMR (100 MHz, $CDCl_3$): δ = 14.0, 21.7, 25.8, 26.5, 29.6, 31.6, 36.0, 38.0, 44.3, 48.1, 50.5, 107.6, 111.5, 118.3, 122.1, 122.6, 123.3, 123.5, 124.9, 125.3, 130.0, 135.0, 136.7, 138.2, 138.6, 139.2, 139.7, 147.5, 149.6, 163.5, 221.0 ppm. HRMS (ESI$^+$): calcd for $C_{31}H_{29}BrNO_2S$ $[M+H]^+$ 558.1097, 560.1076, found 558.1099, 560.1074.

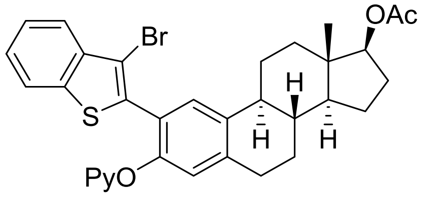

(8*R*,9*S*,13*S*,14*S*,17*S*)-2-(3-Bromobenzo[*b*]thiophen-2-yl)-13-methyl-3-(pyridin-2-yloxy)-7,8,9,11,12,13,14,15,16,17-decahydro-6*H*-cyclopenta[*a*]phenanthren-17-yl acetate (3u)

Purification via silica gel column chromatography (petroleum ether/acetone/Et_3N = 200/10/1, v/v/v) afforded the desired product **3u** as a white solid (91.5 mg, 76% yield). M.p.: 88-90 °C. ^{1}H NMR (400 MHz, $CDCl_3$): δ = 0.86 (s, 3H), 1.28-1.63 (m, 6H), 1.70-1.80 (m, 2H), 1.88-1.95 (m, 2H), 2.06 (s, 3H), 2.19-2.36 (m, 3H), 2.93 (t, J = 4.4 Hz, 2H), 4.70 (t, J = 8.4 Hz, 1H), 6.80 (d, J = 8.0 Hz, 1H), 6.84 (t, J = 5.6 Hz, 1H),

6.99 (s, 1H), 7.32 (t, J = 6.8 Hz, 1H), 7.40 (t, J = 6.8 Hz, 1H), 7.48-7.52 (m, 2H), 7.69 (d, J = 8.0 Hz, 1H), 7.77 (d, J = 8.0 Hz, 1H), 8.12 (d, J = 3.6 Hz, 1H) ppm. ^{13}C NMR (100 MHz, $CDCl_3$): δ = 12.2, 21.3, 23.4, 26.1, 27.1, 27.7, 29.7, 36.9, 38.2, 43.0, 44.1, 49.9, 82.8, 107.6, 111.5, 118.3, 122.1, 122.5, 123.1, 123.4, 124.9, 125.2, 129.9, 135.2, 137.1, 138.2, 138.6, 139.2, 139.9, 147.5, 149.4, 163.5, 171.4 ppm. HRMS (ESI$^+$): calcd for $C_{33}H_{33}BrNO_3S$ [M+H]$^+$ 602.1359, 604.1339, found 602.1355, 604.1337.

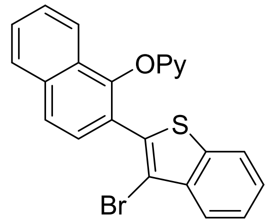

2-((2-(3-Bromobenzo[*b*]thiophen-2-yl)naphthalen-1-yl)oxy)pyridine (3v)

Purification via silica gel column chromatography (petroleum ether/acetone/Et_3N = 120/5/1, v/v/v) afforded the desired product **3v** as a white solid (53.6 mg, 62% yield). M.p.: 173-174 °C. ^{1}H NMR (400 MHz, DMSO-d_6):δ = 6.98 (t, J = 6.0 Hz, 1H), 7.03 (d, J = 8.4 Hz, 1H), 7.44 (t, J = 7.6 Hz, 1H), 7.51 (t, J = 7.6 Hz, 1H), 7.56 (t, J = 7.6 Hz, 1H), 7.65 (t, J = 7.6 Hz, 1H), 7.69-7.74 (m, 2H), 7.77 (d, J = 7.6 Hz, 1H), 7.83 (d, J = 8.4 Hz, 1H), 7.93 (d, J = 4.8 Hz, 1H), 7.97 (d, J = 7.6 Hz, 1H), 8.01 (d, J = 8.4 Hz, 1H), 8.10 (d, J = 8.0 Hz, 1H) ppm. ^{13}C NMR (100 MHz, DMSO-d_6): δ = 107.7, 110.3, 118.8, 122.4, 122.5, 122.7, 122.9, 125.4, 125.5, 125.9, 127.2, 127.52, 127.54, 128.2, 128.5, 134.5, 134.8, 137.2, 137.9, 140.2, 147.1, 147.3, 163.3 ppm. HRMS (ESI$^+$): calcd for $C_{23}H_{15}BrNOS$ [M+H]$^+$ 432.0052, 434.0032, found 432.0055, 434.0034.

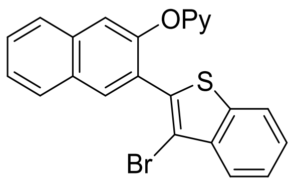

2-((3-(3-Bromobenzo[*b*]thiophen-2-yl)naphthalen-2-yl)oxy)pyridine (3w)

Purification via silica gel column chromatography (petroleum ether/acetone/Et_3N = 120/5/1, v/v/v) afforded the desired product **3w** as a white solid (47.5 mg, 55% yield). M.p.: 116-117 °C. ^{1}H NMR (400 MHz, DMSO-d_6): δ = 6.93 (d, J = 8.4 Hz, 1H), 7.00 (t, J = 6.0 Hz, 1H), 7.44 (t, J = 7.6 Hz, 1H), 7.51 (t, J = 7.6 Hz, 1H), 7.57 (t, J = 7.2 Hz, 1H), 7.61 (t, J = 7.6 Hz, 1H), 7.68 (t, J = 6.8 Hz, 1H), 7.76 (d, J = 7.6 Hz, 1H),

7.83 (s, 1H), 7.96 (d, J = 4.0 Hz, 1H), 7.98 (d, J = 4.0 Hz, 1H), 8.03-8.07 (m, 2H), 8.22 (s, 1H) ppm. ^{13}C NMR (100 MHz, DMSO-d_6): δ = 107.8, 111.1, 119.0, 119.3, 122.7, 123.0, 125.55, 125.58, 125.9, 126.1, 127.2, 127.6, 128.1, 130.2, 132.5, 134.1, 134.4, 137.3, 137.9, 140.0, 147.2, 149.2, 163.0 ppm. HRMS (ESI$^+$): calcd for $C_{23}H_{15}BrNOS$ [M+H]$^+$ 432.0052, 434.0032, found 432.0051, 434.0028.

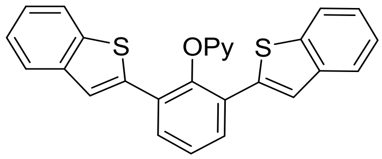

2-(2,6-Bis(benzo[*b*]thiophen-2-yl)phenoxy)pyridine (3x)

Purification via silica gel column chromatography (petroleum ether/acetone/Et_3N = 120/5/1, v/v/v) afforded the desired product **3x** as a white solid (62.6 mg, 72% yield). M.p.: 191-192 °C. ^{1}H NMR (400 MHz, DMSO-d_6): δ = 6.86 (t, J = 6.0 Hz, 1H), 7.16 (d, J = 8.0 Hz, 1H), 7.29-7.36 (m, 4H), 7.53 (t, J = 7.6 Hz, 1H), 7.70 (t, J = 7.6 Hz, 1H), 7.80 (d, J = 5.2 Hz, 4H), 7.85 (d, J = 4.8 Hz, 1H), 7.90 (t, J = 8.4 Hz, 4H) ppm. ^{13}C NMR (100 MHz, DMSO-d_6): δ = 111.5, 118.7, 122.1, 123.5, 123.8, 124.6, 124.7, 126.6, 129.2, 130.1, 138.1, 139.2, 139.5, 139.8, 146.5, 146.9, 161.9 ppm. HRMS (ESI$^+$): calcd for $C_{27}H_{18}NOS_2$ [M+H]$^+$ 436.0824, found 436.0825.

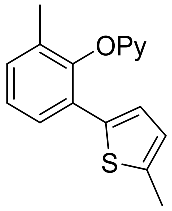

2-(2-Methyl-6-(5-methylthiophen-2-yl)phenoxy)pyridine (4a)

Purification via silica gel column chromatography (petroleum ether/acetone/Et_3N = 100/2/1, v/v/v) afforded the desired product **4a** as a white solid (48.1 mg, 85% yield). M.p.: 82-83 °C. ^{1}H NMR (400 MHz, $CDCl_3$): δ = 2.13 (s, 3H), 2.41 (s, 3H), 6.62 (s, 1H), 6.85 (d, J = 8.0 Hz, 1H), 6.90 (t, J = 6.4 Hz, 1H), 9.13 (d, J = 2.8 Hz, 1H), 7.18 (d, J = 4.8 Hz, 2H), 7.53 (t, J = 4.8 Hz, 1H), 7.63 (t, J = 8.0 Hz, 1H), 8.12 (d, J = 4.4 Hz, 1H) ppm. ^{13}C NMR (100 MHz, $CDCl_3$): δ = 15.3, 17.1, 110.3, 117.9, 125.4, 125.8, 126.1, 126.9, 128.6, 130.0, 132.5, 136.7, 139.4, 140.4, 147.9, 148.0, 163.0 ppm. HRMS (ESI$^+$): calcd for $C_{17}H_{15}NNaOS$ [M+Na]$^+$ 304.0767, found 304.0777.

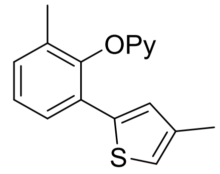

2-(2-Methyl-6-(4-methylthiophen-2-yl)phenoxy)pyridine (4b)

Purification via silica gel column chromatography (petroleum ether/acetone/Et_3N = 100/2/1, v/v/v) afforded the desired product **4b** as a white solid (32.8 mg, 59% yield). M.p.: 104-106 °C. ^1H NMR (400 MHz, $CDCl_3$): δ = 2.13 (s, 3H), 2.19 (s, 3H), 6.80 (s, 1H), 6.86 (d, J = 8.4 Hz, 1H), 6.90 (t, J = 6.4 Hz, 1H), 7.14 (s, 1H), 7.19-7.20 (m, 2H), 7.55 (t, J = 4.8 Hz, 1H), 7.61-7.65 (m, 1H), 8.11 (d, J = 4.0 Hz, 1H) ppm. ^{13}C NMR (100 MHz, $CDCl_3$): δ = 15.9, 17.1, 110.4, 118.0, 121.5, 125.8, 127.0, 128.4, 130.3, 132.6, 137.5, 138.7, 139.4, 148.0, 148.1, 162.9 ppm. HRMS (ESI$^+$): calcd for $C_{17}H_{15}NNaOS$ $[M+Na]^+$ 304.0767, found 304.0773.

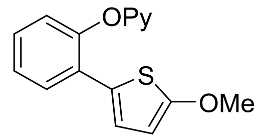

2-(2-(5-Methoxythiophen-2-yl)phenoxy)pyridine (4c)

Purification via silica gel column chromatography (petroleum ether/acetone/Et_3N = 150/10/1, v/v/v) afforded the desired product **4c** as a white solid (29.4 mg, 52% yield). M.p.: 60-61 °C. ^1H NMR (400 MHz, $CDCl_3$): δ = 3.85 (s, 3H), 6.12 (d, J = 4.0 Hz, 1H), 6.92 (d, J = 8.0 Hz, 1H), 6.96 (t, J = 6.0 Hz, 1H), 7.06 (d, J = 4.0 Hz, 1H), 7.13 (d, J = 7.6 Hz, 1H), 7.2-7.28 (m, 2H), 7.64-7.68 (m, 2H), 8.17 (d, J = 4.8 Hz, 1H) ppm. ^{13}C NMR (100 MHz, $CDCl_3$): δ = 60.1, 104.0, 111.5, 118.5, 123.3, 123.7, 124.6, 125.5, 127.5, 127.8, 128.3, 139.5, 147.9, 149.5, 163.5, 167.1 ppm. HRMS (ESI$^+$): calcd for $C_{16}H_{14}NO_2S$ $[M+H]^+$ 284.0740, found 284.0738.

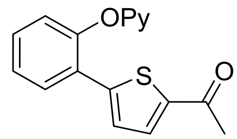

1-(5-(2-(Pyridin-2-yloxy)phenyl)thiophen-2-yl)ethan-1-one (4d)

Purification via silica gel column chromatography (petroleum ether/acetone/Et_3N =

90/10/1, v/v/v) afforded the desired product **4d** as a white solid (41.3 mg, 70% yield). M.p.: 113-114 °C. ^{1}H NMR (400 MHz, DMSO-d_6): δ = 2.49 (s, 3H), 7.11-7.16 (m, 2H), 7.21 (d, J = 8.0 Hz, 1H), 7.35 (t, J = 7.6 Hz, 1H), 7.45 (t, J = 6.8 Hz, 1H), 7.66 (d, J = 4.0 Hz, 1H), 7.86-7.91 (m, 2H), 7.95 (d, J = 6.8 Hz, 1H), 8.09 (d, J = 3.6 Hz, 1H) ppm. ^{13}C NMR (100 MHz, DMSO-d_6): δ = 26.5, 111.7, 119.4, 123.7, 125.6, 125.9, 127.2, 128.9, 130.2, 133.9, 140.4, 143.6, 145.7, 147.5, 150.0, 162.4, 191.0 ppm. HRMS (ESI$^+$): calcd for $C_{17}H_{14}NO_2S$ [M+H]$^+$ 296.0740, found 296.0744.

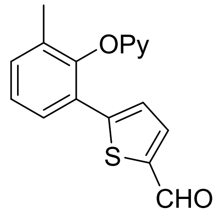

5-(3-Methyl-2-(pyridin-2-yloxy)phenyl)thiophene-2-carbaldehyde (4e)

Purification via silica gel column chromatography (petroleum ether/acetone/Et_3N = 90/10/1, v/v/v) afforded the desired product **4e** as a white solid (39.3 mg, 66% yield). M.p.: 114-116 °C. ^{1}H NMR (400 MHz, $CDCl_3$): δ = 2.14 (s, 3H), 6.92 (t, J = 6.0 Hz, 1H), 6.96 (d, J = 8.4 Hz, 1H), 7.23-7.27 (m, 1H), 7.32 (t, J = 7.2 Hz, 1H), 7.42 (d, J = 4.0 Hz, 1H), 7.59-7.63 (m, 2H), 7.65-7.69 (m, 1H), 8.06 (d, J = 4.0 Hz, 1H), 9.81 (s, 1H) ppm. ^{13}C NMR (100 MHz, $CDCl_3$): δ = 17.1, 110.9, 118.4, 126.1, 127.0, 127.2, 127.4, 132.4, 133.1, 136.5, 139.8, 143.1, 147.8, 148.8, 149.4, 162.5, 183.2 ppm. HRMS (ESI$^+$): calcd for $C_{17}H_{13}NNaO_2S$ [M+Na]$^+$ 318.0559, found 318.0568.

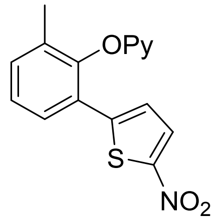

2-(2-Methyl-6-(5-nitrothiophen-2-yl)phenoxy)pyridine (4f)

Purification via silica gel column chromatography (petroleum ether/acetone/Et_3N = 90/10/1, v/v/v) afforded the desired product **4f** as a white solid (33.6 mg, 54% yield). M.p.: 146-148 °C. ^{1}H NMR (400 MHz, $CDCl_3$): δ = 2.13 (s, 3H), 6.95-6.98 (m, 1H), 7.03 (d, J = 8.4 Hz, 1H), 7.25-7.29 (m, 2H), 7.35 (d, J = 6.4 Hz, 1H), 7.60 (d, J = 6.4 Hz, 1H), 7.70-7.73 (m, 1H), 7.80 (d, J = 4.4 Hz, 1H), 8.05-8.07 (m, 1H) ppm. ^{13}C

NMR (100 MHz, $CDCl_3$): δ = 17.1, 111.1, 118.8, 124.9, 126.2, 126.3, 126.8, 128.7, 132.9, 133.3, 140.0, 146.9, 147.9, 148.8, 162.1 ppm. HRMS (ESI$^+$): calcd for $C_{16}H_{12}N_2NaO_3S$ $[M+Na]^+$ 335.0461, found 335.0471.

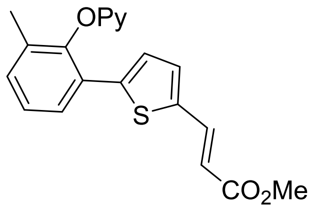

Methyl (*E*)-3-(5-(3-methyl-2-(pyridin-2-yloxy)phenyl)thiophen-2-yl)acrylate (4g)

Purification via silica gel column chromatography (petroleum ether/acetone/Et_3N = 90/10/1, v/v/v) afforded the desired product **4g** as a white solid (61.6 mg, 88% yield). M.p.: 79-81 °C. ^{1}H NMR (400 MHz, $CDCl_3$): δ = 2.13 (s, 3H), 3.76 (s, 3H), 6.40 (d, J = 15.6 Hz, 1H), 6.89-6.93 (m, 2H), 7.11 (d, J = 3.6 Hz, 1H), 7.19-7.28 (m, 3H), 7.56 (d, J = 7.2 Hz, 1H), 7.64-7.69 (m, 2H), 8.09 (d, J = 4.0 Hz, 1H) ppm. ^{13}C NMR (100 MHz, $CDCl_3$): δ = 17.1, 51.8, 110.6, 115.9, 118.3, 126.0, 126.96, 127.01, 127.6, 131.3, 131.4, 132.9, 137.5, 139.4, 139.6, 142.5, 147.9, 148.4, 162.7, 167.5 ppm. HRMS (ESI$^+$): calcd for $C_{20}H_{17}NNaO_3S$ $[M+Na]^+$ 374.0821, found 374.0824.

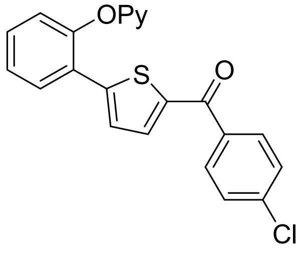

(4-Chlorophenyl)(5-(2-(pyridin-2-yloxy)phenyl)thiophen-2-yl)methanone (4h)

Purification via silica gel column chromatography (petroleum ether/acetone/Et_3N = 90/10/1, v/v/v) afforded the desired product **4h** as a white solid (39.1 mg, 50% yield). M.p.: 119-120 °C. ^{1}H NMR (400 MHz, $CDCl_3$): δ = 6.99-7.03 (m, 2H), 7.21 (d, J = 8.0 Hz, 1H), 7.30 (t, J = 7.2 Hz, 1H), 7.40-7.46 (m, 4H), 7.52 (d, J = 4.0 Hz, 1H), 7.72 (t, J = 6.8 Hz, 1H), 7.77-7.80 (m, 3H), 8.16 (d, J = 3.6 Hz, 1H) ppm. ^{13}C NMR (100 MHz, $CDCl_3$): δ = 112.1, 119.1, 123.4, 125.6, 126.4, 126.8, 128.8, 129.5, 130.1, 130.7, 135.1, 136.6, 138.6, 139.8, 142.5, 147.9, 148.2, 150.8, 163.0, 187.1 ppm. HRMS (ESI$^+$): calcd for $C_{22}H_{15}ClNO_2S$ $[M+H]^+$ 392.0507, 394.0477, found 392.0507,

394.0477.

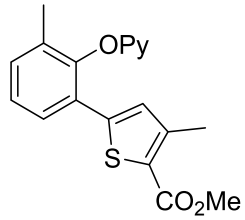

Methyl 3-methyl-5-(3-methyl-2-(pyridin-2-yloxy)phenyl)thiophene-2-carboxyla - te (4i)

Purification via silica gel column chromatography (petroleum ether/acetone/Et_3N = 150/10/1, v/v/v) afforded the desired product **4i** as a white solid (44.1 mg, 65% yield). M.p.: 118-120 °C. ^{1}H NMR (400 MHz, $CDCl_3$): δ = 2.13 (s, 3H), 2.46 (s, 3H), 3.81 (s, 3H), 6.90-6.92 (m, 2H), 7.13 (s, 1H), 7.19-7.28 (m, 2H), 7.55 (d, J = 7.6 Hz, 1H), 7.65 (t, J = 7.6 Hz, 1H), 8.08 (d, J = 3.6 Hz, 1H) ppm. ^{13}C NMR (100 MHz, $CDCl_3$): δ = 16.2, 17.1, 51.7, 110.7, 118.2, 125.9, 126.2, 127.2, 127.4, 130.6, 131.6, 132.9, 139.6, 143.4, 146.1, 147.9, 148.6, 162.6, 163.5 ppm. HRMS (ESI$^+$): calcd for $C_{19}H_{18}NO_3S$ $[M+H]^+$ 340.1002, found 340.1004.

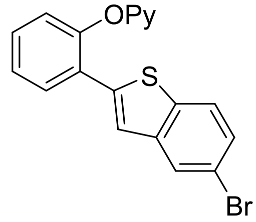

2-(2-(5-Bromobenzo[*b*]thiophen-2-yl)phenoxy)pyridine (4j)

Purification via silica gel column chromatography (petroleum ether/acetone/Et_3N = 120/5/1, v/v/v) afforded the desired product **4j** as a white solid (38.9 mg, 51% yield). M.p.: 99-100 °C. ^{1}H NMR (400 MHz, $CDCl_3$): δ = 6.95-6.99 (m, 2H), 7.21 (d, J = 8.4 Hz, 1H), 7.31 (t, J = 7.2 Hz, 1H), 7.35 (d, J = 8.4 Hz,1H), 7.41 (t, J = 6.8 Hz, 1H), 7.55 (s, 1H), 7.62 (d, J = 8.8 Hz, 1H), 7.68 (t, J = 6.8 Hz, 1H), 7.79 (d, J = 6.0 Hz, 1H), 7.86 (s, 1H), 8.16 (d, J = 3.6 Hz, 1H) ppm. ^{13}C NMR (100 MHz, $CDCl_3$): δ = 111.6, 118.3, 118.8, 121.9, 123.36, 123.38, 125.6, 126.2, 127.1, 127.3, 129.7, 130.1, 138.8, 139.7, 141.2, 141.6, 148.0, 150.8, 163.3 ppm. HRMS (ESI$^+$): calcd for $C_{19}H_{13}BrNOS$ $[M+H]^+$ 381.9896, 383.9875, found381.9891, 383.9871.

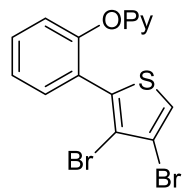

2-(2-(3,4-Dibromothiophen-2-yl)phenoxy)pyridine (4k)

Purification via silica gel column chromatography (petroleum ether/acetone/Et_3N = 150/5/1, v/v/v) afforded the desired product **4k** as yellow oil (41.9 mg, 51% yield). ^1H NMR (400 MHz, DMSO-d_6): δ = 6.91 (d, J = 8.4 Hz, 1H), 7.06 (t, J = 6.0 Hz, 1H), 7.25 (d, J = 8.4 Hz, 1H), 7.36 (t, J = 6.8 Hz, 1H), 7.51-7.56 (m, 2H), 7.77 (t, J = 6.4 Hz, 1H), 7.88 (s, 1H), 8.07 (d, J = 3.6 Hz, 1H) ppm. ^{13}C NMR (100 MHz, DMSO-d_6): δ = 111.1, 112.4, 113.3, 119.0, 122.9, 125.1, 125.5, 131.0, 132.1, 135.0, 140.1, 147.2, 151.4, 162.7 ppm. HRMS (ESI$^+$): calcd for $C_{15}H_{10}Br_2NOS$ $[M+H]^+$ 409.8844, 411.8824, 413.8803, found 409.8850, 411.8830, 413.8808.

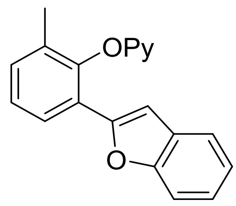

2-(2-(Benzofuran-2-yl)-6-methylphenoxy)pyridine (4l)

Purification via silica gel column chromatography (petroleum ether/acetone/Et_3N = 100/2/1, v/v/v) afforded the desired product **4l** as a white solid (44.1 mg, 73% yield). M.p.: 104-105 °C. ^1H NMR (400 MHz, $CDCl_3$): δ = 2.16 (s, 3H), 6.86-6.92 (m, 2H), 7.07 (s, 1H), 7.14 (t, J = 7.6 Hz, 1H), 7.22 (t, J = 7.6 Hz, 1H), 7.26-7.31 (m, 2H), 7.43-7.48 (m, 2H), 7.63 (t, J = 8.0 Hz, 1H), 7.99 (d, J = 7.2 Hz, 1H), 8.13 (d, J = 4.4 Hz, 1H) ppm. ^{13}C NMR (100 MHz, $CDCl_3$): δ = 16.9, 106.3, 109.7, 111.0, 118.2, 121.2, 122.8, 124.5, 124.6, 125.6, 126.0, 129.5, 131.4, 132.5, 139.7, 148.3, 148.5, 151.8, 154.1, 162.7 ppm. HRMS (ESI$^+$): calcd for $C_{20}H_{15}NNaO_2$ $[M+Na]^+$ 324.0995, found 324.1001.

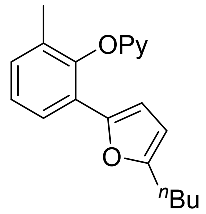

2-(2-(5-Butylfuran-2-yl)-6-methylphenoxy)pyridine (4m)

Purification via silica gel column chromatography (petroleum ether/acetone/Et_3N = 160/4/1, v/v/v) afforded the desired product **4m** as yellow oil (55.2 mg, 90% yield). ^1H NMR (400 MHz, $CDCl_3$): δ = 0.92 (t, J = 7.6 Hz, 3H), δ = 1.32-1.41 (m, 2H), 1.56-1.64 (m, 2H), δ = 2.13 (s, 3H), 2.61 (t, J = 7.6 Hz, 2H), 5.94 (d, J = 3.2 Hz, 1H), 6.55 (d, J = 3.2 Hz, 1H), 6.81 (d, J = 8.0 Hz, 1H), 6.91-6.94 (m, 1H), 7.15 (d, J = 7.6 Hz, 1H), 7.21 (t,J = 7.6 Hz, 1H), 7.61-7.66 (m, 1H), 7.76 (d, J = 8.0 Hz, 1H), 8.15-8.17 (m, 1H) ppm. ^{13}C NMR (100 MHz, $CDCl_3$): δ = 14.0, 16.9, 22.4, 27.9, 30.2, 107.2, 109.6, 110.7, 117.9, 124.1, 125.1, 125.8, 129.5, 132.3, 139.6, 146.9, 148.0, 148.2, 156.0, 163.0 ppm. HRMS (ESI$^+$): calcd for $C_{20}H_{21}NNaO_2$ [M+Na]$^+$ 330.1465, found 330.1466.

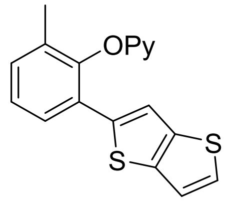

2-(2-Methyl-6-(thieno[3,2-*b*]thiophen-2-yl)phenoxy)pyridine (4n)

Purification via silica gel column chromatography petroleum ether/acetone/Et_3N = 150/5/1, v/v/v) afforded the desired product **4n** as a white solid (28.3 mg, 45% yield). M.p.: 126-127 °C. ^1H NMR (400 MHz, $CDCl_3$): δ = 2.14 (s, 3H), 6.87-6.90 (m, 2H), 7.16 (d, J = 5.2 Hz, 1H), 7.22-7.24 (m, 2H), 7.28 (d, J = 5.2 Hz, 1H), 7.50 (s, 1H), 7.57-7.63 (m, 2H), 8.10 (d, J = 4.4 Hz, 1H) ppm. ^{13}C NMR (100 MHz, $CDCl_3$): δ = 17.1, 110.4, 118.1, 118.3, 119.5, 126.0, 126.9, 127.3, 128.6, 130.8, 132.7, 139.3, 139.5, 139.8, 141.3, 148.0, 148.2, 162.8 ppm. HRMS (ESI$^+$): calcd for $C_{18}H_{13}NNaOS_2$ [M+Na]$^+$ 346.0331, found 346.0342.

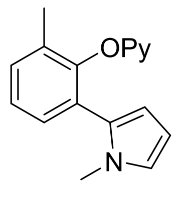

2-(2-Methyl-6-(1-methyl-*1H*-pyrrol-2-yl)phenoxy)pyridine (4o)

Purification via silica gel column chromatography (petroleum ether/acetone/Et_3N = 120/3/1, v/v/v) afforded the desired product **4o** as a white solid (27.2 mg, 52% yield).

M.p.: 91-93 °C. ^1H NMR (400 MHz, $CDCl_3$): δ = 2.12 (s, 3H), 3.56 (s, 3H), 6.40 (t, J = 2.4 Hz, 1H), 6.50 (t, J = 2.4 Hz, 1H), 6.69 (d, J = 8.4 Hz, 1H), 6.87-6.90 (m, 1H), 6.94 (m, 1H), 7.08 (d, J = 7.2 Hz, 1H), 7.16 (t, J = 7.6 Hz, 1H), 7.47 (d, J = 7.6 Hz, 1H), 7.55-7.59 (m, 1H), 8.17-8.19 (m, 1H) ppm. ^{13}C NMR (100 MHz, $CDCl_3$): δ = 17.1, 36.4, 108.1, 109.3, 117.6, 119.9, 121.80, 121.82, 125.8, 126.3, 128.2, 129.6, 132.0, 139.4, 148.0, 148.3, 163.4 ppm. HRMS (ESI$^+$): calcd for $C_{17}H_{16}N_2NaO$ $[M+Na]^+$ 287.1155, found 287.1158.

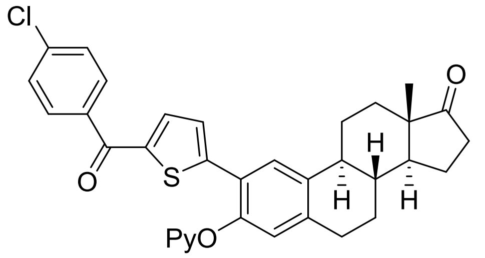

(8*R*,9*S*,13*S*,14*S*)-2-(5-(4-Chlorobenzoyl)thiophen-2-yl)-13-methyl-3-(pyridin-2-yloxy)-6,7,8,9,11,12,13,14,15,16-decahydro-17*H*-cyclopenta[*a*]phenanthren-17-one (4p)

Purification via silica gel column chromatography (petroleum ether/acetone/Et_3N = 90/10/1, v/v/v) afforded the desired product **4p** as a white solid (60.2mg, 53% yield). M.p.: 184-186 °C. ^1H NMR (400 MHz, DMSO-d_6): δ = 0.86 (s, 3H), 1.14-1.21 (m, 2H), 1.35-1.62 (m, 6H), 1.81 (d, J = 6.0 Hz, 1H), 1.94-2.10 (m, 4H), 2.30 (t, J = 12.0 Hz, 1H), 2.42-2.58 (m, 3H), 2.85 (s, 2H), 6.92 (s, 1H), 7.12 (t, J = 8.4 Hz, 2H), 7.60 (d, J = 8.0 Hz, 3H), 7.68 (d, J = 3.6 Hz, 1H), 7.79 (d, J = 8.0 Hz, 3H), 7.88 (t, J = 7.6 Hz, 1H), 8.12 (d, J = 3.6 Hz, 1H) ppm. ^{13}C NMR (100 MHz, DMSO-d_6): δ = 13.5, 21.2, 25.3, 25.7, 28.7, 31.3, 35.4, 37.4, 43.5, 47.3, 49.6, 111.6, 119.2, 123.0, 123.3, 125.5, 126.7, 128.8, 130.6, 135.7, 136.1, 137.2, 137.3, 139.6, 140.3, 141.3, 147.5, 147.6, 148.0, 162.6, 186.0, 219.6 ppm. HRMS (ESI$^+$): calcd for $C_{34}H_{31}ClNO_3S$ $[M+H]^+$ 568.1708, 569.1741, found 568.1705, 569.1744.

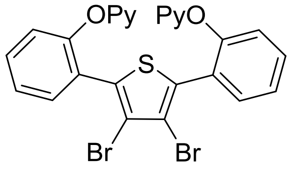

2,2'-(((3,4-Dibromothiophene-2,5-diyl)bis(2,1-phenylene))bis(oxy))dipyridine (4q)

Purification via silica gel column chromatography (petroleum ether/acetone/Et_3N = 90/10/1, v/v/v) afforded the desired product **4q** as a white solid (48.7 mg, 42% yield). M.p.: 40-42 °C. ^{1}H NMR (400 MHz, DMSO-d_6): δ = 6.84 (d, J = 8.3 Hz, 2H), 7.04 (t, J = 6.0 Hz, 2H), 7.19 (d, J = 8.0 Hz, 2H), 7.33 (t, J = 8.0 Hz, 2H), 7.46-7.52 (m, 4H), 7.74 (t, J = 6.8 Hz, 2H), 8.01 (d, J = 3.2 Hz, 2H) ppm. ^{13}C NMR (100 MHz, DMSO-d_6): δ = 111.3, 113.3, 119.1, 122.8, 124.98, 125.03, 131.0, 132.0, 134.3, 140.1, 147.2, 151.5, 162.7 ppm. HRMS (ESI$^+$): calcd for $C_{26}H_{17}Br_2N_2O_2S$ $[M+H]^+$ 578.9372, 580.9352, 582.9331, found 578.9366, 580.9360, 582.9323.

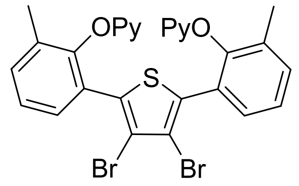

2,2'-(((3,4-Dibromothiophene-2,5-diyl)bis(6-methyl-2,1-phenylene))bis(oxy)) - dipyridine (4r)

Purification via silica gel column chromatography (petroleum ether/acetone/Et_3N = 90/10/1, v/v/v) afforded the desired product **4r** as a white solid (111.9 mg, 92% yield). M.p.: 46-48 °C. ^{1}H NMR (400 MHz, DMSO-d_6): δ = 2.02 (s, 6H), 6.75 (d, J = 8.0 Hz, 2H), 7.01 (t, J = 6.4 Hz, 2H), 7.21-7.27 (m, 4H), 7.39 (d, J = 3.6 Hz, 2H), 7.73 (t, J = 6.4 Hz, 2H), 7.94 (d, J = 3.2 Hz, 2H) ppm. ^{13}C NMR (100 MHz, DMSO-d_6): δ = 16.4, 110.3, 113.1, 118.6, 125.2, 126.1, 129.7, 131.9, 132.4, 134.5, 140.1, 147.0, 149.5, 162.3 ppm. HRMS (ESI$^+$): calcd for $C_{28}H_{21}Br_2N_2O_2S$ $[M+H]^+$ 606.9685, 608.9665, 610.9644, found 606.9688, 608.9660, 610.9651.

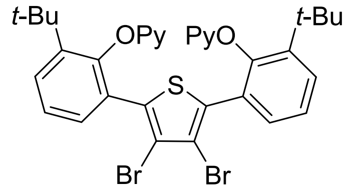

2,2'-(((3,4-Dibromothiophene-2,5-diyl)bis(6-(*tert*-butyl)-2,1-phenylene))bis(oxy)) - dipyridine (4s)

Purification via silica gel column chromatography (petroleum ether/acetone/Et_3N = 90/10/1, v/v/v) afforded the desired product **4s** as a white solid (121.8 mg, 88% yield). M.p.: 185-186 °C. ^{1}H NMR (400 MHz, DMSO-d_6): δ = 1.30 (s, 18H), 6.72 (d, J = 8.4

Hz, 2H), 6.89-6.93 (m, 4H), 7.25 (t, J = 8.0 Hz, 2H), 7.52 (d, J = 7.6 Hz, 2H), 7.64 (d, J = 7.6 Hz, 2H), 7.90 (d, J = 3.6 Hz, 2H) ppm. ^{13}C NMR (100 MHz, DMSO-d_6): δ = 30.3, 34.7, 110.6, 112.9, 118.3, 124.8, 126.8, 128.5, 129.9, 135.3, 139.4, 142.6, 146.9, 150.5, 162.3 ppm. HRMS (ESI$^+$): calcd for $C_{34}H_{33}Br_2N_2O_2S$ [M+H]$^+$ 691.0624, 693.0604, 695.0583, found 691.0625, 693.0611, 695.0584.

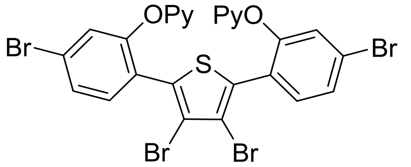

2,2'-(((3,4-Dibromothiophene-2,5-diyl)bis(5-bromo-2,1-phenylene))bis(oxy)) -dipyridine (4t)

Purification via silica gel column chromatography (petroleum ether/acetone/Et_3N = 90/10/1, v/v/v) afforded the desired product **4t** as a white solid (135.8 mg, 92% yield). M.p.: 61-62 °C. ^{1}H NMR (400 MHz, DMSO-d_6): δ = 6.86 (d, J = 8.4 Hz, 2H), 7.07 (t, J = 6.0 Hz, 2H), 7.43 (d, J = 8.4 Hz, 2H), 7.49 (s, 2H), 7.54 (d, J = 8.4 Hz, 2H), 7.76 (t, J = 8.0 Hz, 2H), 8.03 (d, J = 3.6 Hz, 2H) ppm. ^{13}C NMR (100 MHz, DMSO-d_6): δ = 111.4, 113.9, 119.5, 123.1, 124.4, 125.9, 128.1, 133.5, 140.3, 147.2, 152.0, 162.2 ppm. HRMS (ESI$^+$): calcd for $C_{26}H_{15}Br_4N_2O_2S$ [M+H]$^+$ 736.7562, 738.7541, 740.7521, found 736.7566, 738.7545, 740.7531.

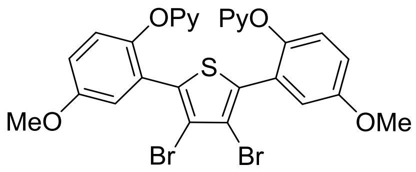

2,2'-(((3,4-Dibromothiophene-2,5-diyl)bis(4-methoxy-2,1-phenylene))bis(oxy)) -dipyridine (4u)

Purification via silica gel column chromatography (petroleum ether/acetone/Et_3N = 80/20/1, v/v/v) afforded the desired product **4u** as a white solid (87.1 mg, 68% yield). M.p.: 37-39 °C. ^{1}H NMR (400 MHz, DMSO-d_6): δ = 3.79 (s, 6H), 6.76 (d, J = 8.0 Hz, 2H), 6.98-7.08 (m, 6H), 7.13 (d, J = 8.8 Hz, 2H), 7.72 (t, J = 8.0 Hz, 2H), 7.98 (d, J = 4.4 Hz, 2H) ppm. ^{13}C NMR (100 MHz, DMSO-d_6): δ = 55.7, 110.9, 113.3, 114.4, 118.8, 124.2, 125.9, 134.1, 139.9, 144.6, 147.1, 155.9, 163.1 ppm. HRMS (ESI$^+$):

calcd for $C_{28}H_{21}Br_2N_2O_4S$ $[M+H]^+$ 638.9583, 640.9563, 642.9542, found 638.9574, 640.9562, 642.9561.

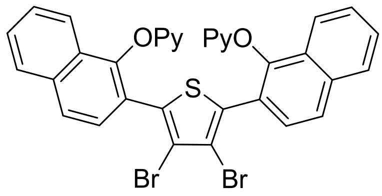

2,2'-(((3,4-Dibromothiophene-2,5-diyl)bis(naphthalene-2,1-diyl))bis(oxy)) -dipyridine (4v)

Purification via silica gel column chromatography (petroleum ether/acetone/Et_3N = 90/10/1, v/v/v) afforded the desired product **4v** as a white solid (86.9 mg, 64% yield). M.p.: 94-95 °C. ^1H NMR (400 MHz, DMSO-d_6): δ = 6.96 (d, J = 8.0 Hz, 2H), 7.03 (t, J = 6.0 Hz, 2H), 7.49-7.55 (m, 4H), 7.61 (d, J = 7.6 Hz, 2H), 7.75 (d, J = 8.4 Hz, 2H), 7.81 (t, J = 7.6 Hz, 2H), 7.85 (d, J = 4.8 Hz, 2H), 7.94 (d, J = 8.8 Hz, 2H), 8.04 (d, J = 8.4 Hz, 2H) ppm. ^{13}C NMR (100 MHz, DMSO-d_6): δ = 110.3, 113.7, 118.9, 122.1, 122.3, 125.4, 127.2, 127.4, 127.5, 128.1, 128.2, 134.7, 140.2, 147.06, 147.12, 163.3 ppm. HRMS (ESI$^+$): calcd for $C_{34}H_{20}Br_2N_2NaO_2S$ $[M+Na]^+$ 700.9504, 702.9484, 704.9464, found 700.9503, 702.9479, 704.9476.

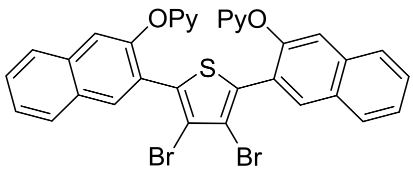

2,2'-(((3,4-Dibromothiophene-2,5-diyl)bis(naphthalene-3,2-diyl))bis(oxy)) -dipyridine (4w)

Purification via silica gel column chromatography (petroleum ether/acetone/Et_3N = 90/10/1, v/v/v) afforded the desired product **4w** as a white solid (65.2 mg, 48% yield). M.p.: 61-63 °C. ^1H NMR (400 MHz, DMSO-d_6): δ = 6.92 (d, J = 8.4 Hz, 2H), 7.05 (t, J = 6.0 Hz, 2H), 7.52-7.60 (m, 4H), 7.75-7.79 (m, 4H), 7.92 (d, J = 7.6 Hz, 2H), 8.02 (d, J = 6.8 Hz, 4H), 8.07 (s, 2H) ppm.^{13}C NMR (100 MHz, DMSO-d_6): δ = 111.4, 113.8, 119.15, 119.21, 125.1, 126.1, 127.1, 127.7, 128.1, 130.1, 132.2, 134.0, 134.6, 140.1, 147.3, 149.2, 162.9 ppm. HRMS (ESI$^+$): calcd for $C_{34}H_{21}Br_2N_2O_2S$ $[M+H]^+$ 678.9685, 680.9665, 682.9644, found 678.9686, 680.9674, 682.9653.

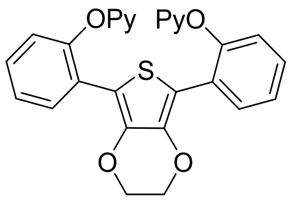

5,7-Bis(2-(pyridin-2-yloxy)phenyl)-2,3-dihydrothieno[3,4-*b*][1,4]dioxine (4x)

Purification via silica gel column chromatography (petroleum ether/acetone/Et_3N = 100/25/1, v/v/v) afforded the desired product **4x** as a white solid (56.7 mg, 59% yield). M.p.: 149-150 °C. ^1H NMR (400 MHz, DMSO-d_6): δ = 4.26 (s, 4H), 6.81 (d, J = 8.0 Hz, 2H), 7.04-7.10 (m, 4H), 7.23-7.30 (m, 4H), 7.76 (d, J = 7.6 Hz, 2H), 7.97 (d, J = 7.2 Hz, 2H), 8.03 (d, J = 3.6 Hz, 2H) ppm.^{13}C NMR (100 MHz, DMSO-d_6): δ = 64.2, 110.9, 111.5, 118.8, 123.3, 124.8, 125.2, 127.8, 129.5, 138.7, 139.9, 147.2, 149.3, 162.6 ppm. HRMS (ESI$^+$): calcd for $C_{28}H_{21}N_2O_4S$ $[M+H]^+$ 481.1217, found 481.1221.

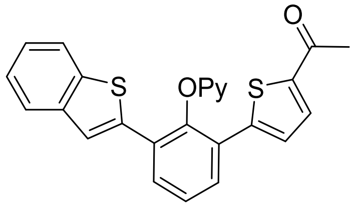

1-(5-(3-(Benzo[*b*]thiophen-2-yl)-2-(pyridin-2-yloxy)phenyl)thiophen-2-yl)ethan-1-one (4y)

Purification via silica gel column chromatography (petroleum ether/acetone/Et_3N = 80/20/1, v/v/v) afforded the desired product **4y** as a white solid (64.9 mg, 76% yield). M.p.: 180-182 °C. ^1H NMR (400 MHz, DMSO-d_6): δ = 2.47 (s, 3H), 6.90 (t, J = 6.0 Hz, 1H), 7.16 (d, J = 8.0 Hz, 1H), 7.28-7.35 (m, 2H), 7.51 (t, J = 8.0 Hz, 1H), 7.62 (d, J = 4.0 Hz, 1H), 7.74 (d, J = 7.2 Hz, 1H), 7.79 (d, J = 7.6 Hz, 2H), 7.84 (t, J = 5.2 Hz, 2H), 7.87-7.94 (m, 3H) ppm. ^{13}C NMR (100 MHz, DMSO-d_6): δ = 26.5, 111.6, 118.9, 122.1, 123.7, 123.8, 124.6, 124.8, 126.7, 128.1, 128.4, 129.3, 129.6, 130.7, 133.8, 137.9, 139.2, 139.4, 140.0, 143.9, 145.6, 146.4, 147.0, 161.6, 190.9 ppm. HRMS (ESI$^+$): calcd for $C_{25}H_{18}NO_2S_2$ $[M+H]^+$ 428.0773, found 428.0771.

XII. References

(1) J. W. Kang, K. Moseley and P. M. Maitlis, *J. Am. Chem. Soc.*, 1969, **91**, 5970.

(2) D. Maiti and S. L. Buchwald, *J. Org. Chem.*, 2010, **75**, 1791.

(3) (a) J.-H. Chu, P.-S. Lin and M.-J. Wu, *Organometallics*, 2010, **29**, 4058; (b) L.

Ackermann, E. Diers and A. Manvar, *Org. Lett.*, 2012, **14**, 1154.

(4) A. Martínez, M. Fernández, J. C. Estévez, R. J. Estévez and L. Castedo, *Tetrahedron*, 2005, **61**, 1353.

XIII. Copies of ^{1}H and ^{13}C NMR spectra

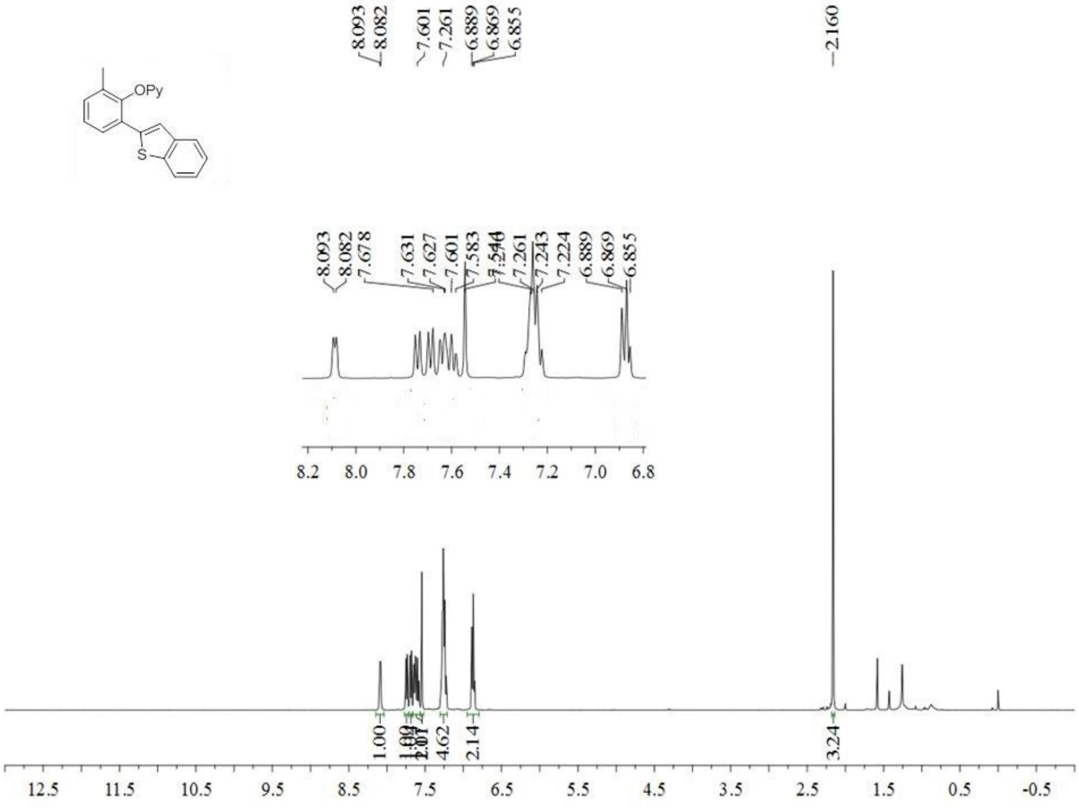

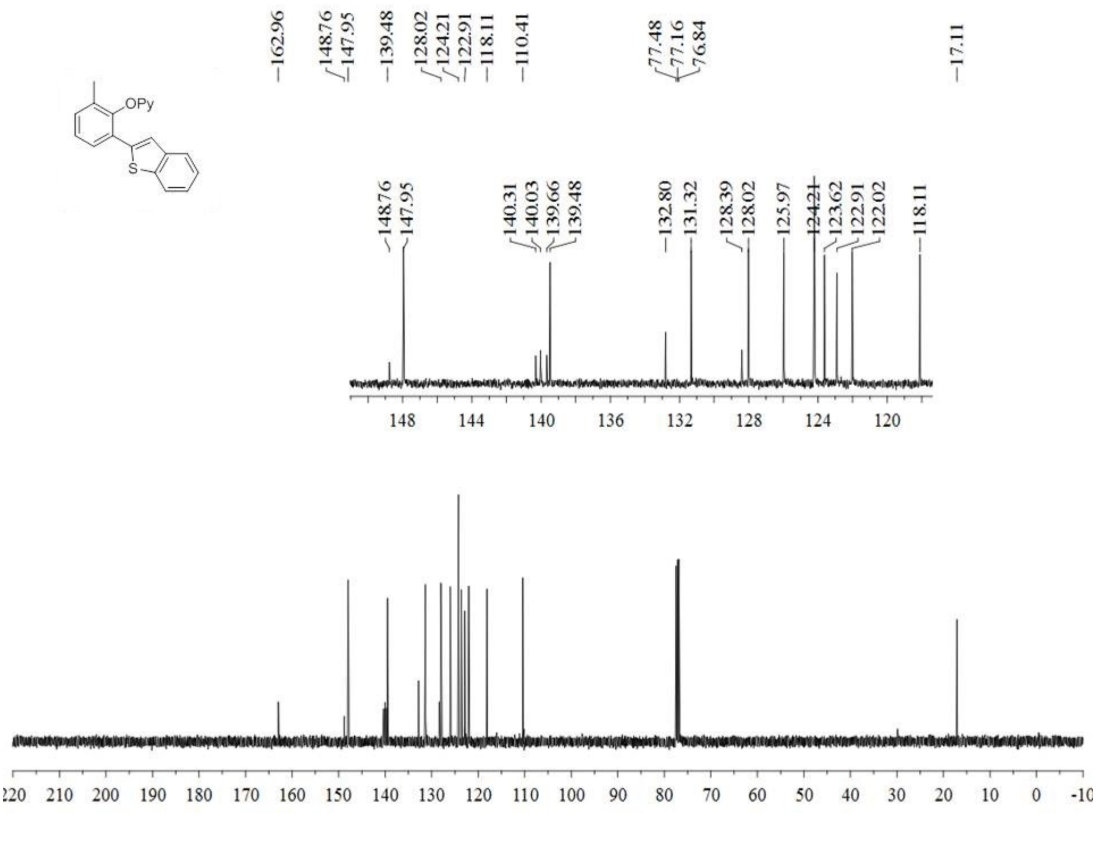

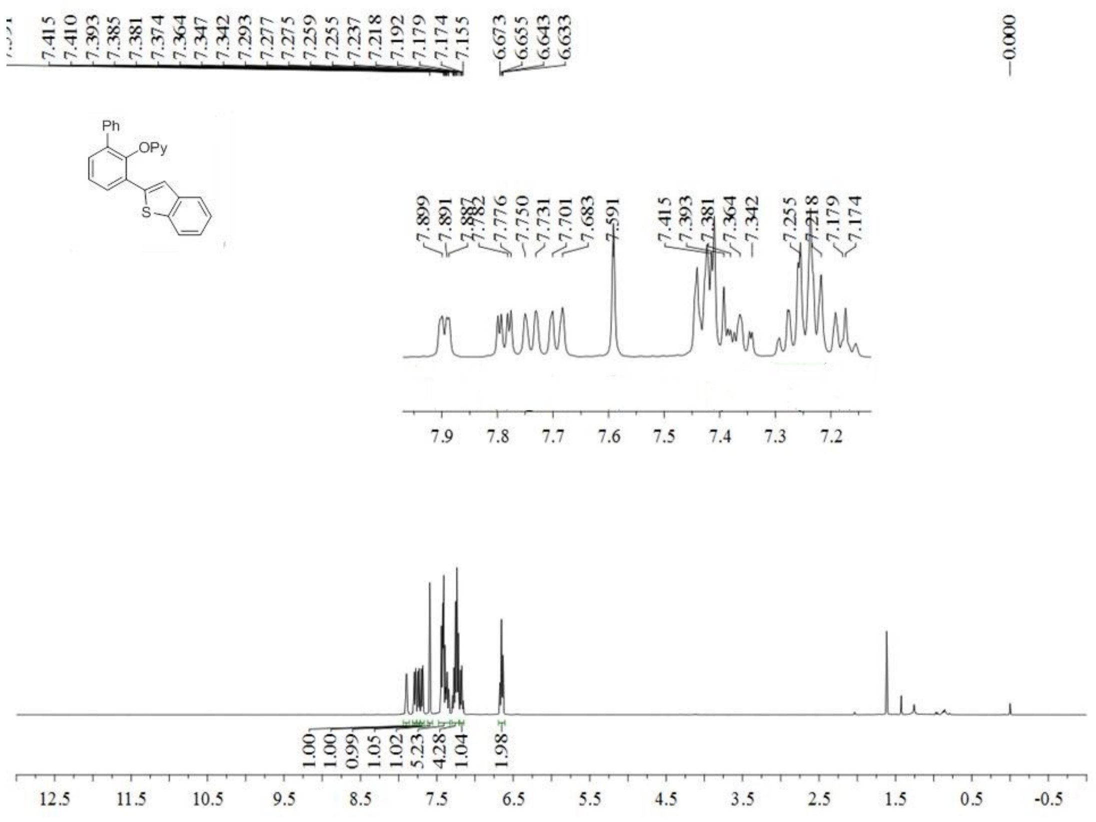
7.415
7.410
7.393
7.385
7.381
7.374
7.364
7.347
7.342
7.293
7.277
7.275
7.259
7.255
7.237
7.218
7.192
7.179
7.174
7.155
6.673
6.655
6.643
6.633
0.000
Ph
OPy
S
7.899
7.891
7.887
7.782
7.776
7.750
7.731
7.701
7.683
7.591
7.415
7.393
7.381
7.364
7.342
7.255
7.218
7.179
7.174
7.9 7.8 7.7 7.6 7.5 7.4 7.3 7.2
1.00
1.00
0.99
1.05
1.02
5.23
4.28
1.04
1.98
12.5 11.5 10.5 9.5 8.5 7.5 6.5 5.5 4.5 3.5 2.5 1.5 0.5 -0.5

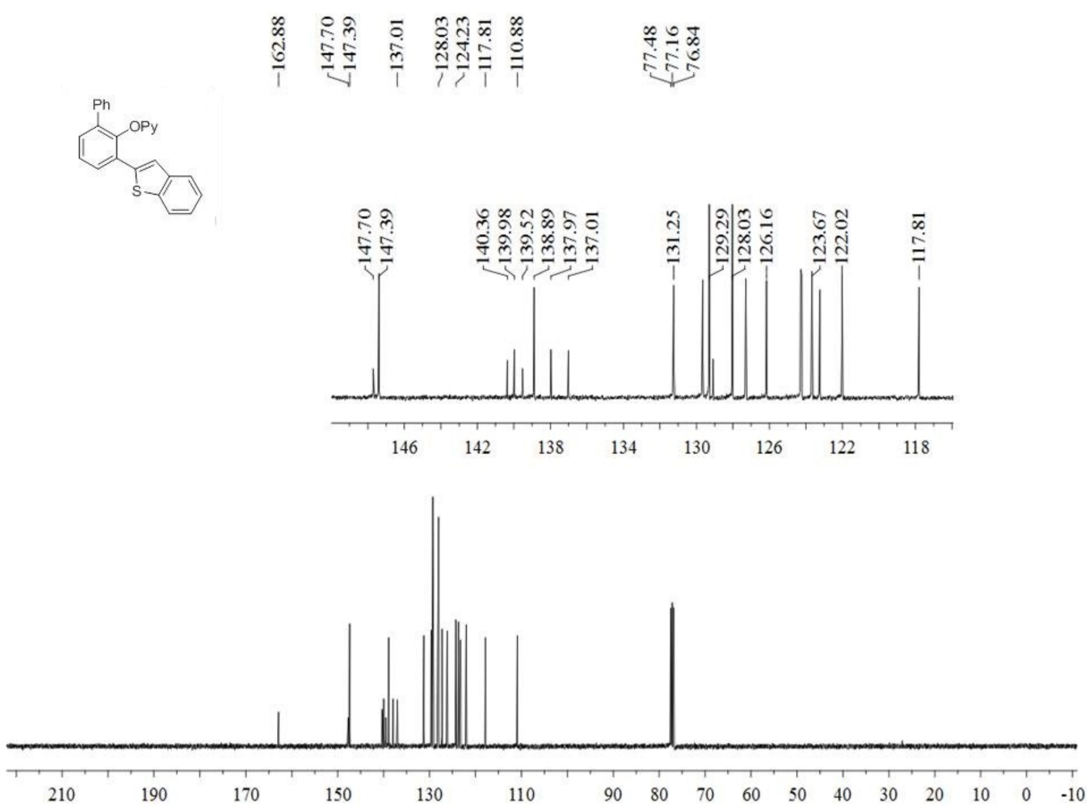
162.88
147.70
147.39
137.01
128.03
124.23
117.81
110.88
77.48
77.16
76.84
Ph
OPy
S
147.70
147.39
140.36
139.98
139.52
138.89
137.97
137.01
131.25
129.29
128.03
126.16
123.67
122.02
117.81
146 142 138 134 130 126 122 118
210 190 170 150 130 110 90 80 70 60 50 40 30 20 10 0 -10

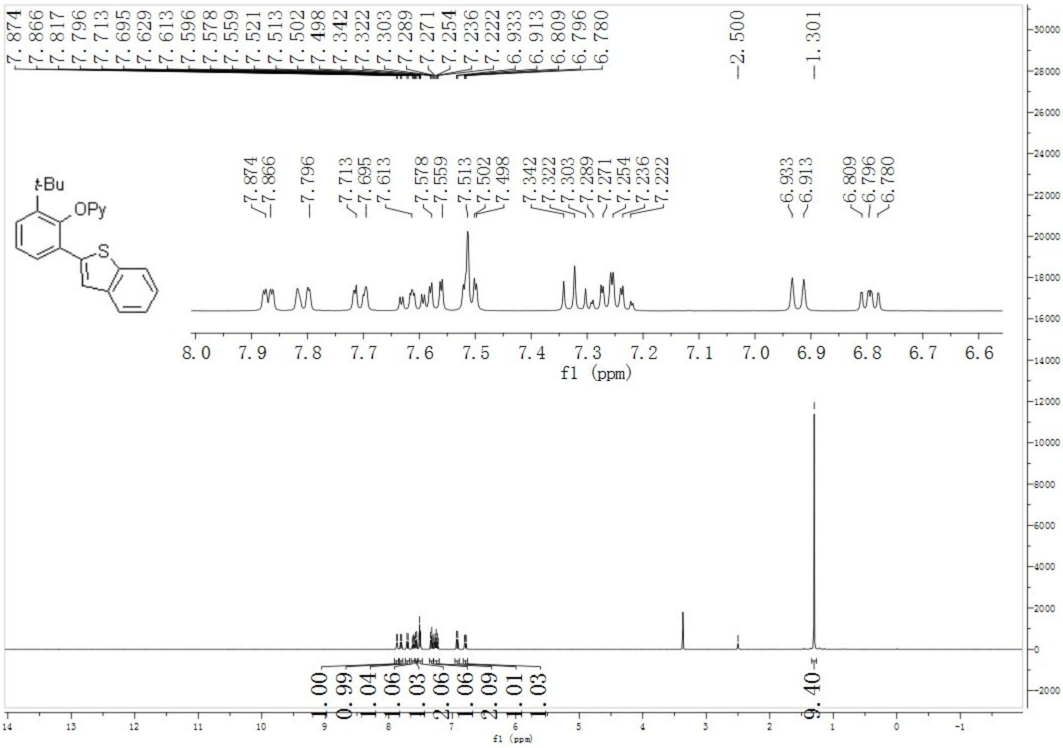
t-Bu
OPy
S
f1 (ppm)

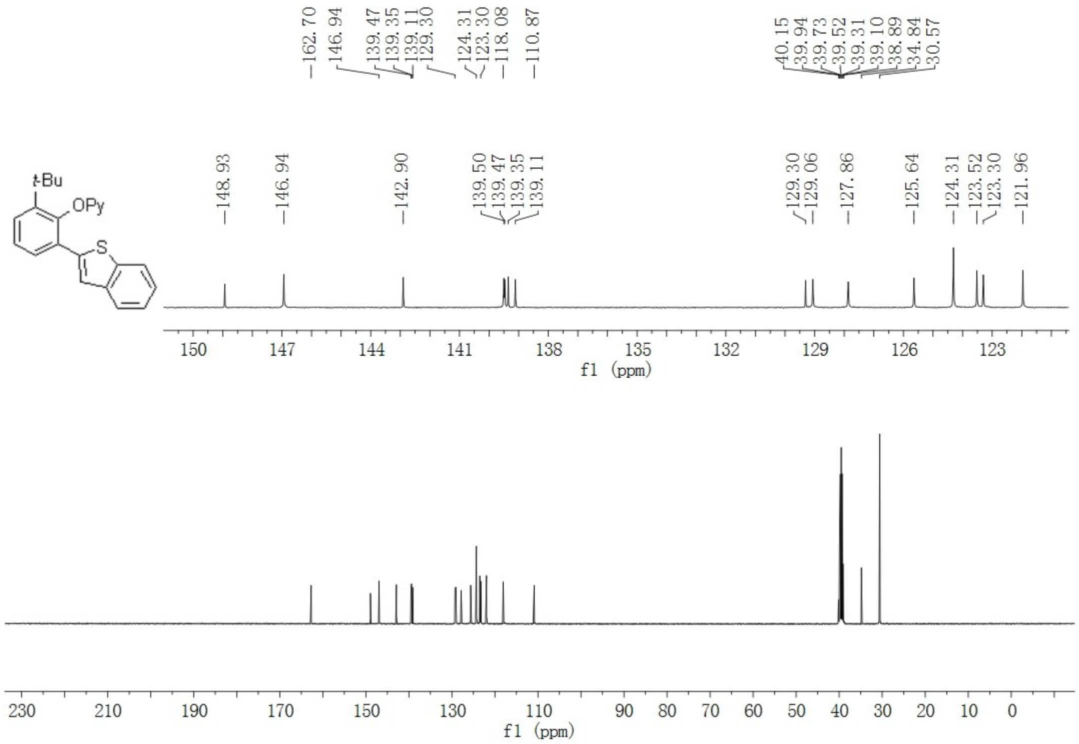
t-Bu
OPy
S
f1 (ppm)

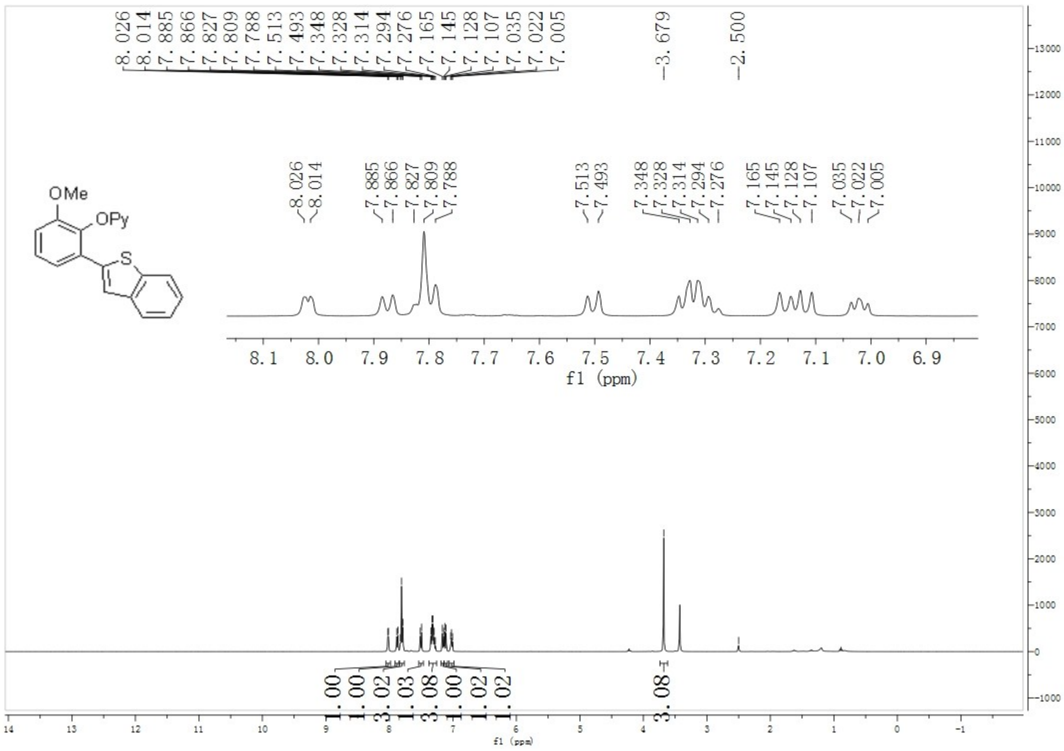

8.026 8.014 7.885 7.866 7.827 7.809 7.788 7.513 7.493 7.348 7.328 7.314 7.294 7.276 7.165 7.145 7.128 7.107 7.035 7.022 7.005
3.679
2.500
OMe
OPy
S
f1 (ppm)
1.00 1.00 3.02 1.03 3.08 1.00 1.02 1.02
3.08

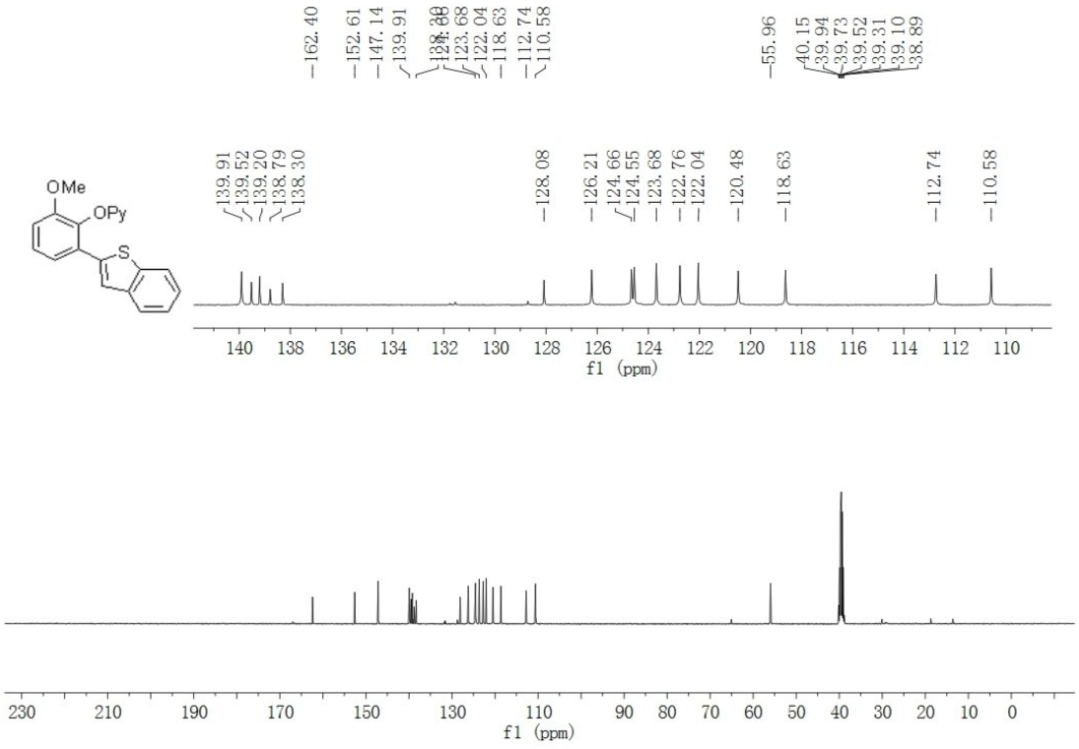

162.40 152.61 147.14 139.91 128.08 124.66 123.68 122.04 118.63 112.74 110.58
55.96
40.15 39.94 39.73 39.52 39.31 39.10 38.89
139.91 139.52 139.20 138.79 138.30
128.08 126.21 124.66 124.55 123.68 122.76 122.04 120.48 118.63 112.74 110.58
OMe
OPy
S
f1 (ppm)

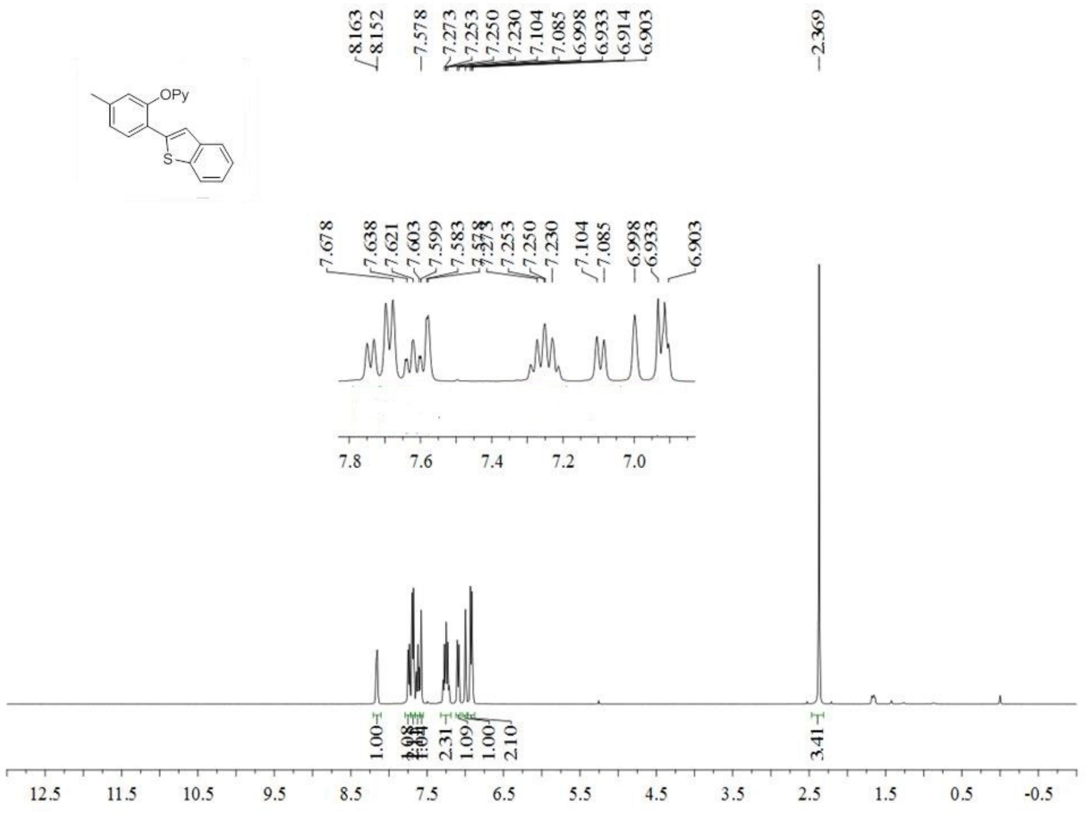
OPy
S
8.163
8.152
7.578
7.273
7.253
7.250
7.230
7.104
7.085
6.998
6.933
6.914
6.903
2.369
7.678
7.638
7.621
7.603
7.599
7.583
1.00
2.31
1.09
1.00
2.10
3.41

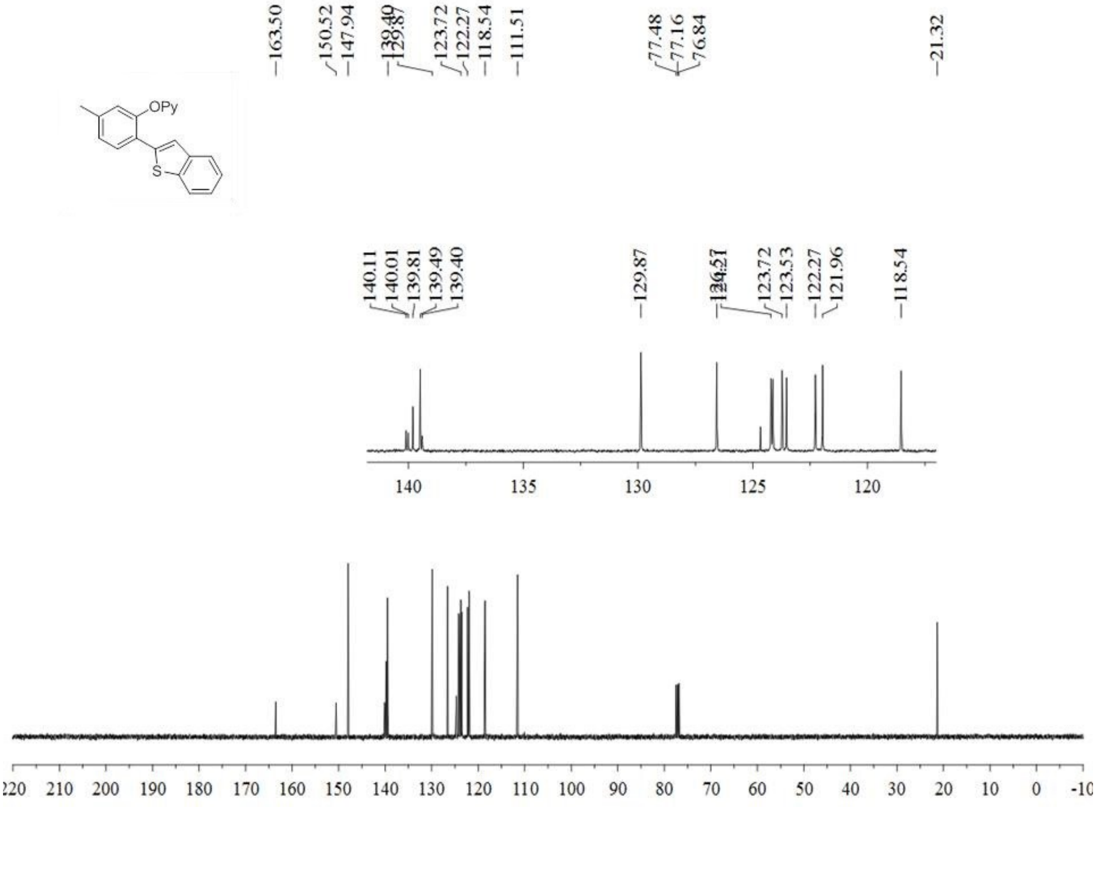
OPy
S
163.50
150.52
147.94
123.72
122.27
118.54
111.51
77.48
77.16
76.84
21.32
140.11
140.01
139.81
139.49
139.40
129.87
123.72
123.53
122.27
121.96
118.54

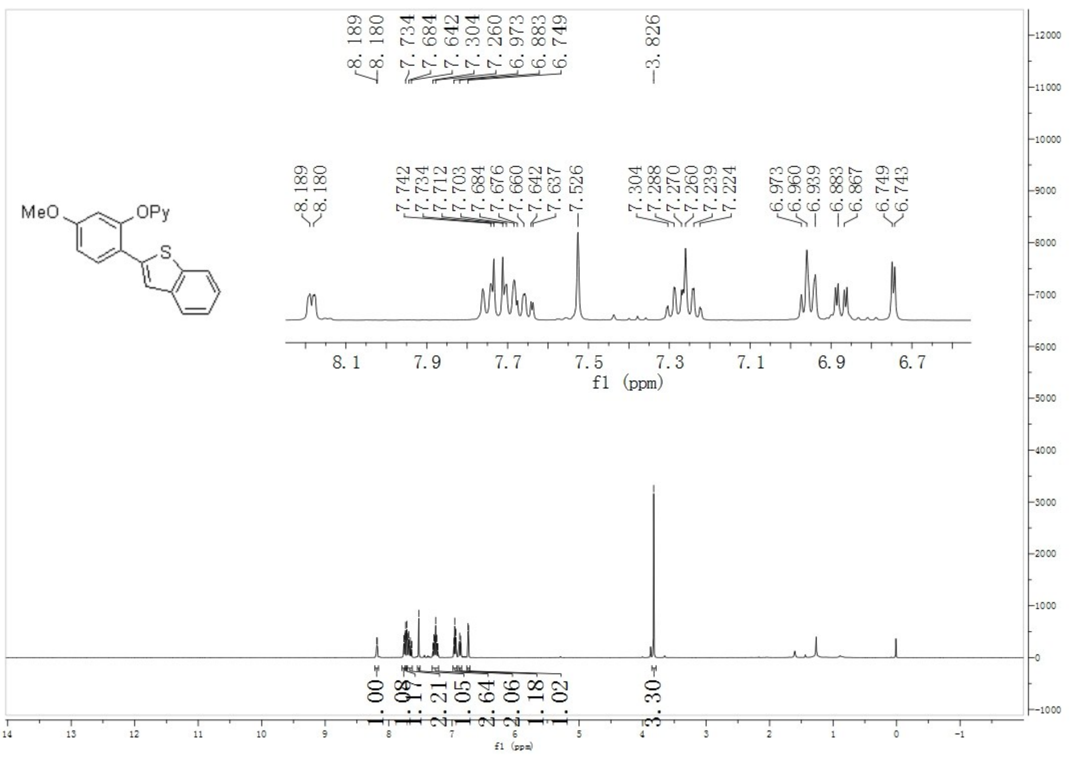
MeO
OPy
S
8.189
8.180
7.734
7.684
7.642
7.304
7.260
6.973
6.883
6.749
3.826
7.742
7.734
7.712
7.703
7.684
7.676
7.660
7.642
7.637
7.526
7.304
7.288
7.270
7.260
7.239
7.224
6.973
6.960
6.939
6.883
6.867
6.749
6.743
1.00
1.08
1.17
2.21
1.05
2.64
2.06
1.18
1.02
3.30
f1 (ppm)

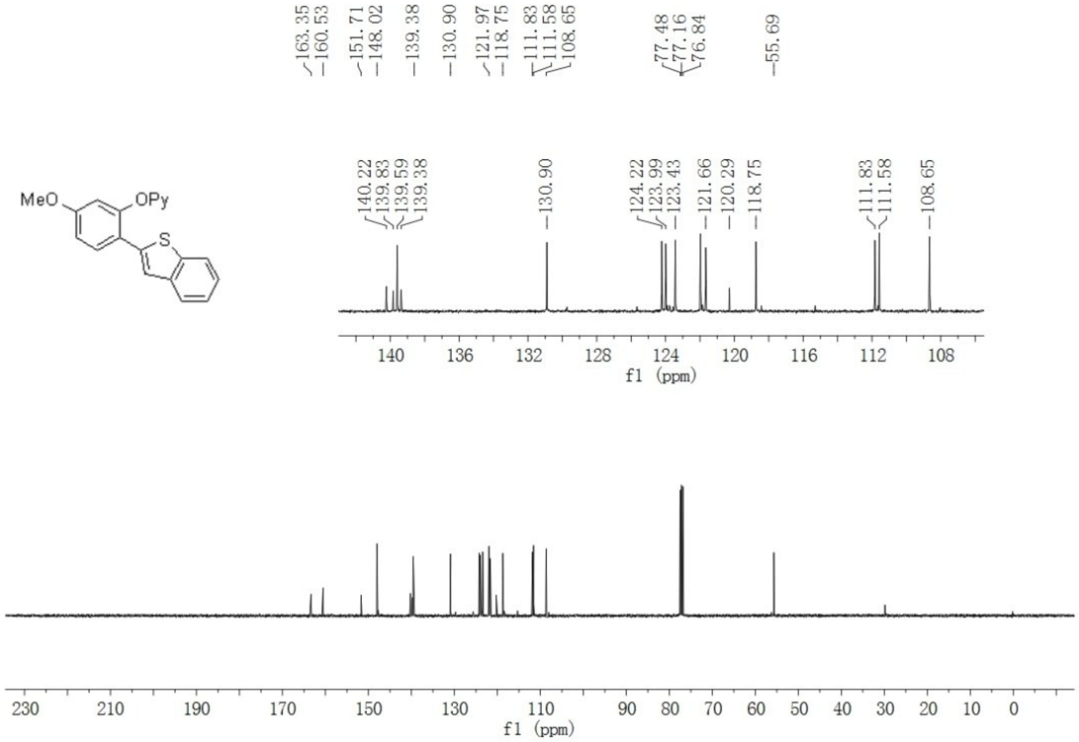
163.35
160.53
151.71
148.02
139.38
130.90
121.97
118.75
111.83
111.58
108.65
77.48
77.16
76.84
55.69
MeO
OPy
S
140.22
139.83
139.59
139.38
130.90
124.22
123.99
123.43
121.66
120.29
118.75
111.83
111.58
108.65
f1 (ppm)

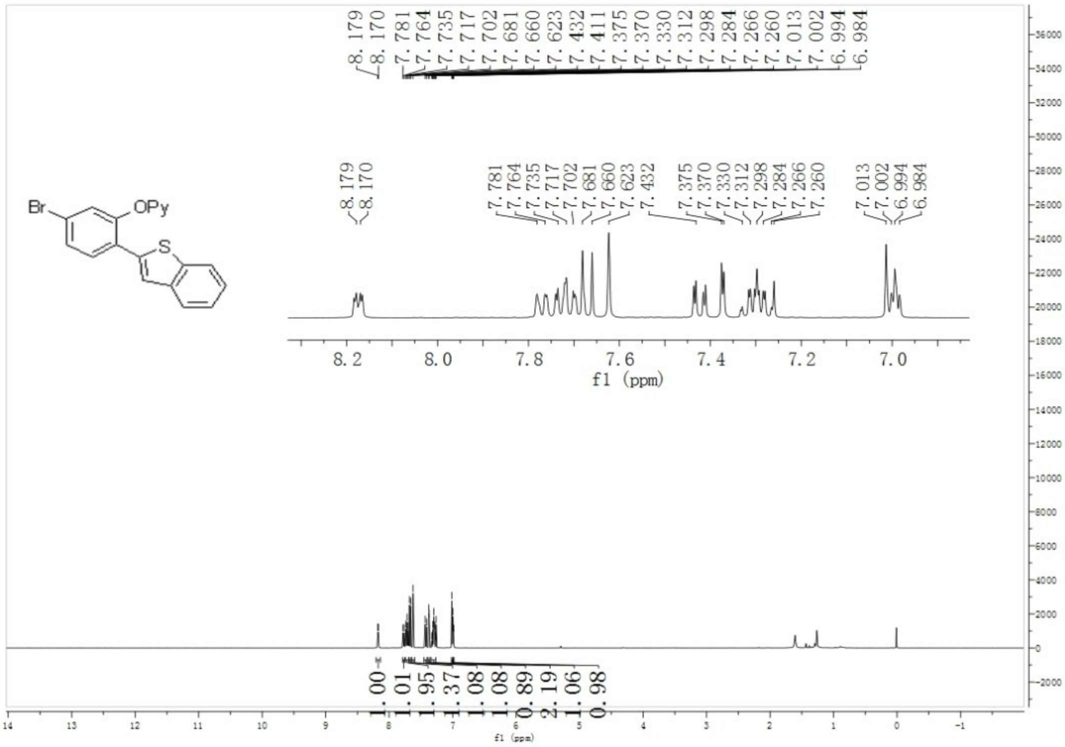

Br
OPy
S
8.179
8.170
7.781
7.764
7.735
7.717
7.702
7.681
7.660
7.623
7.432
7.411
7.375
7.370
7.330
7.312
7.298
7.284
7.266
7.260
7.013
7.002
6.994
6.984
f1 (ppm)
1.00
1.01
1.95
1.37
1.08
1.08
0.89
2.19
1.06
0.98

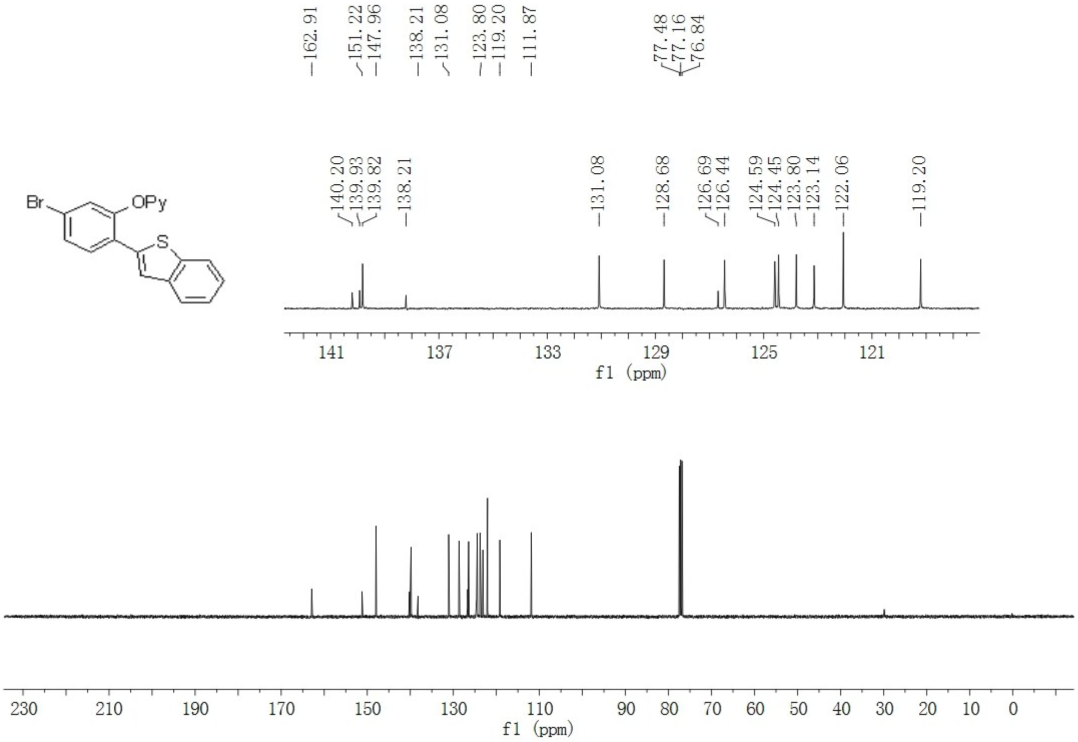

Br
OPy
S
162.91
151.22
147.96
138.21
131.08
123.80
119.20
111.87
77.48
77.16
76.84
140.20
139.93
139.82
138.21
131.08
128.68
126.69
126.44
124.59
124.45
123.80
123.14
122.06
119.20
f1 (ppm)

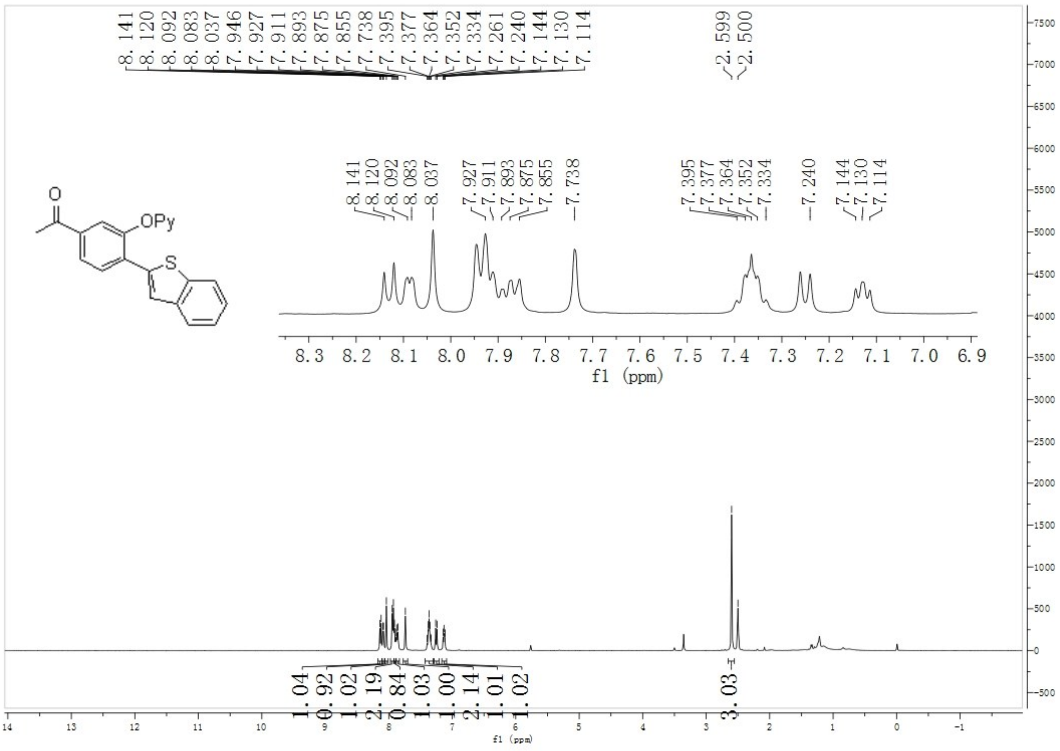
8.141 8.120 8.092 8.083 8.037 7.946 7.927 7.911 7.893 7.875 7.855 7.738 7.395 7.377 7.364 7.352 7.334 7.261 7.240 7.144 7.130 7.114
2.599 2.500
O
OPy
S
8.141 8.120 8.092 8.083 8.037 7.927 7.911 7.893 7.875 7.855 7.738 7.395 7.377 7.364 7.352 7.334 7.240 7.144 7.130 7.114
8.3 8.2 8.1 8.0 7.9 7.8 7.7 7.6 7.5 7.4 7.3 7.2 7.1 7.0 6.9
f1 (ppm)
1.04 0.92 1.02 2.19 0.84 1.03 1.00 2.14 1.01 1.02
3.03
14 13 12 11 10 9 8 7 6 5 4 3 2 1 0 -1
f1 (ppm)
7500 7000 6500 6000 5500 5000 4500 4000 3500 3000 2500 2000 1500 1000 500 0 -500

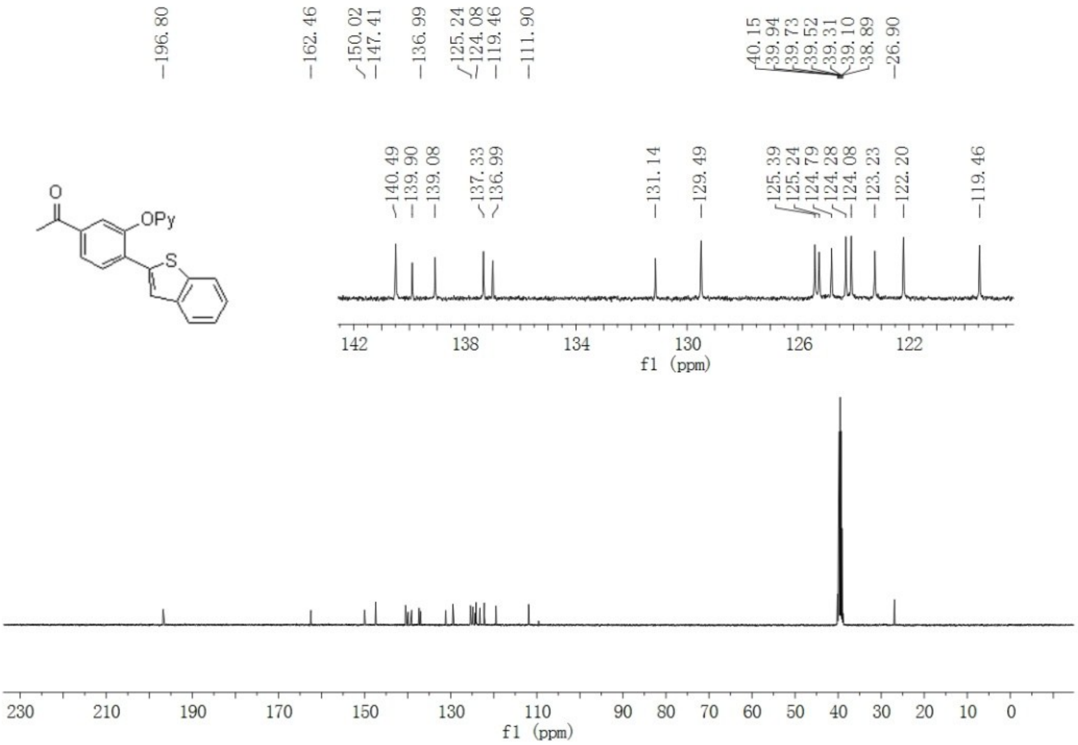
196.80
162.46
150.02 147.41
136.99
125.24 124.08 119.46
111.90
40.15 39.94 39.73 39.52 39.31 39.10 38.89
26.90
O
OPy
S
140.49 139.90 139.08
137.33 136.99
131.14
129.49
125.39 125.24 124.79 124.28 124.08
123.23
122.20
119.46
142 138 134 130 126 122
f1 (ppm)
230 210 190 170 150 130 110 90 80 70 60 50 40 30 20 10 0
f1 (ppm)

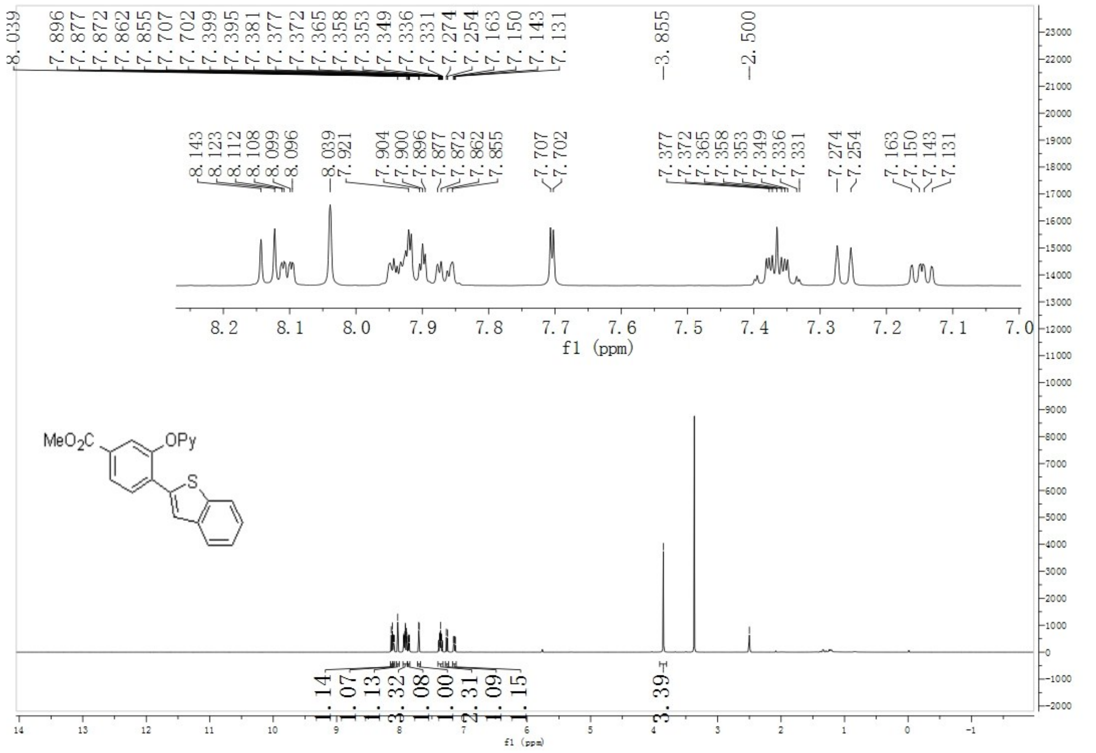

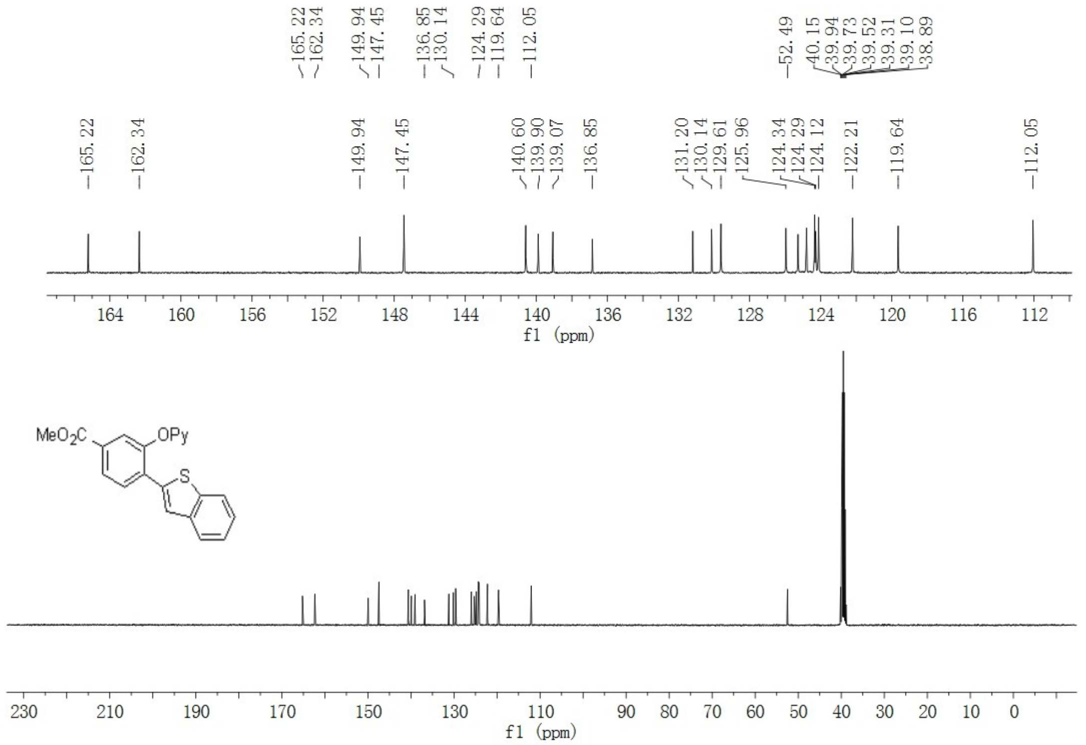

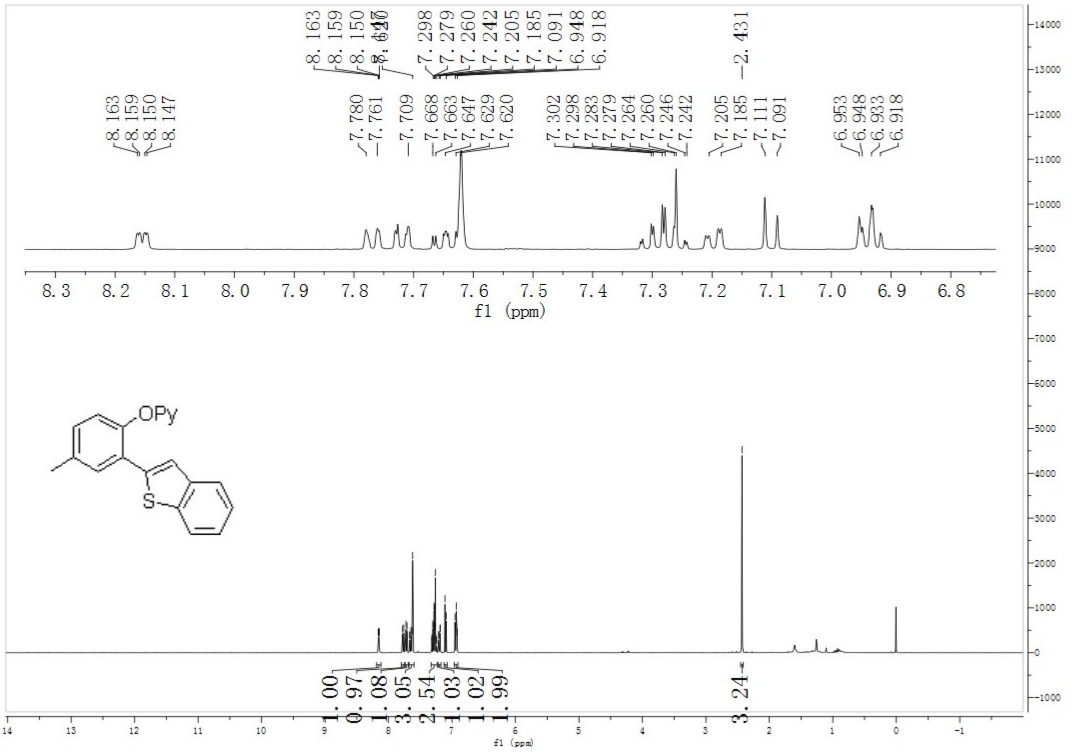
8.163
8.159
8.150
8.147
7.780
7.761
7.709
7.668
7.663
7.647
7.629
7.620
7.302
7.298
7.283
7.279
7.264
7.260
7.246
7.242
7.205
7.185
7.111
7.091
6.953
6.948
6.933
6.918
2.431
OPy
S
1.00
0.97
1.08
3.05
2.54
1.03
1.02
1.99
3.24
f1 (ppm)

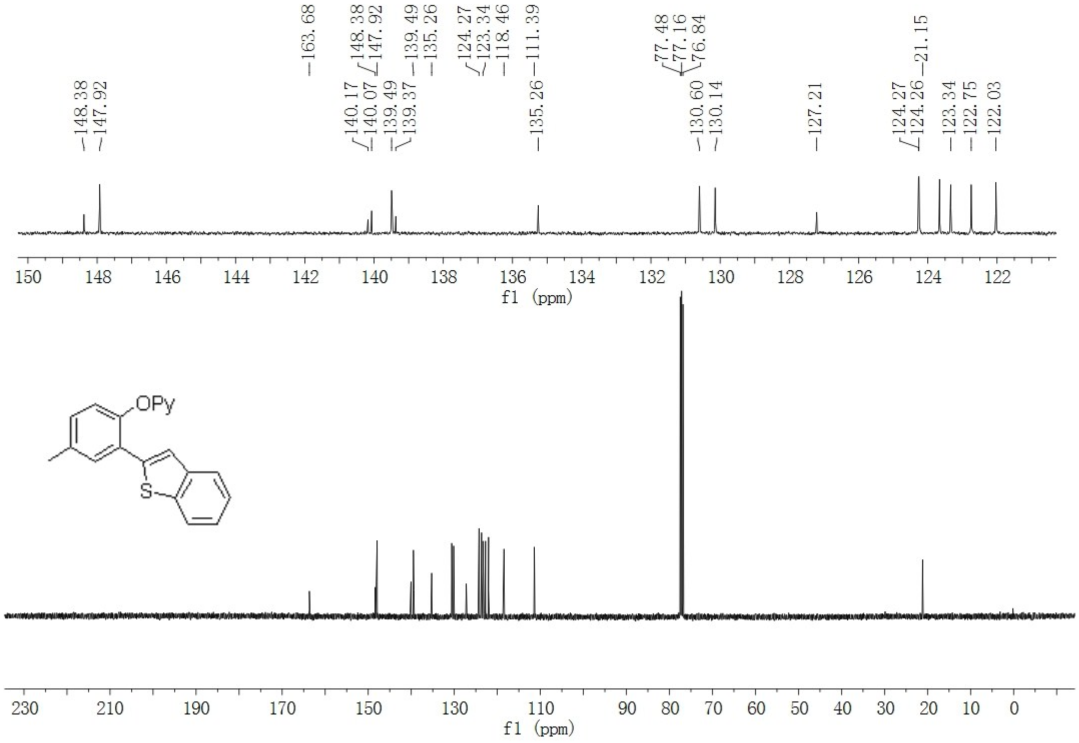
163.68
148.38
147.92
140.17
140.07
139.49
139.37
135.26
130.60
130.14
127.21
124.27
124.26
123.34
122.75
122.03
118.46
111.39
77.48
77.16
76.84
21.15
OPy
S
f1 (ppm)

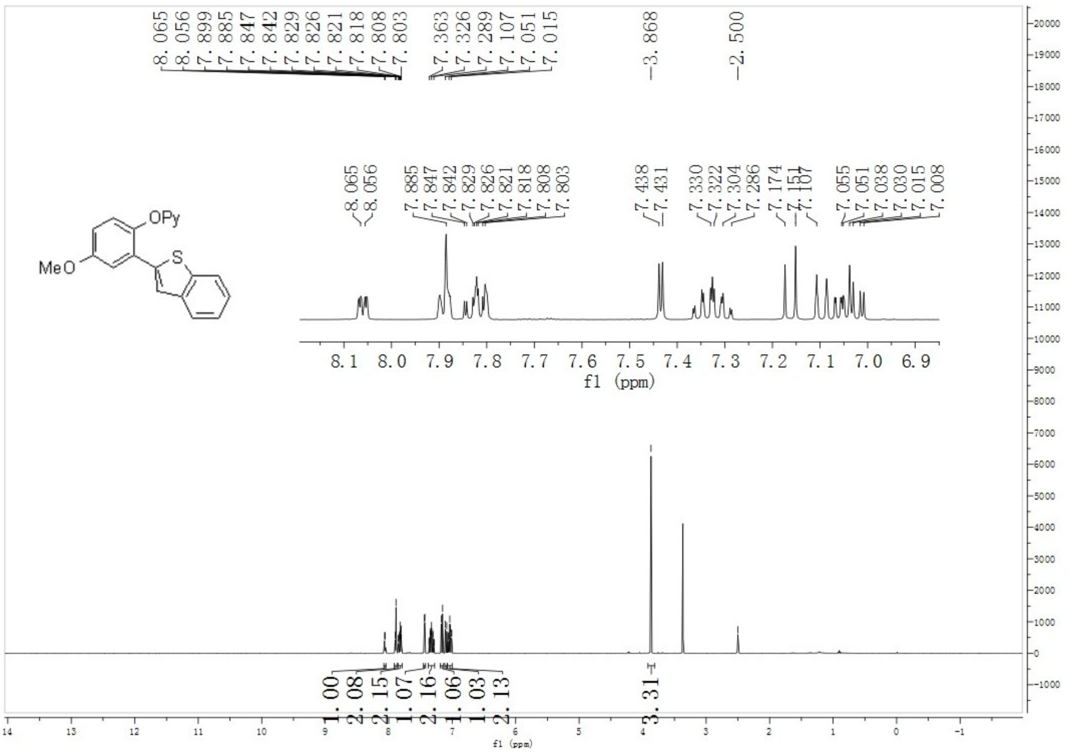

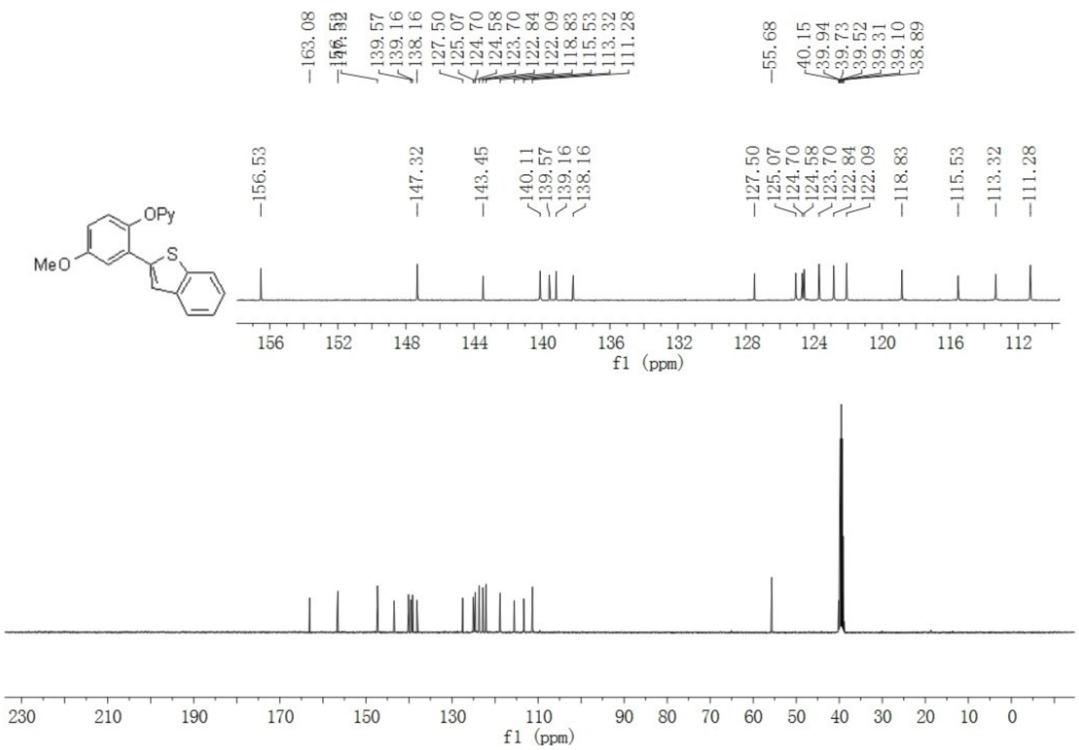

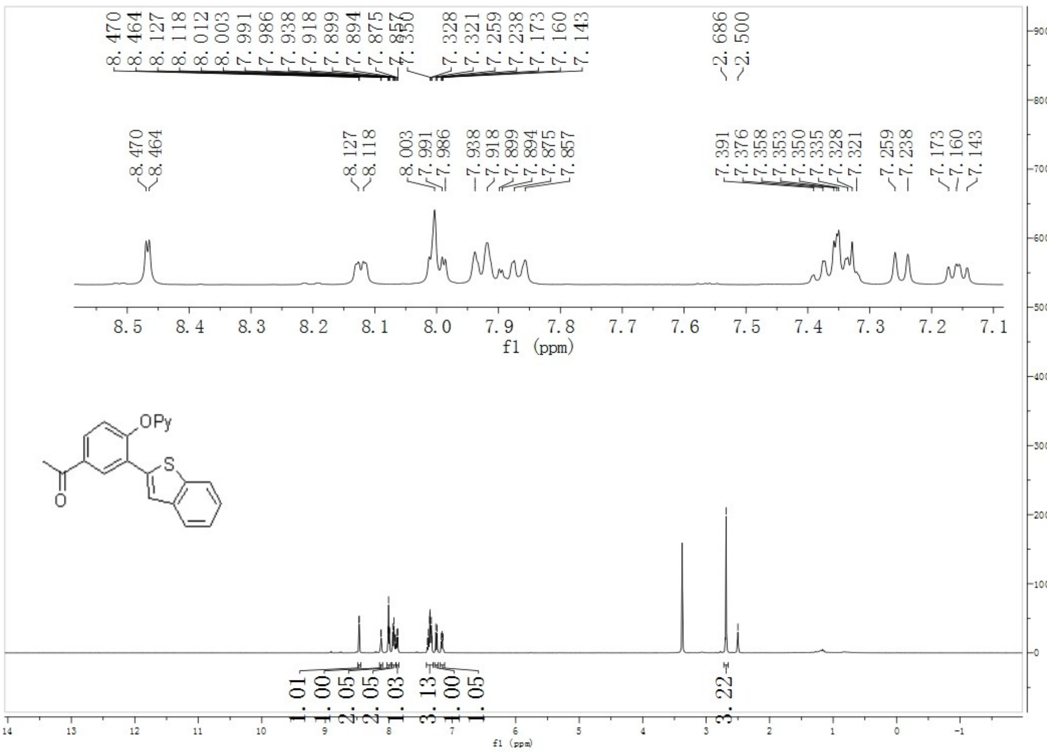

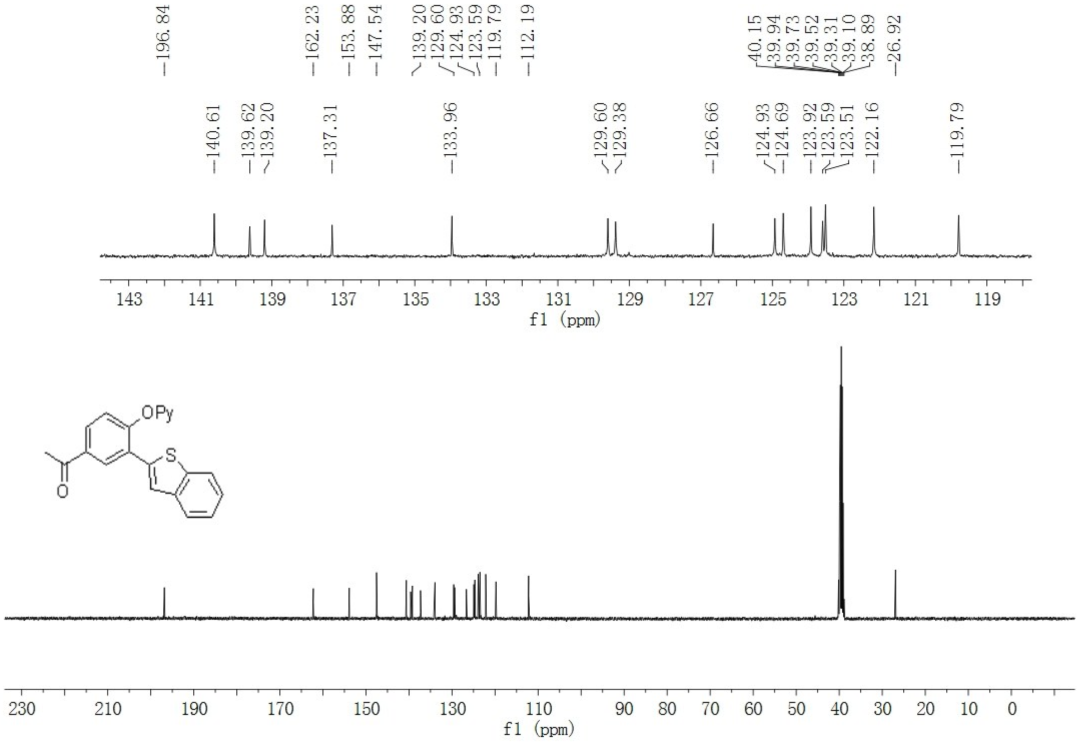

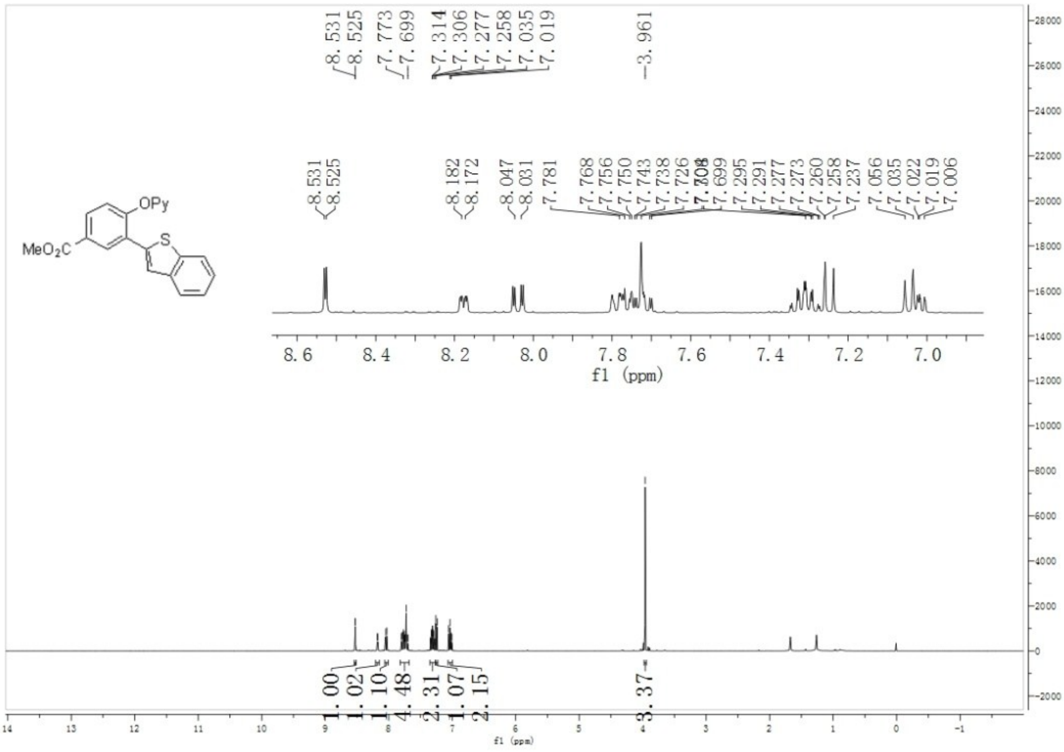

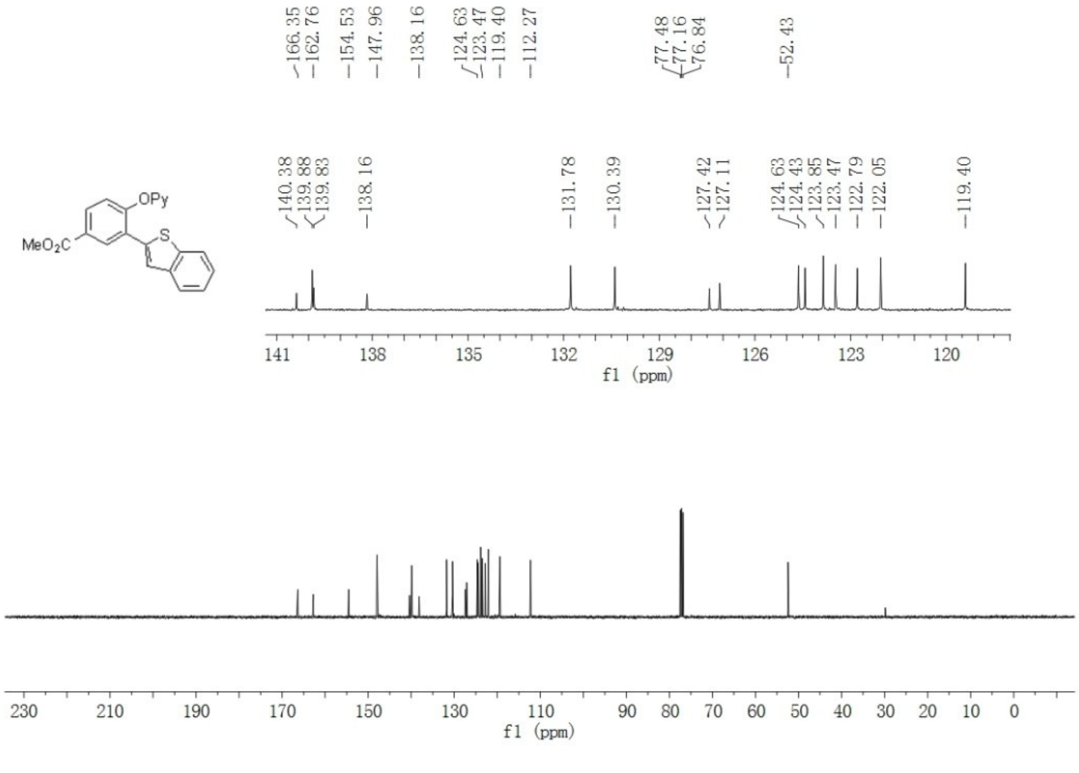

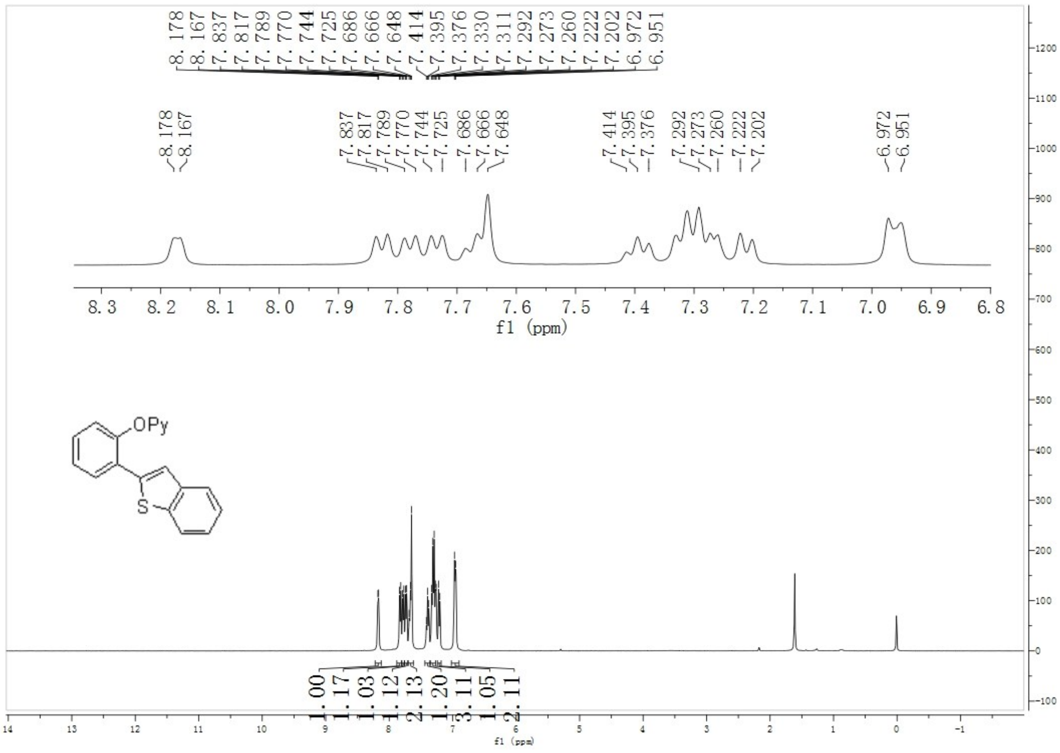

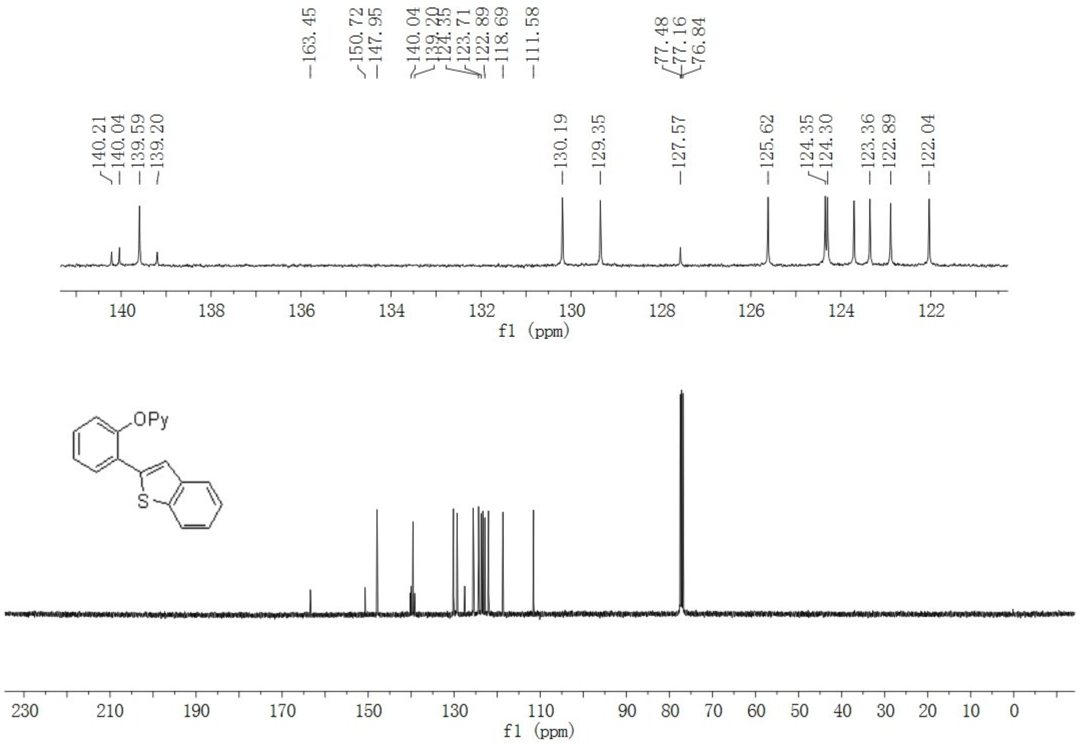

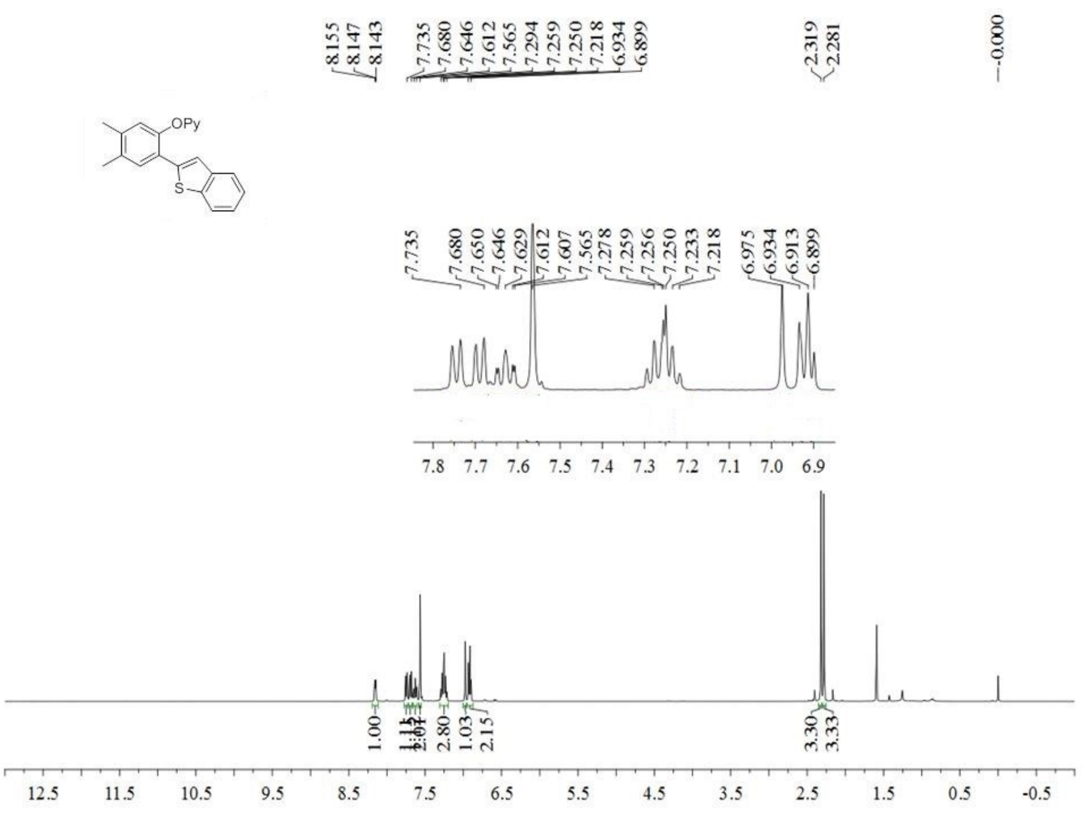
8.155
8.147
8.143
7.735
7.680
7.646
7.612
7.565
7.294
7.259
7.250
7.218
6.934
6.899
2.319
2.281
-0.000
OPy
S
7.735
7.680
7.650
7.646
7.629
7.612
7.607
7.565
7.278
7.259
7.256
7.250
7.233
7.218
6.975
6.934
6.913
6.899
7.8 7.7 7.6 7.5 7.4 7.3 7.2 7.1 7.0 6.9
1.00
2.80
1.03
2.15
3.30
3.33
12.5 11.5 10.5 9.5 8.5 7.5 6.5 5.5 4.5 3.5 2.5 1.5 0.5 -0.5

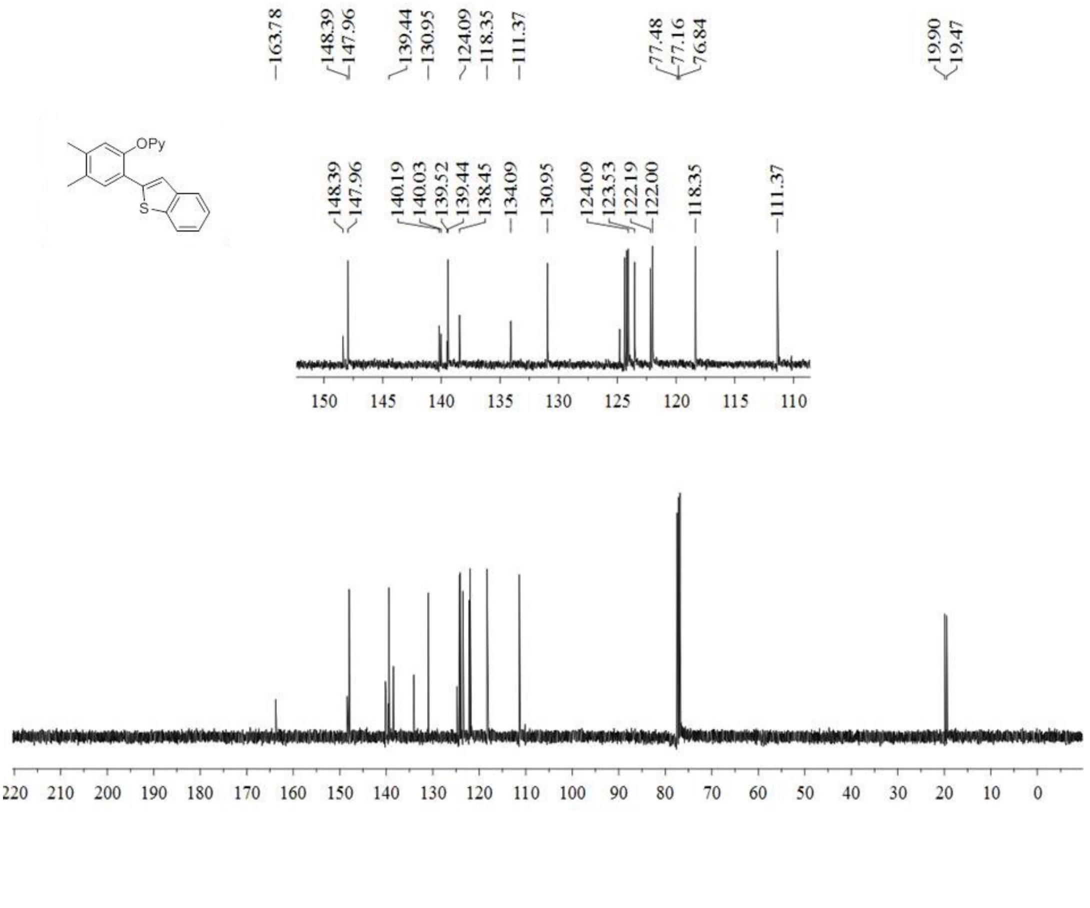
163.78
148.39
147.96
139.44
130.95
124.09
118.35
111.37
77.48
77.16
76.84
19.90
19.47
OPy
S
148.39
147.96
140.19
140.03
139.52
139.44
138.45
134.09
130.95
124.09
123.53
122.19
122.00
118.35
111.37
150 145 140 135 130 125 120 115 110
220 210 200 190 180 170 160 150 140 130 120 110 100 90 80 70 60 50 40 30 20 10 0

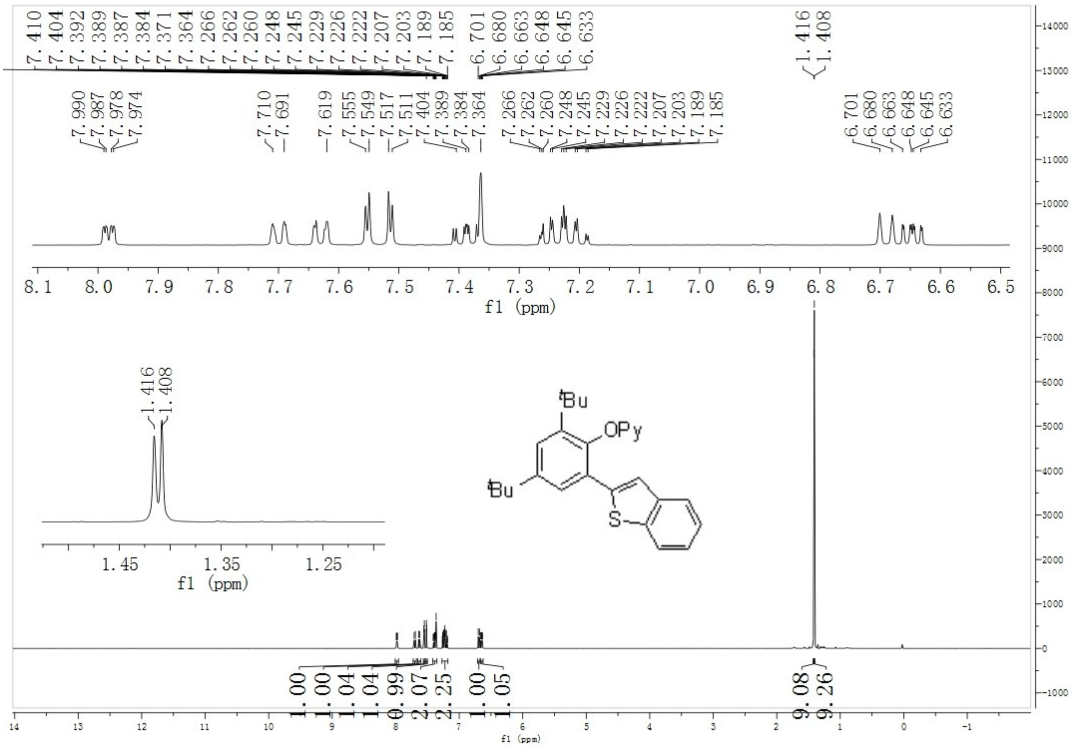
7.410 7.404 7.392 7.389 7.387 7.384 7.371 7.364 7.266 7.262 7.260 7.248 7.245 7.229 7.226 7.222 7.207 7.203 7.189 7.185 6.701 6.680 6.663 6.648 6.645 6.633
1.416 1.408
7.990 7.987 7.978 7.974 7.710 7.691 7.619 7.555 7.549 7.517 7.511 7.404 7.389 7.384 7.364 7.266 7.262 7.260 7.248 7.245 7.229 7.226 7.222 7.207 7.203 7.189 7.185
6.701 6.680 6.663 6.648 6.645 6.633
8.1 8.0 7.9 7.8 7.7 7.6 7.5 7.4 7.3 7.2 7.1 7.0 6.9 6.8 6.7 6.6 6.5
f1 (ppm)
1.416 1.408
1.45 1.35 1.25
f1 (ppm)
tBu
OPy
tBu
S
1.00 1.00 1.04 1.04 0.99 2.07 2.25 1.00 1.05
9.08 9.26
f1 (ppm)

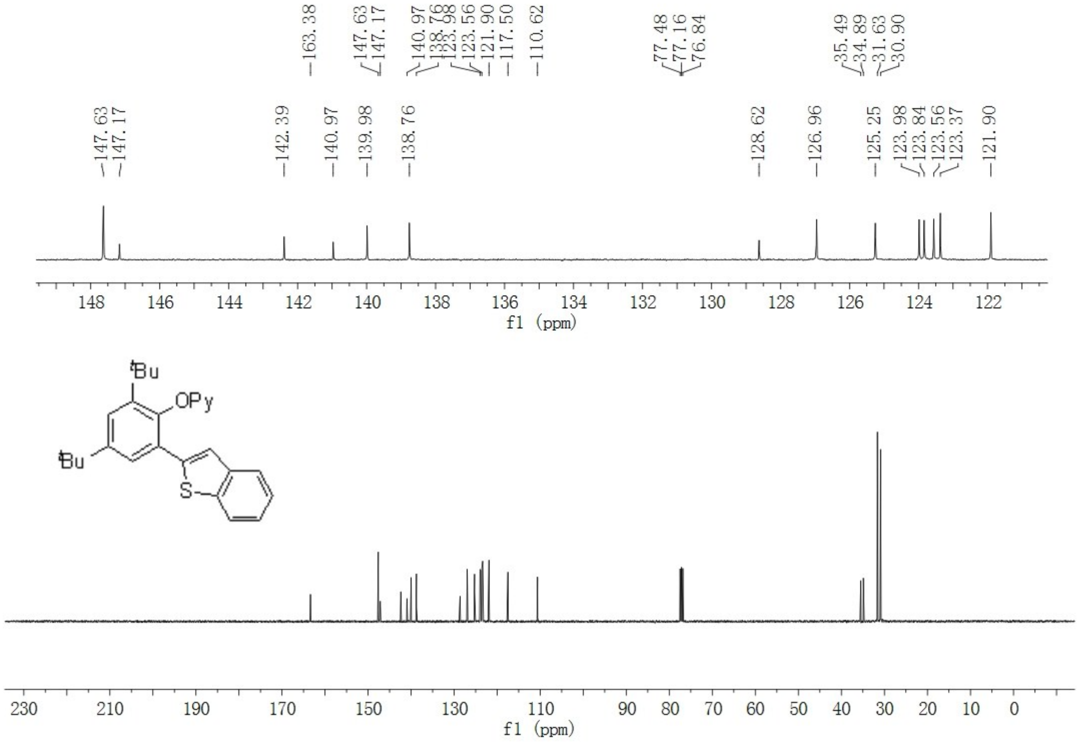
163.38 147.63 147.17 140.97 138.76 123.98 123.56 121.90 117.50 110.62
77.48 77.16 76.84
35.49 34.89 31.63 30.90
147.63 147.17 142.39 140.97 139.98 138.76 128.62 126.96 125.25 123.98 123.84 123.56 123.37 121.90
148 146 144 142 140 138 136 134 132 130 128 126 124 122
f1 (ppm)
tBu
OPy
tBu
S
230 210 190 170 150 130 110 90 80 70 60 50 40 30 20 10 0
f1 (ppm)

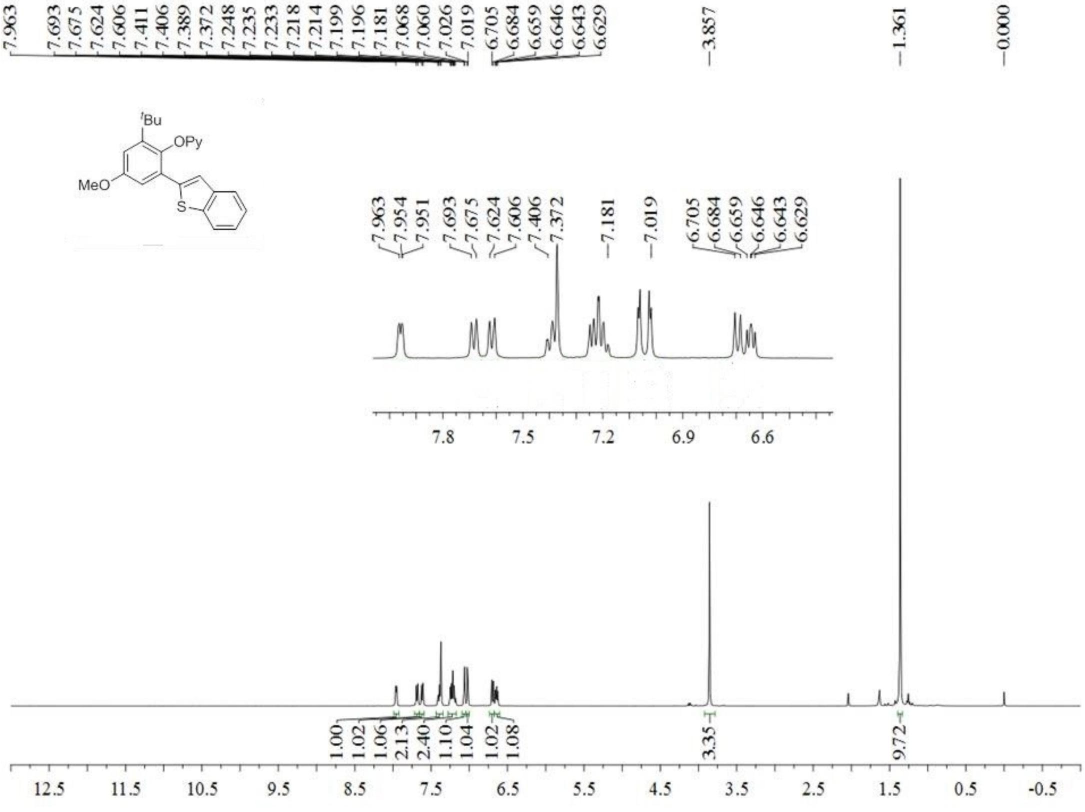
7.963
7.693
7.675
7.624
7.606
7.411
7.406
7.389
7.372
7.248
7.235
7.233
7.218
7.214
7.199
7.196
7.181
7.068
7.060
7.026
7.019
6.705
6.684
6.659
6.646
6.643
6.629
3.857
1.361
0.000
tBu
OPy
MeO
S
7.963
7.954
7.951
7.693
7.675
7.624
7.606
7.406
7.372
7.181
7.019
6.705
6.684
6.659
6.646
6.643
6.629
7.8
7.5
7.2
6.9
6.6
1.00
1.02
1.06
2.13
2.40
1.10
1.04
1.02
1.08
3.35
9.72
12.5
11.5
10.5
9.5
8.5
7.5
6.5
5.5
4.5
3.5
2.5
1.5
0.5
-0.5

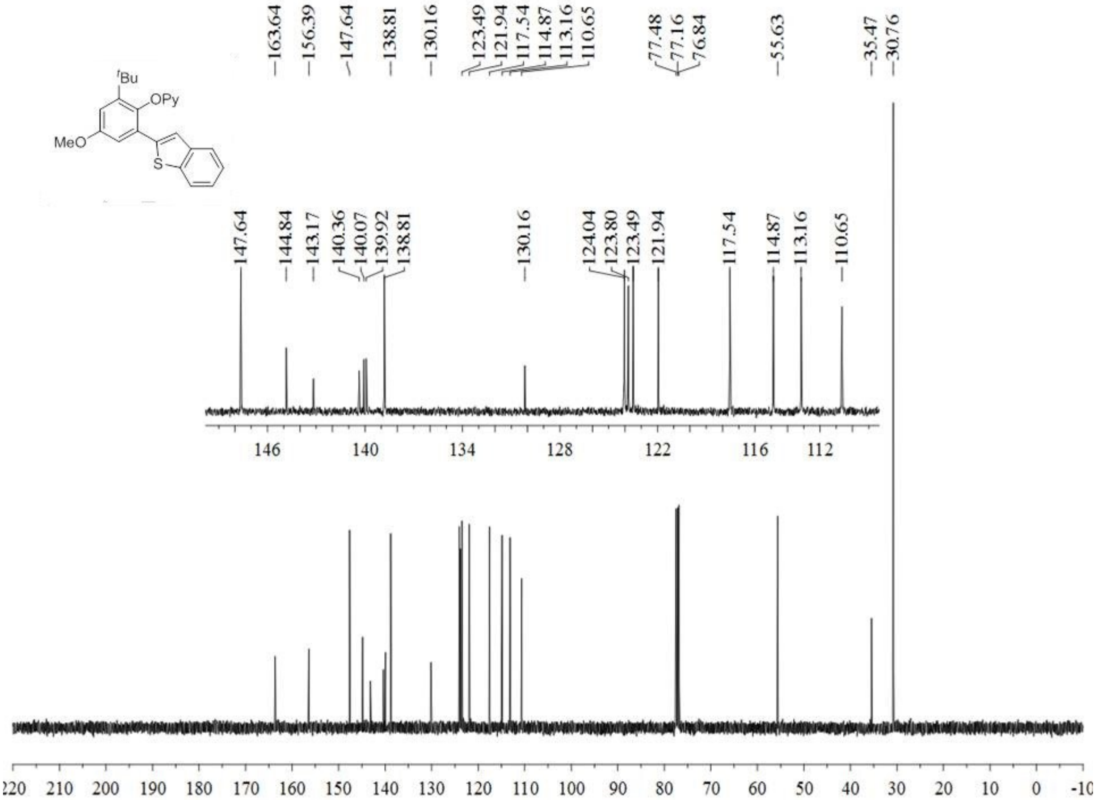
163.64
156.39
147.64
138.81
130.16
123.49
121.94
117.54
114.87
113.16
110.65
77.48
77.16
76.84
55.63
35.47
30.76
tBu
OPy
MeO
S
147.64
144.84
143.17
140.36
140.07
139.92
138.81
130.16
124.04
123.80
123.49
121.94
117.54
114.87
113.16
110.65
146
140
134
128
122
116
112
220
210
200
190
180
170
160
150
140
130
120
110
100
90
80
70
60
50
40
30
20
10
0
-10

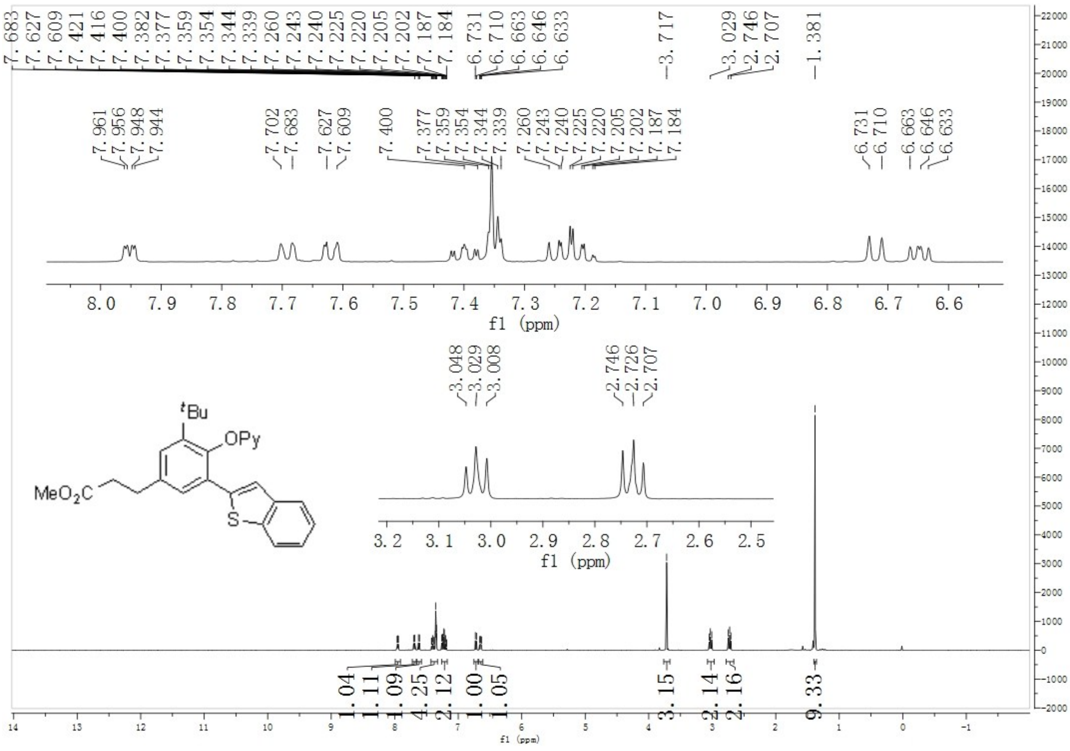
tBu
OPy
MeO_2C
S
f1 (ppm)
1.04
1.11
1.09
4.25
2.12
1.00
1.05
3.15
2.14
2.16
9.33

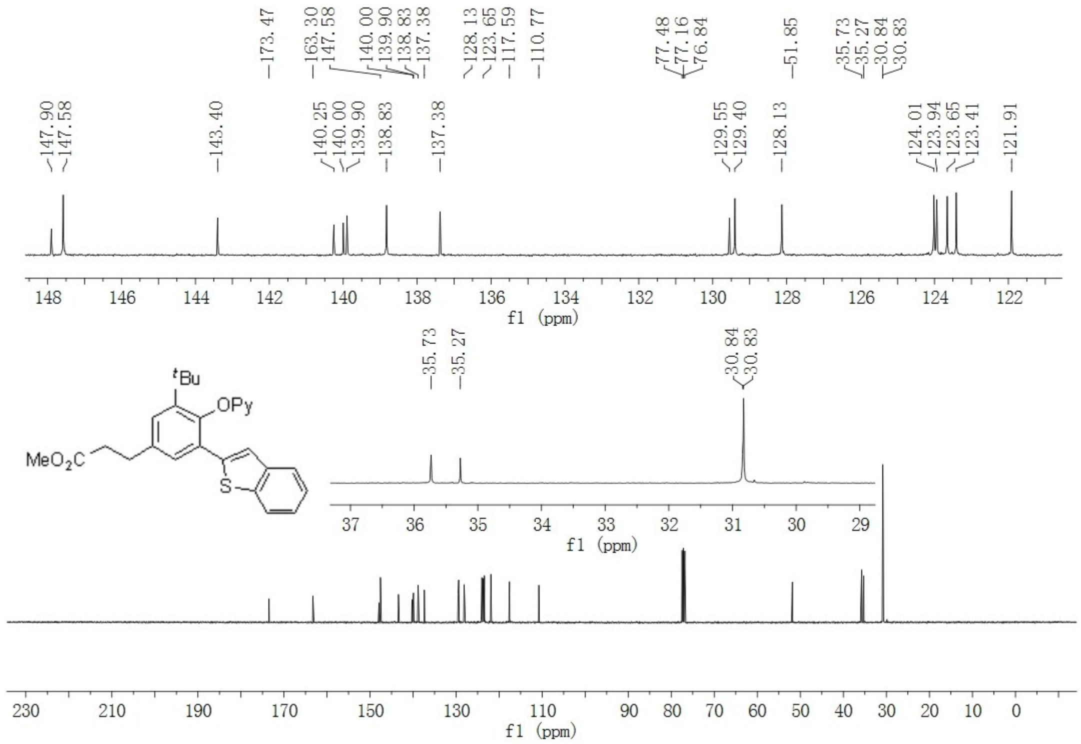
tBu
OPy
MeO_2C
S
f1 (ppm)

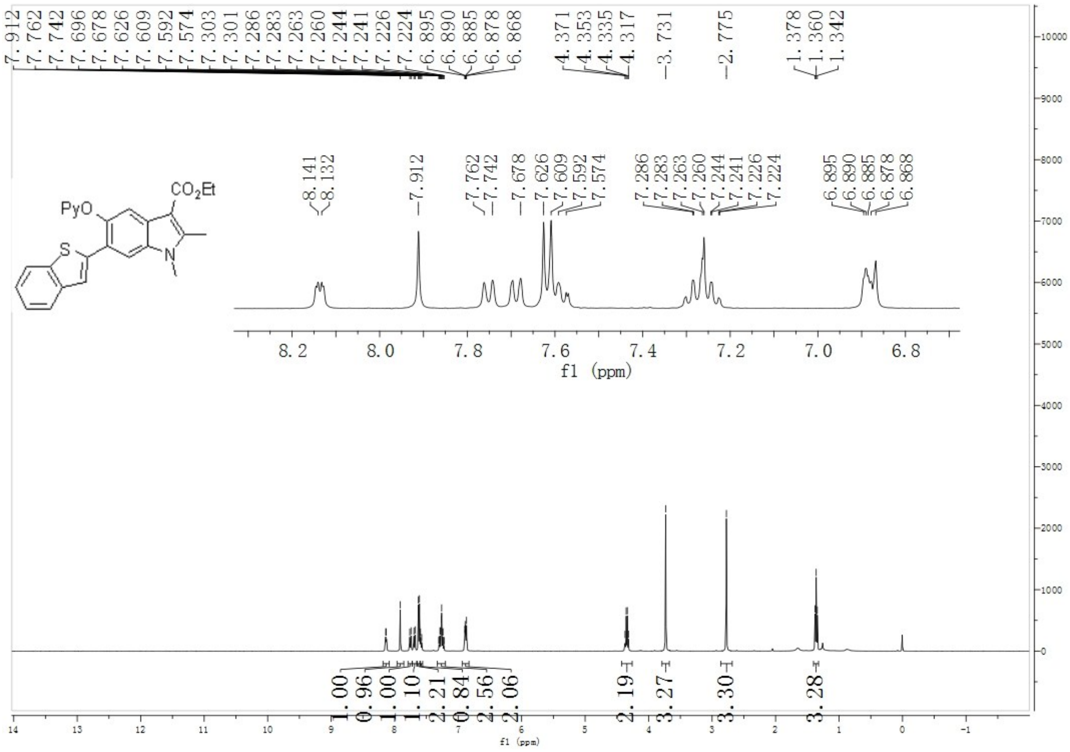
PyO
CO2Et
S
N
8.141
8.132
7.912
7.762
7.742
7.678
7.626
7.609
7.592
7.574
7.286
7.283
7.263
7.260
7.244
7.241
7.226
7.224
6.895
6.890
6.885
6.878
6.868
4.371
4.353
4.335
4.317
3.731
2.775
1.378
1.360
1.342
1.00
0.96
1.00
1.10
2.21
0.84
2.56
2.06
2.19
3.27
3.30
3.28
f1 (ppm)

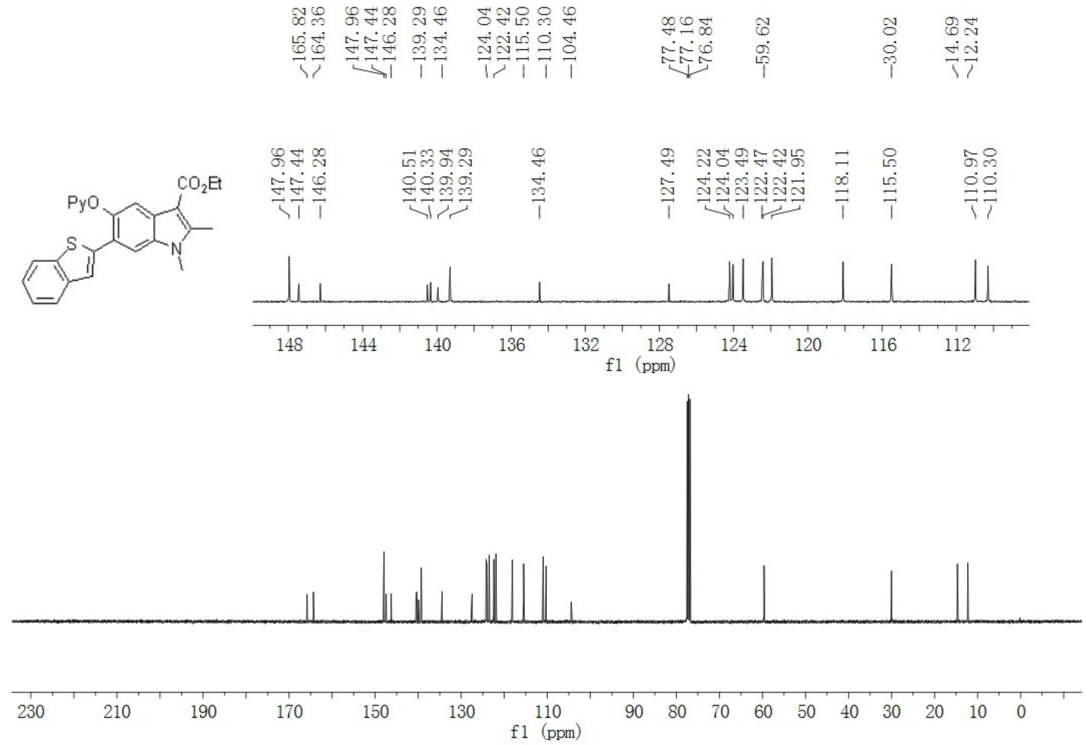
PyO
CO2Et
S
N
165.82
164.36
147.96
147.44
146.28
139.29
134.46
124.04
122.42
115.50
110.30
104.46
77.48
77.16
76.84
59.62
30.02
14.69
12.24
140.51
140.33
139.94
127.49
124.22
123.49
122.47
121.95
118.11
110.97
f1 (ppm)

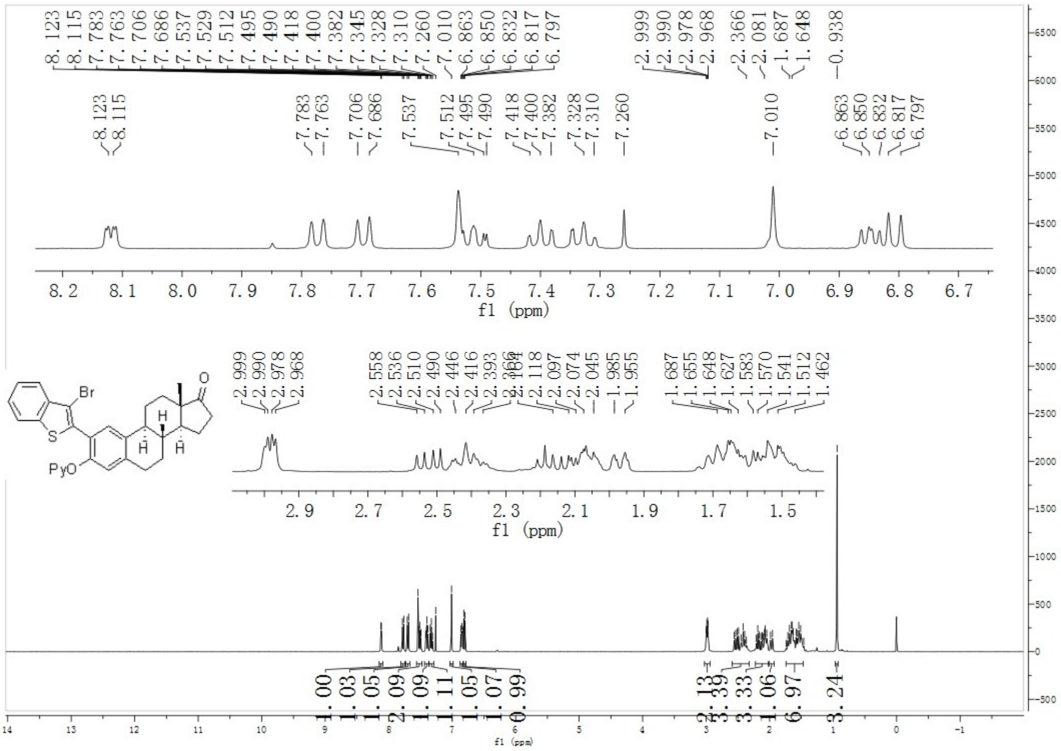

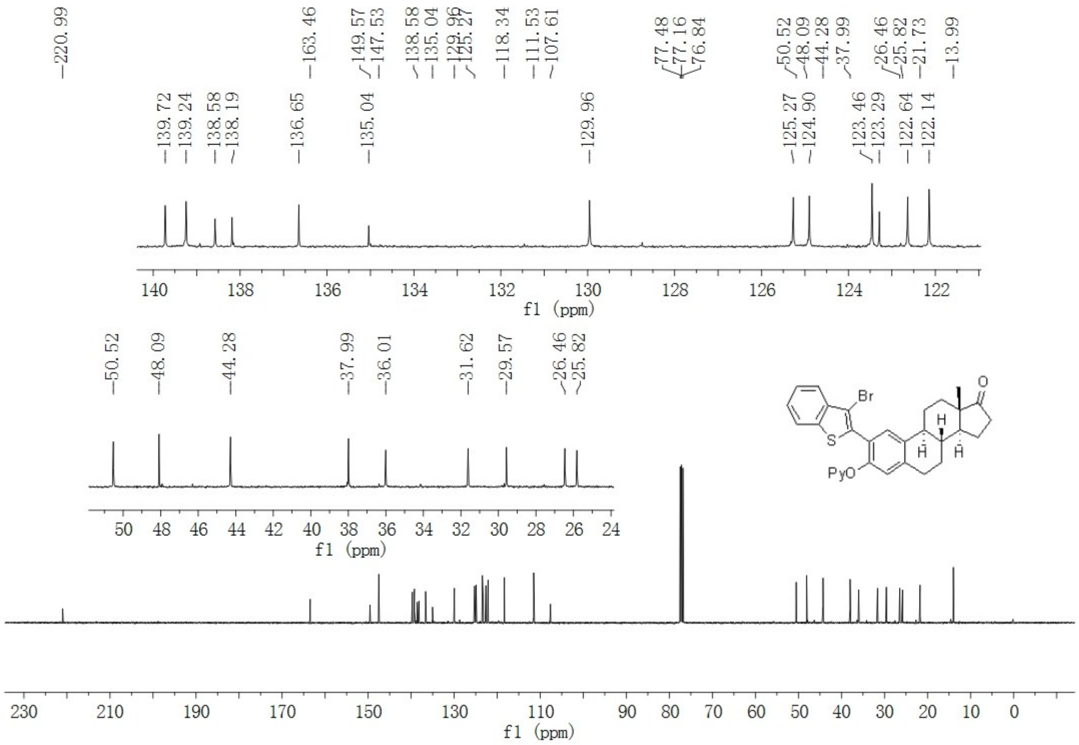

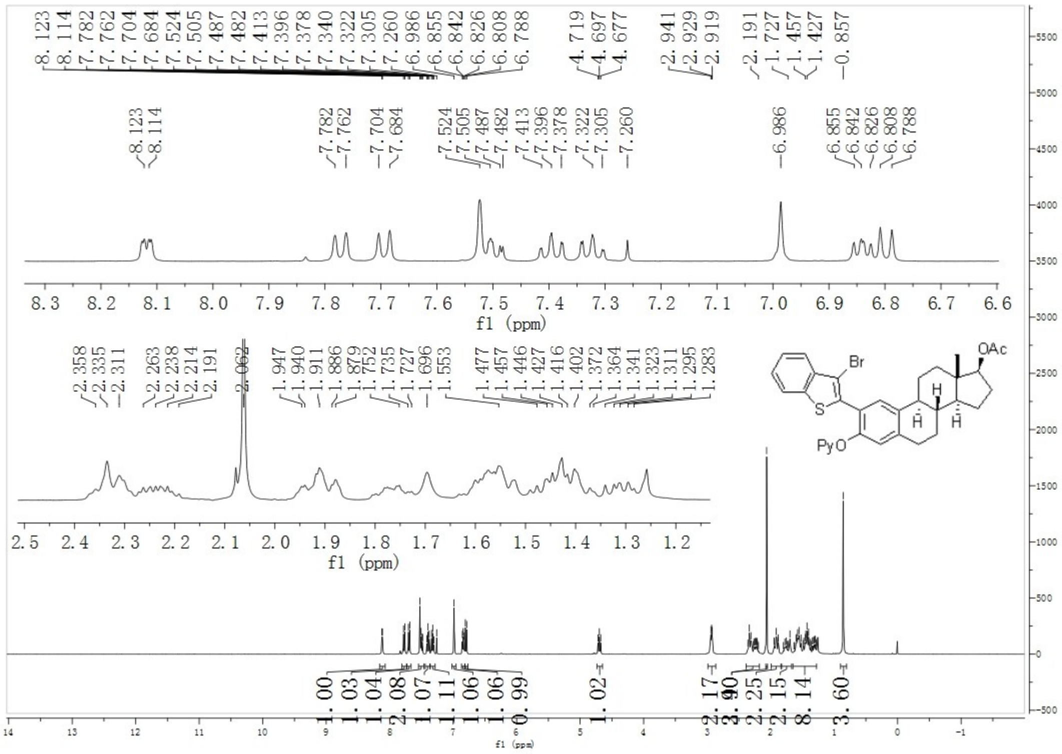

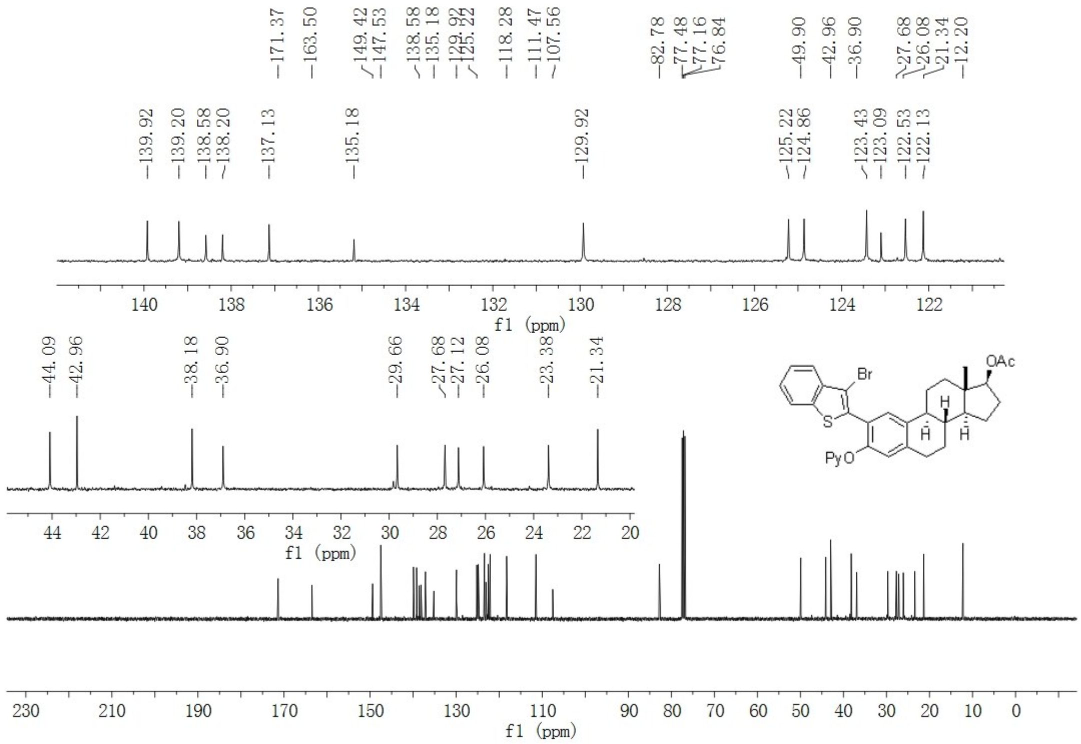

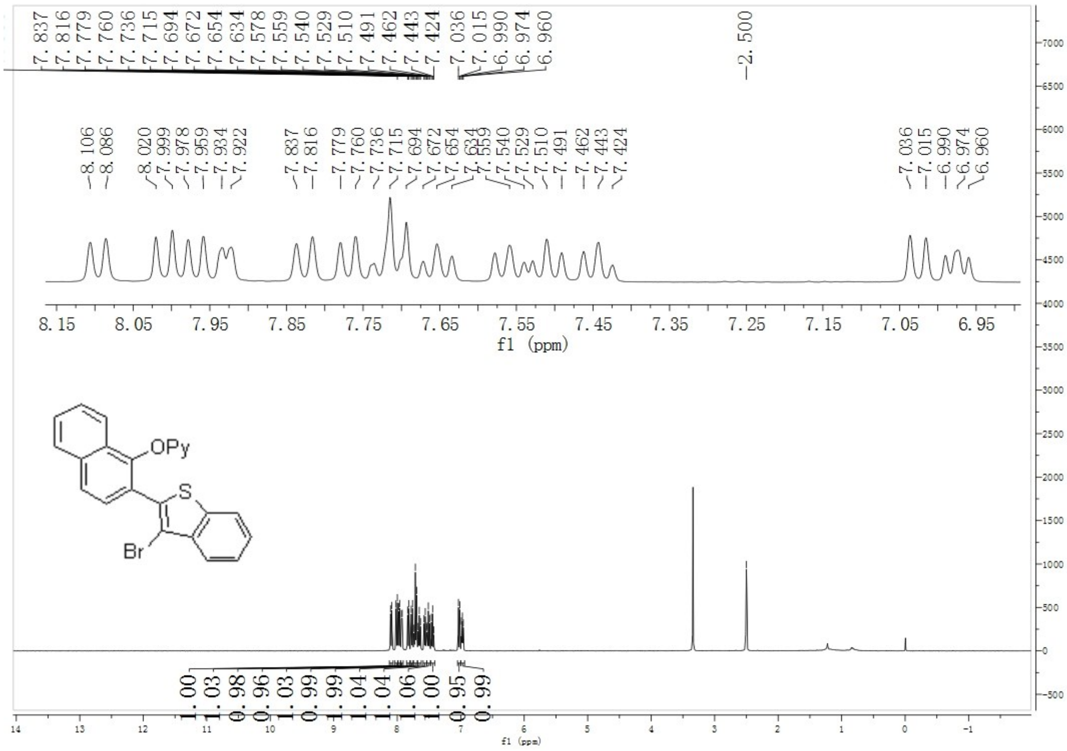
7.837 7.816 7.779 7.760 7.736 7.715 7.694 7.672 7.654 7.634 7.578 7.559 7.540 7.529 7.510 7.491 7.462 7.443 7.424 7.036 7.015 6.990 6.974 6.960
2.500
8.106 8.086 8.020 7.999 7.978 7.959 7.934 7.922 7.837 7.816 7.779 7.760 7.736 7.715 7.694 7.672 7.654 7.634 7.559 7.540 7.529 7.510 7.491 7.462 7.443 7.424
7.036 7.015 6.990 6.974 6.960
f1 (ppm)
OPy
S
Br
1.00 1.03 0.98 0.96 1.03 0.99 1.99 1.04 1.04 1.06 1.00 0.95 0.99
f1 (ppm)

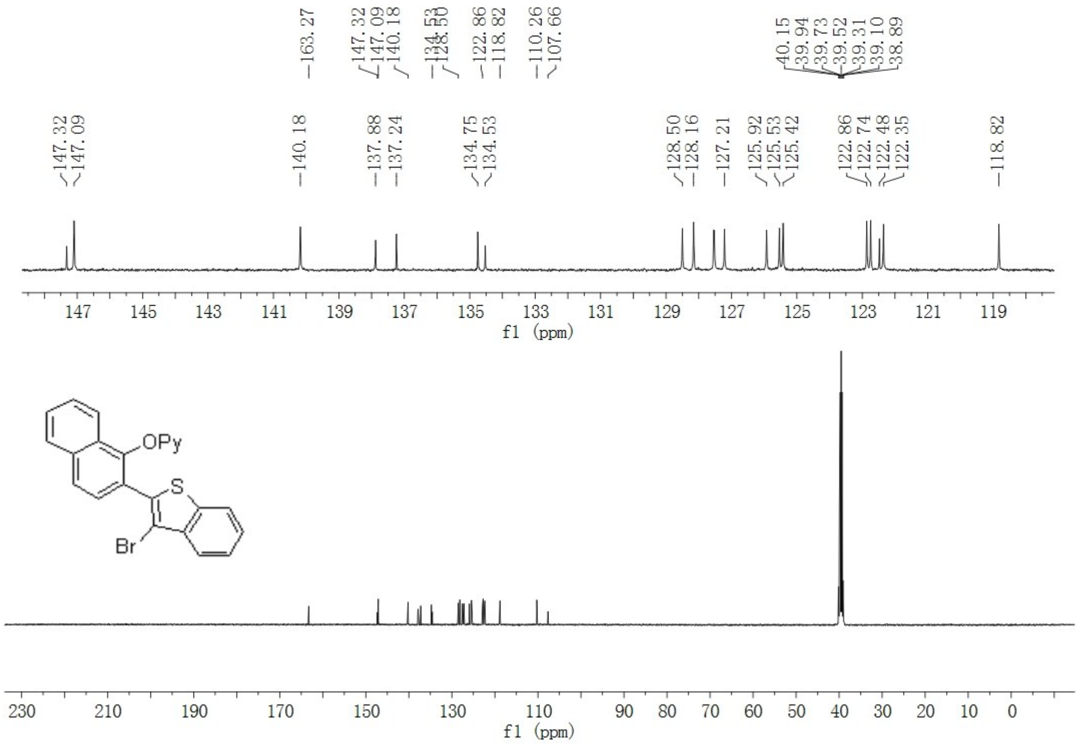
163.27
147.32 147.09 140.18
134.53 128.50
122.86 118.82
110.26 107.66
40.15 39.94 39.73 39.52 39.31 39.10 38.89
147.32 147.09
140.18
137.88 137.24
134.75 134.53
128.50 128.16
127.21
125.92 125.53 125.42
122.86 122.74 122.48 122.35
118.82
f1 (ppm)
OPy
S
Br
f1 (ppm)

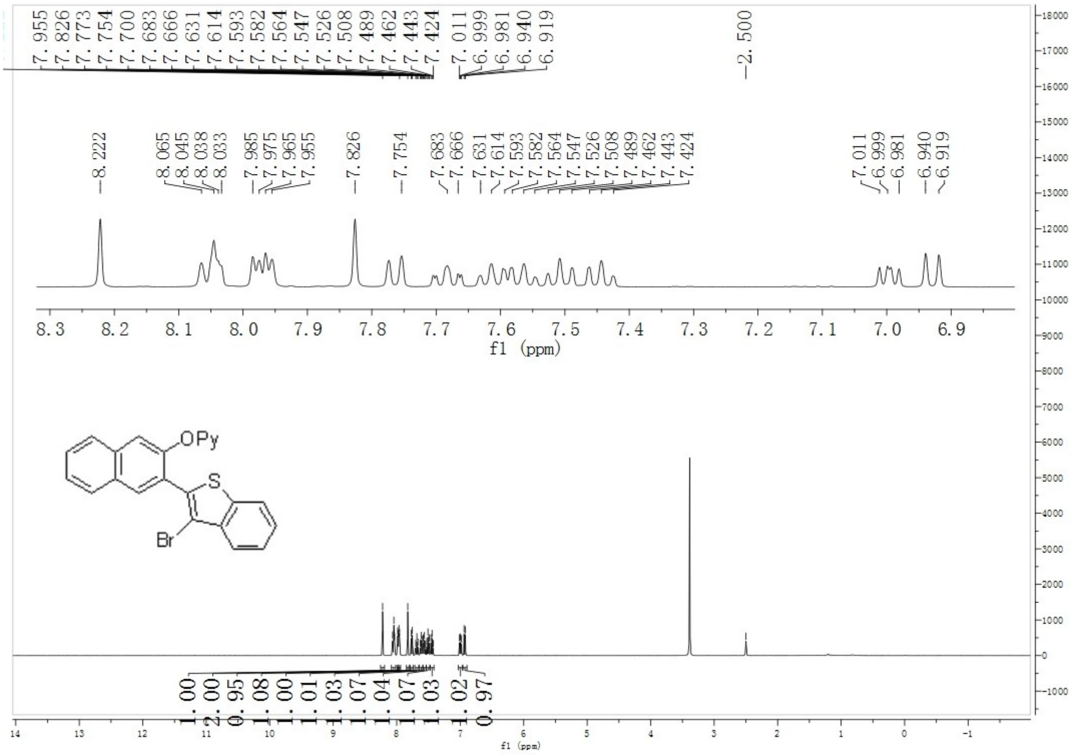
OPy
S
Br
f1 (ppm)

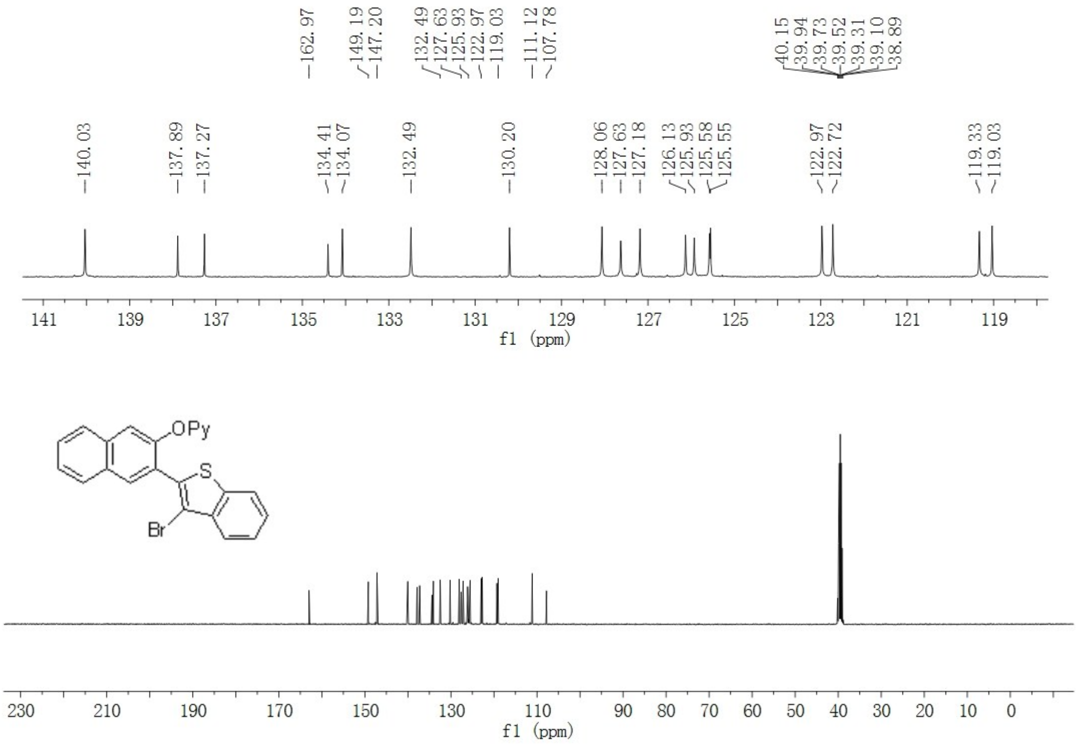
OPy
S
Br
f1 (ppm)

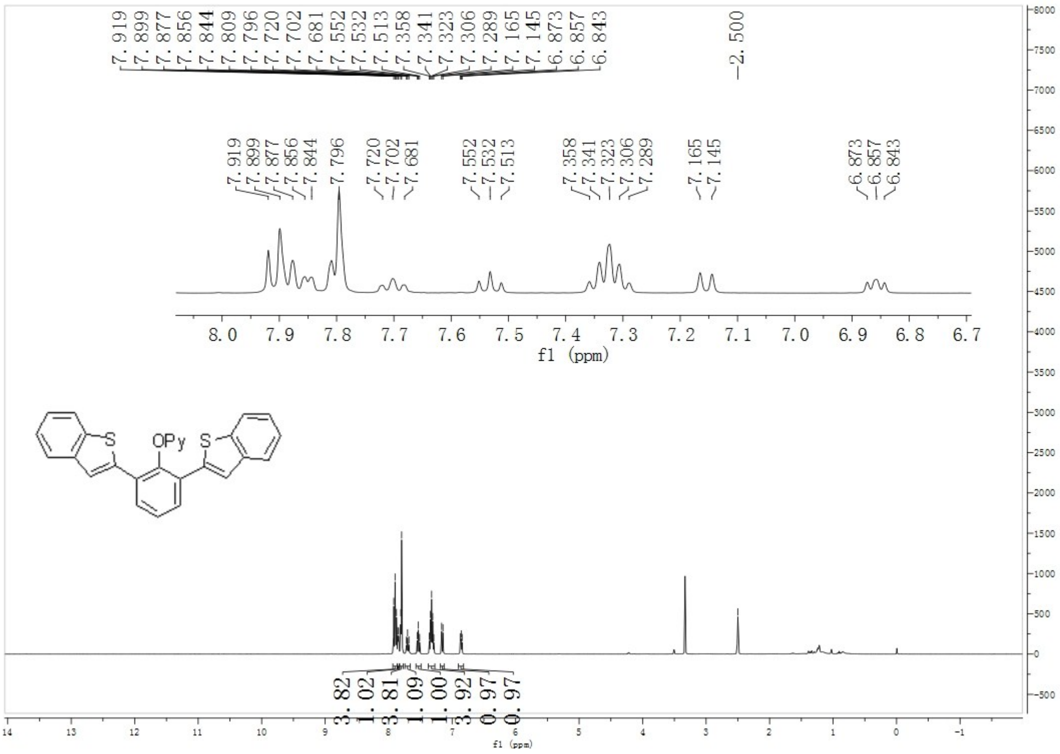
7.919
7.899
7.877
7.856
7.844
7.809
7.796
7.720
7.702
7.681
7.552
7.532
7.513
7.358
7.341
7.323
7.306
7.289
7.165
7.145
6.873
6.857
6.843
2.500
f1 (ppm)
OPy
S
S
3.82
1.02
3.81
1.09
1.00
3.92
0.97
0.97

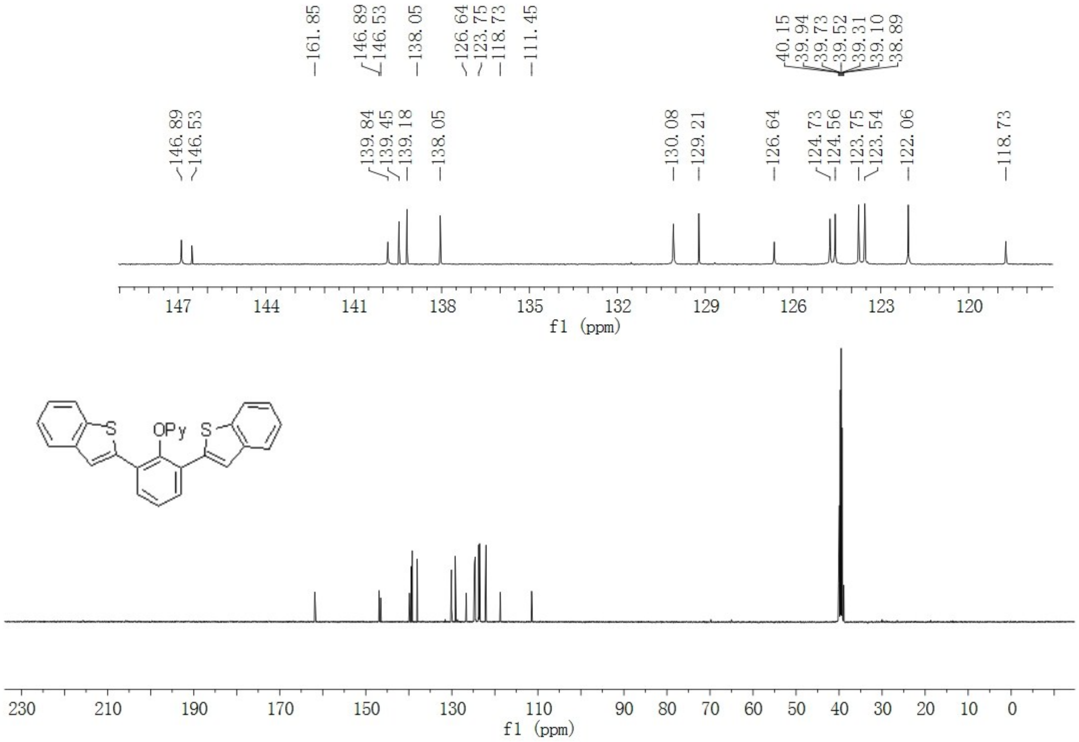
161.85
146.89
146.53
138.05
126.64
123.75
118.73
111.45
40.15
39.94
39.73
39.52
39.31
39.10
38.89
139.84
139.45
139.18
130.08
129.21
124.73
124.56
123.54
122.06
f1 (ppm)
OPy
S
S

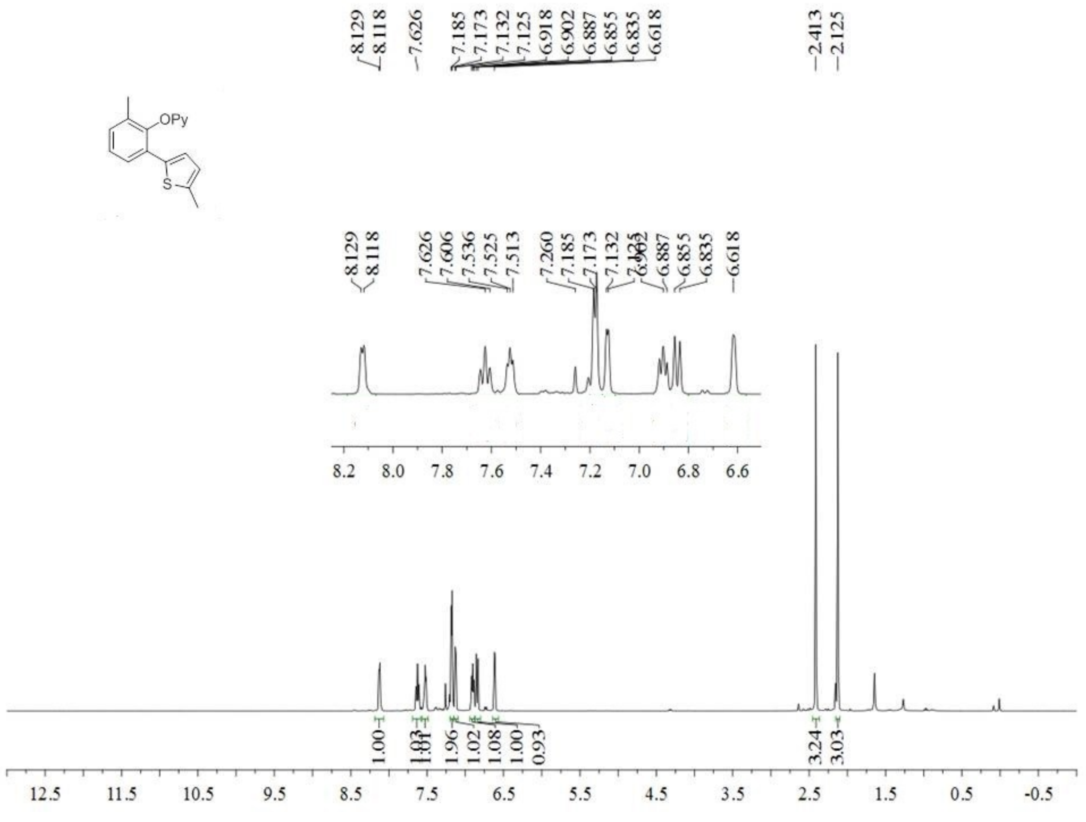
8.129
8.118
7.626
7.185
7.173
7.132
7.125
6.918
6.902
6.887
6.855
6.835
6.618
2.413
2.125
OPy
S
8.129
8.118
7.626
7.606
7.536
7.525
7.513
7.260
7.185
7.173
7.132
7.125
6.902
6.887
6.855
6.835
6.618
8.2 8.0 7.8 7.6 7.4 7.2 7.0 6.8 6.6
1.00
1.03
1.01
1.96
1.02
1.08
1.00
0.93
3.24
3.03
12.5 11.5 10.5 9.5 8.5 7.5 6.5 5.5 4.5 3.5 2.5 1.5 0.5 -0.5

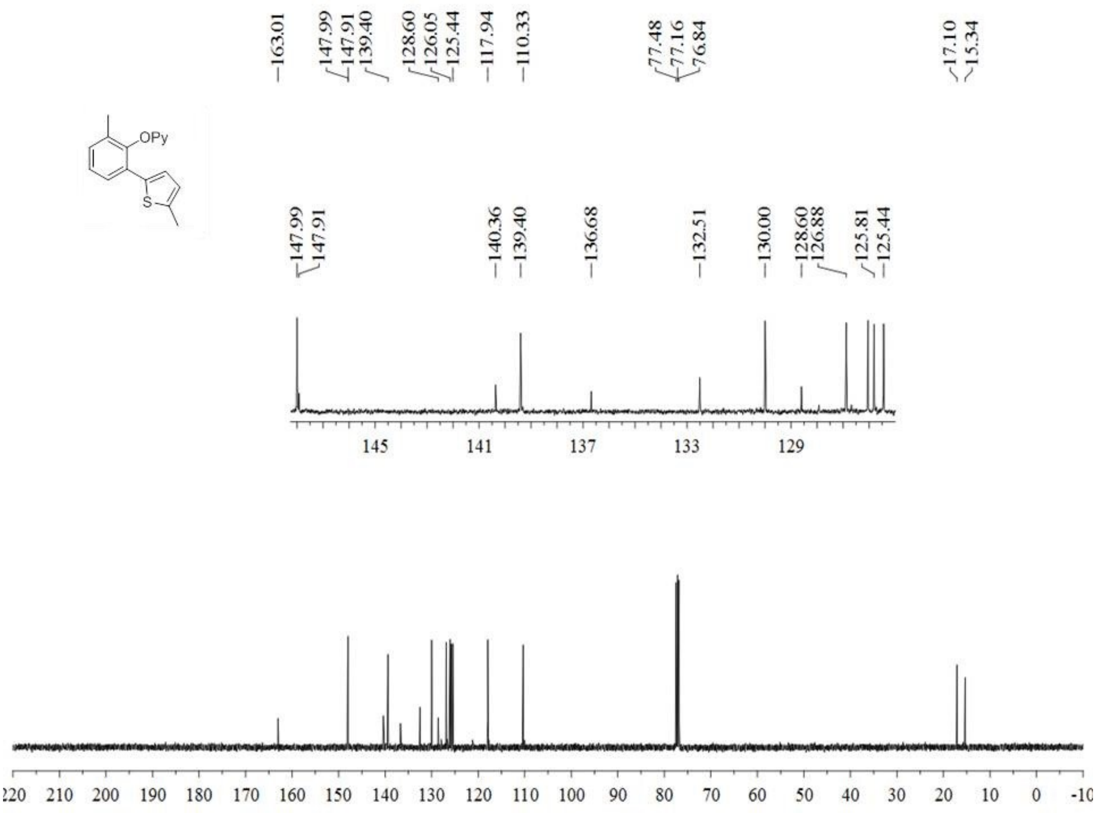
163.01
147.99
147.91
139.40
128.60
126.05
125.44
117.94
110.33
77.48
77.16
76.84
17.10
15.34
OPy
S
147.99
147.91
140.36
139.40
136.68
132.51
130.00
128.60
126.88
125.81
125.44
145 141 137 133 129
220 210 200 190 180 170 160 150 140 130 120 110 100 90 80 70 60 50 40 30 20 10 0 -10

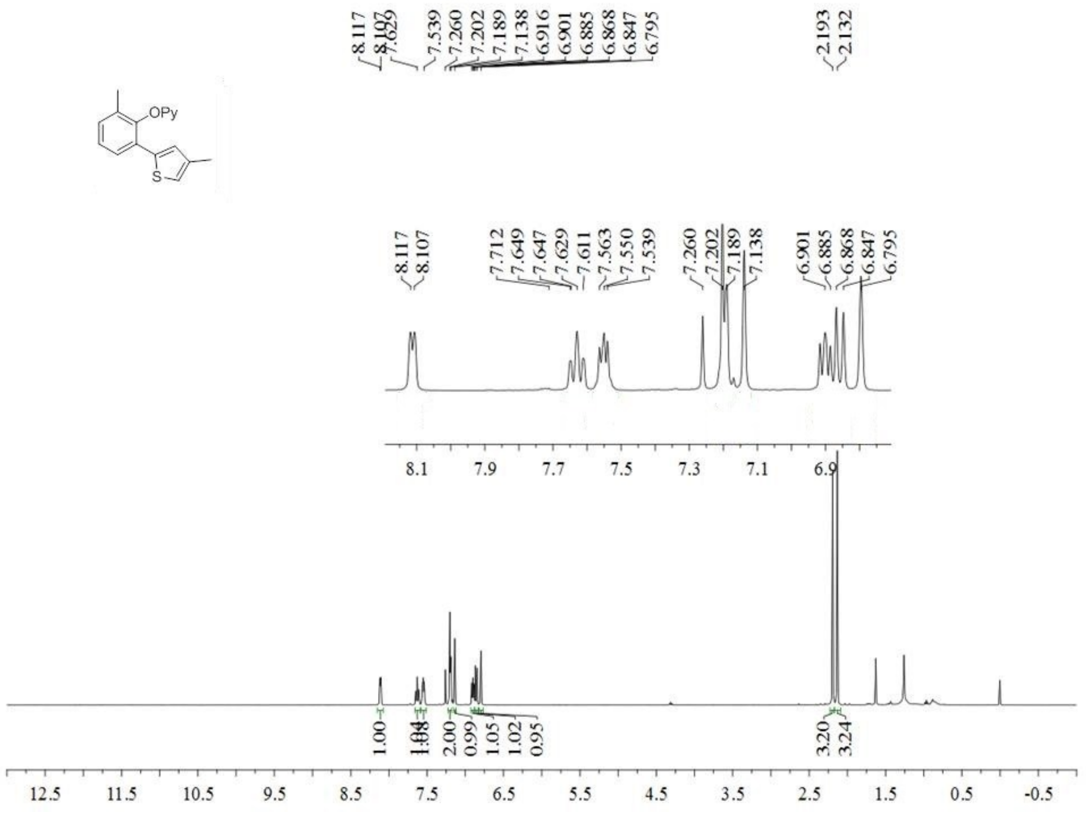
OPy
S
8.117
8.107
7.629
7.539
7.260
7.202
7.189
7.138
6.916
6.901
6.885
6.868
6.847
6.795
2.193
2.132
7.712
7.649
7.647
7.611
7.563
7.550
1.00
1.04
1.08
2.00
0.99
1.05
1.02
0.95
3.20
3.24

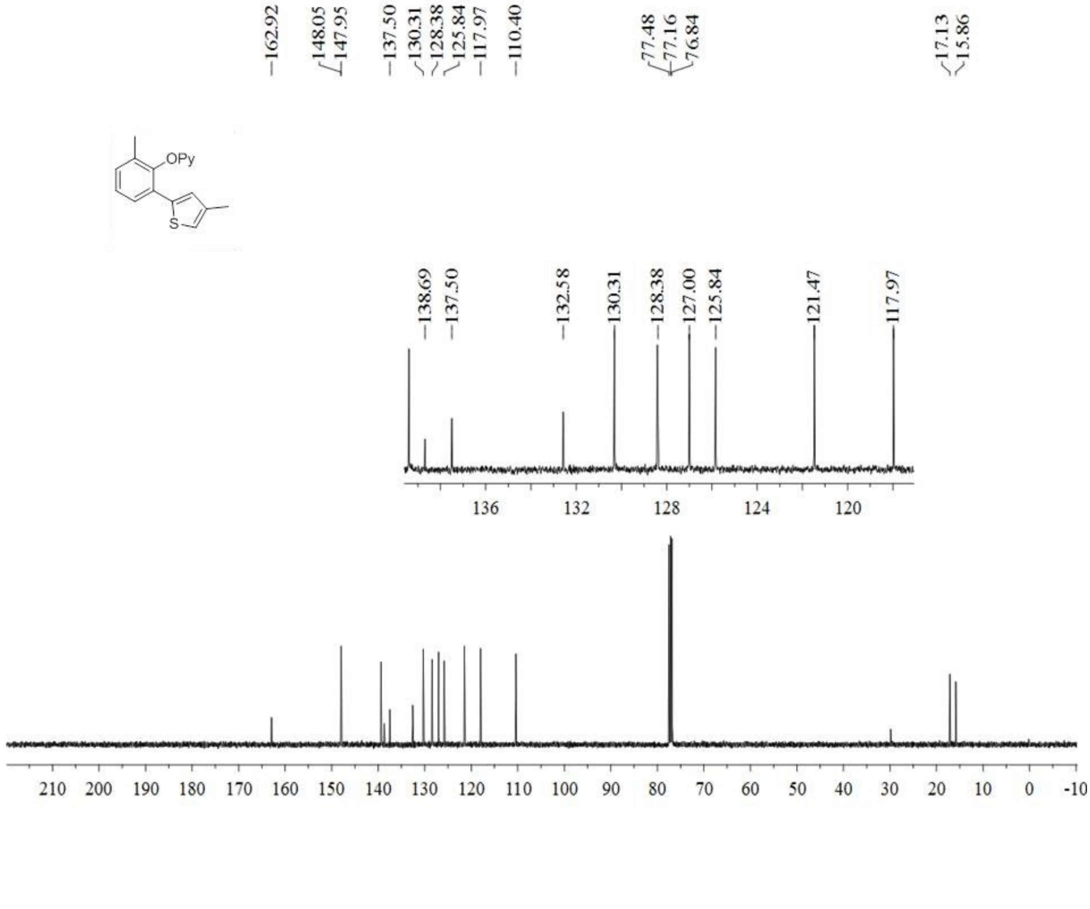
OPy
S
162.92
148.05
147.95
137.50
130.31
128.38
125.84
117.97
110.40
77.48
77.16
76.84
17.13
15.86
138.69
132.58
127.00
121.47

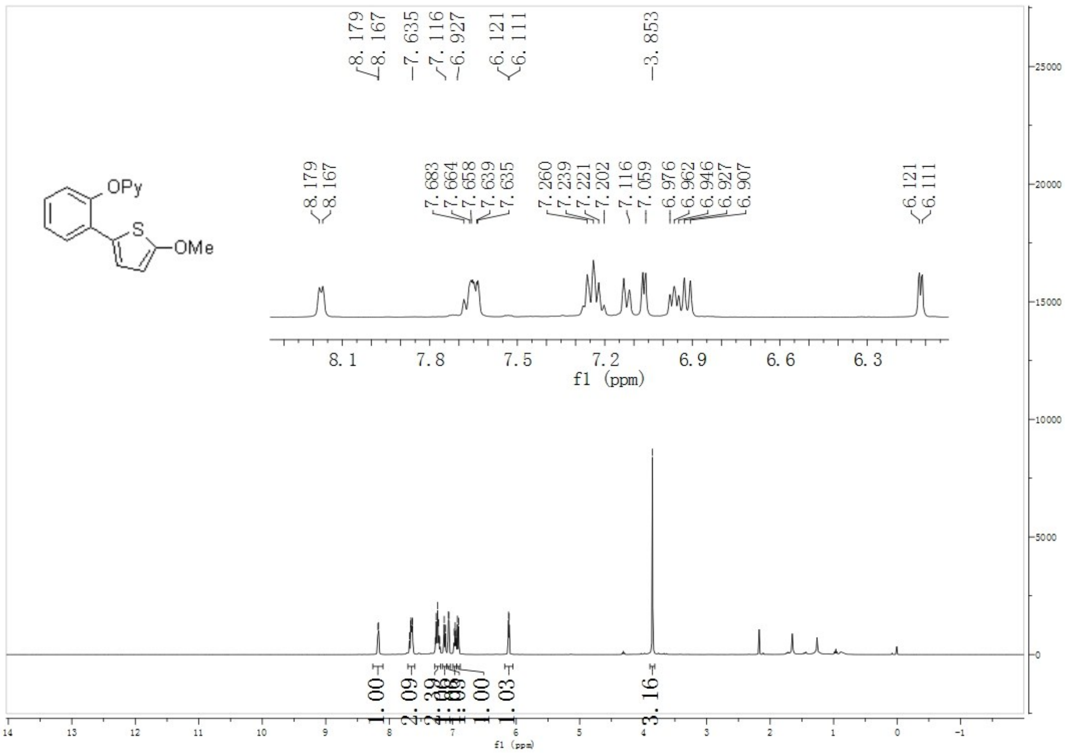
OPy
S
OMe
8.179
8.167
7.635
7.116
6.927
6.121
6.111
3.853
7.683
7.664
7.658
7.639
7.635
7.260
7.239
7.221
7.202
7.116
7.059
6.976
6.962
6.946
6.927
6.907
f1 (ppm)
1.00
2.09
1.00
1.03
3.16

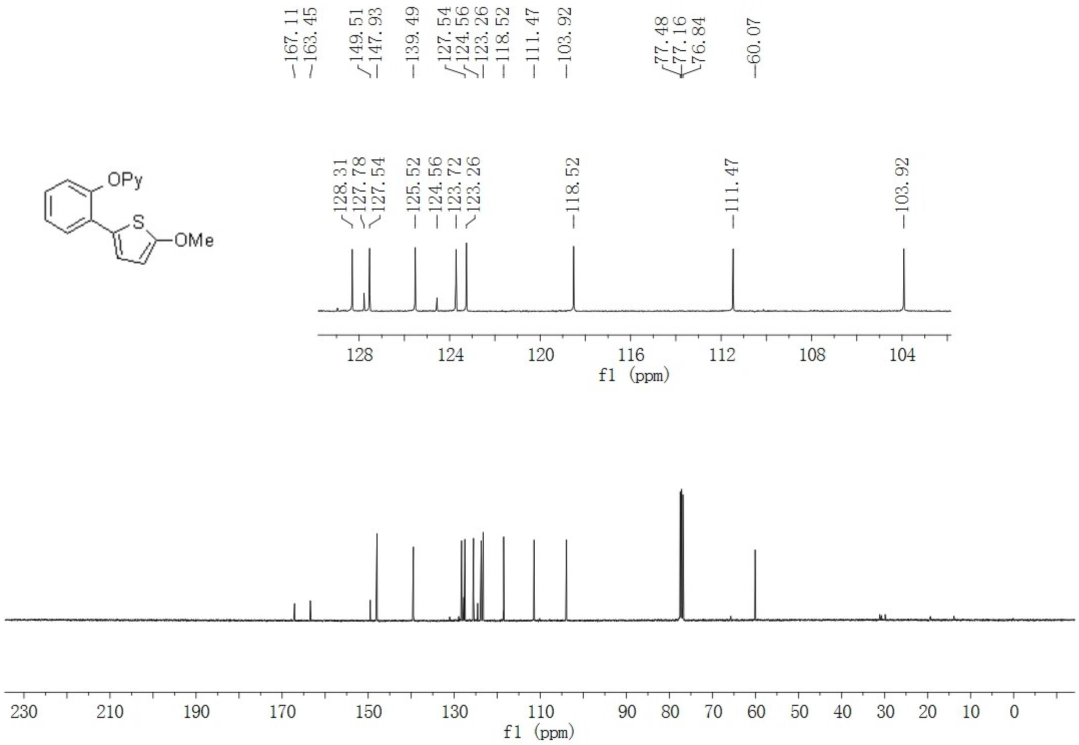
167.11
163.45
149.51
147.93
139.49
127.54
124.56
123.26
118.52
111.47
103.92
77.48
77.16
76.84
60.07
OPy
S
OMe
128.31
127.78
127.54
125.52
124.56
123.72
123.26
118.52
111.47
103.92
f1 (ppm)

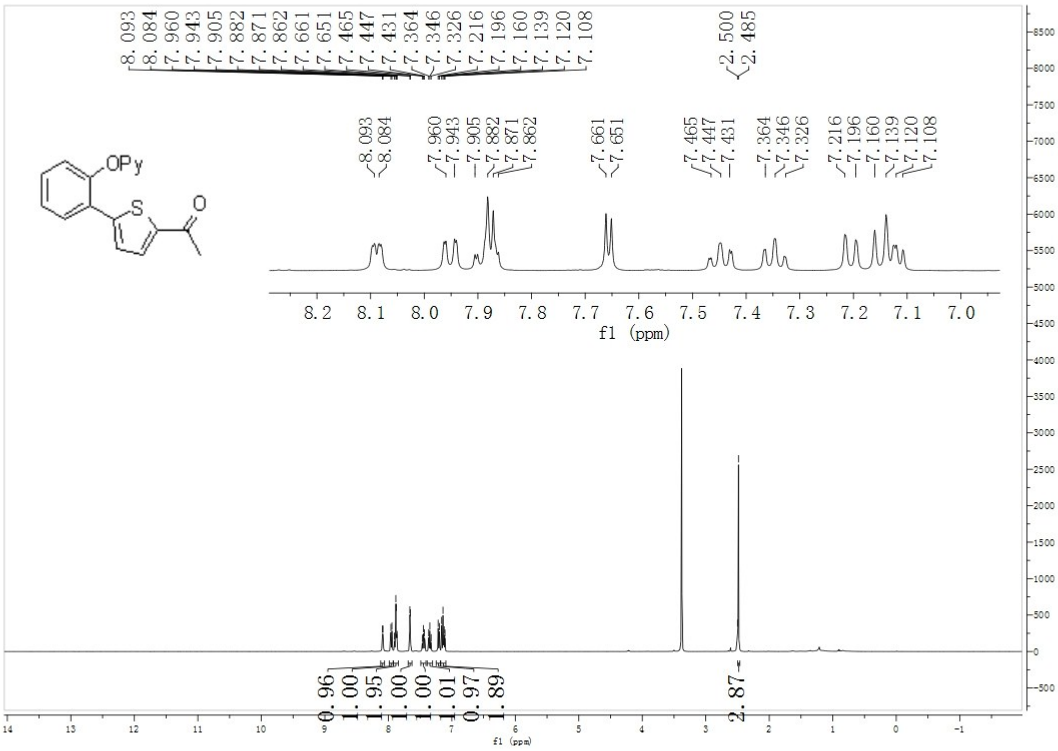
OPy
S
O
8.093 8.084 7.960 7.943 7.905 7.882 7.871 7.862 7.661 7.651 7.465 7.447 7.431 7.364 7.346 7.326 7.216 7.196 7.160 7.139 7.120 7.108
2.500 2.485
0.96 1.00 1.95 1.00 1.00 1.01 0.97 1.89
2.87
f1 (ppm)

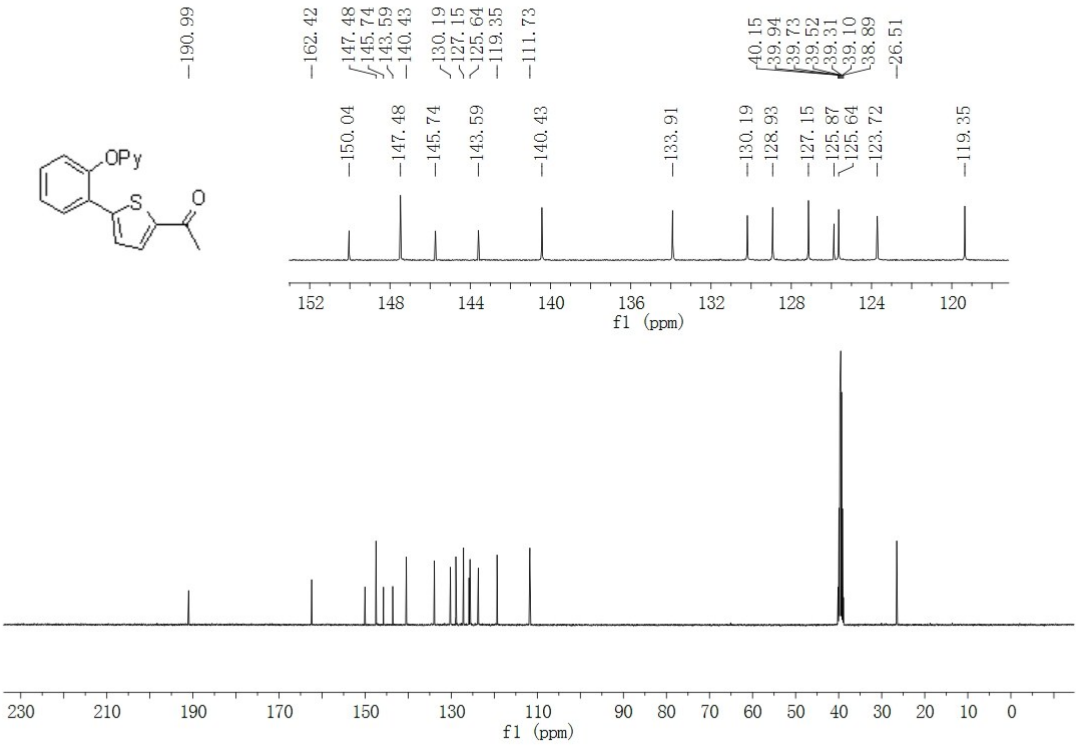
OPy
S
O
190.99
162.42 147.48 145.74 143.59 140.43 130.19 127.15 125.64 119.35 111.73
40.15 39.94 39.73 39.52 39.31 39.10 38.89 26.51
150.04 147.48 145.74 143.59 140.43 133.91 130.19 128.93 127.15 125.87 125.64 123.72 119.35
f1 (ppm)

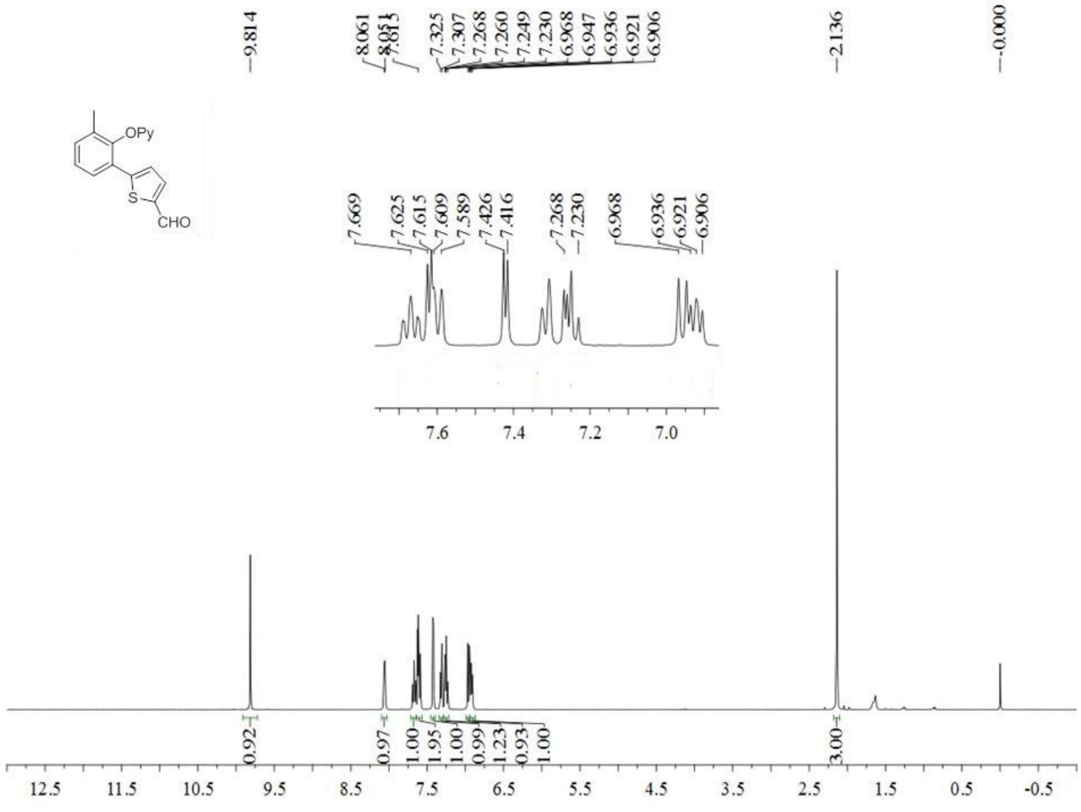
9.814
8.061
8.051
7.615
7.325
7.307
7.268
7.260
7.249
7.230
6.968
6.947
6.936
6.921
6.906
2.136
0.000
OPy
S
CHO
7.669
7.625
7.615
7.609
7.589
7.426
7.416
7.268
7.230
6.968
6.936
6.921
6.906
7.6 7.4 7.2 7.0
0.92
0.97
1.00
1.95
1.00
0.99
1.23
0.93
1.00
3.00
12.5 11.5 10.5 9.5 8.5 7.5 6.5 5.5 4.5 3.5 2.5 1.5 0.5 -0.5

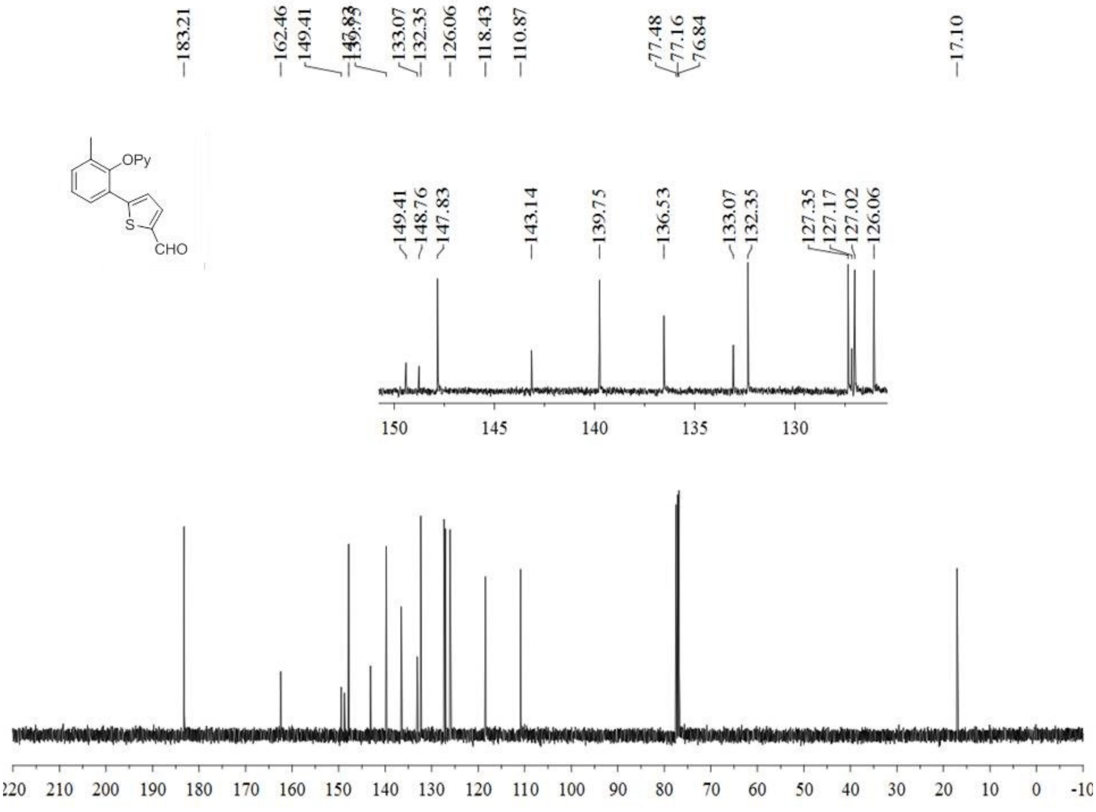
183.21
162.46
149.41
147.83
139.75
133.07
132.35
126.06
118.43
110.87
77.48
77.16
76.84
17.10
OPy
S
CHO
149.41
148.76
147.83
143.14
139.75
136.53
133.07
132.35
127.35
127.17
127.02
126.06
150 145 140 135 130
220 210 200 190 180 170 160 150 140 130 120 110 100 90 80 70 60 50 40 30 20 10 0 -10

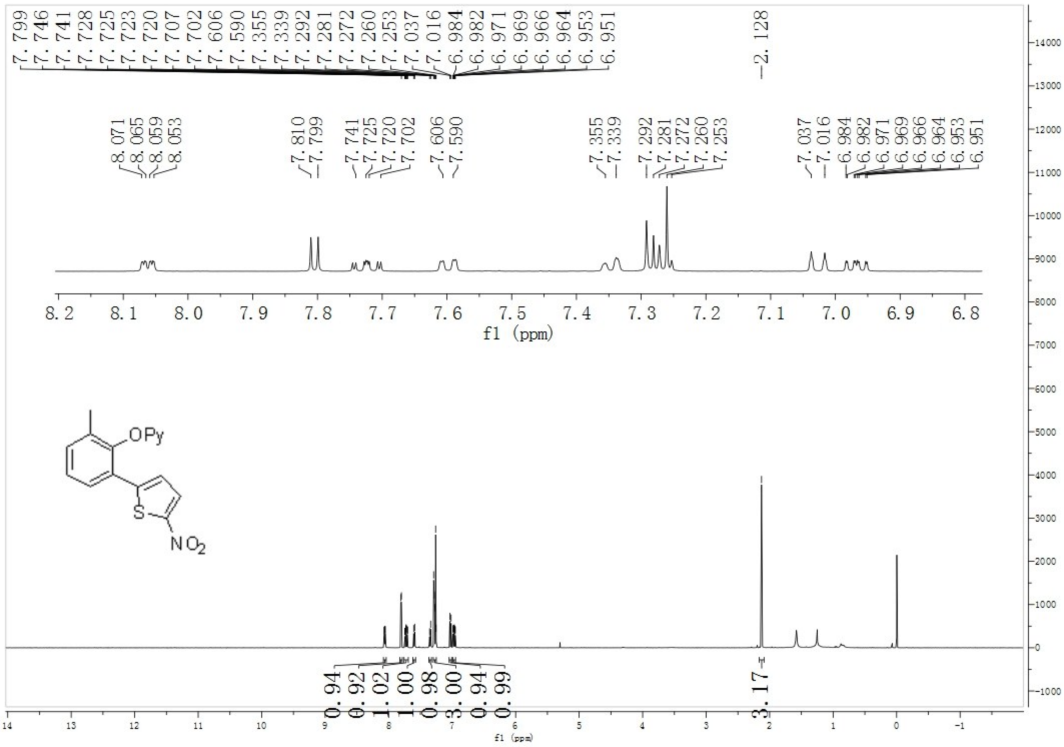

OPy
S
NO2
f1 (ppm)
-2.128

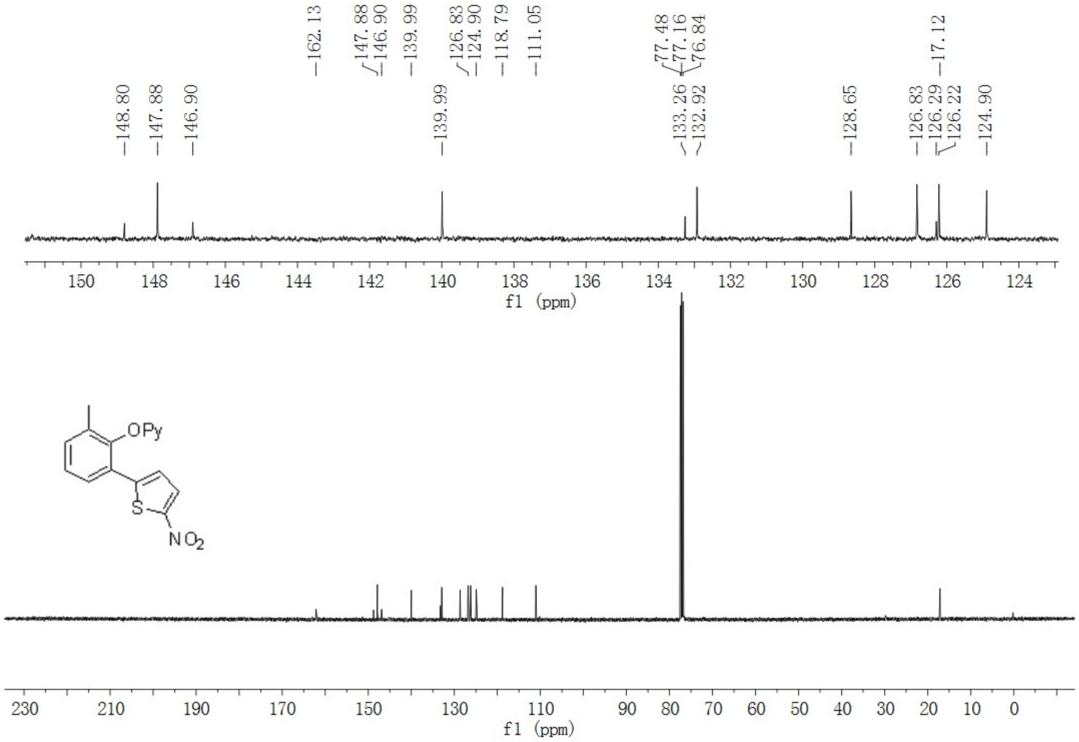

OPy
S
NO2
f1 (ppm)
162.13
147.88
146.90
139.99
126.83
124.90
118.79
111.05
77.48
77.16
76.84
17.12

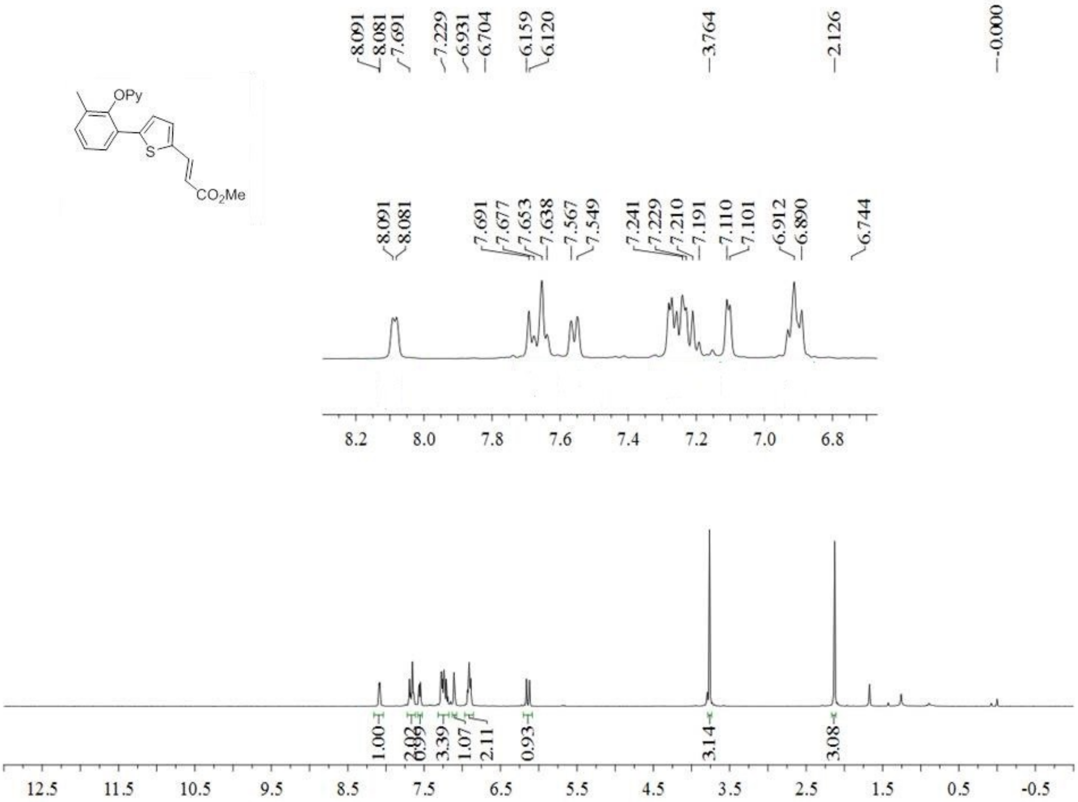
OPy
S
CO2Me
8.091
8.081
7.691
7.229
6.931
6.704
6.159
6.120
3.764
2.126
-0.000
8.091
8.081
7.691
7.677
7.653
7.638
7.567
7.549
7.241
7.229
7.210
7.191
7.110
7.101
6.912
6.890
6.744
8.2
8.0
7.8
7.6
7.4
7.2
7.0
6.8
1.00
2.02
0.99
3.39
1.07
2.11
0.93
3.14
3.08
12.5
11.5
10.5
9.5
8.5
7.5
6.5
5.5
4.5
3.5
2.5
1.5
0.5
-0.5

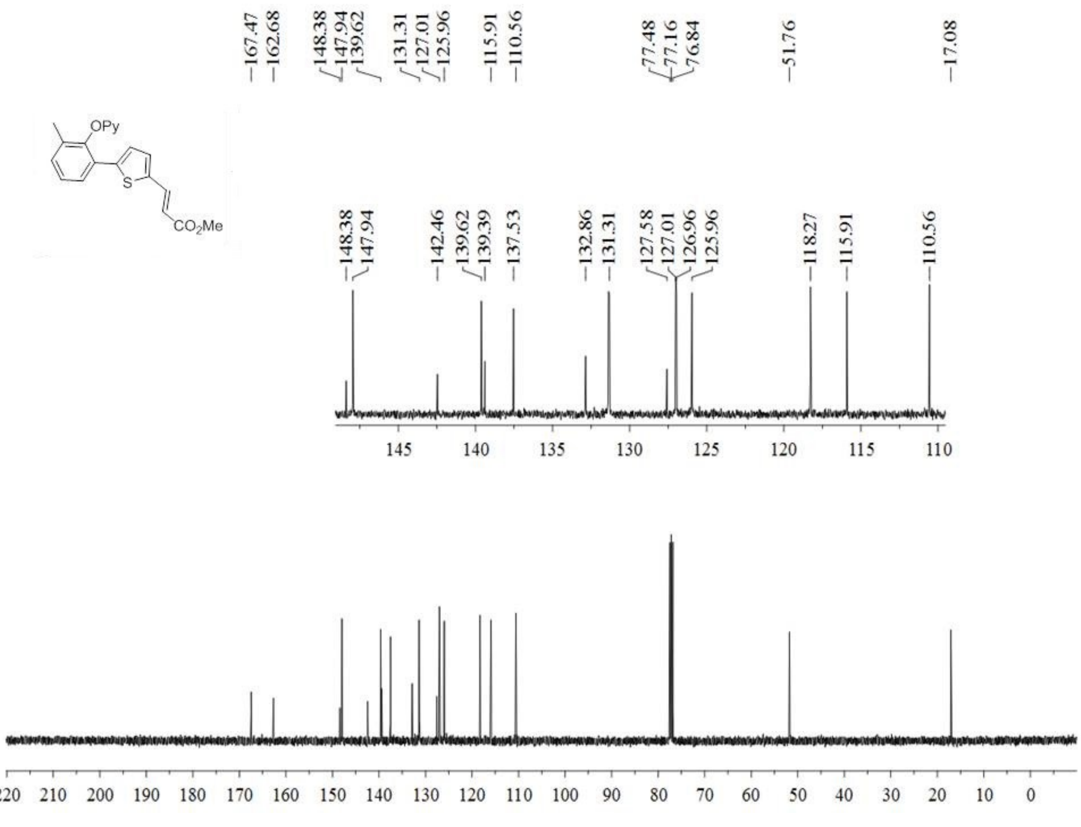
167.47
162.68
148.38
147.94
139.62
131.31
127.01
125.96
115.91
110.56
77.48
77.16
76.84
51.76
17.08
OPy
S
CO2Me
148.38
147.94
142.46
139.62
139.39
137.53
132.86
131.31
127.58
127.01
126.96
125.96
118.27
115.91
110.56
145
140
135
130
125
120
115
110
220
210
200
190
180
170
160
150
140
130
120
110
100
90
80
70
60
50
40
30
20
10
0

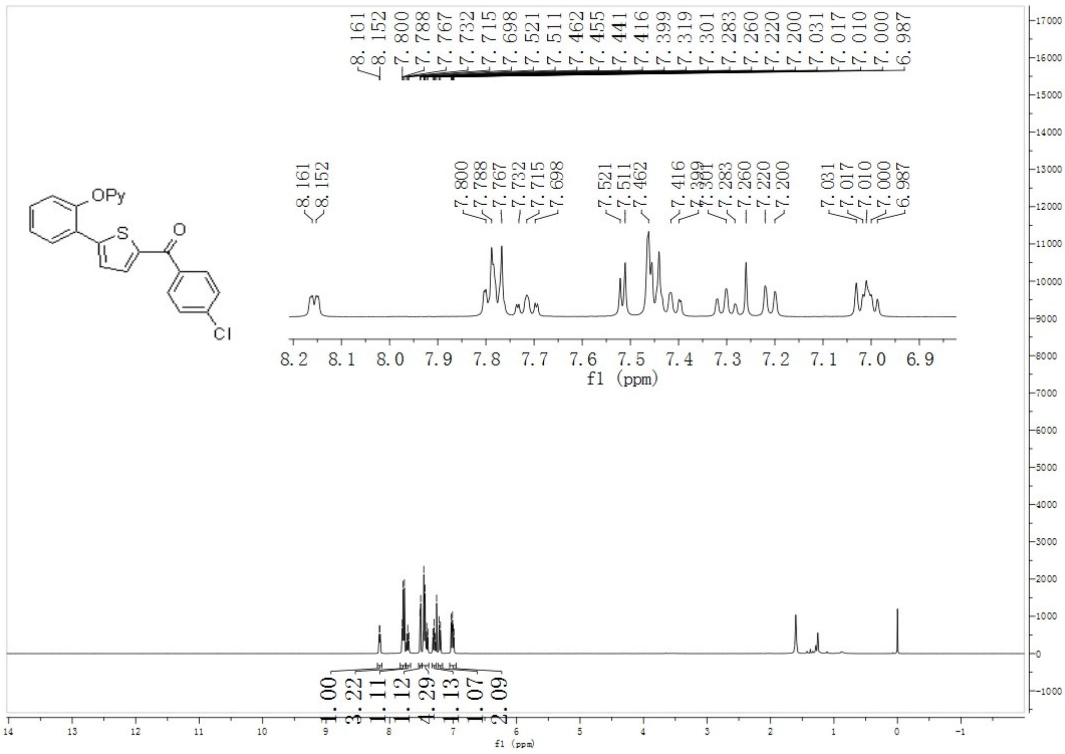

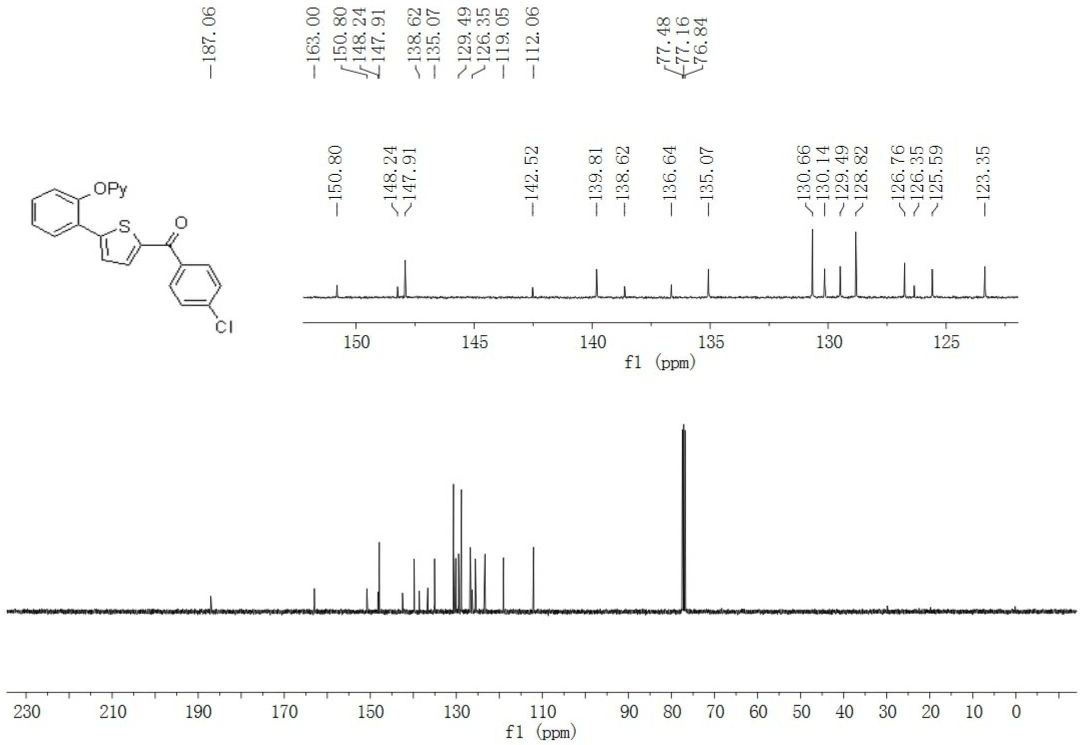

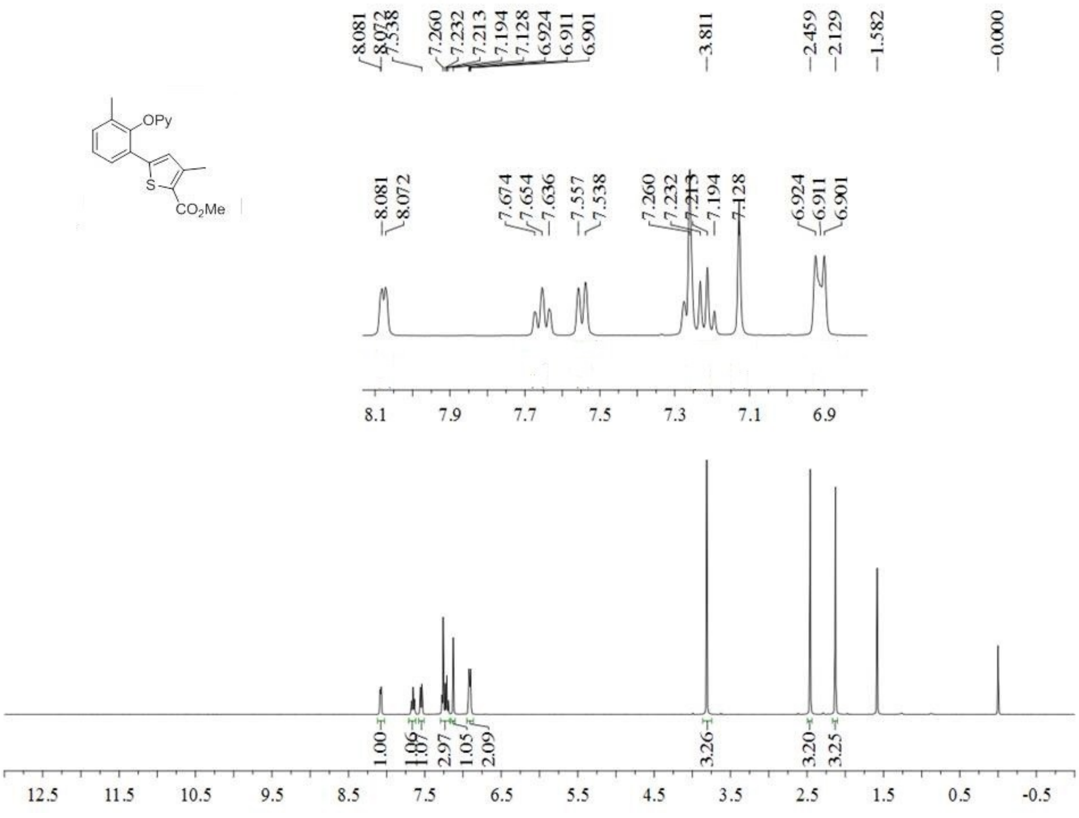
OPy
S
CO2Me
8.081
8.072
7.538
7.260
7.232
7.213
7.194
7.128
6.924
6.911
6.901
3.811
2.459
2.129
1.582
0.000
7.674
7.654
7.636
7.557
7.538
1.00
1.06
1.07
2.97
1.05
2.09
3.26
3.20
3.25

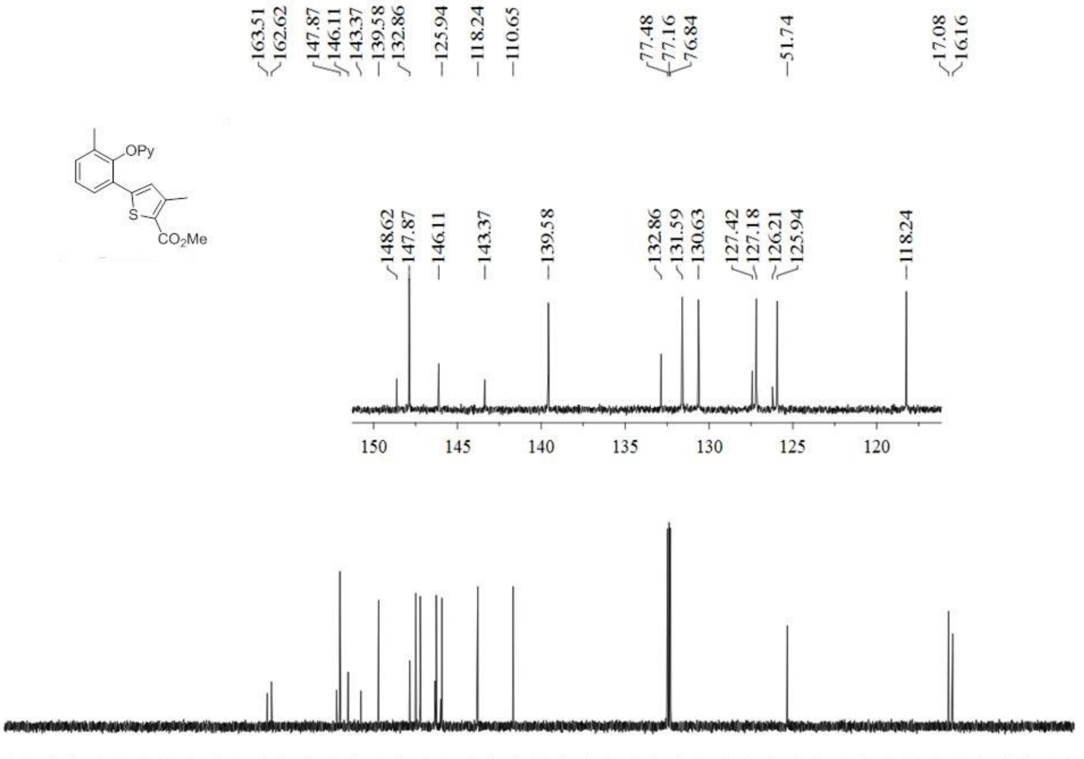
OPy
S
CO2Me
163.51
162.62
147.87
146.11
143.37
139.58
132.86
125.94
118.24
110.65
77.48
77.16
76.84
51.74
17.08
16.16
148.62
131.59
130.63
127.42
127.18
126.21

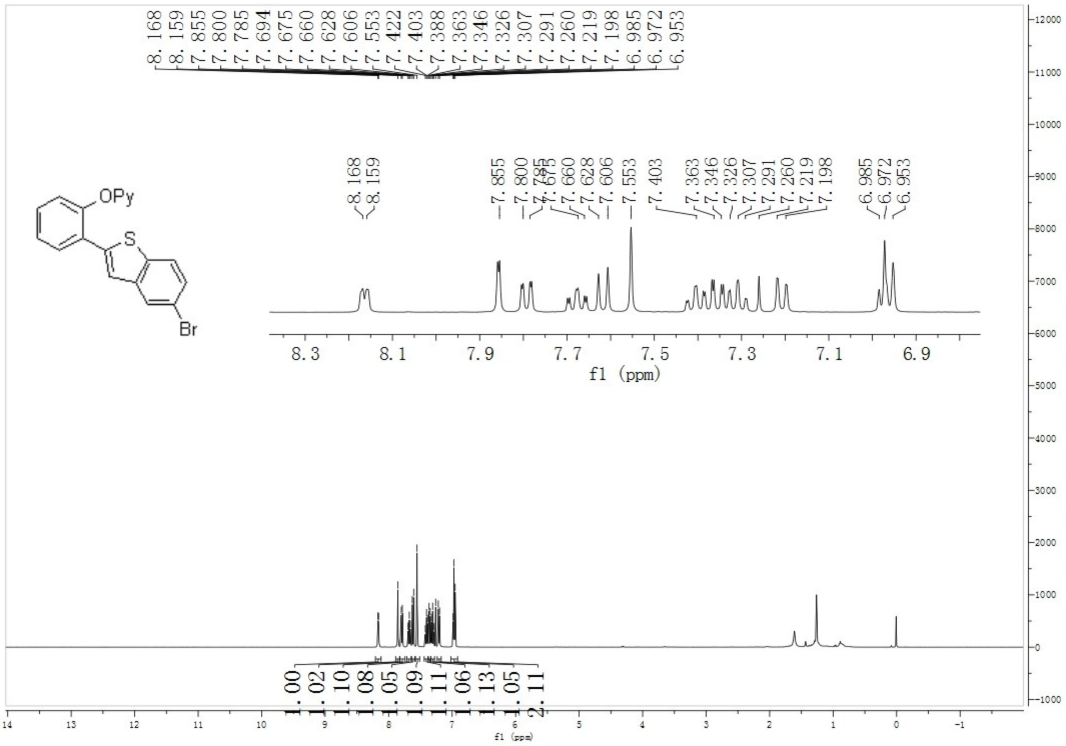
8.168
8.159
7.855
7.800
7.785
7.694
7.675
7.660
7.628
7.606
7.553
7.422
7.403
7.388
7.363
7.346
7.326
7.307
7.291
7.260
7.219
7.198
6.985
6.972
6.953
OPy
S
Br
f1 (ppm)
1.00
1.02
1.10
1.08
1.05
1.09
1.11
1.06
1.13
1.05
2.11

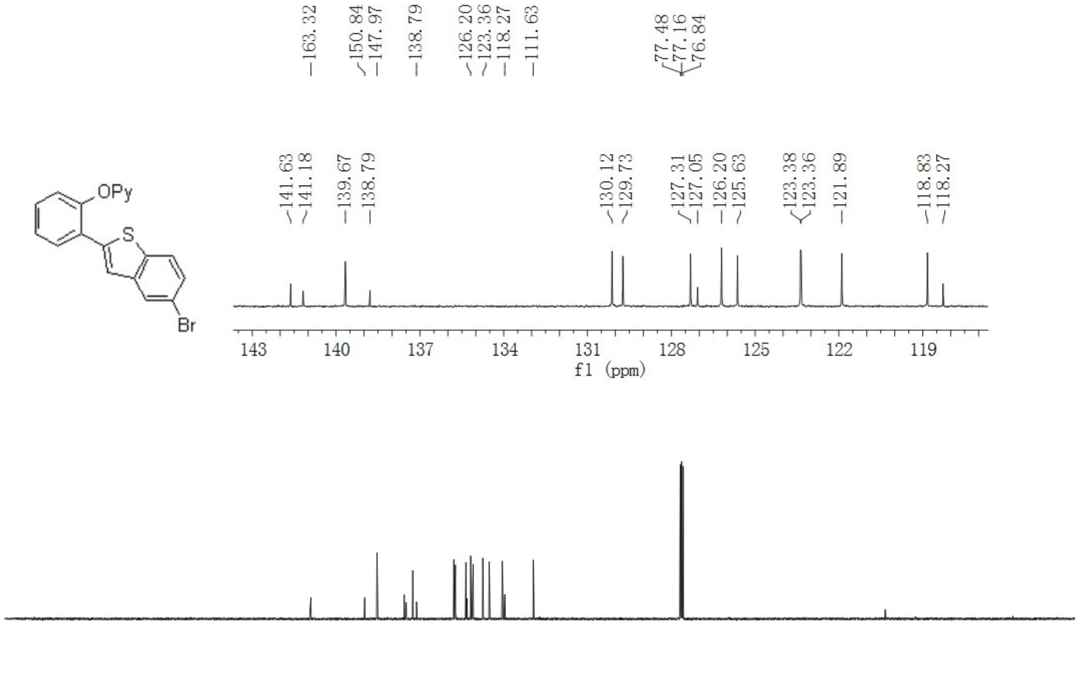
163.32
150.84
147.97
138.79
126.20
123.36
118.27
111.63
77.48
77.16
76.84
OPy
S
Br
141.63
141.18
139.67
138.79
130.12
129.73
127.31
127.05
126.20
125.63
123.38
123.36
121.89
118.83
118.27
f1 (ppm)

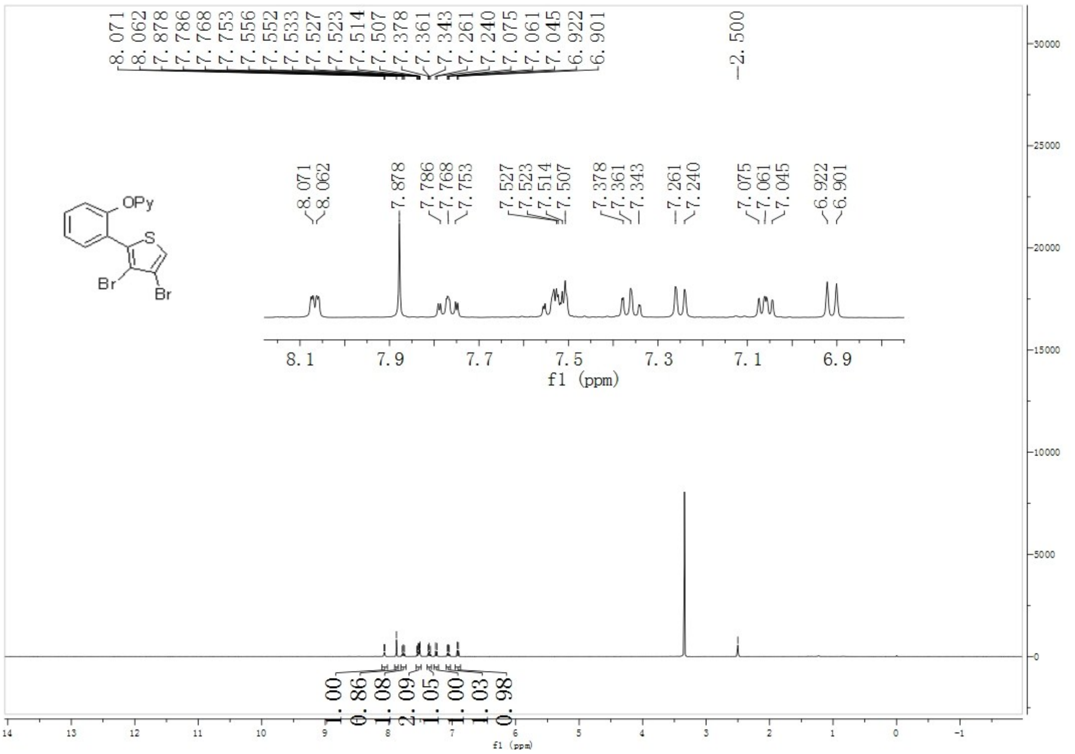
OPy
S
Br
Br
8.071
8.062
7.878
7.786
7.768
7.753
7.527
7.523
7.514
7.507
7.378
7.361
7.343
7.261
7.240
7.075
7.061
7.045
6.922
6.901
2.500
1.00
0.86
1.08
2.09
2.05
1.00
1.03
0.98
f1 (ppm)

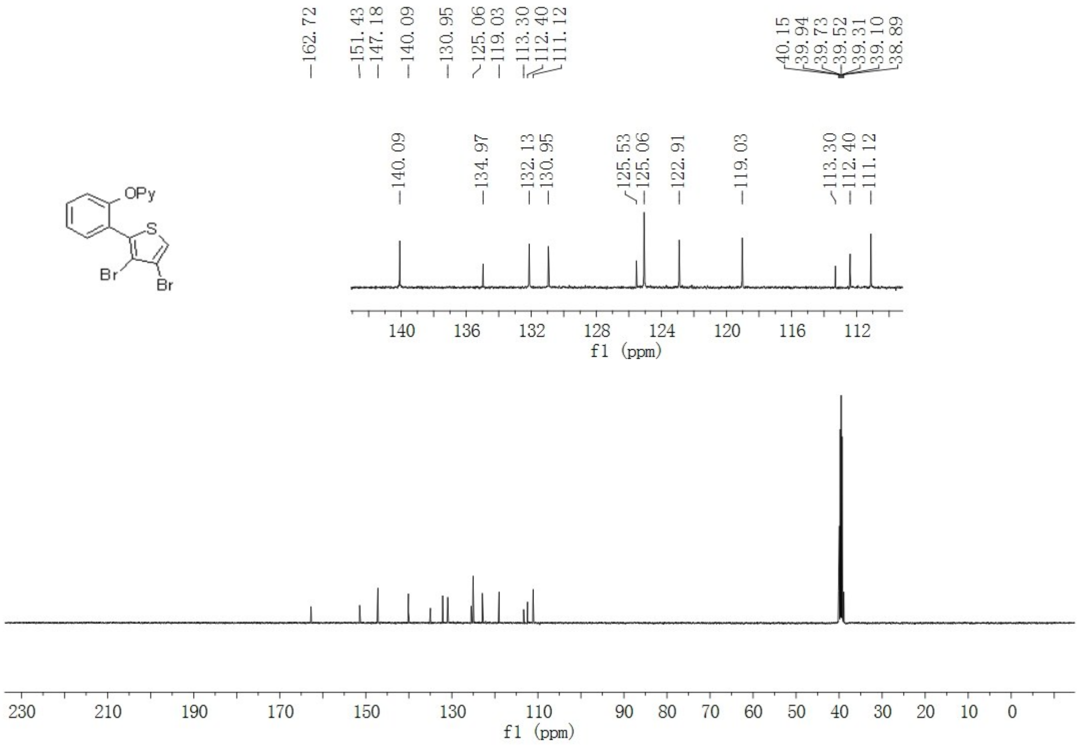
OPy
S
Br
Br
162.72
151.43
147.18
140.09
130.95
125.06
119.03
113.30
112.40
111.12
40.15
39.94
39.73
39.52
39.31
39.10
38.89
134.97
132.13
125.53
122.91
f1 (ppm)

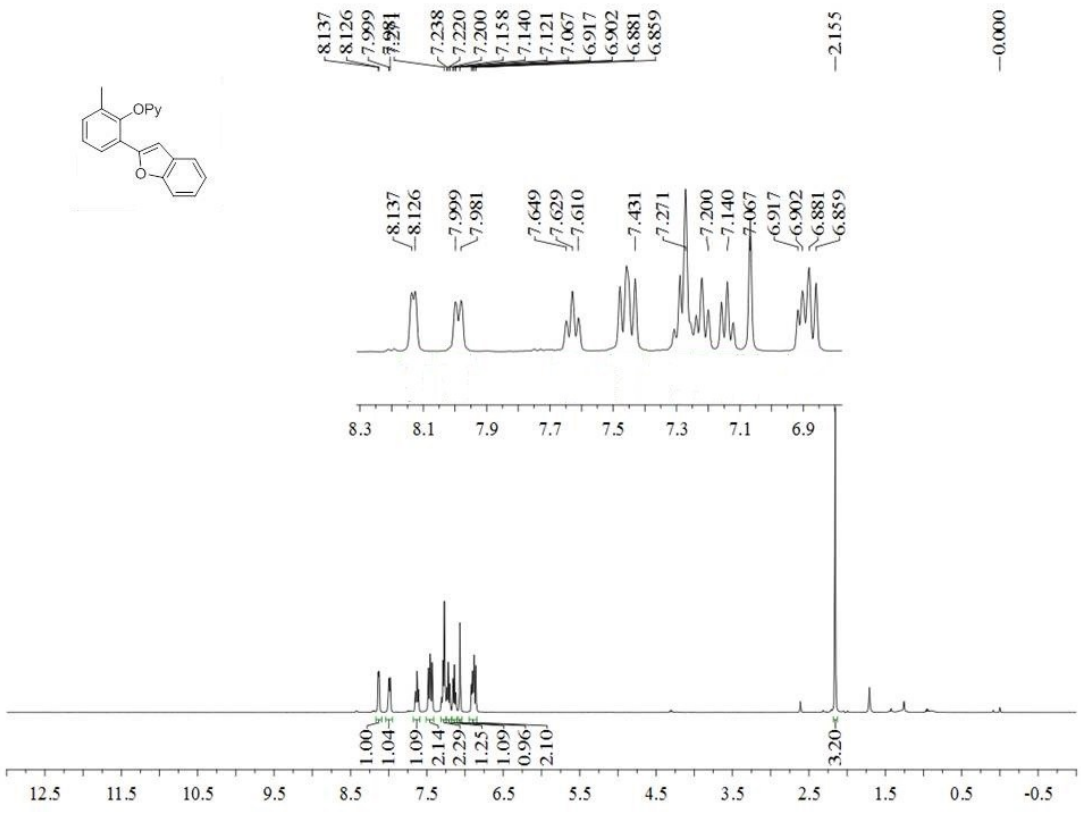
OPy
8.137
8.126
7.999
7.981
7.271
7.238
7.220
7.200
7.158
7.140
7.121
7.067
6.917
6.902
6.881
6.859
2.155
0.000
8.137
8.126
7.999
7.981
7.649
7.629
7.610
7.431
7.271
7.200
7.140
7.067
6.917
6.902
6.881
6.859
8.3
8.1
7.9
7.7
7.5
7.3
7.1
6.9
1.00
1.04
1.09
2.14
2.29
1.25
1.09
0.96
2.10
3.20
12.5
11.5
10.5
9.5
8.5
7.5
6.5
5.5
4.5
3.5
2.5
1.5
0.5
-0.5

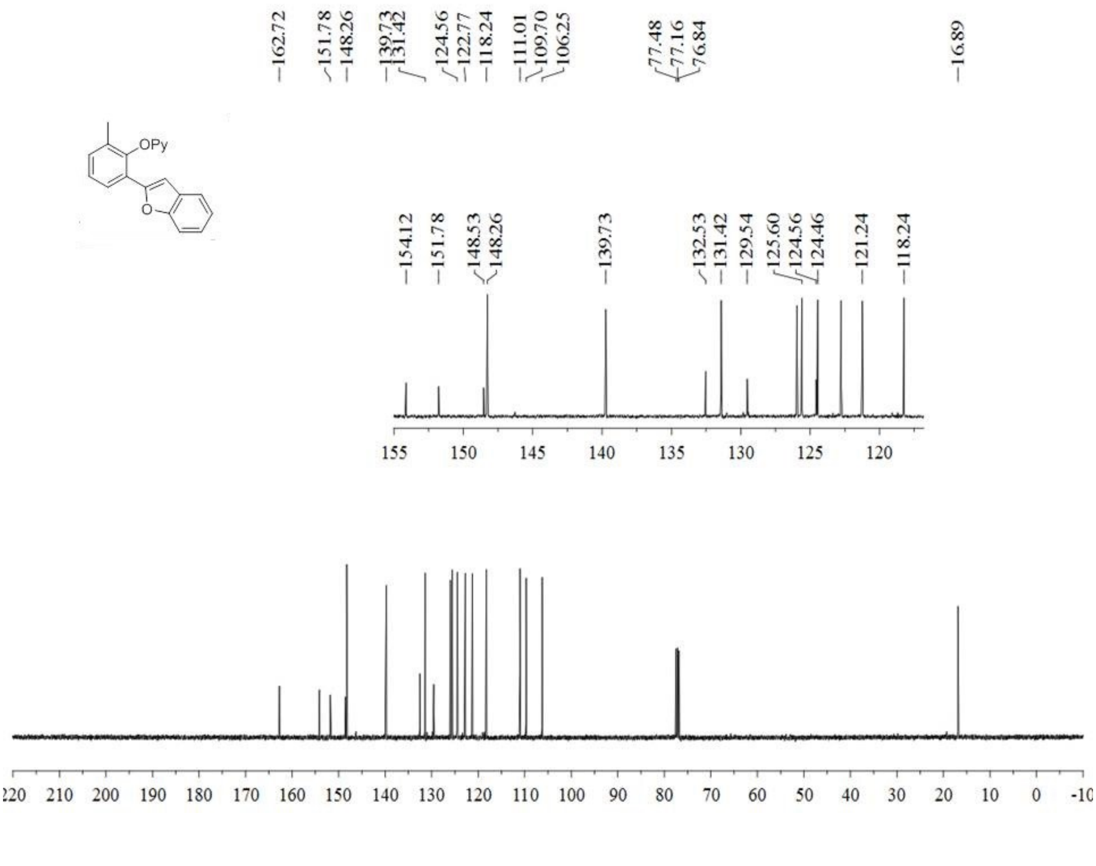
162.72
151.78
148.26
139.73
131.42
124.56
122.77
118.24
111.01
109.70
106.25
77.48
77.16
76.84
16.89
OPy
154.12
151.78
148.53
148.26
139.73
132.53
131.42
129.54
125.60
124.56
124.46
121.24
118.24
155
150
145
140
135
130
125
120
220
210
200
190
180
170
160
150
140
130
120
110
100
90
80
70
60
50
40
30
20
10
0
-10

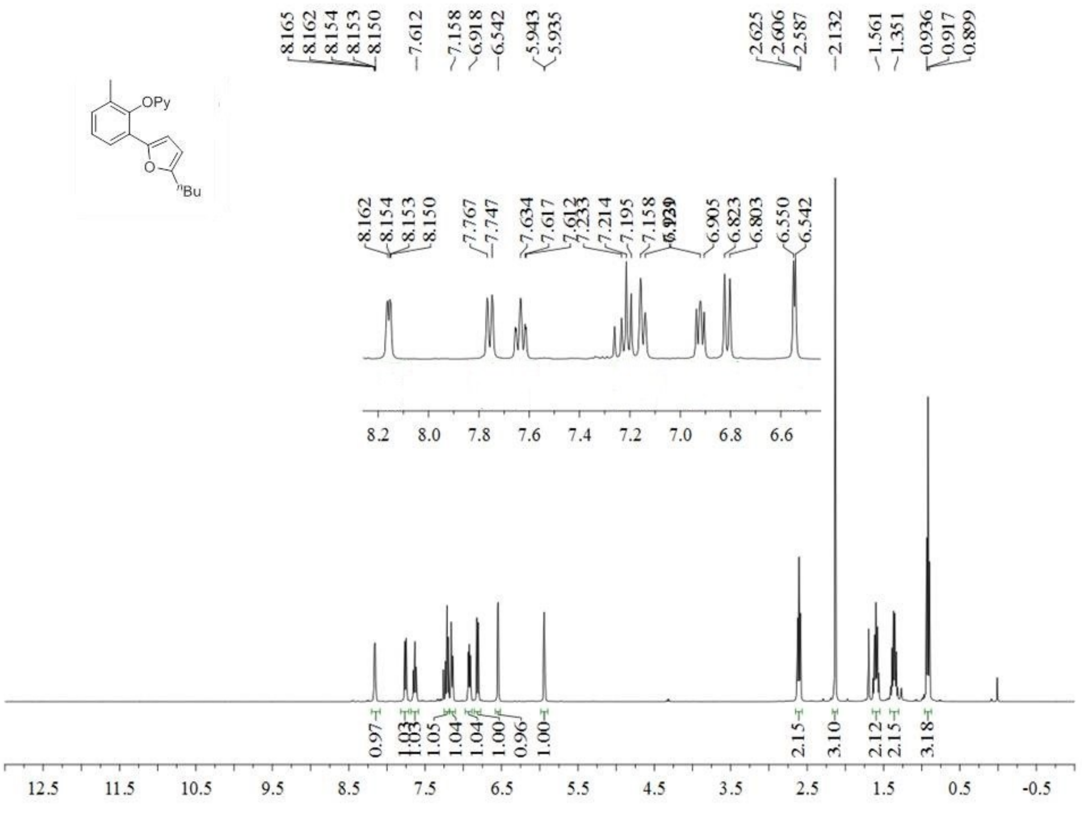
OPy
nBu
8.165 8.162 8.154 8.153 8.150 7.612 7.158 6.918 6.542 5.943 5.935 2.625 2.606 2.587 2.132 1.561 1.351 0.936 0.917 0.899
8.162 8.154 8.153 8.150 7.767 7.747 7.634 7.617 7.612 7.233 7.214 7.195 7.158 6.905 6.823 6.803 6.550 6.542
8.2 8.0 7.8 7.6 7.4 7.2 7.0 6.8 6.6
0.97 1.03 1.03 1.05 1.04 1.04 1.00 0.96 1.00 2.15 3.10 2.12 2.15 3.18
12.5 11.5 10.5 9.5 8.5 7.5 6.5 5.5 4.5 3.5 2.5 1.5 0.5 -0.5

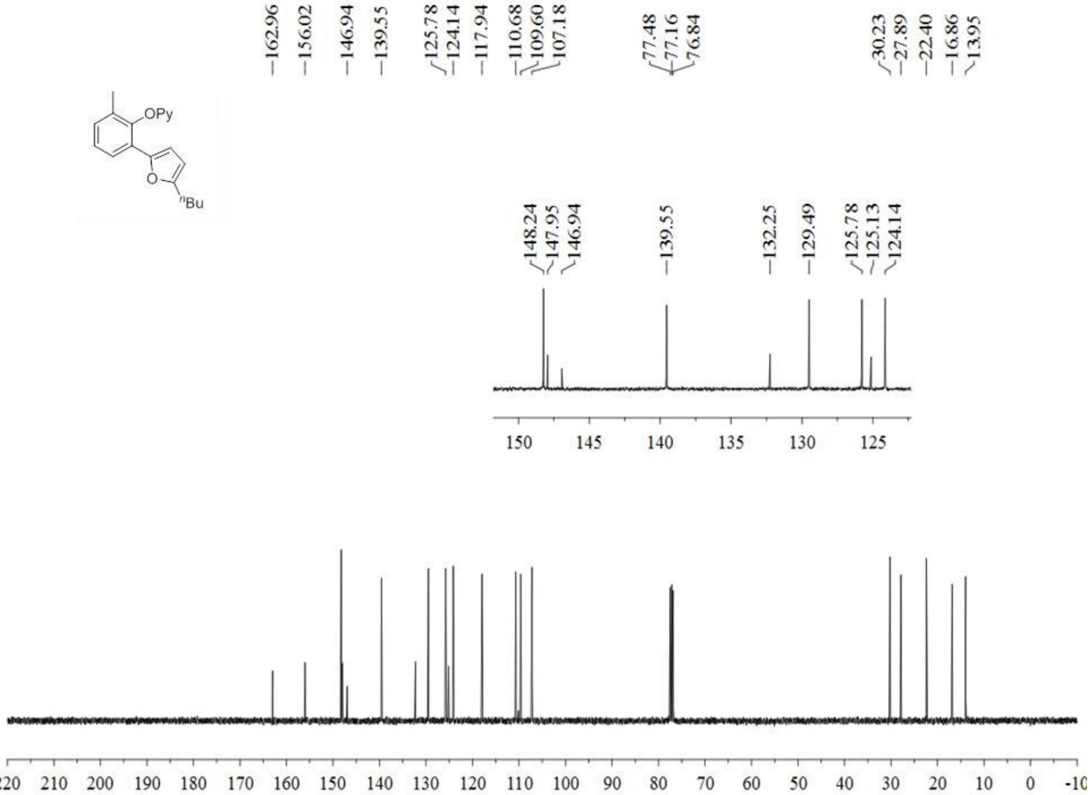
162.96 156.02 146.94 139.55 125.78 124.14 117.94 110.68 109.60 107.18 77.48 77.16 76.84 30.23 27.89 22.40 16.86 13.95
OPy
nBu
148.24 147.95 146.94 139.55 132.25 129.49 125.78 125.13 124.14
150 145 140 135 130 125
220 210 200 190 180 170 160 150 140 130 120 110 100 90 80 70 60 50 40 30 20 10 0 -10

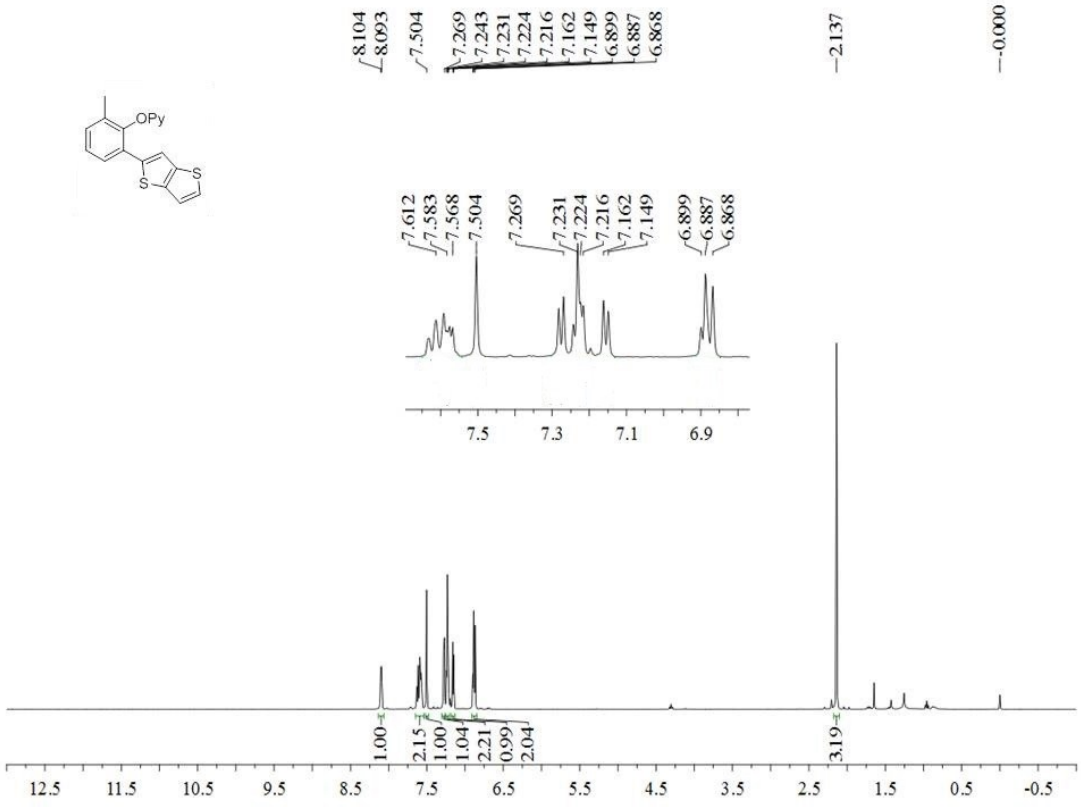
OPy
S
S
8.104
8.093
7.504
7.269
7.243
7.231
7.224
7.216
7.162
7.149
6.899
6.887
6.868
2.137
0.000
7.612
7.583
7.568
7.5
7.3
7.1
6.9
1.00
2.15
1.00
1.04
2.21
0.99
2.04
3.19
12.5
11.5
10.5
9.5
8.5
7.5
6.5
5.5
4.5
3.5
2.5
1.5
0.5
-0.5

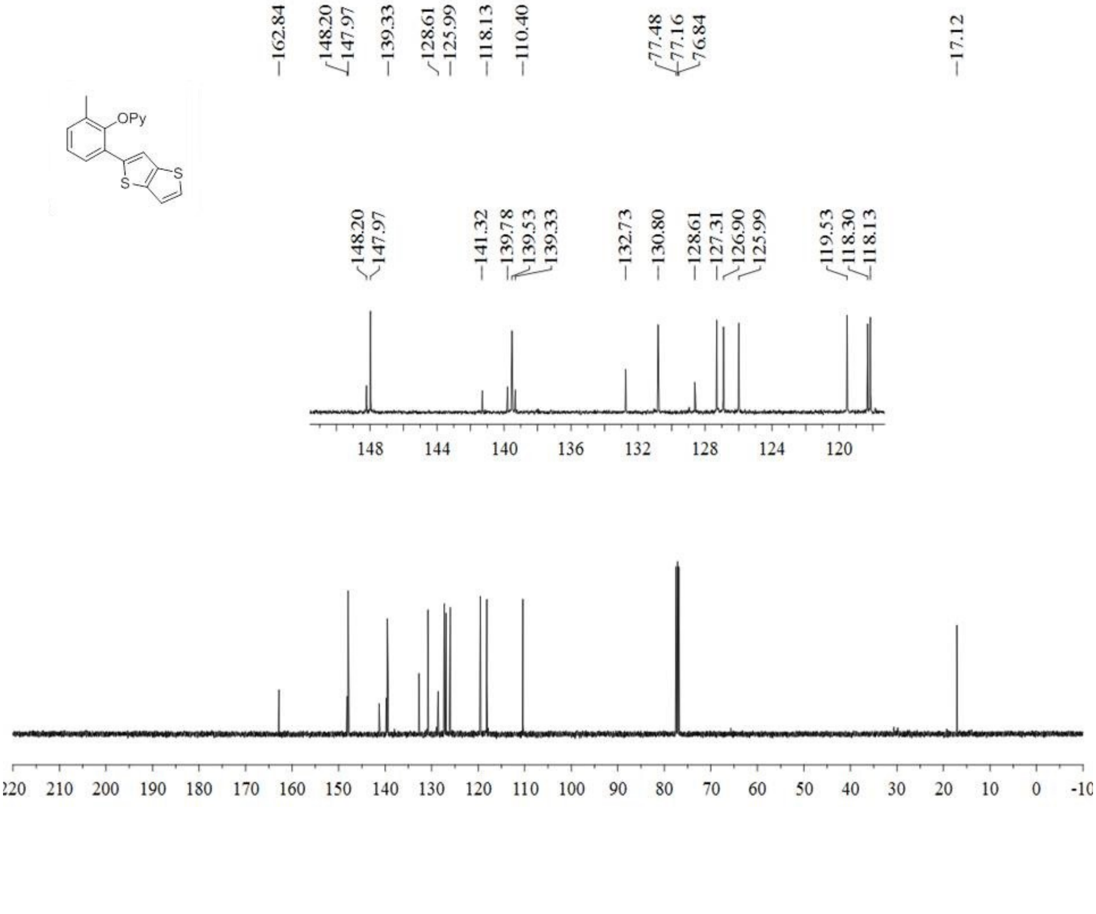
OPy
S
S
162.84
148.20
147.97
139.33
128.61
125.99
118.13
110.40
77.48
77.16
76.84
17.12
141.32
139.78
139.53
132.73
130.80
127.31
126.90
119.53
118.30
148
144
140
136
132
128
124
120
220
210
200
190
180
170
160
150
140
130
120
110
100
90
80
70
60
50
40
30
20
10
0
-10

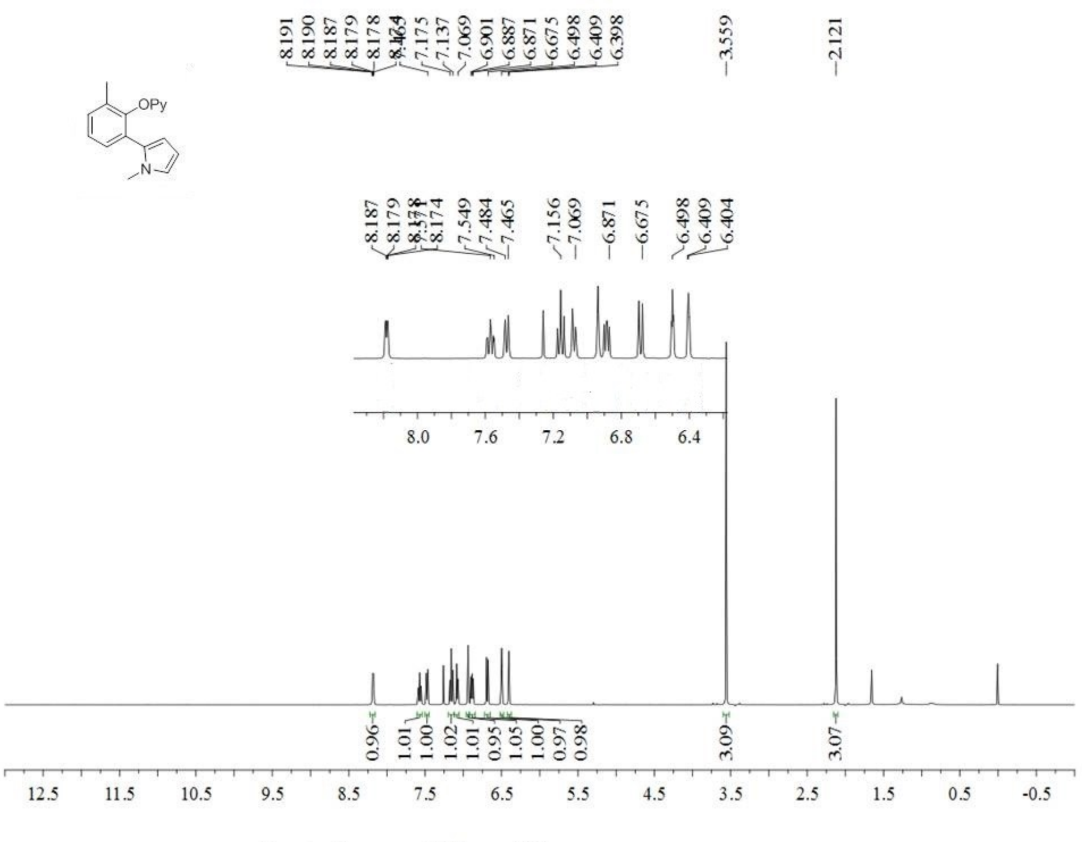
OPy
N
8.191
8.190
8.187
8.179
8.178
7.175
7.137
7.069
6.901
6.887
6.871
6.675
6.498
6.409
6.398
3.559
2.121
8.187
8.179
8.174
7.549
7.484
7.465
7.156
7.069
6.871
6.675
6.498
6.409
6.404
8.0
7.6
7.2
6.8
6.4
0.96
1.01
1.00
1.02
1.01
0.95
1.05
1.00
0.97
0.98
3.09
3.07
12.5
11.5
10.5
9.5
8.5
7.5
6.5
5.5
4.5
3.5
2.5
1.5
0.5
-0.5

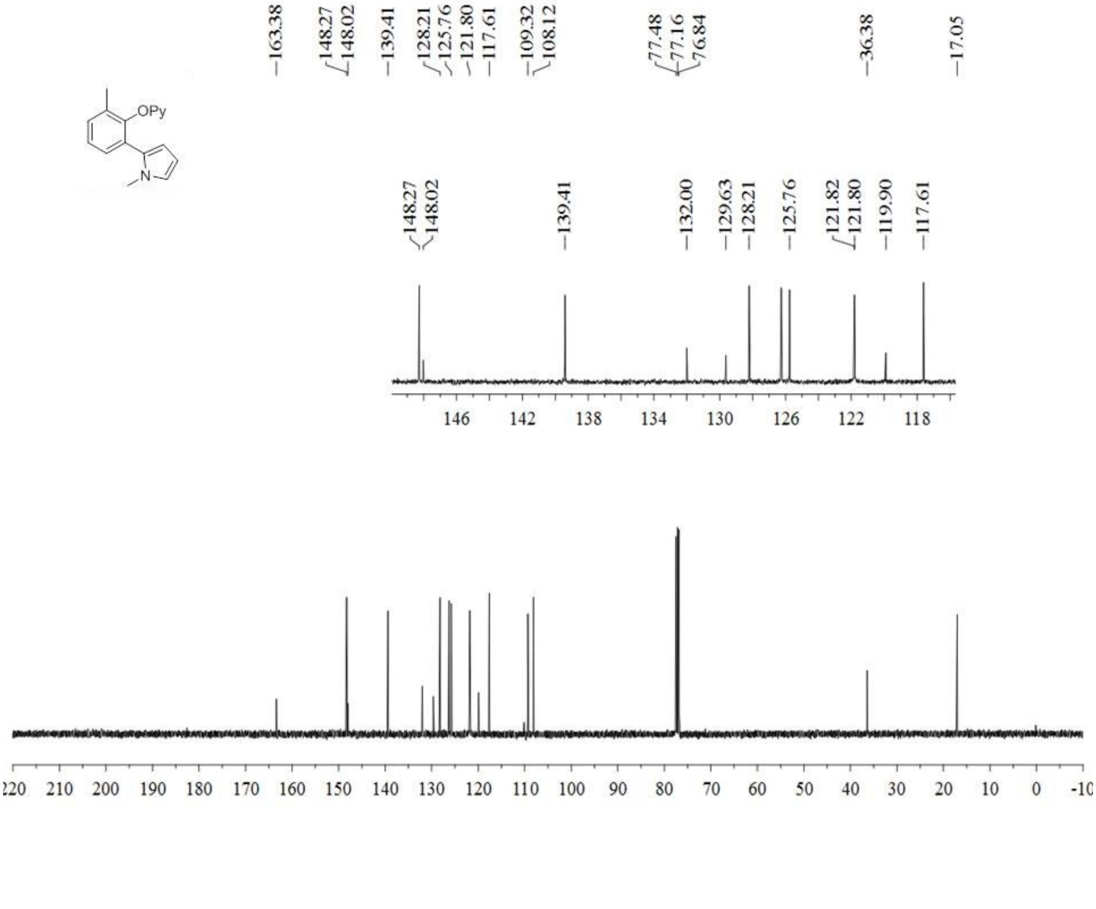
163.38
148.27
148.02
139.41
128.21
125.76
121.80
117.61
109.32
108.12
77.48
77.16
76.84
36.38
17.05
OPy
N
148.27
148.02
139.41
132.00
129.63
128.21
125.76
121.82
121.80
119.90
117.61
146
142
138
134
130
126
122
118
220
210
200
190
180
170
160
150
140
130
120
110
100
90
80
70
60
50
40
30
20
10
0
-10

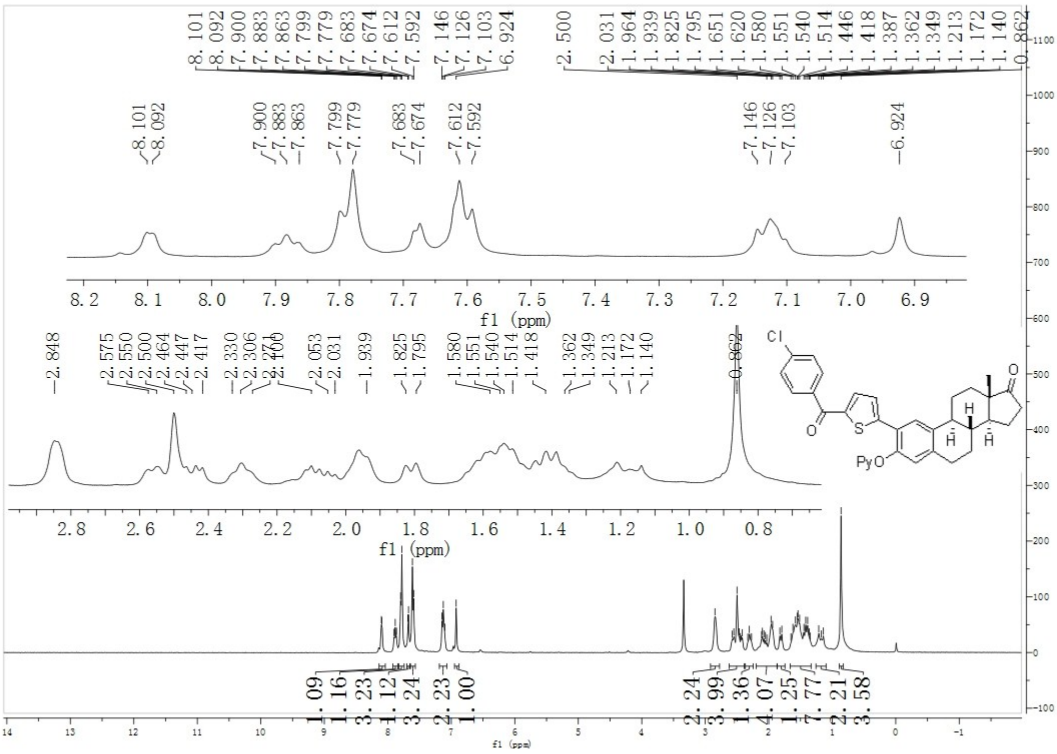
8.101 8.092 7.900 7.883 7.863 7.799 7.779 7.683 7.674 7.612 7.592 7.146 7.126 7.103 6.924 2.500 2.031 1.964 1.939 1.825 1.795 1.651 1.620 1.580 1.551 1.540 1.514 1.446 1.418 1.387 1.362 1.349 1.213 1.172 1.140 0.862
1.09 1.16 3.23 1.12 3.24 2.23 1.00 2.24 3.99 1.36 4.07 1.25 7.77 2.21 3.58
f1 (ppm)
Cl
O
S
H
PyO

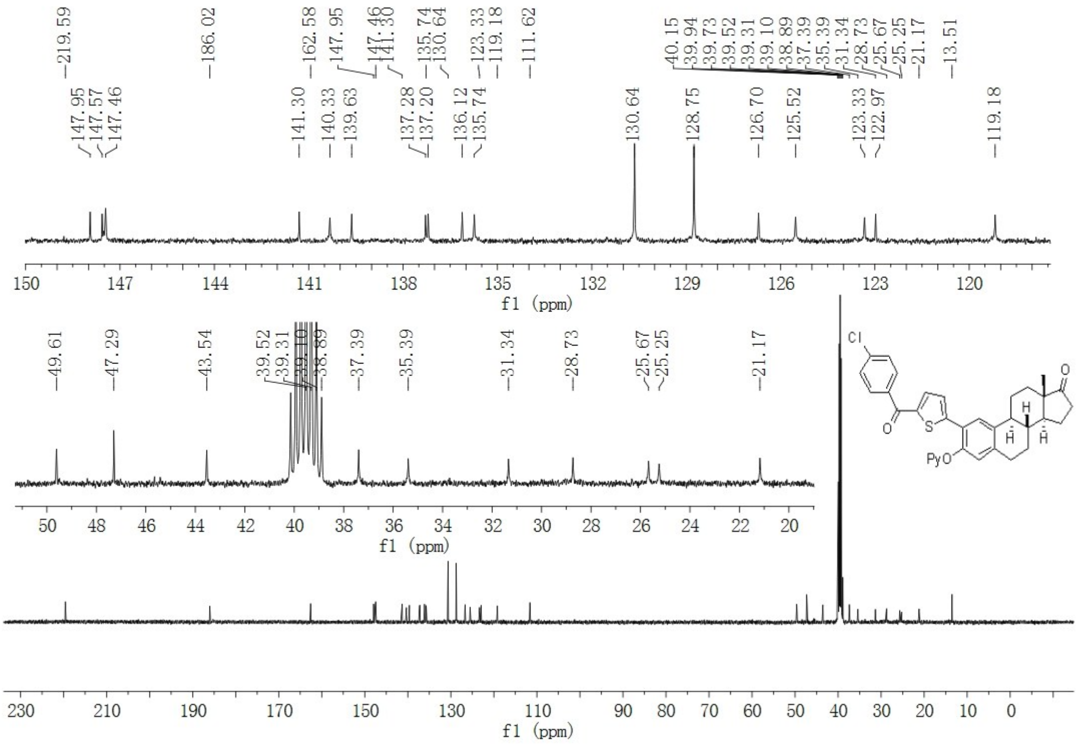
219.59 186.02 162.58 147.95 147.46 141.30 135.74 130.64 123.33 119.18 111.62 40.15 39.94 39.73 39.52 39.31 39.10 38.89 37.39 35.39 31.34 28.73 25.67 25.25 21.17 13.51
f1 (ppm)
Cl
O
S
PyO

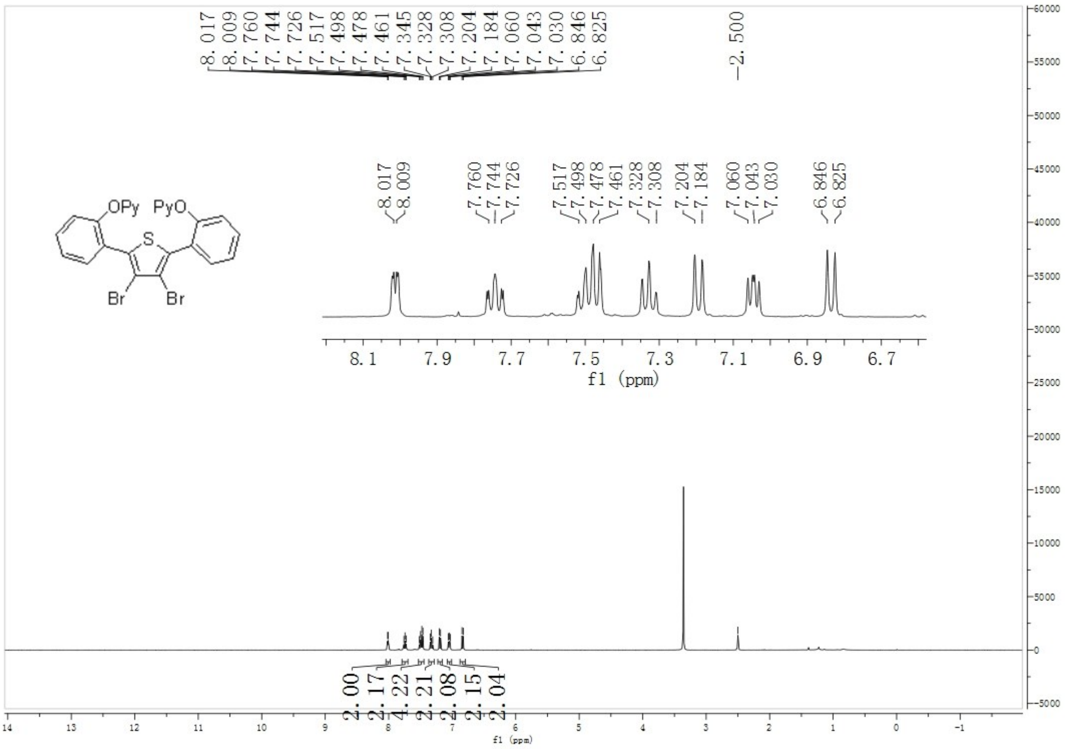

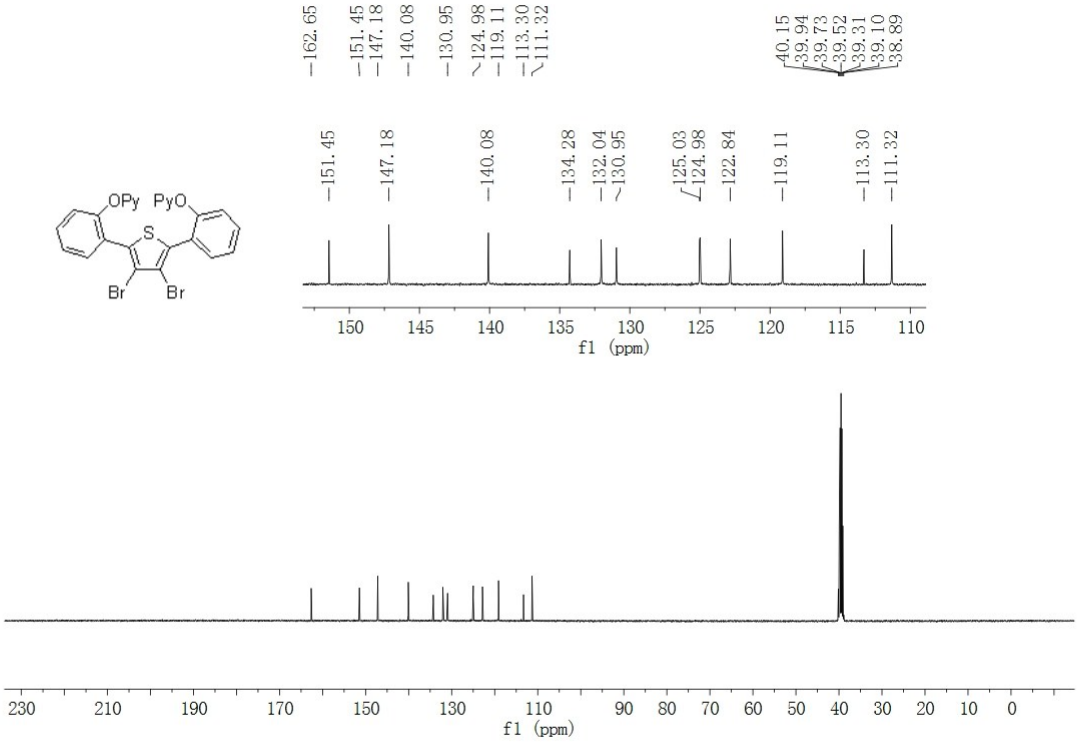

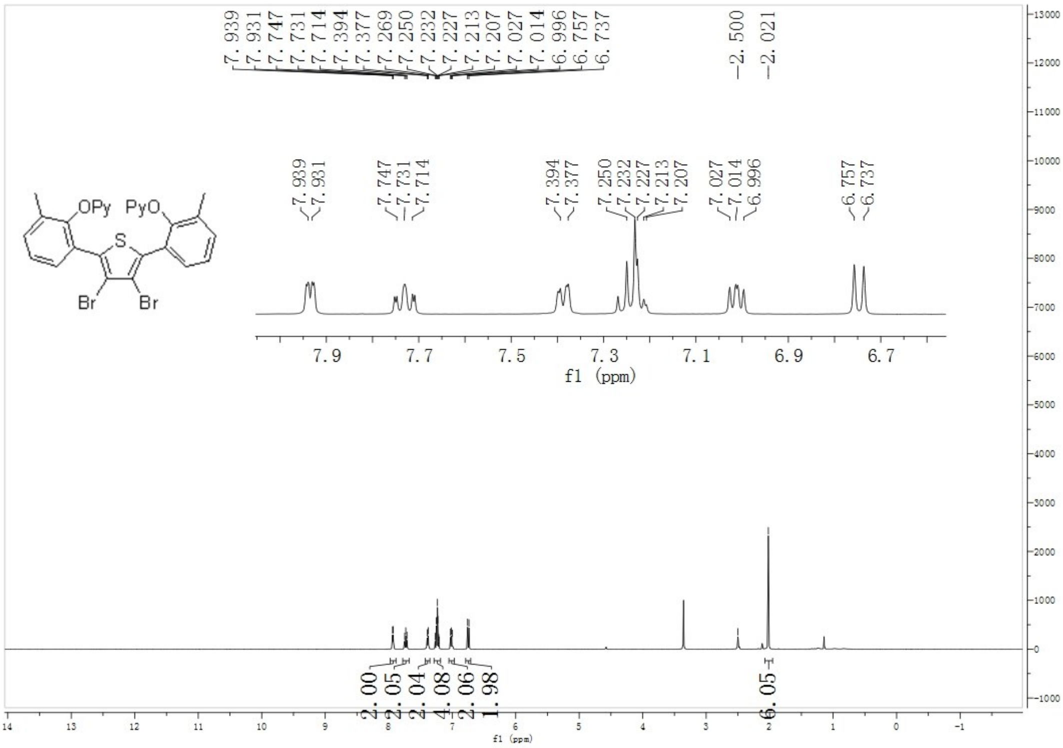
7.939 7.931 7.747 7.731 7.714 7.394 7.377 7.269 7.250 7.232 7.227 7.213 7.207 7.027 7.014 6.996 6.757 6.737
2.500
2.021
OPy PyO
S
Br Br
2.00 2.05 2.04 4.08 2.06 1.98
6.05
f1 (ppm)

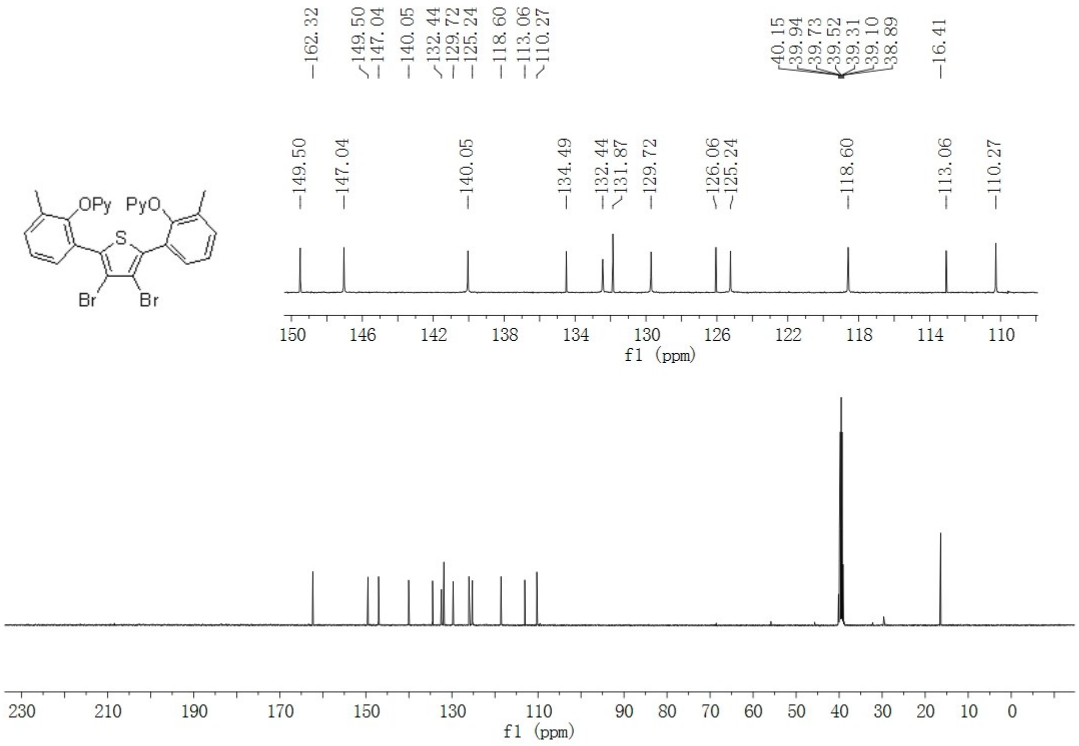
162.32
149.50 147.04
140.05
132.44 129.72 125.24
118.60
113.06 110.27
40.15 39.94 39.73 39.52 39.31 39.10 38.89
16.41
134.49 131.87 126.06
OPy PyO
S
Br Br
f1 (ppm)

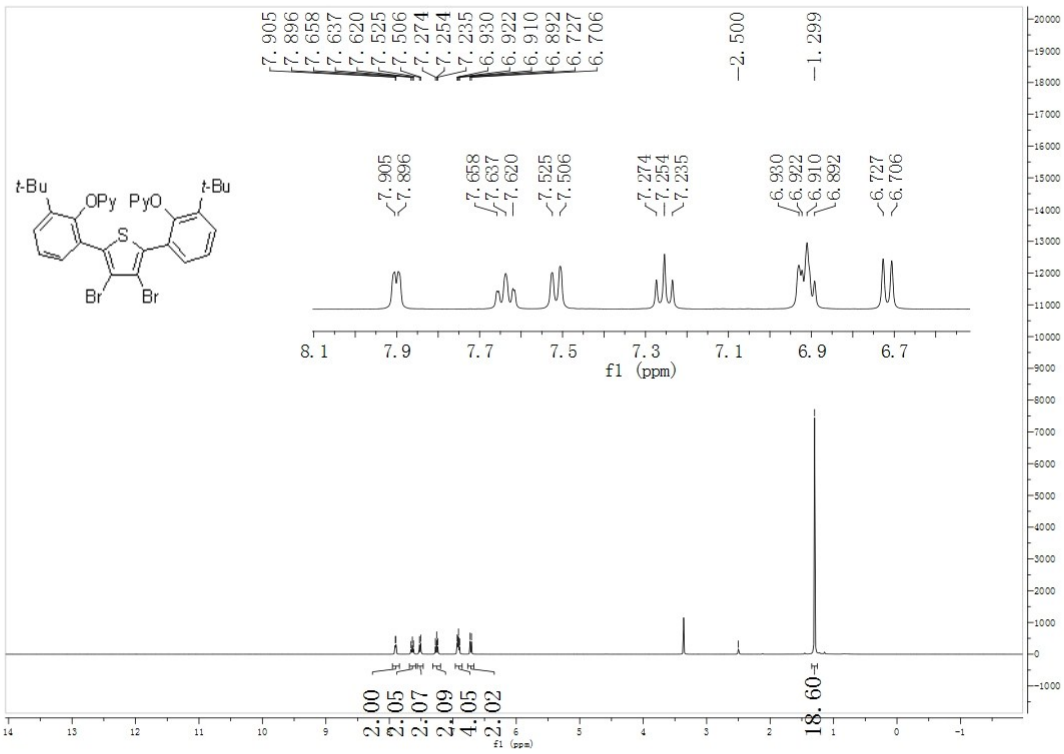
t-Bu
OPy
PyO
t-Bu
S
Br
Br
7.905
7.896
7.658
7.637
7.620
7.525
7.506
7.274
7.254
7.235
6.930
6.922
6.910
6.892
6.727
6.706
2.500
1.299
2.00
2.05
2.07
2.09
4.05
2.02
18.60
f1 (ppm)

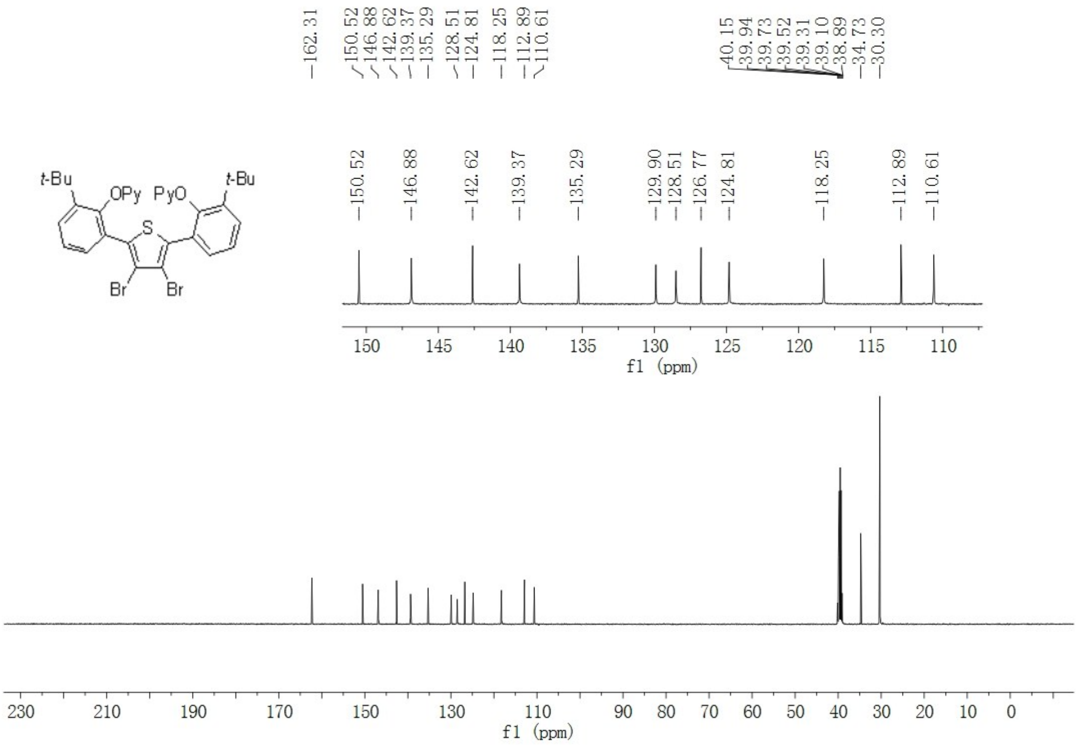
t-Bu
OPy
PyO
t-Bu
S
Br
Br
162.31
150.52
146.88
142.62
139.37
135.29
129.90
128.51
126.77
124.81
118.25
112.89
110.61
40.15
39.94
39.73
39.52
39.31
39.10
38.89
34.73
30.30
f1 (ppm)

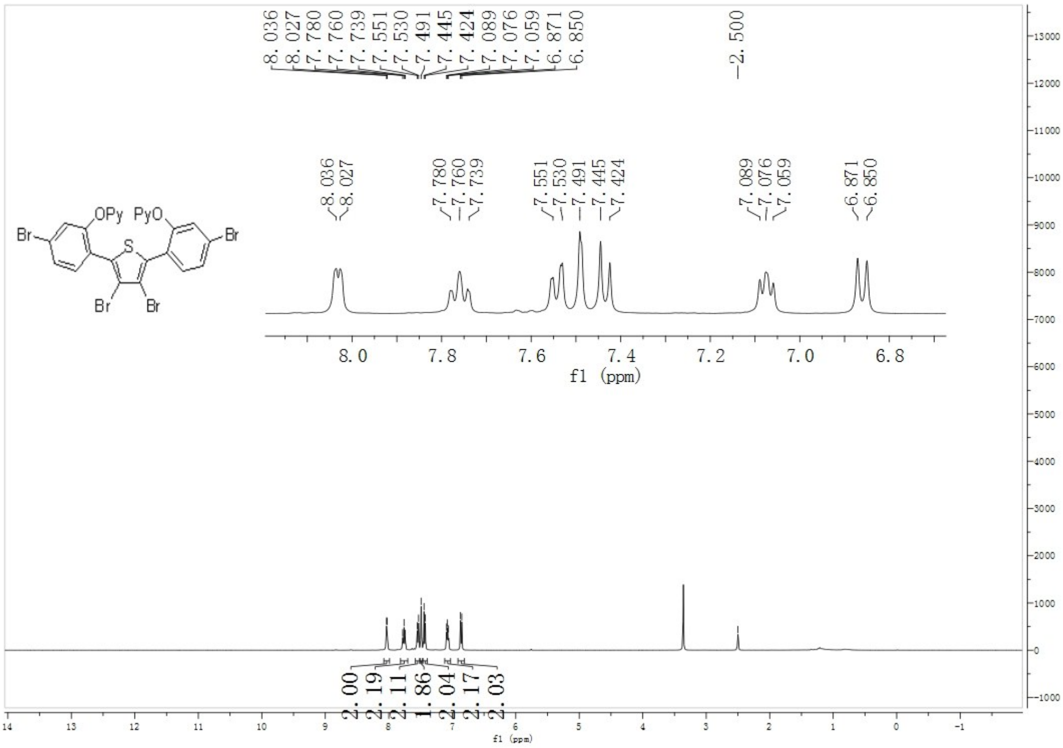
8.036 8.027 7.780 7.760 7.739 7.551 7.530 7.491 7.445 7.424 7.089 7.076 7.059 6.871 6.850
2.500
OPy PyO
Br S Br
Br Br
2.00 2.19 2.11 1.86 2.04 2.17 2.03
f1 (ppm)

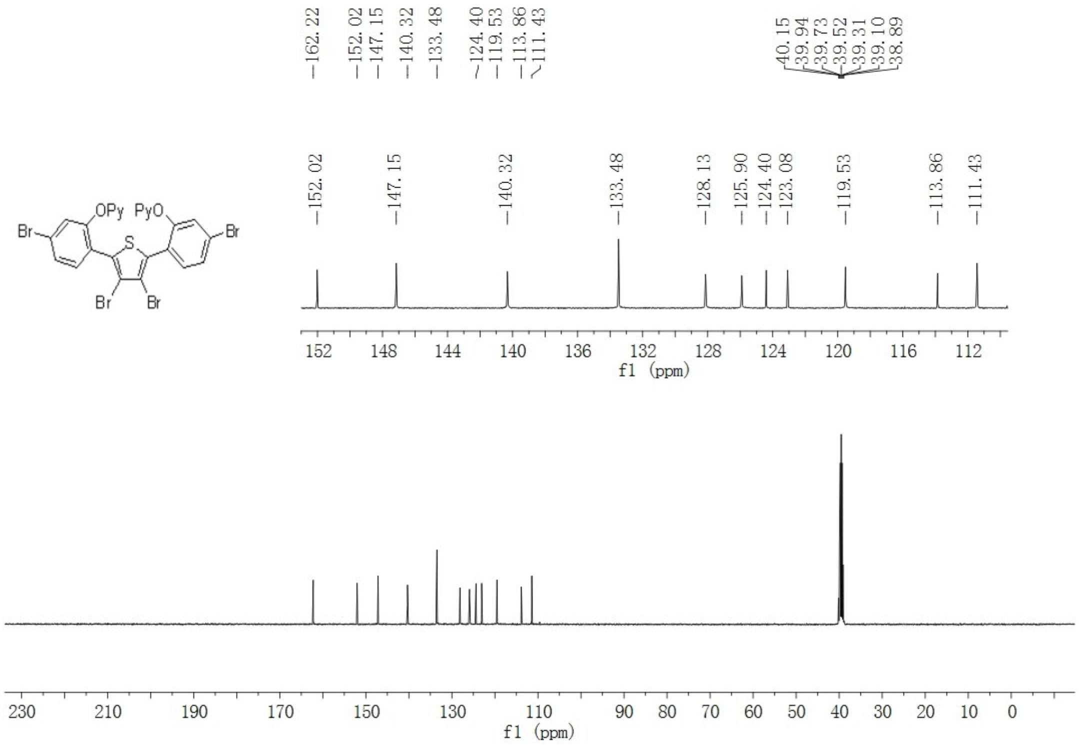
162.22 152.02 147.15 140.32 133.48 124.40 119.53 113.86 111.43
40.15 39.94 39.73 39.52 39.31 39.10 38.89
152.02 147.15 140.32 133.48 128.13 125.90 124.40 123.08 119.53 113.86 111.43
OPy PyO
Br S Br
Br Br
f1 (ppm)

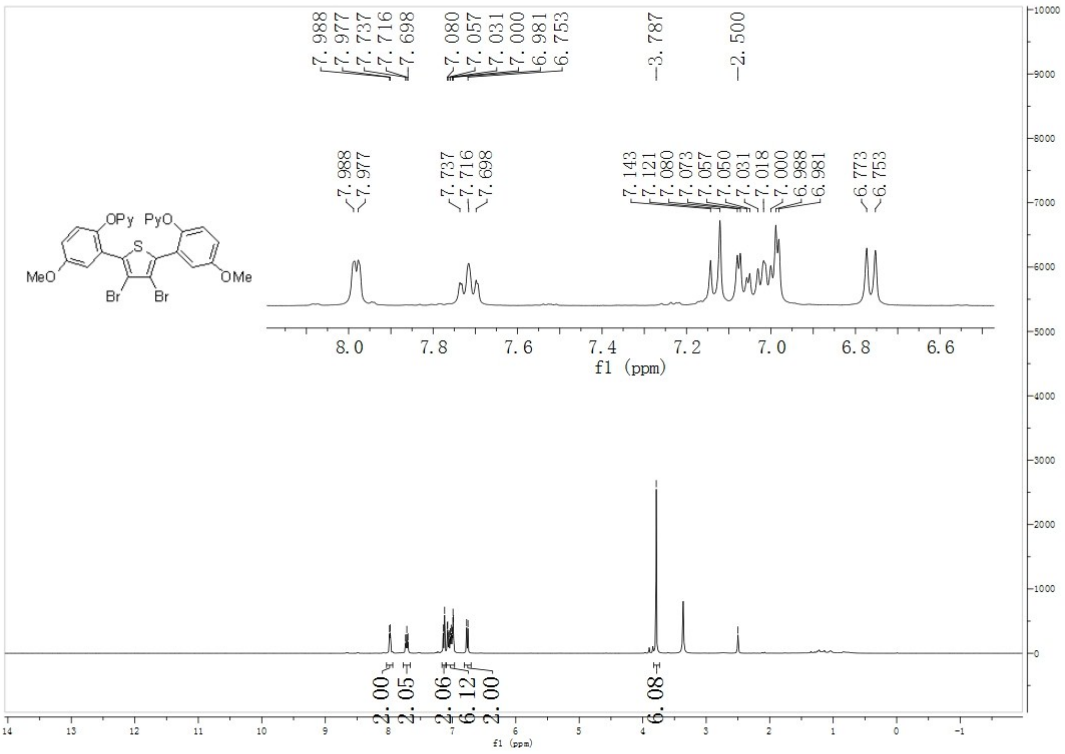
7.988
7.977
7.737
7.716
7.698
7.080
7.057
7.031
7.000
6.981
6.753
3.787
2.500
7.143
7.121
7.073
7.050
7.018
6.988
6.773
OPy PyO
S
MeO
OMe
Br Br
f1 (ppm)
2.00
2.05
2.06
6.12
2.00
6.08

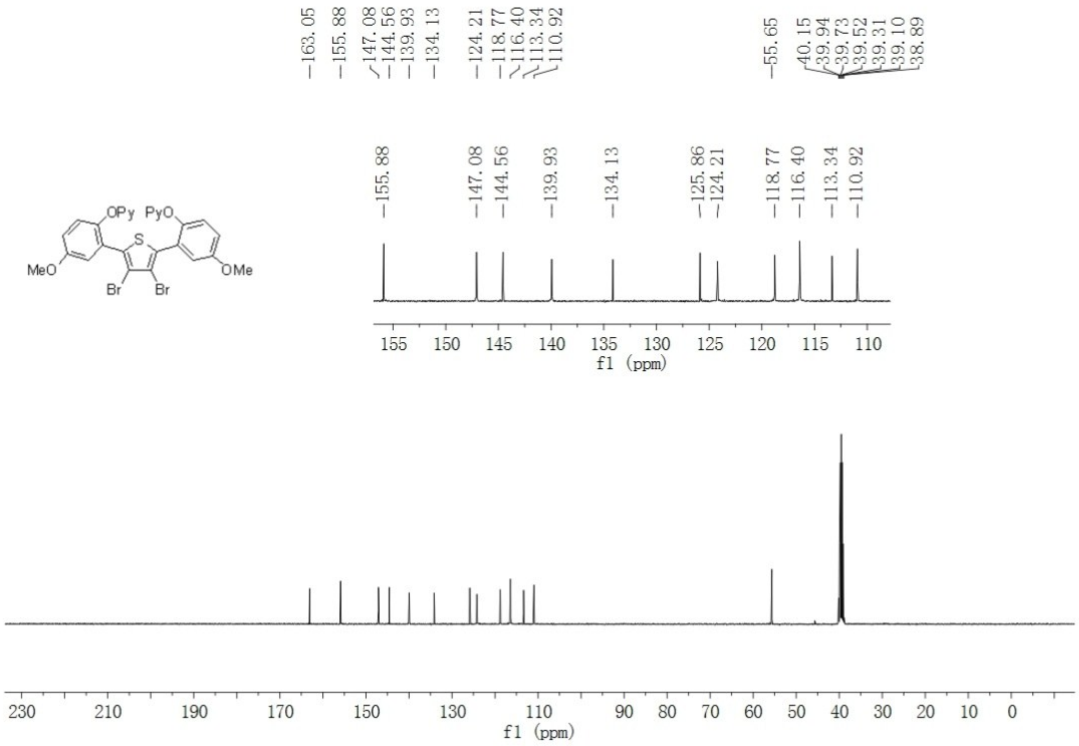
163.05
155.88
147.08
144.56
139.93
134.13
124.21
118.77
116.40
113.34
110.92
55.65
40.15
39.94
39.73
39.52
39.31
39.10
38.89
125.86
OPy PyO
S
MeO
OMe
Br Br
f1 (ppm)

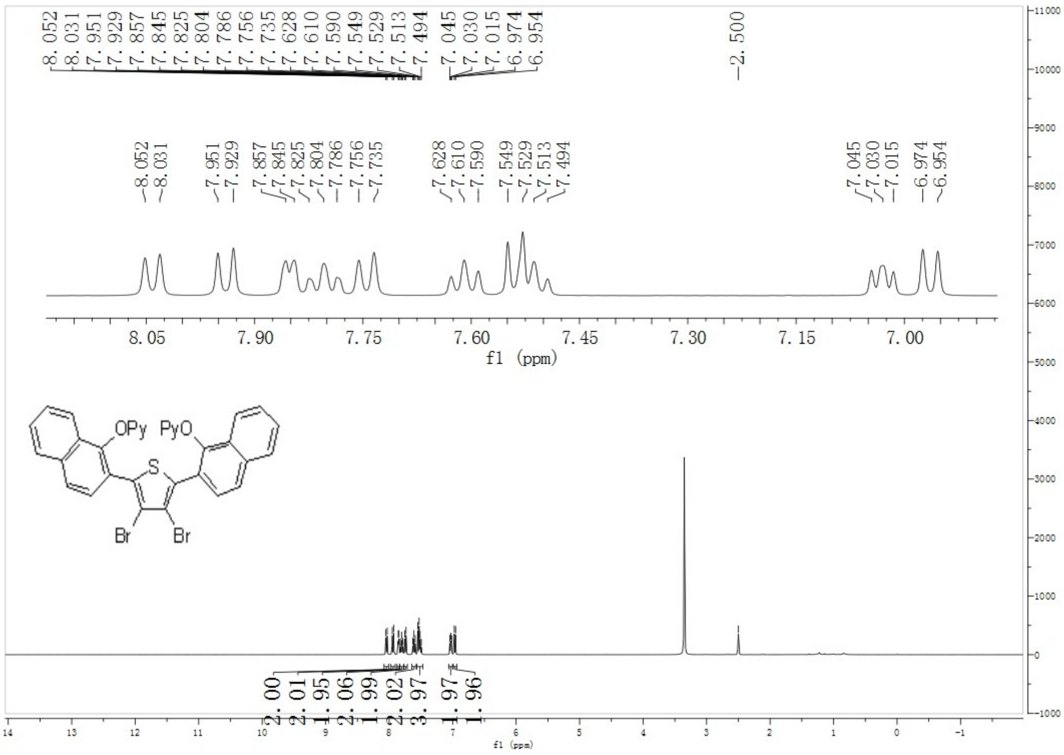
8.052 8.031 7.951 7.929 7.857 7.845 7.825 7.804 7.786 7.756 7.735 7.628 7.610 7.590 7.549 7.529 7.513 7.494 7.045 7.030 7.015 6.974 6.954
2.500
2.00 2.01 1.95 2.06 1.99 2.02 3.97 1.97 1.96
f1 (ppm)
OPy PyO
S
Br Br

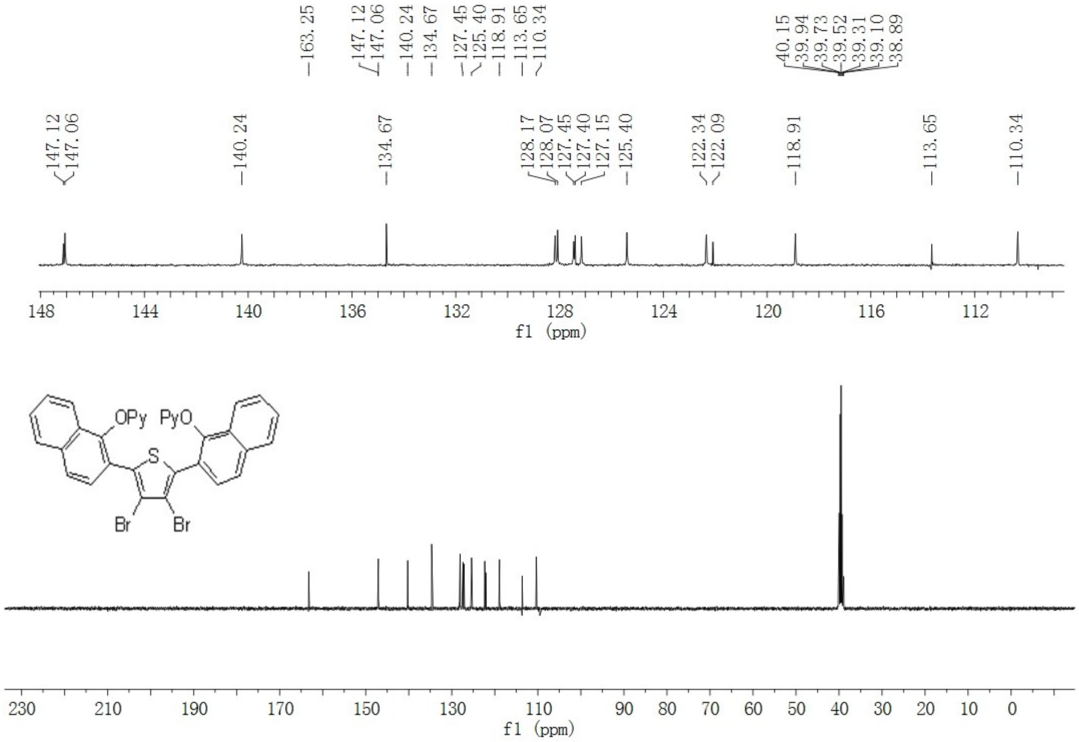
163.25 147.12 147.06 140.24 134.67 127.45 125.40 118.91 113.65 110.34
40.15 39.94 39.73 39.52 39.31 39.10 38.89
128.17 128.07 127.45 127.40 127.15 125.40 122.34 122.09
f1 (ppm)
OPy PyO
S
Br Br

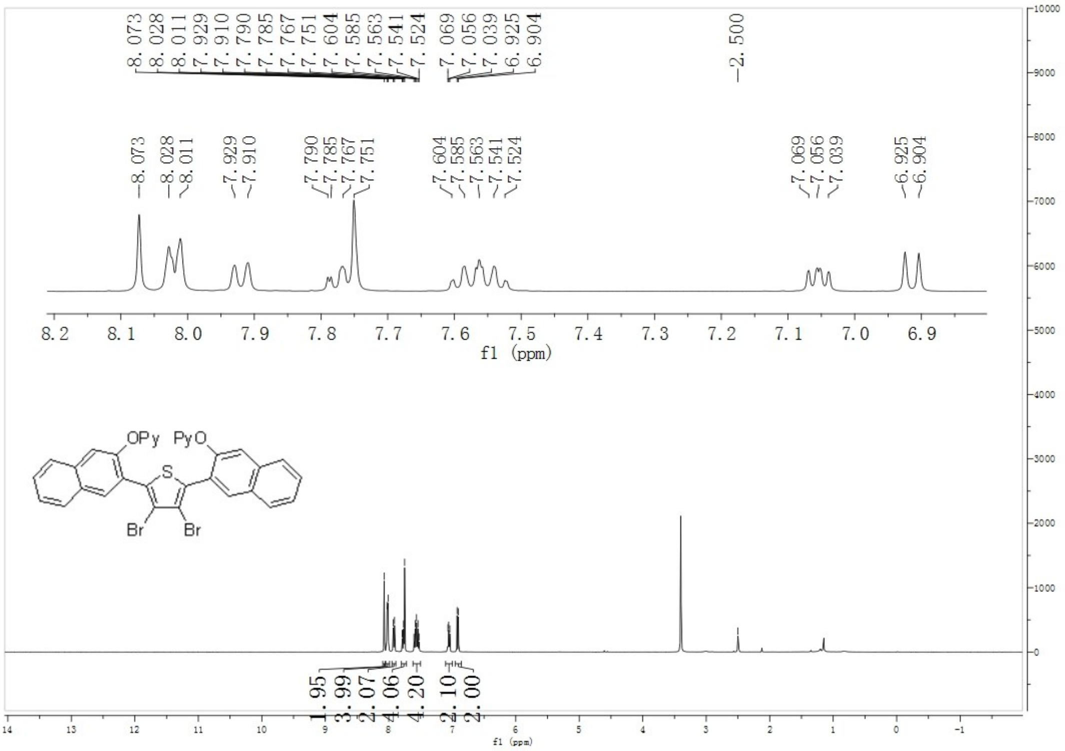

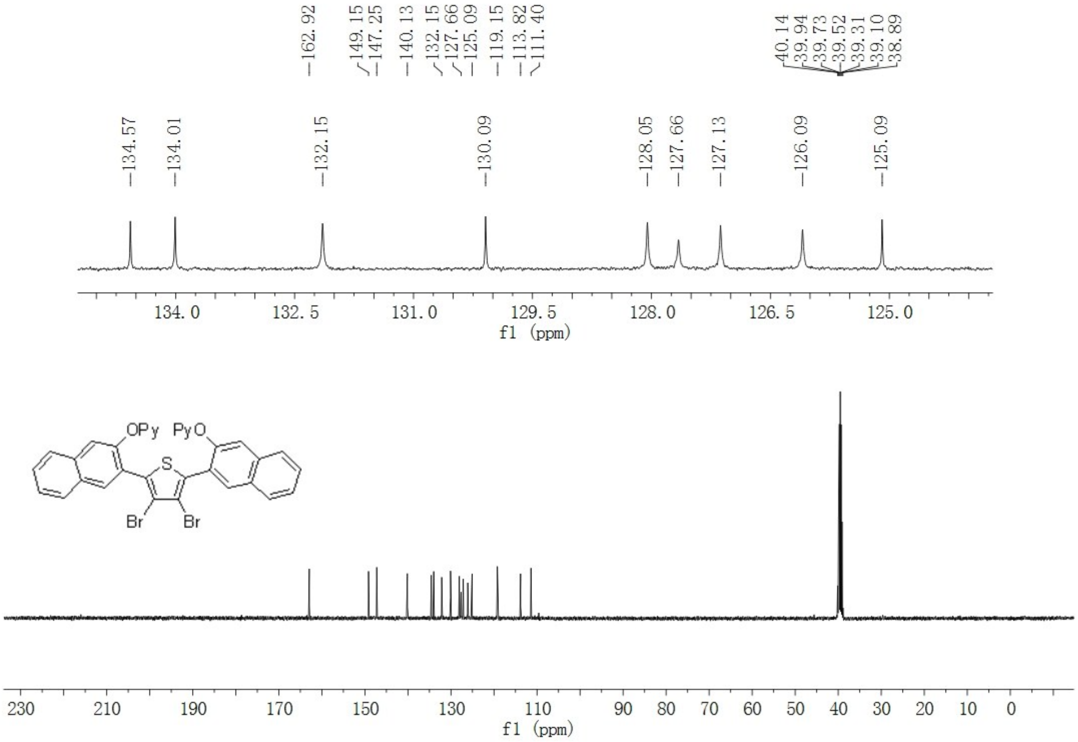

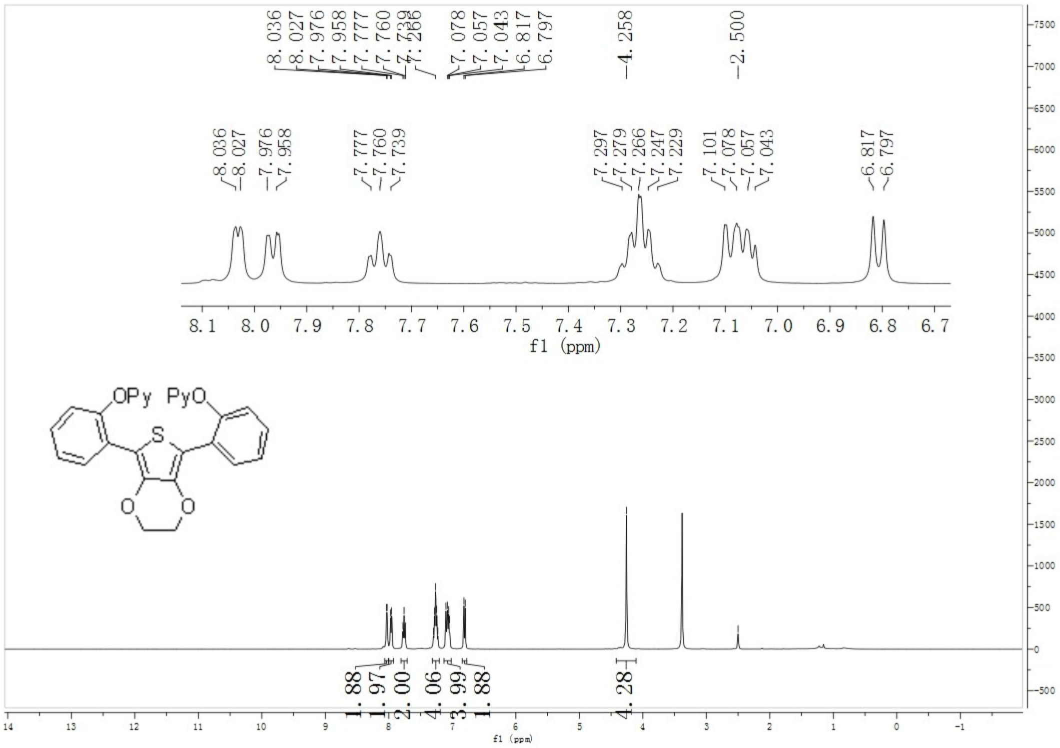
8.036
8.027
7.976
7.958
7.777
7.760
7.739
7.078
7.057
7.043
6.817
6.797
4.258
2.500
7.297
7.279
7.266
7.247
7.229
7.101
f1 (ppm)
OPy PyO
S
O O
1.88
1.97
2.00
4.06
3.99
1.88
4.28

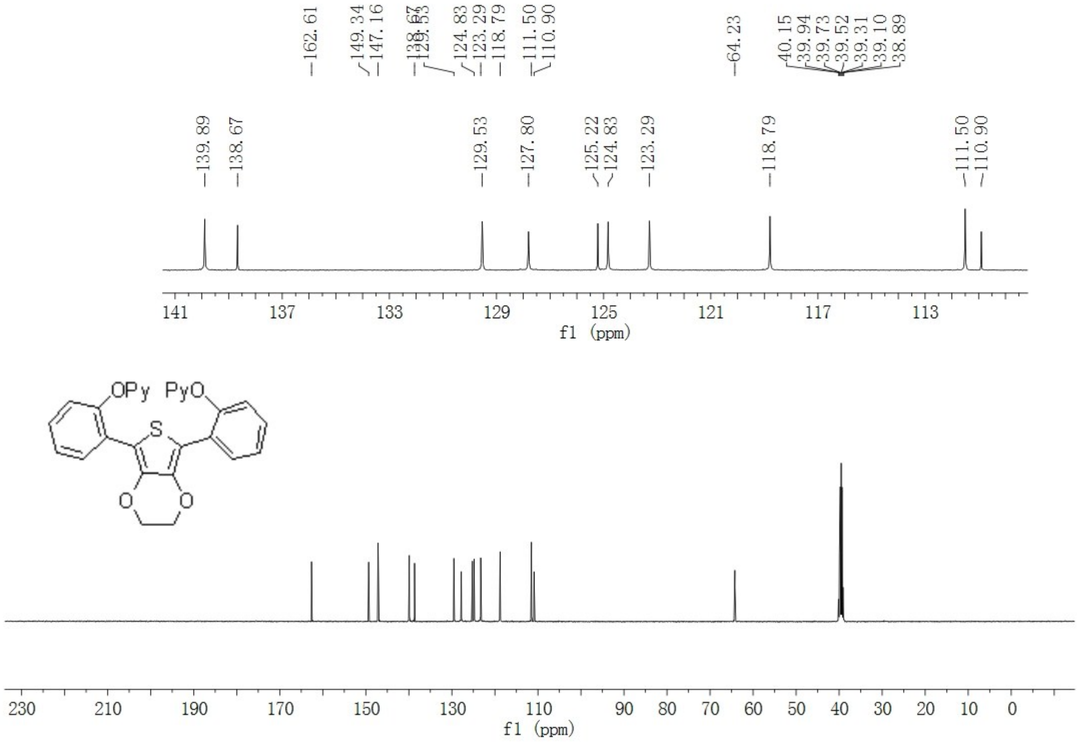
162.61
149.34
147.16
124.83
123.29
118.79
111.50
110.90
64.23
40.15
39.94
39.73
39.52
39.31
39.10
38.89
139.89
138.67
129.53
127.80
125.22
124.83
123.29
118.79
111.50
110.90
f1 (ppm)
OPy PyO
S
O O

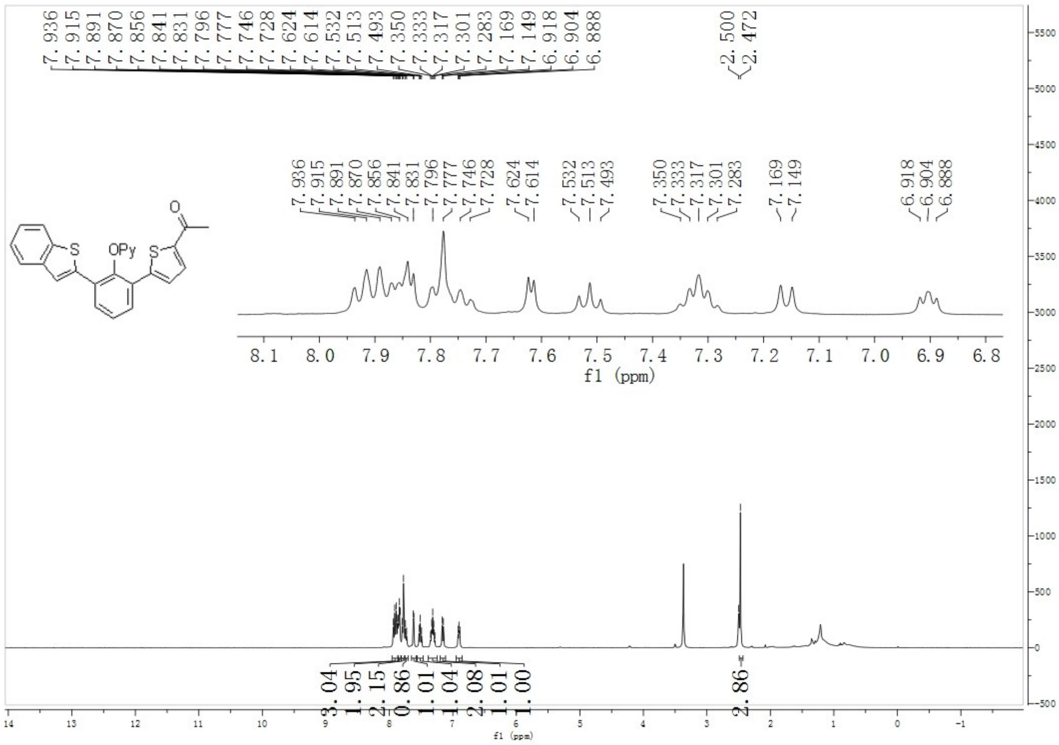

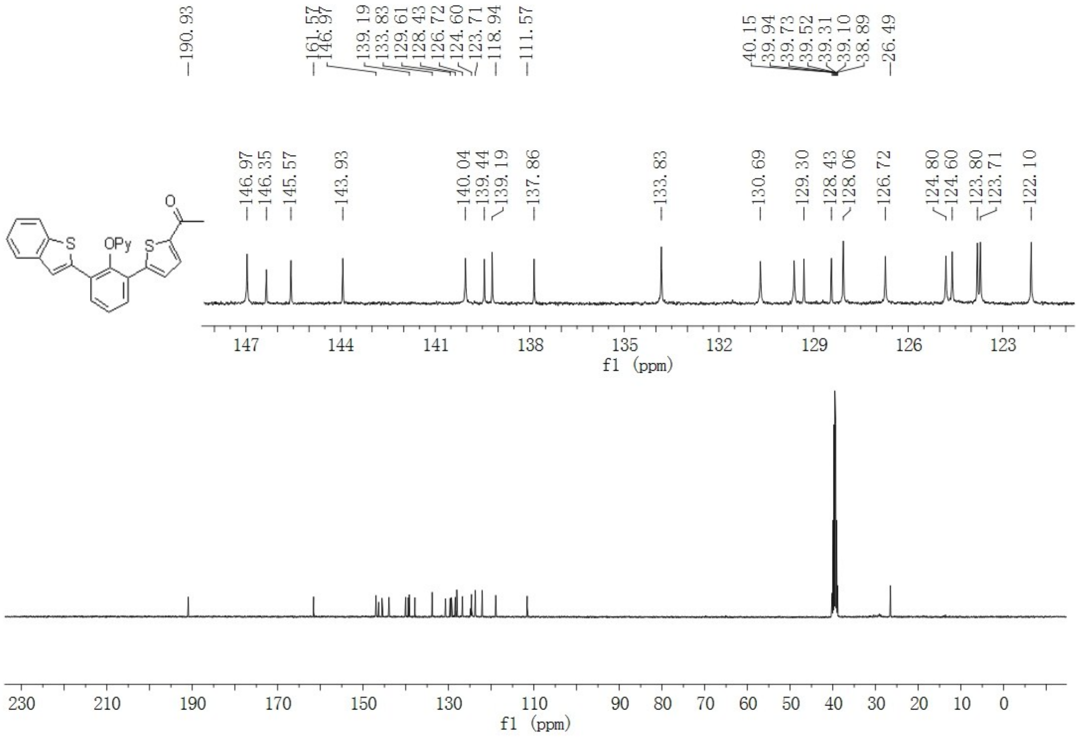

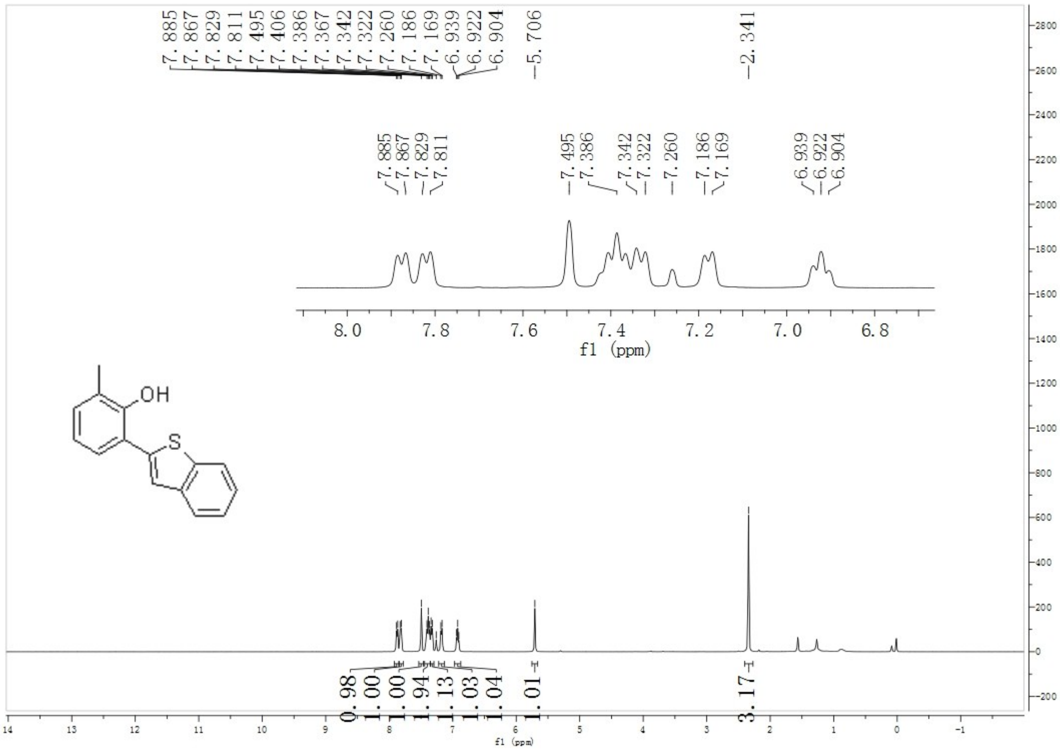
7.885
7.867
7.829
7.811
7.495
7.406
7.386
7.367
7.342
7.322
7.260
7.186
7.169
6.939
6.922
6.904
5.706
2.341
OH
S
f1 (ppm)
0.98
1.00
1.00
1.94
1.13
1.03
1.04
1.01
3.17

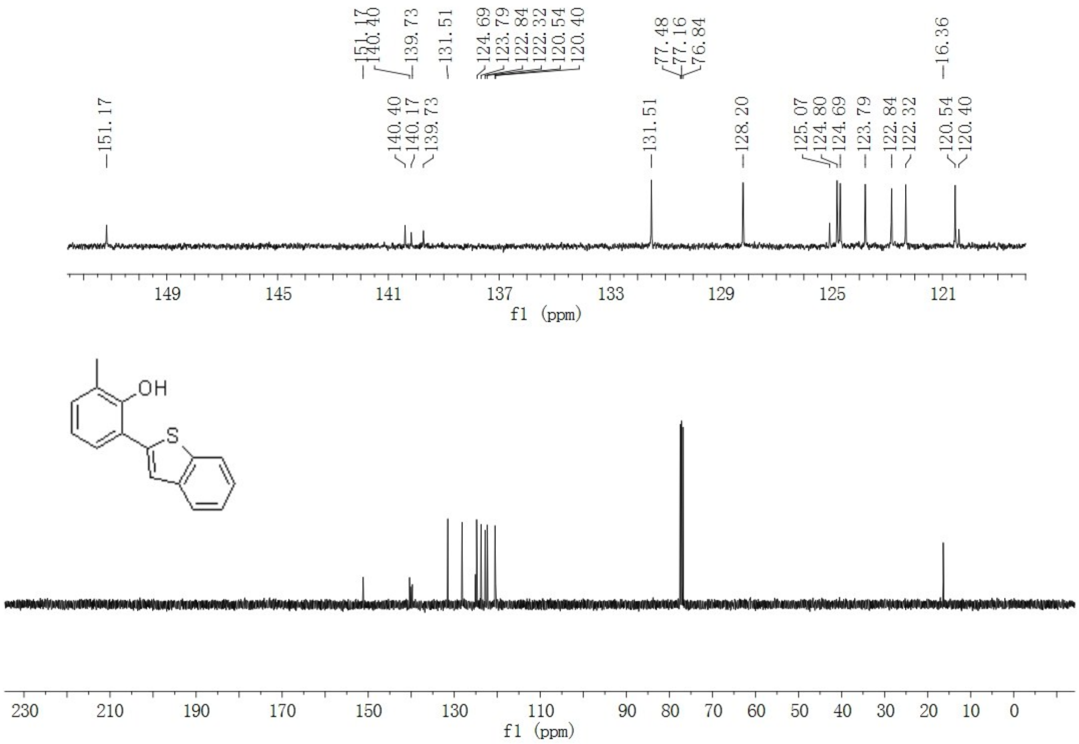
151.17
140.40
139.73
131.51
124.69
123.79
122.84
122.32
120.54
120.40
77.48
77.16
76.84
16.36
140.17
128.20
125.07
124.80
f1 (ppm)
OH
S

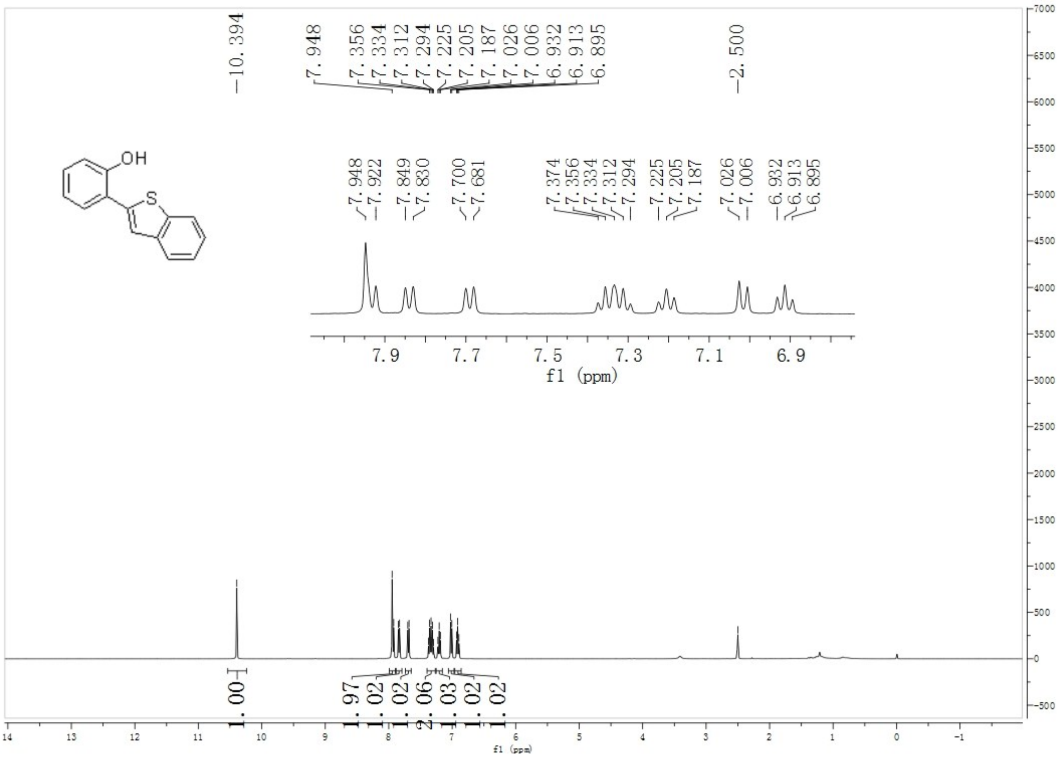
OH
S
10.394
7.948
7.356
7.334
7.312
7.294
7.225
7.205
7.187
7.026
7.006
6.932
6.913
6.895
2.500
7.948
7.922
7.849
7.830
7.700
7.681
7.374
7.356
7.334
7.312
7.294
7.225
7.205
7.187
7.026
7.006
6.932
6.913
6.895
f1 (ppm)
1.00
0.97
1.02
1.02
2.06
1.03
1.02
1.02
f1 (ppm)

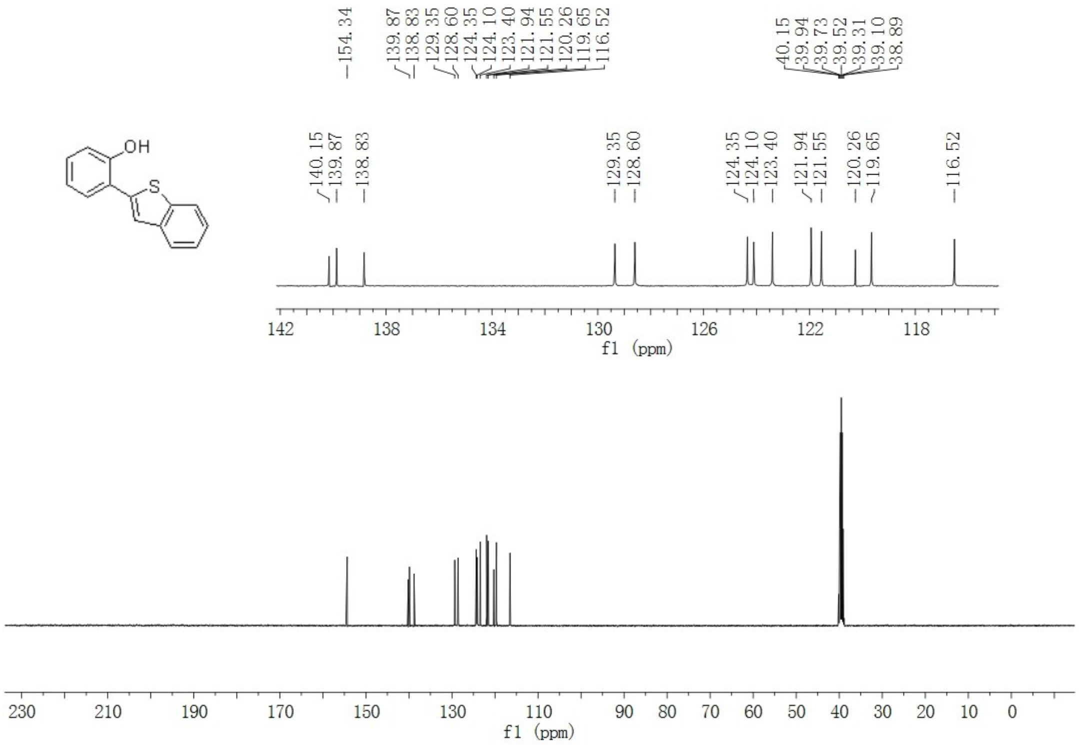
OH
S
154.34
139.87
138.83
129.35
128.60
124.35
124.10
123.40
121.94
121.55
120.26
119.65
116.52
40.15
39.94
39.73
39.52
39.31
39.10
38.89
140.15
139.87
138.83
129.35
128.60
124.35
124.10
123.40
121.94
121.55
120.26
119.65
116.52
f1 (ppm)
f1 (ppm)

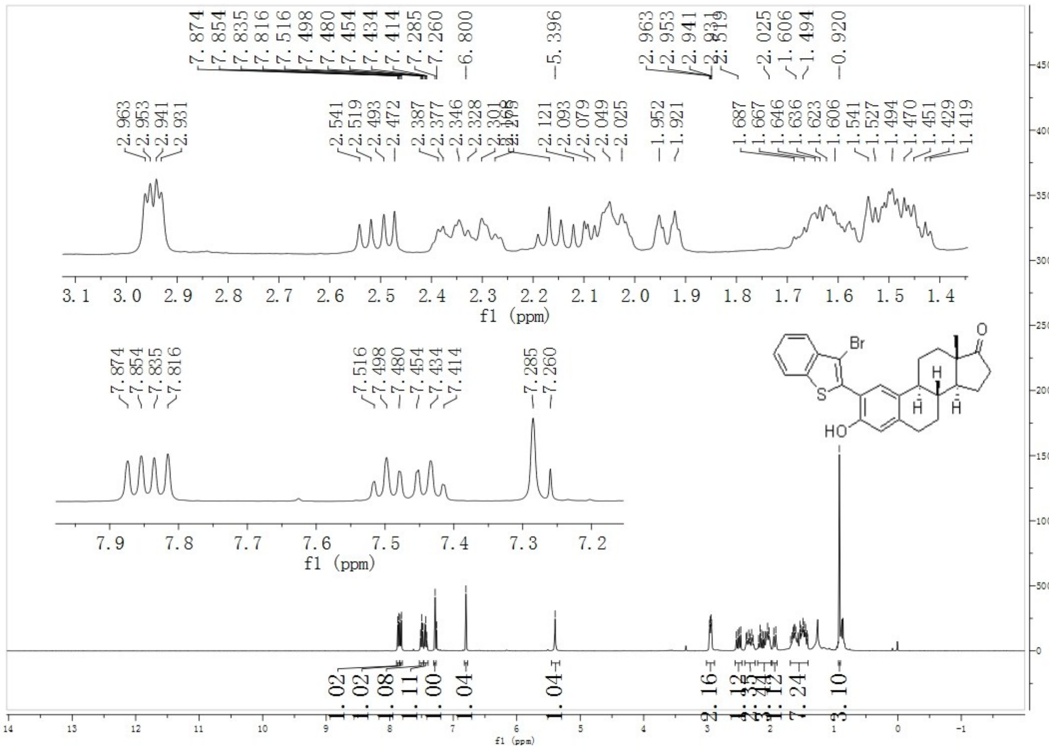

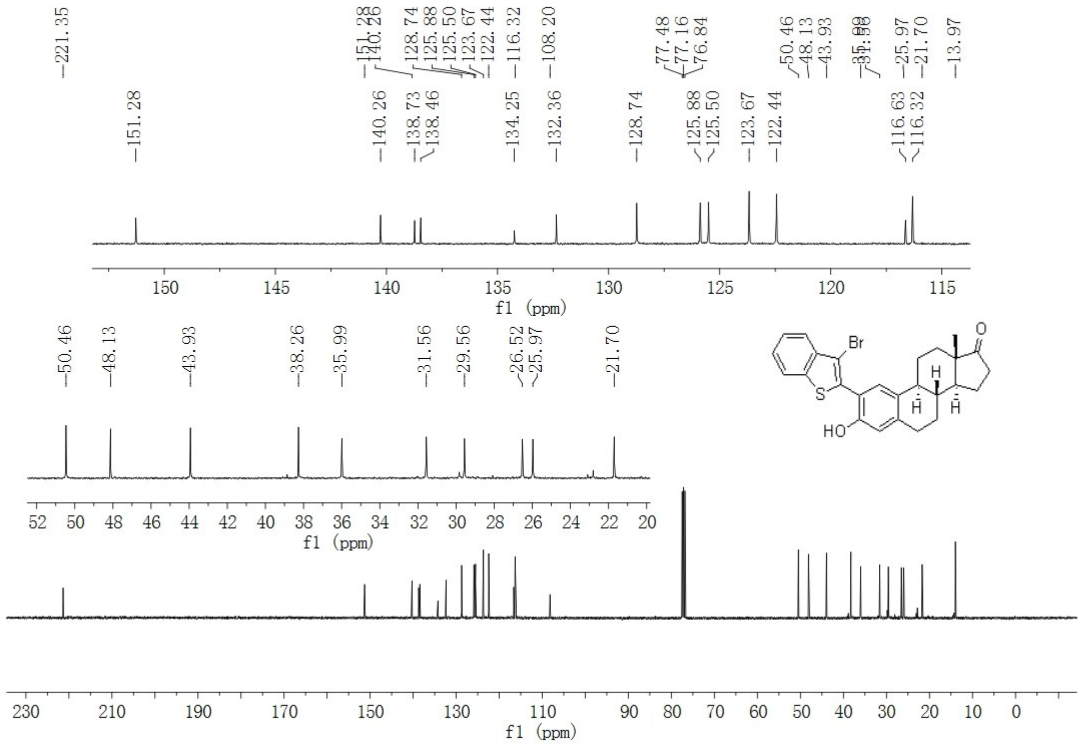

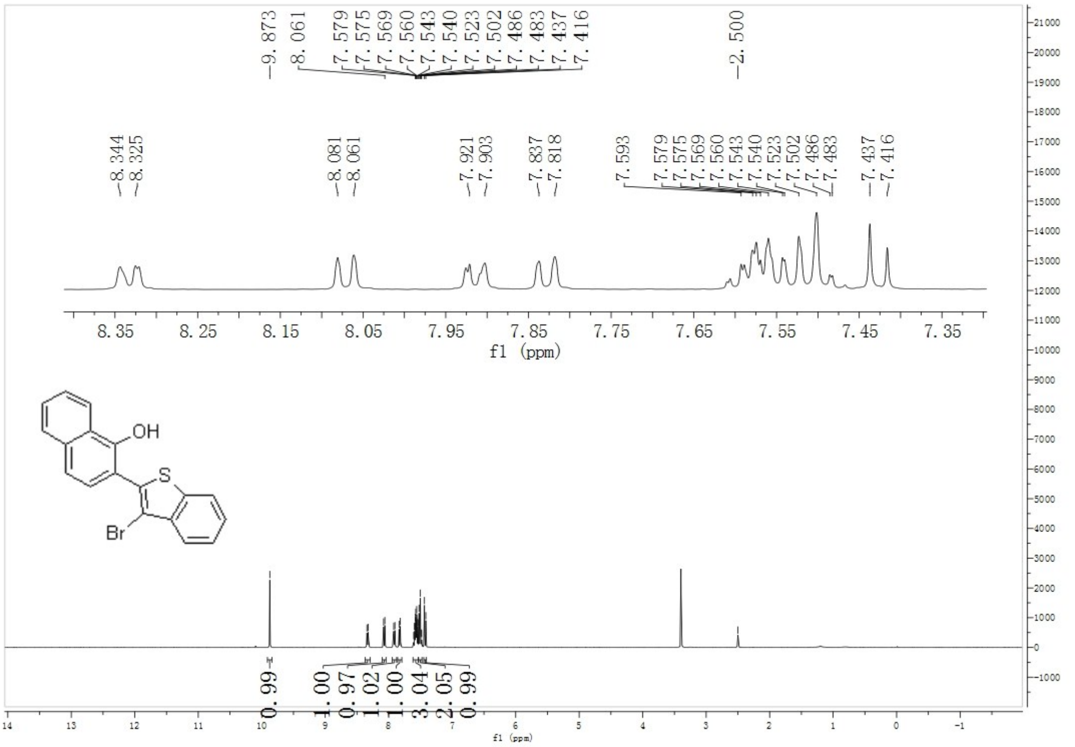

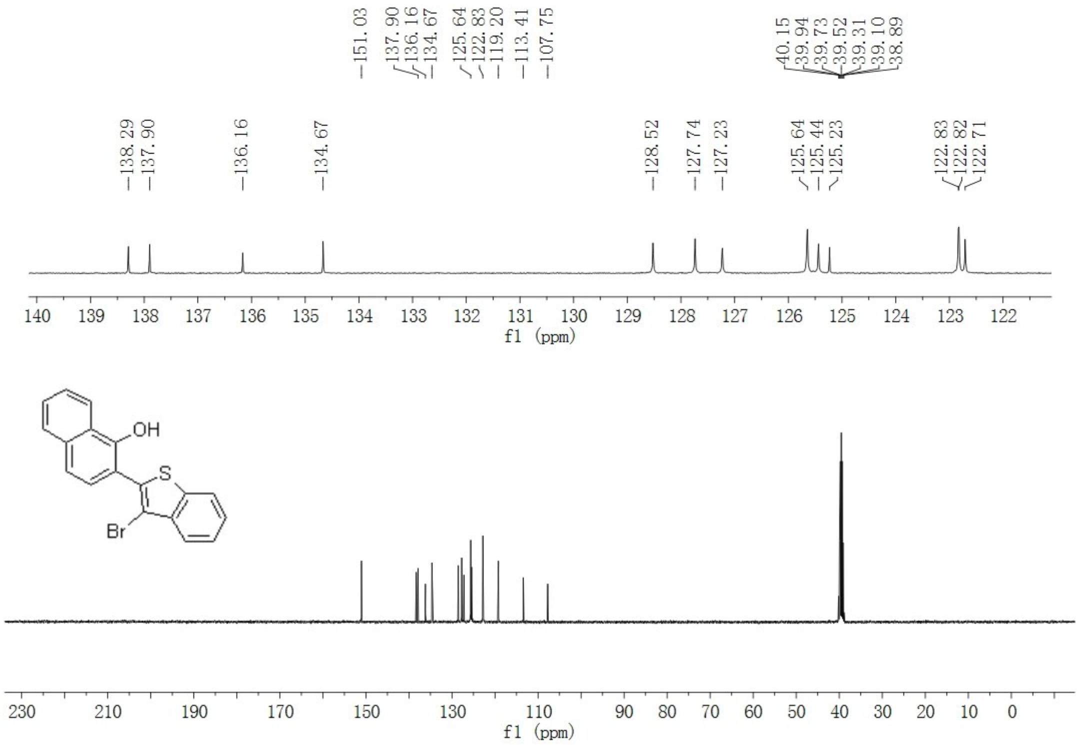

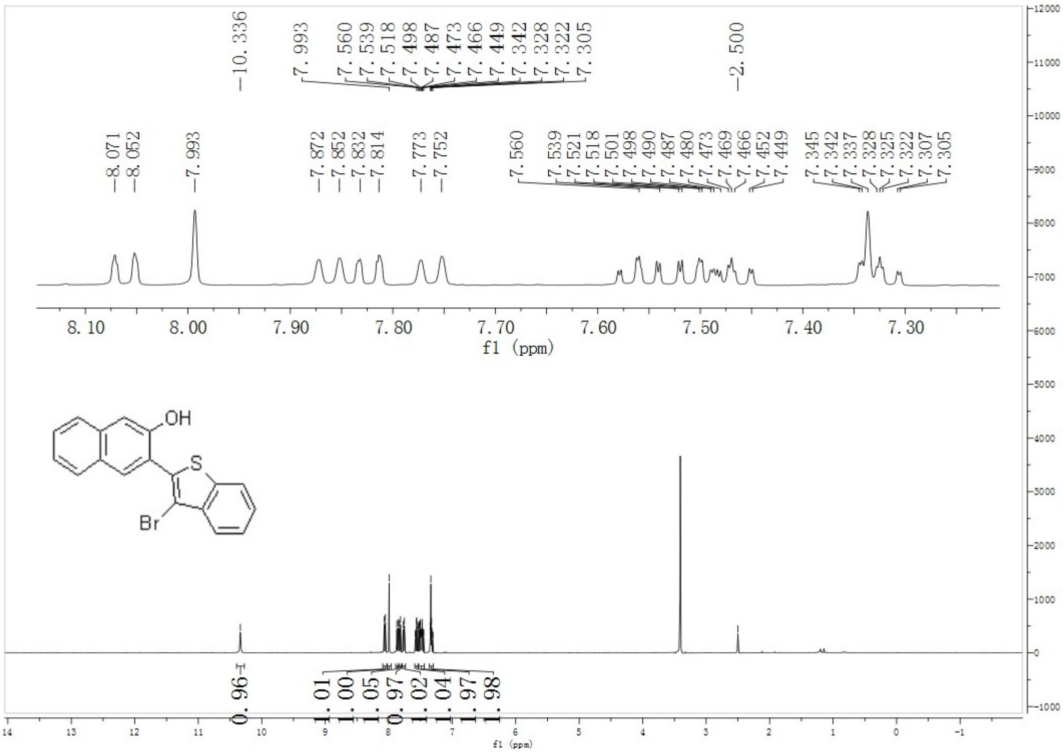
10.336
7.993
7.560
7.539
7.518
7.498
7.487
7.473
7.466
7.449
7.342
7.328
7.322
7.305
2.500
8.071
8.052
7.872
7.852
7.832
7.814
7.773
7.752
7.521
7.501
7.490
7.480
7.469
7.452
7.345
7.337
7.325
7.307
f1 (ppm)
OH
S
Br
0.96
1.01
1.00
1.05
0.97
1.02
1.04
1.97
1.98

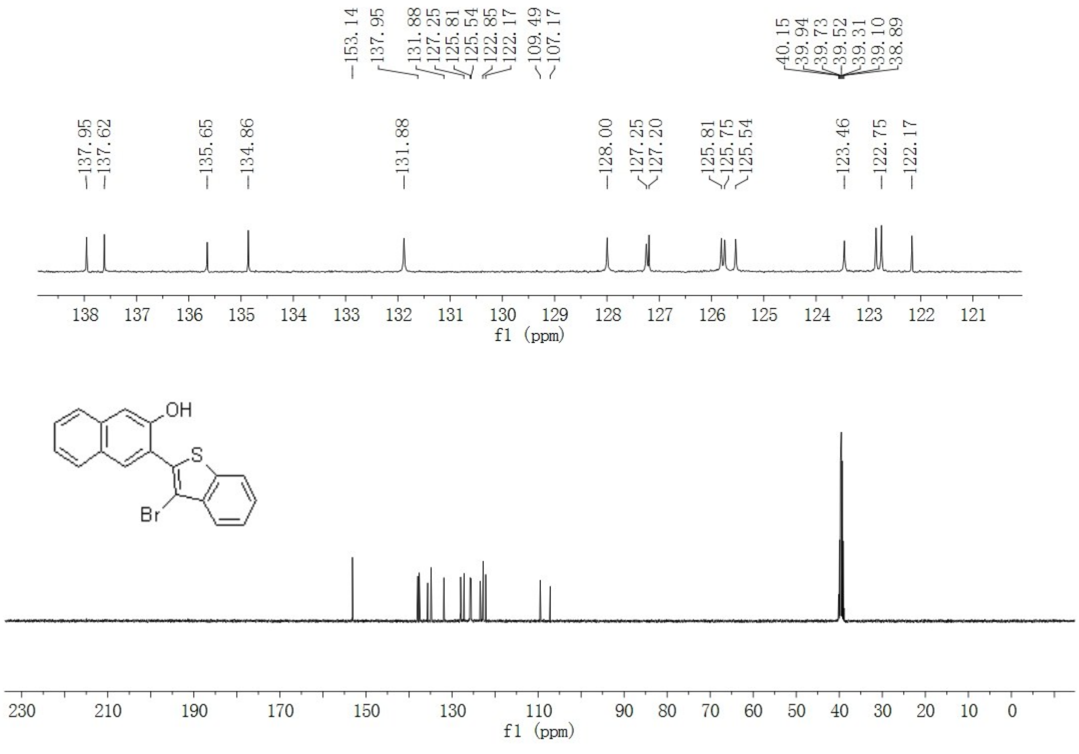
153.14
137.95
131.88
127.25
125.81
125.54
122.85
122.17
109.49
107.17
40.15
39.94
39.73
39.52
39.31
39.10
38.89
137.62
135.65
134.86
128.00
127.20
125.75
123.46
122.75
f1 (ppm)
OH
S
Br

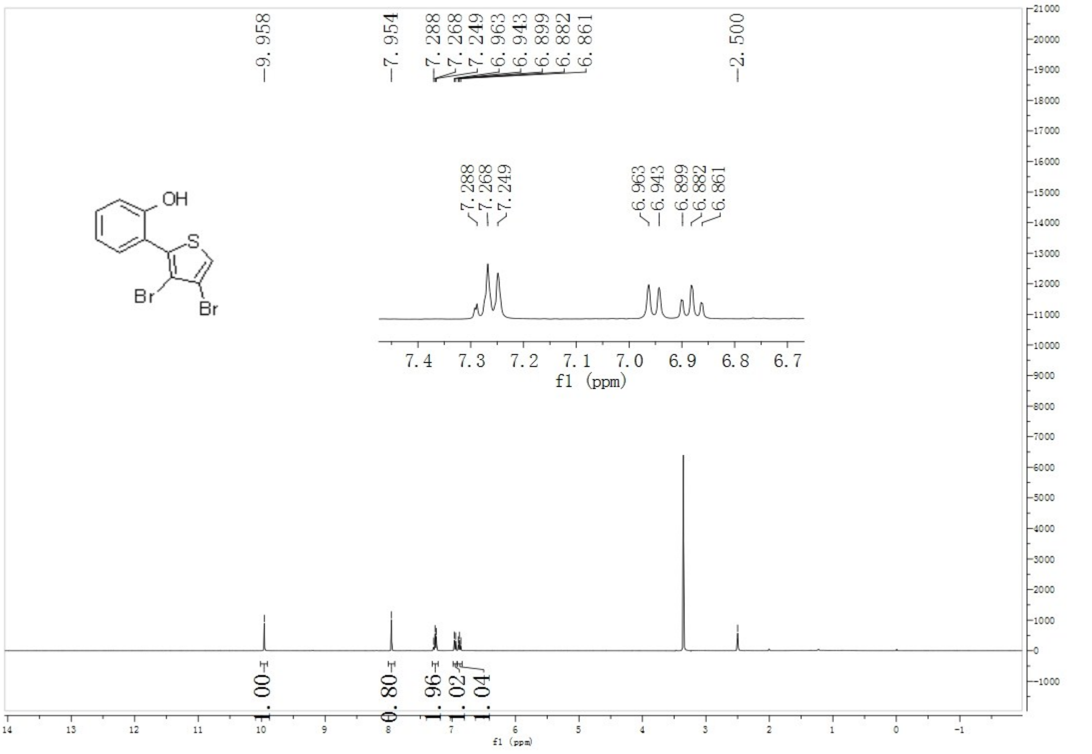
9.958
7.954
7.288
7.268
7.249
6.963
6.943
6.899
6.882
6.861
2.500
OH
S
Br
Br
1.00
0.80
1.96
1.02
1.04
f1 (ppm)

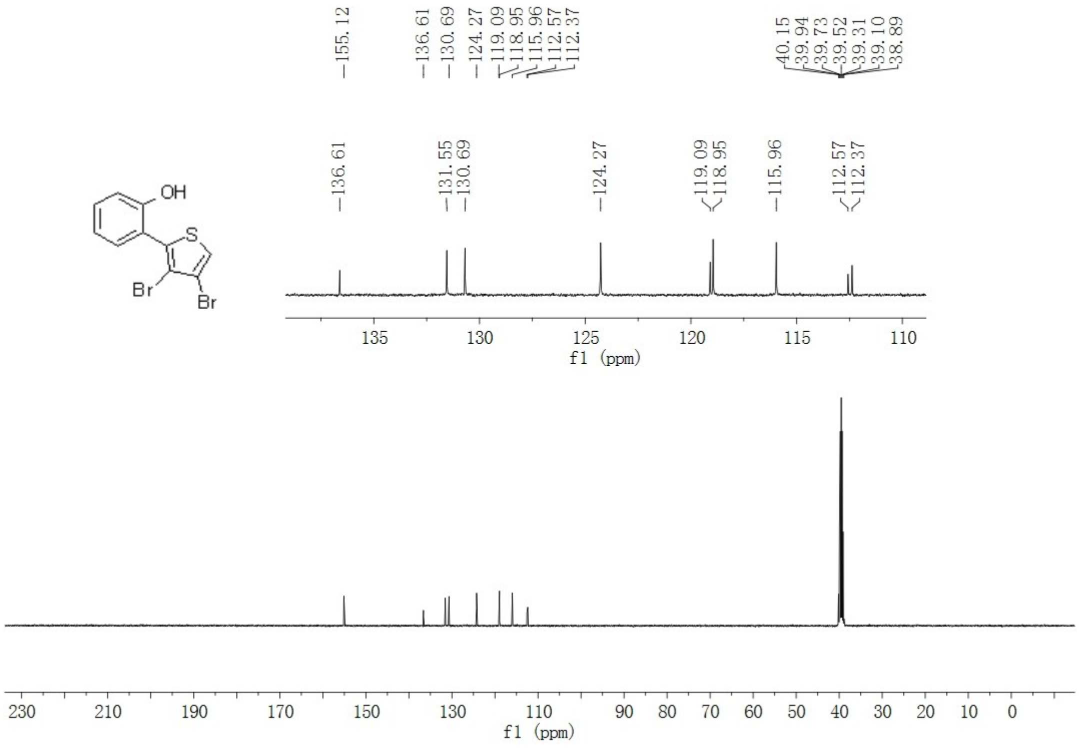
155.12
136.61
130.69
124.27
119.09
118.95
115.96
112.57
112.37
40.15
39.94
39.73
39.52
39.31
39.10
38.89
131.55
OH
S
Br
Br
f1 (ppm)

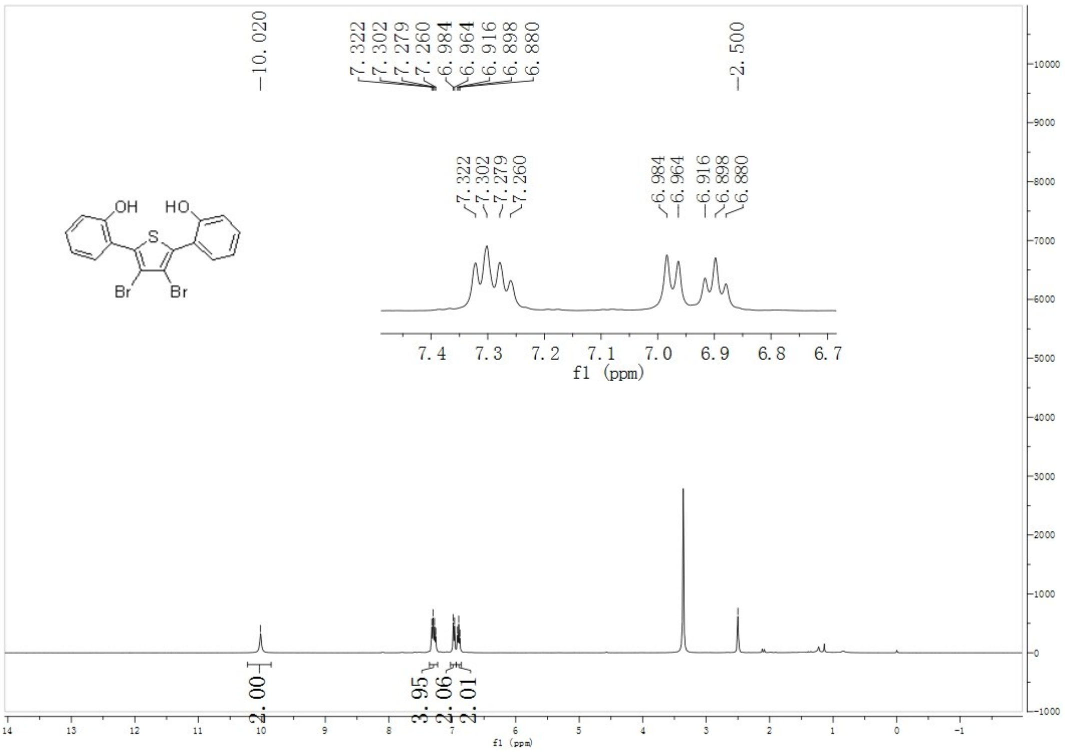
10.020
7.322
7.302
7.279
7.260
6.984
6.964
6.916
6.898
6.880
2.500
OH
HO
S
Br
Br
2.00
3.95
2.06
2.01
f1 (ppm)

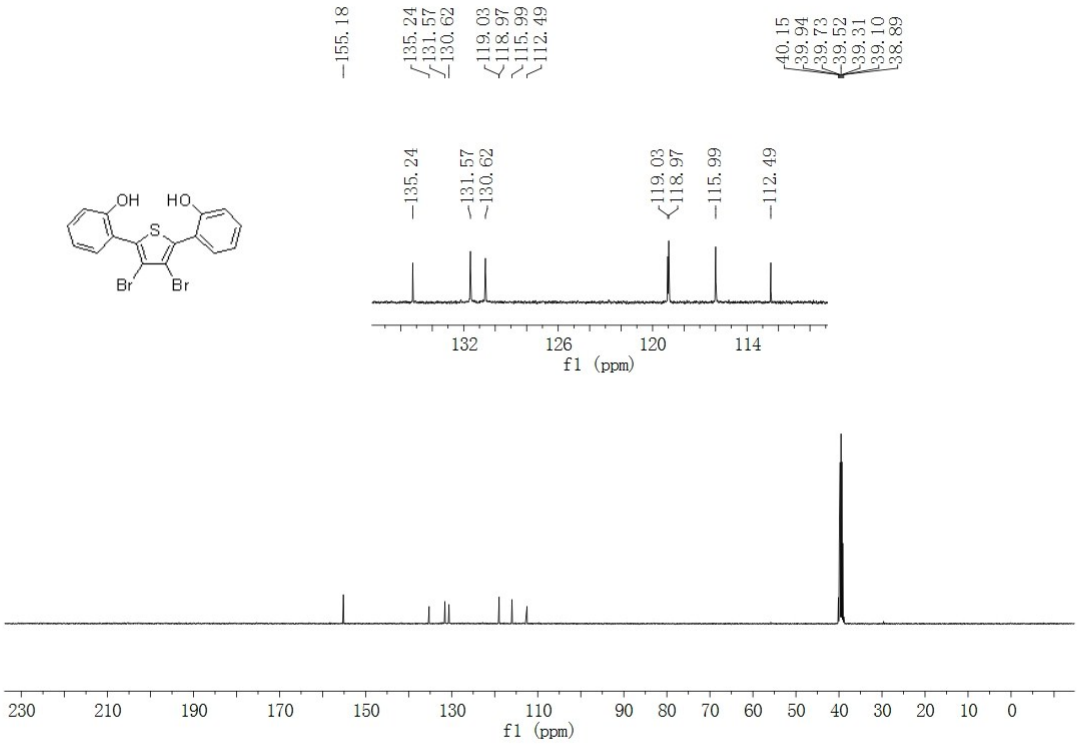
155.18
135.24
131.57
130.62
119.03
118.97
115.99
112.49
40.15
39.94
39.73
39.52
39.31
39.10
38.89
OH
HO
S
Br
Br
f1 (ppm)

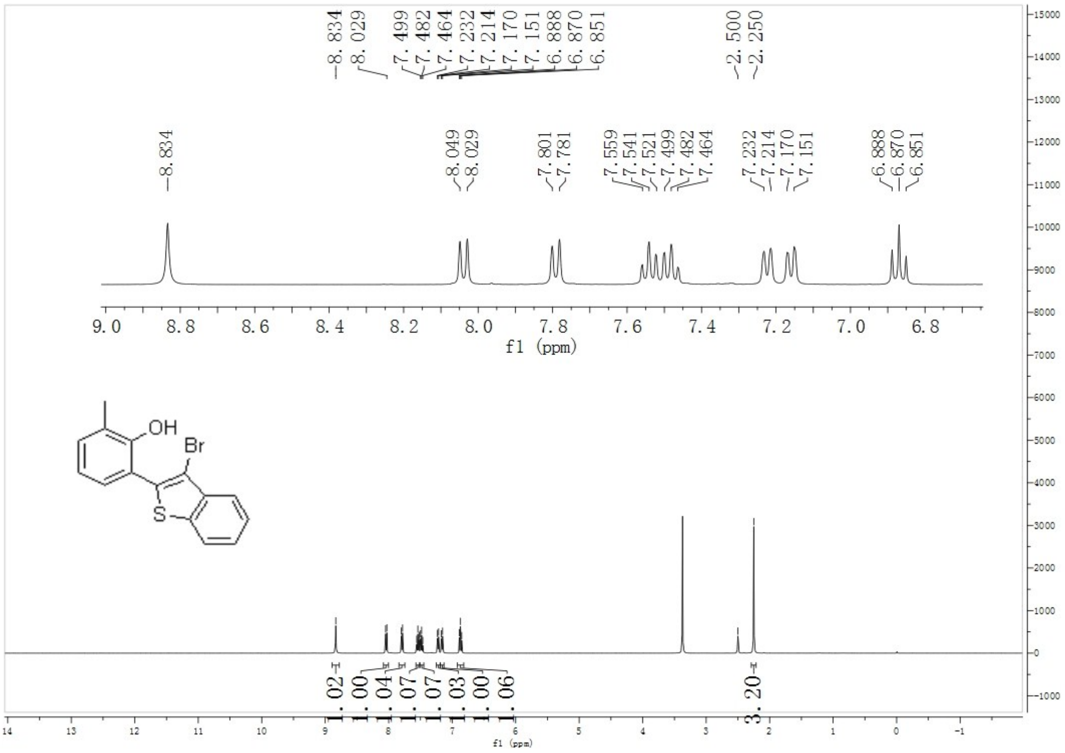

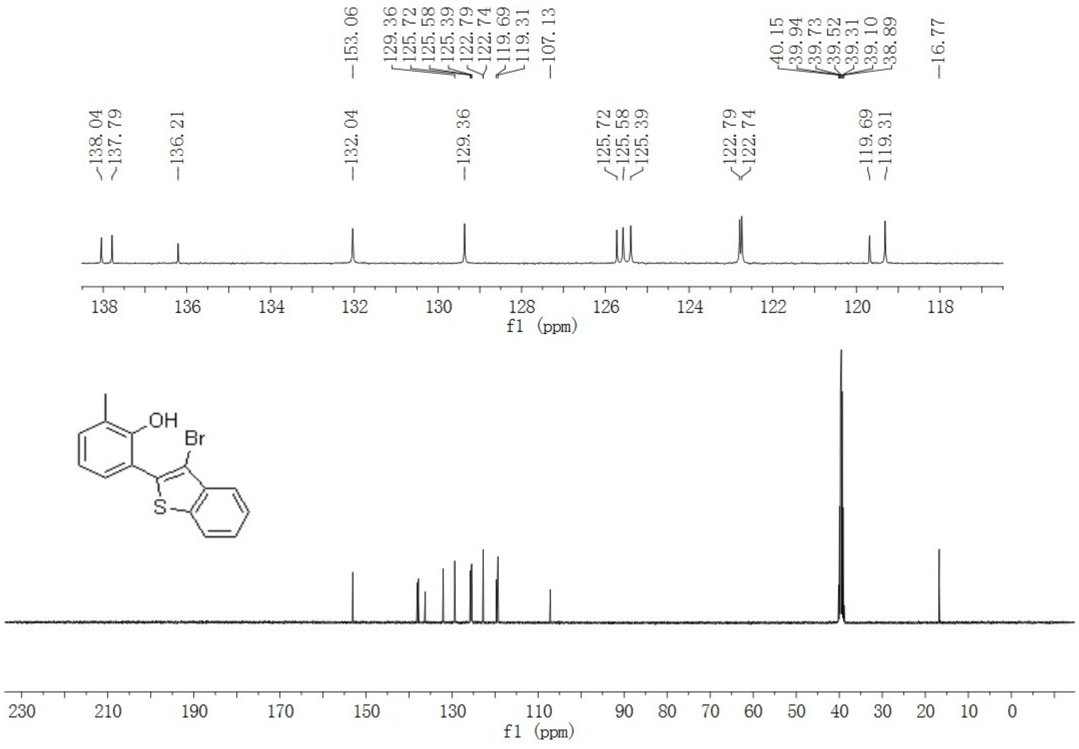

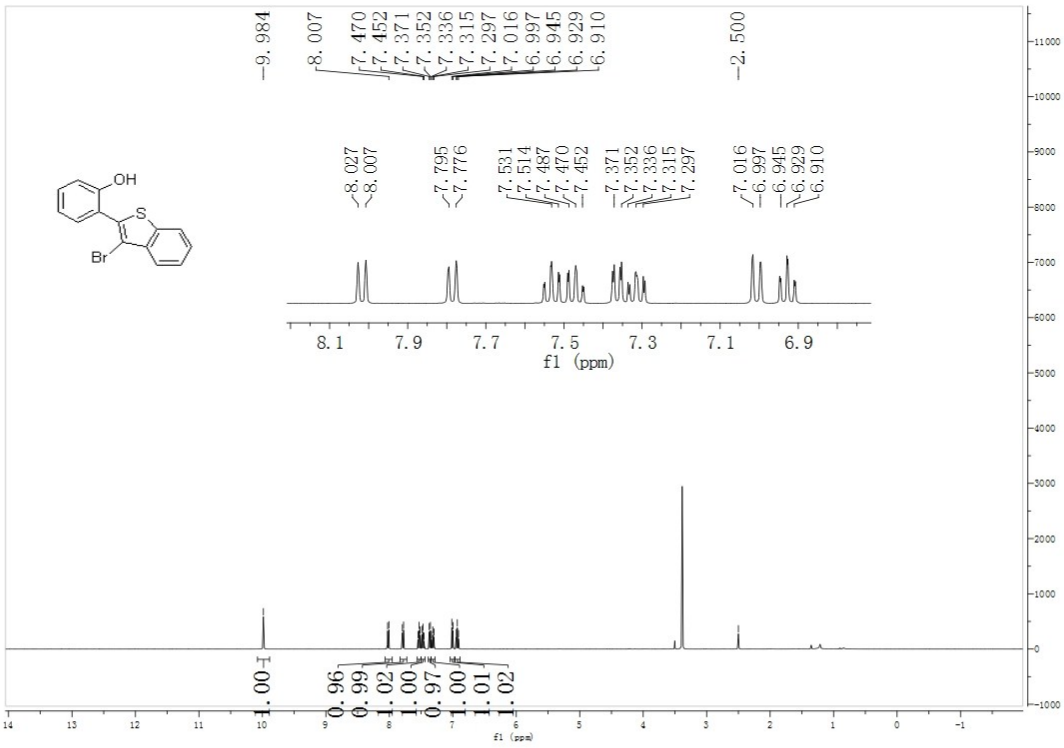
9.984
8.007
7.470
7.452
7.371
7.352
7.336
7.315
7.297
7.016
6.997
6.945
6.929
6.910
2.500
8.027
8.007
7.795
7.776
7.531
7.514
7.487
7.470
7.452
7.371
7.352
7.336
7.315
7.297
7.016
6.997
6.945
6.929
6.910
OH
S
Br
f1 (ppm)
1.00
0.96
0.99
1.02
1.00
0.97
1.00
1.01
1.02

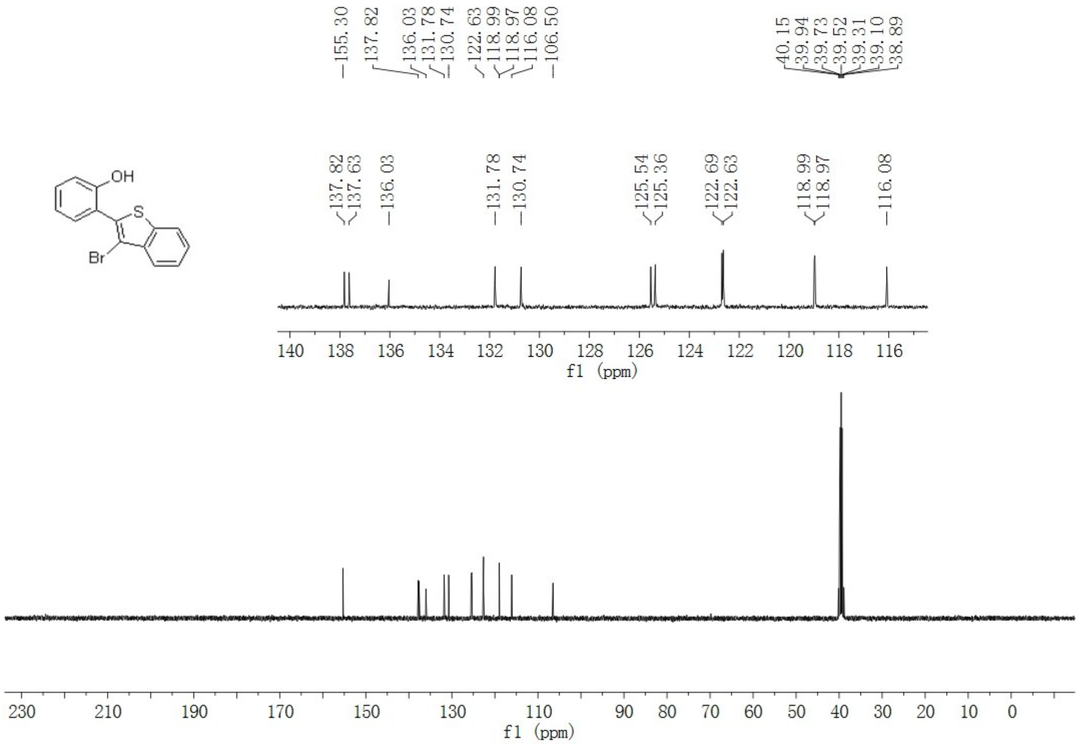
155.30
137.82
136.03
131.78
130.74
122.63
118.99
118.97
116.08
106.50
40.15
39.94
39.73
39.52
39.31
39.10
38.89
137.82
137.63
136.03
131.78
130.74
125.54
125.36
122.69
122.63
118.99
118.97
116.08
OH
S
Br
f1 (ppm)

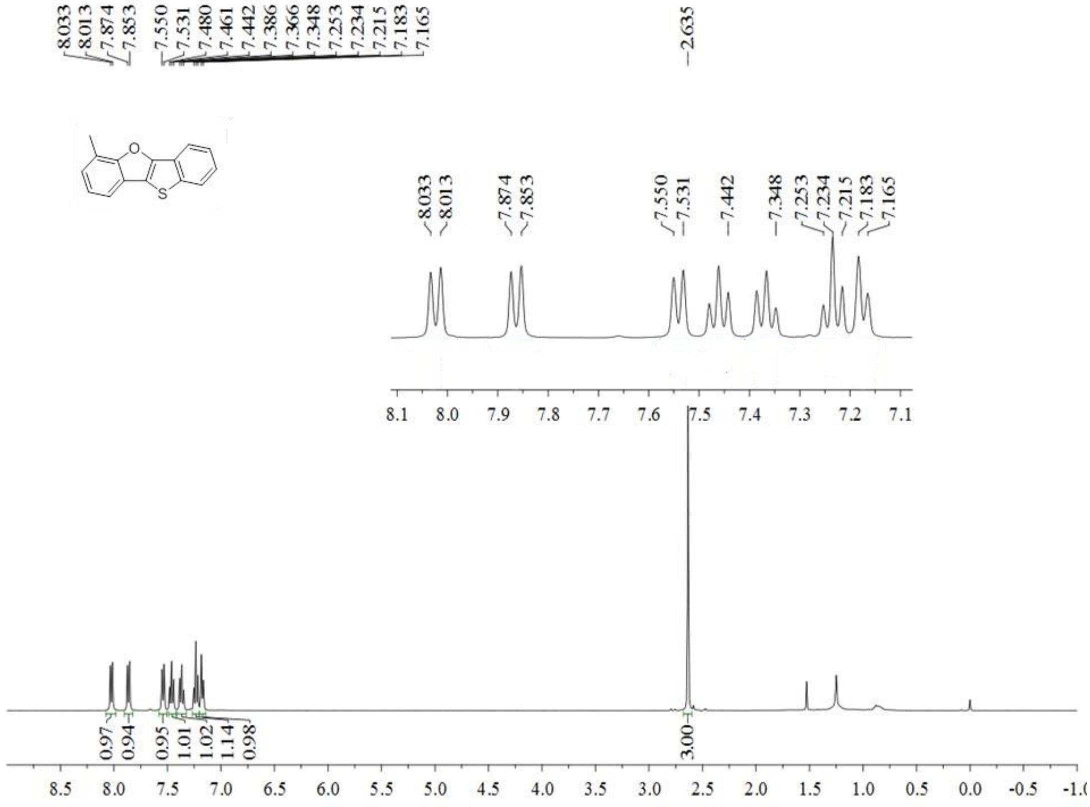

8.033
8.013
7.874
7.853
7.550
7.531
7.480
7.461
7.442
7.386
7.366
7.348
7.253
7.234
7.215
7.183
7.165
2.635
S
0.97
0.94
0.95
1.01
1.02
1.14
0.98
3.00

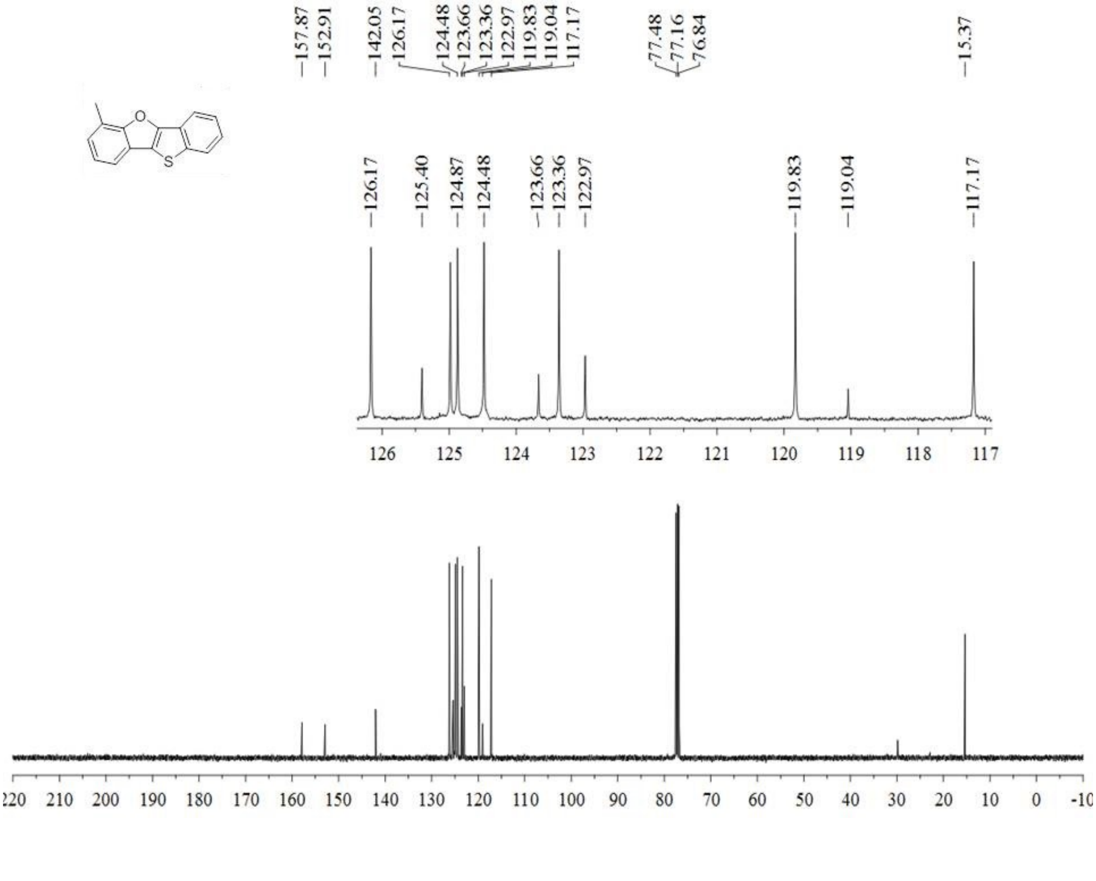

157.87
152.91
142.05
126.17
124.48
123.66
123.36
122.97
119.83
119.04
117.17
77.48
77.16
76.84
15.37
S
125.40
124.87

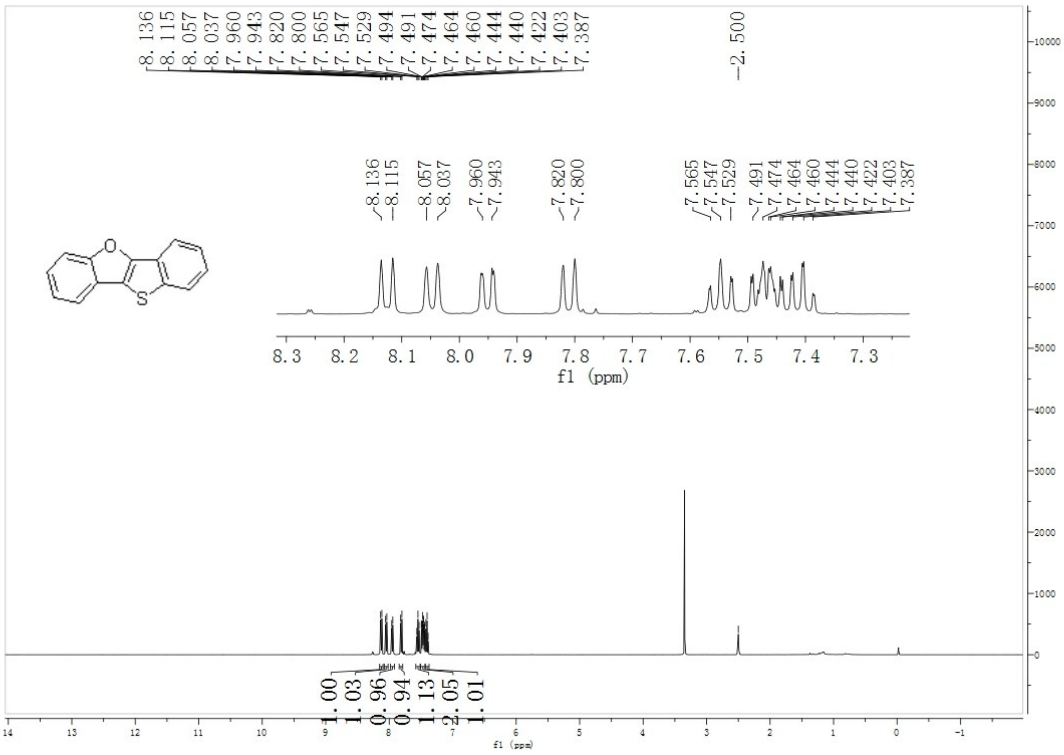
8.136 8.115 8.057 8.037 7.960 7.943 7.820 7.800 7.565 7.547 7.529 7.494 7.491 7.474 7.464 7.460 7.444 7.440 7.422 7.403 7.387
2.500
S
1.00 1.03 0.96 0.94 1.13 2.05 1.01
f1 (ppm)

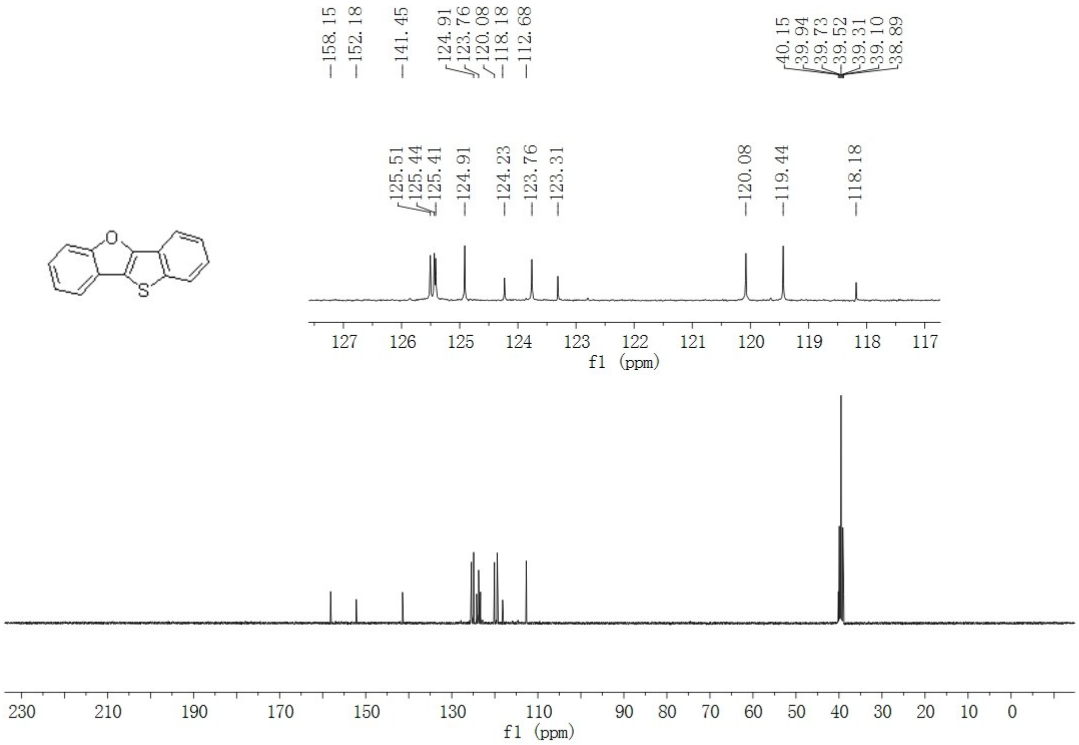
158.15 152.18 141.45 124.91 123.76 120.08 118.18 112.68
40.15 39.94 39.73 39.52 39.31 39.10 38.89
125.51 125.44 125.41 124.91 124.23 123.76 123.31 120.08 119.44 118.18
S
f1 (ppm)

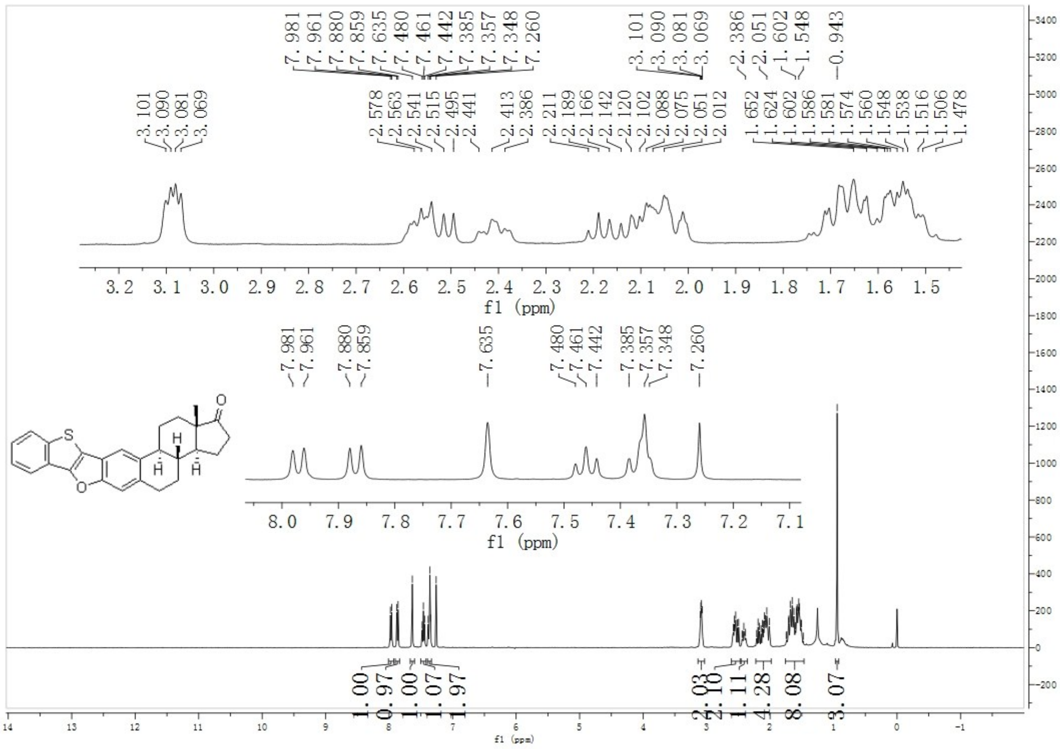

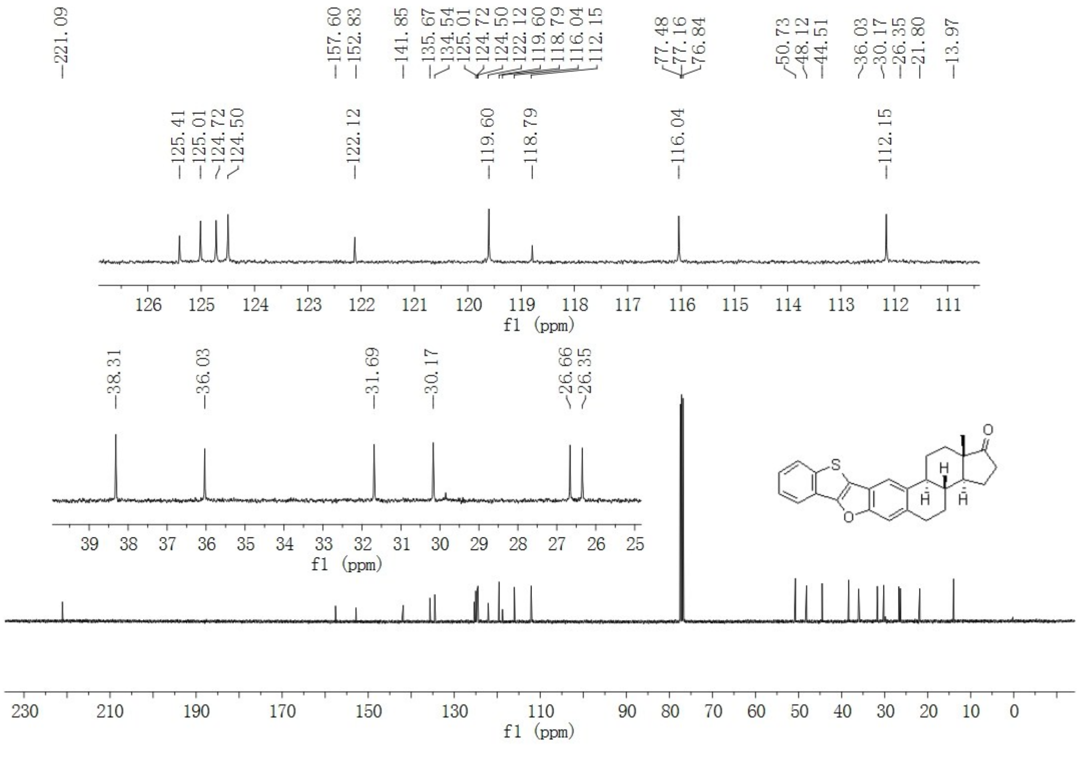

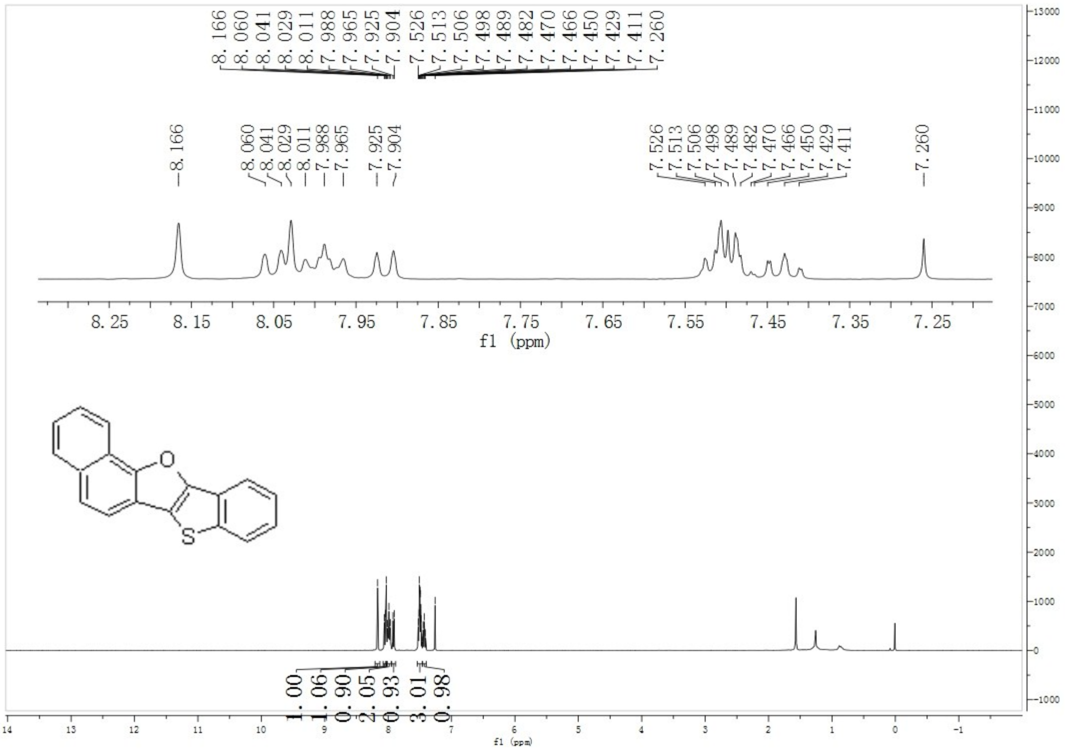
8.166 8.060 8.041 8.029 8.011 7.988 7.965 7.925 7.904 7.526 7.513 7.506 7.498 7.489 7.482 7.470 7.466 7.450 7.429 7.411 7.260
f1 (ppm)
1.00 1.06 0.90 2.05 0.93 3.01 0.98
S

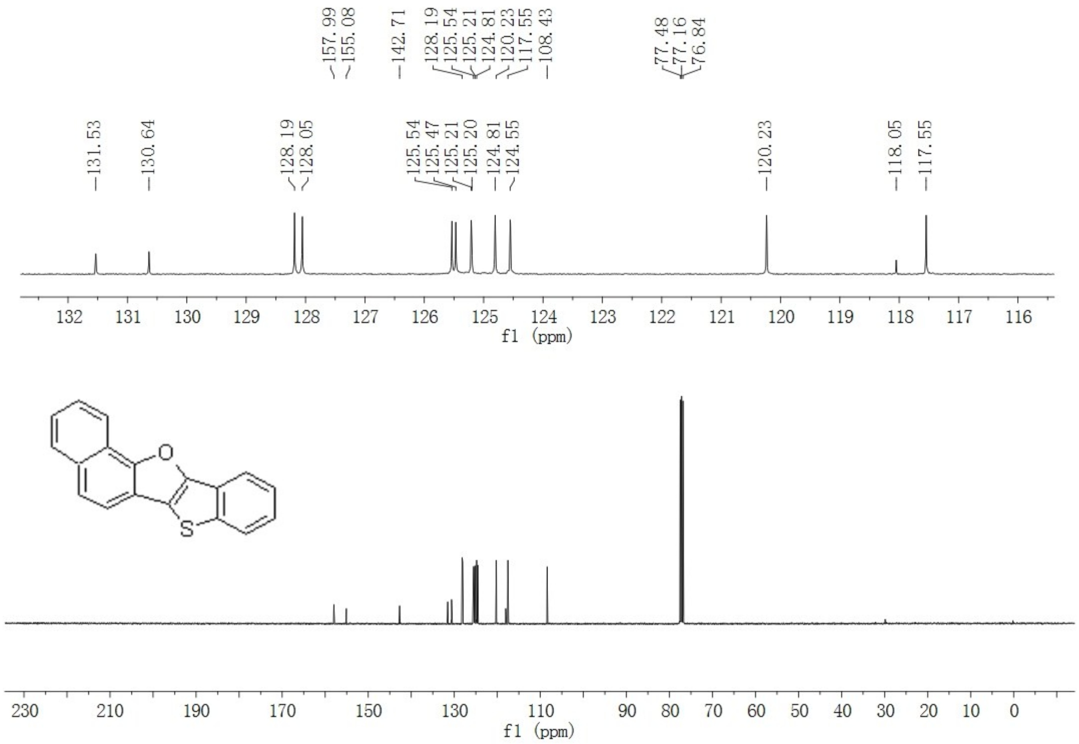
157.99 155.08 142.71 128.19 125.54 125.21 124.81 120.23 117.55 108.43 77.48 77.16 76.84
131.53 130.64 128.19 128.05 125.54 125.47 125.21 125.20 124.81 124.55 120.23 118.05 117.55
f1 (ppm)
S

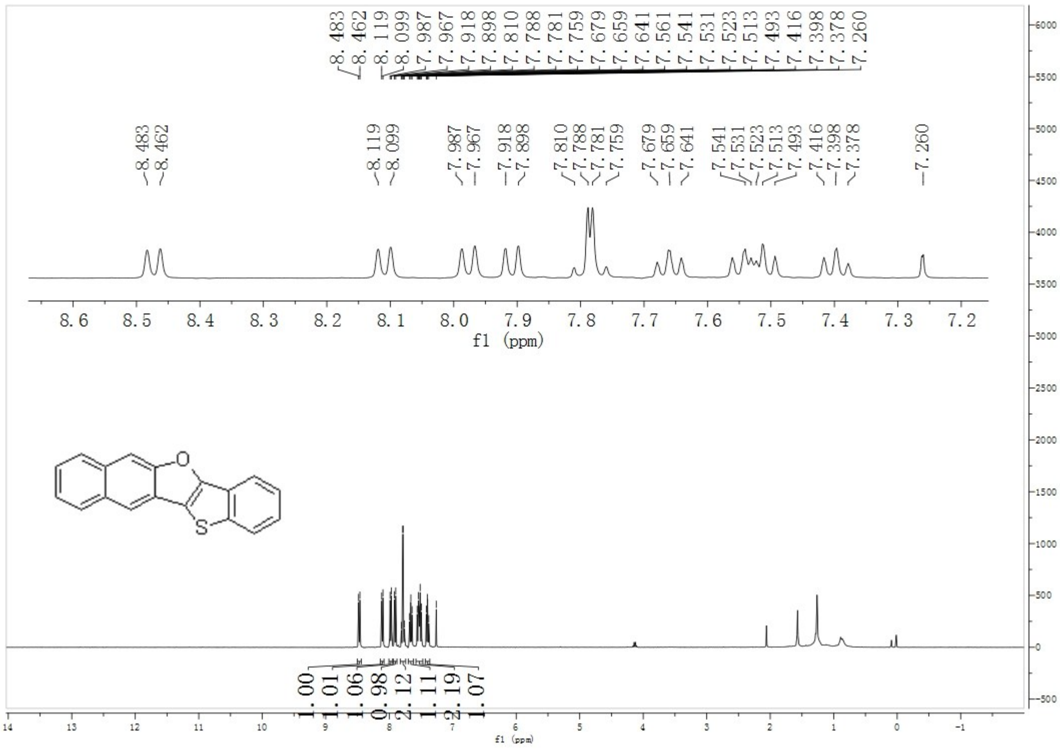
8.483
8.462
8.119
8.099
7.987
7.967
7.918
7.898
7.810
7.788
7.781
7.759
7.679
7.659
7.641
7.561
7.541
7.531
7.523
7.513
7.493
7.416
7.398
7.378
7.260
f1 (ppm)
S
O
1.00
1.01
1.06
0.98
2.12
1.11
2.19
1.07

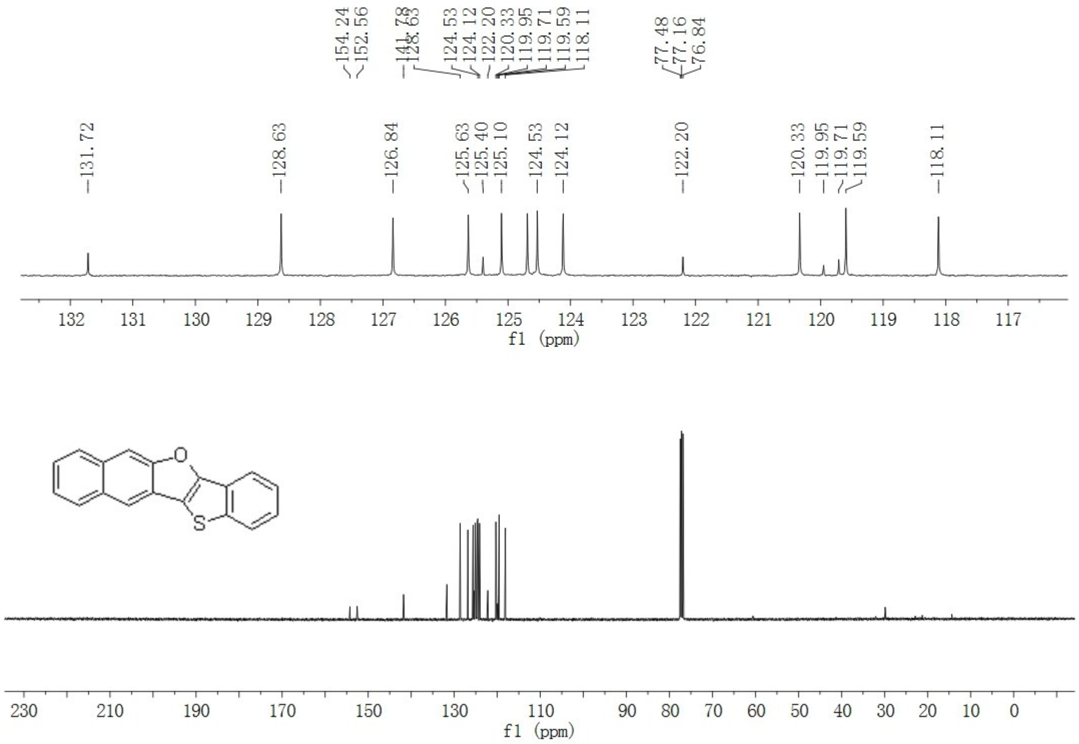
154.24
152.56
141.78
128.63
124.53
124.12
122.20
120.33
119.95
119.71
119.59
118.11
77.48
77.16
76.84
131.72
128.63
126.84
125.63
125.40
125.10
124.53
124.12
122.20
120.33
119.95
119.71
119.59
118.11
f1 (ppm)
S
O

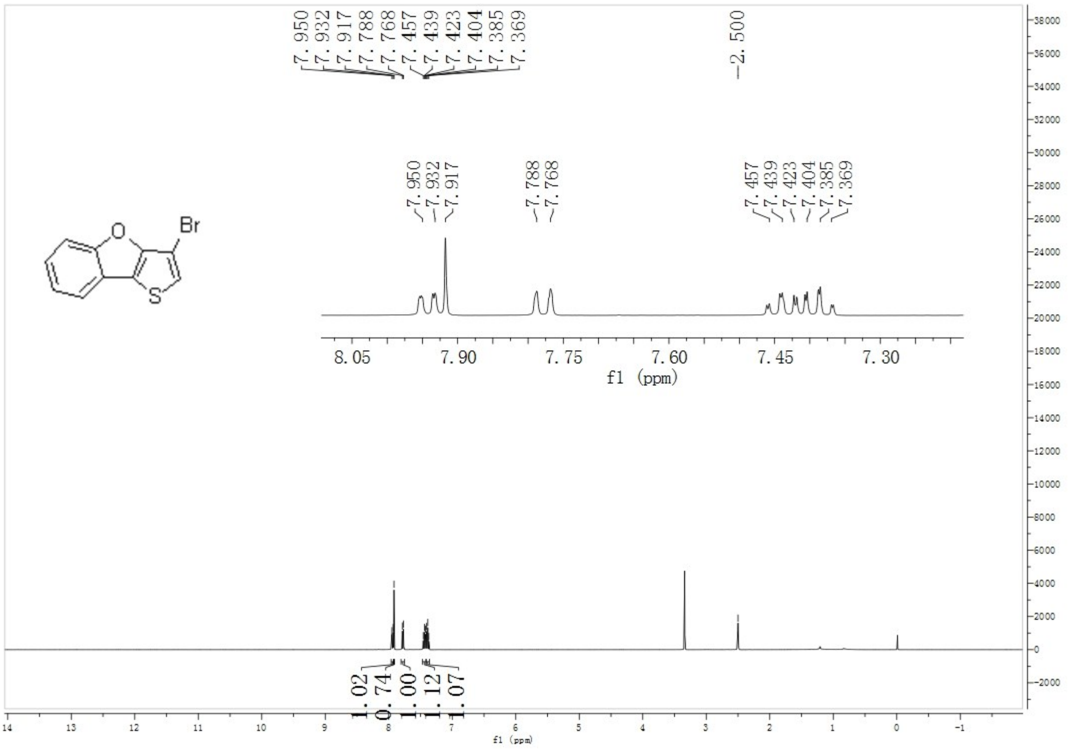

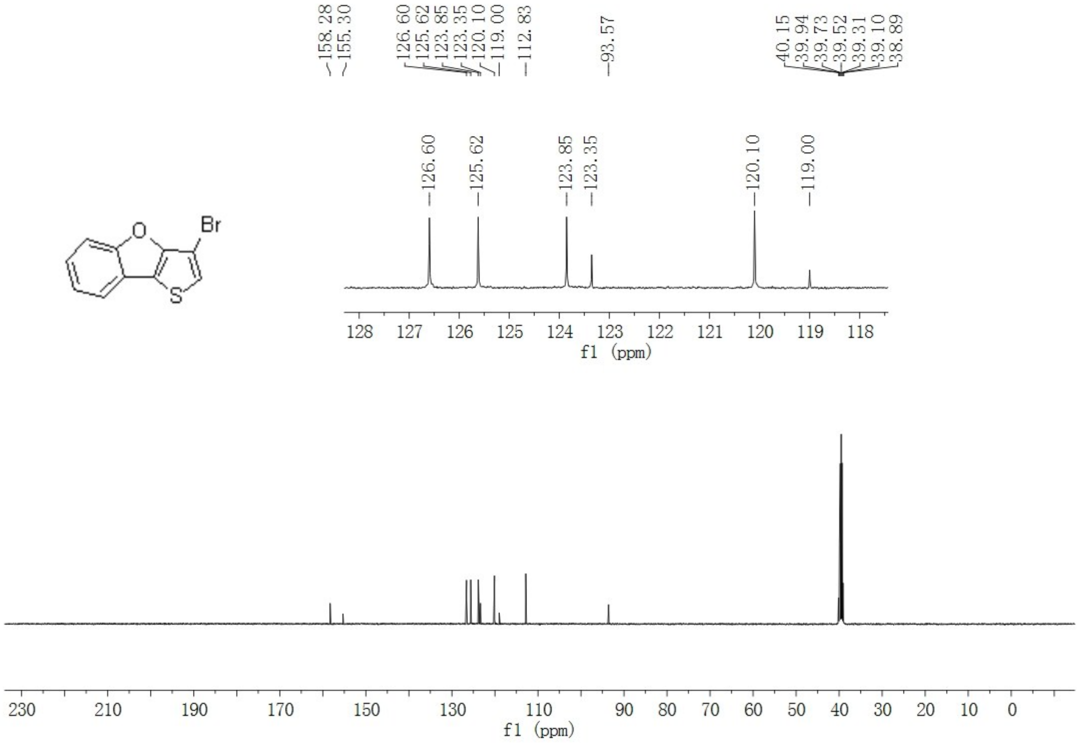

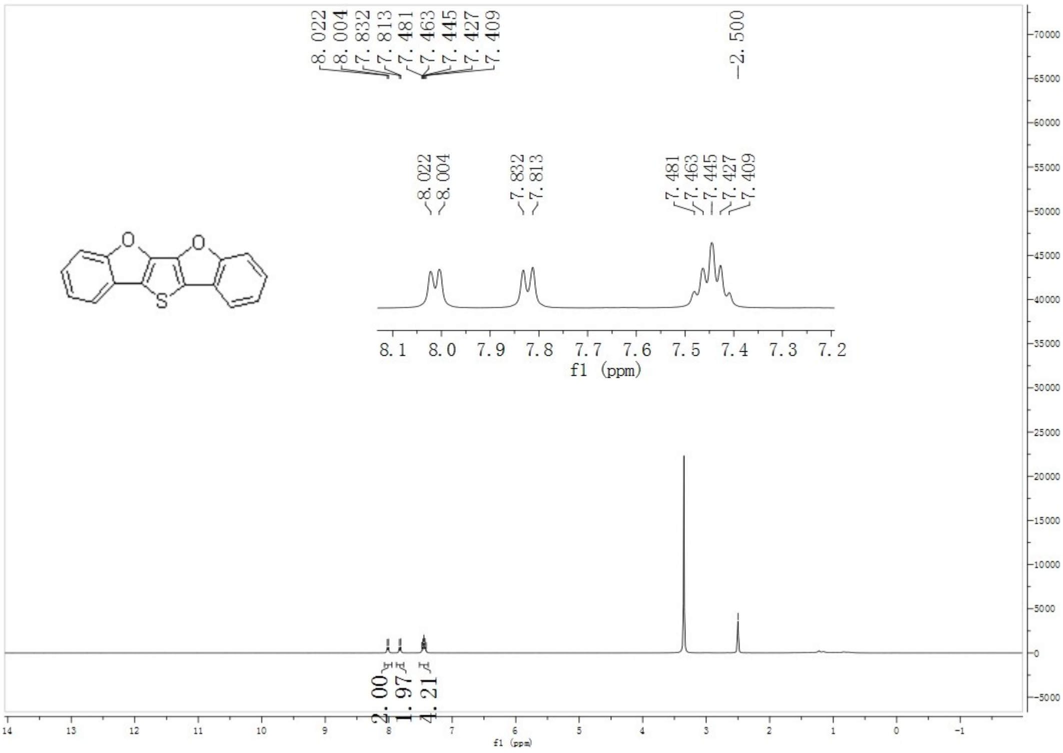
8.022
8.004
7.832
7.813
7.481
7.463
7.445
7.427
7.409
2.500
2.00
1.97
4.21
f1 (ppm)

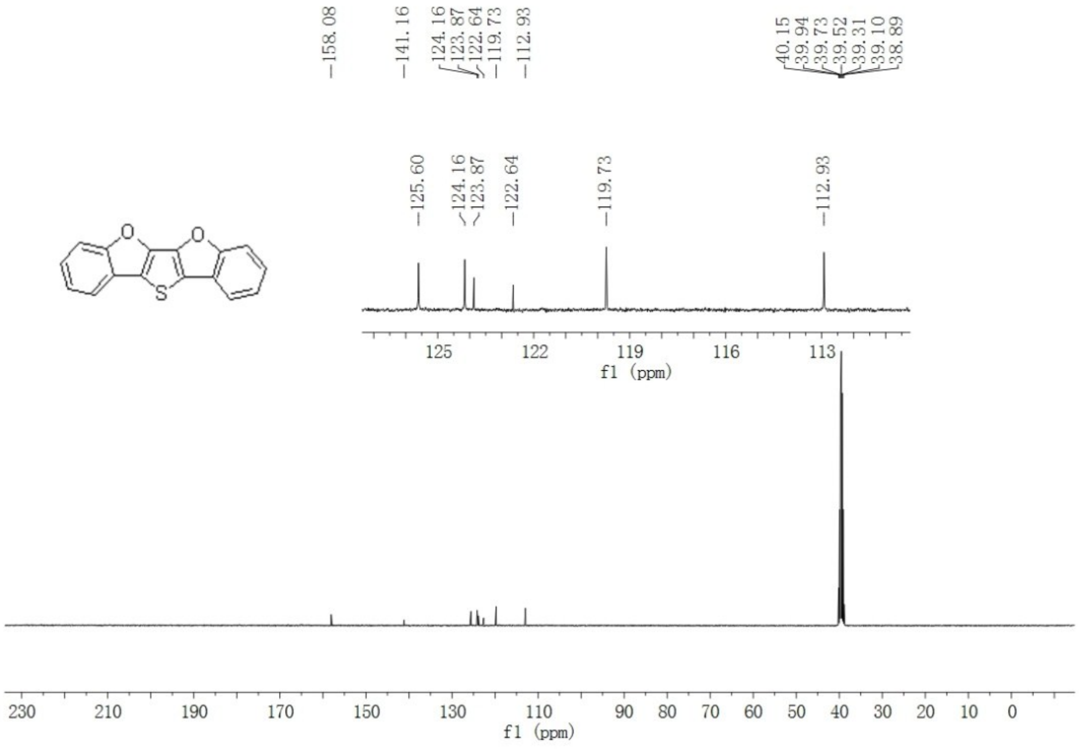
158.08
141.16
124.16
123.87
122.64
119.73
112.93
40.15
39.94
39.73
39.52
39.31
39.10
38.89
125.60
f1 (ppm)
